# Supplementary material for: A preregistered, open pipeline for early cerebral palsy risk assessment from infant videos
Source: Gigascience. 2026 Jan 20;15:giag003. doi: 10.1093/gigascience/giag003 (PMC13152017; doi:10.1093/gigascience/giag003)
Supplement: giag003_GIGA-D-24-00511_original_submission [file giag003_giga-d-24-00511_original_submission.pdf]

GigaScience

Assessing infant risk of cerebral palsy with video-based motion tracking

--Manuscript Draft--

**Manuscript Number:**	GIGA-D-24-00511
**Full Title:**	Assessing infant risk of cerebral palsy with video-based motion tracking
**Article Type:**	Research
**Funding Information:**	National Institute of Child Health and Human Development (1R01HD097686) — Dr. Konrad P Kording<br>Cerebral Palsy Foundation — Dr. Andrea F Duncan
**Abstract:**	Cerebral Palsy (CP) is a common (about 1 in 500 children) health condition caused by abnormal brain development that affects the ability to control movement. Early risk assessment happens through the General Movements Assessment (GMA), a test administered by trained clinicians at 3-4 months of age that has high predictive value for CP. With recent improvements in video-based motion tracking, automated risk assessment for CP based on the GMA is being explored. However, studies generally have used small datasets or were limited in terms of methodological rigor. Here we acquired a large dataset (1060 infants) of videos from a clinical population with elevated CP risk. In a preregistered pipeline using a lock-box set that was not used before algorithm submission we find that our machine learning predictions are highly predictive of the clinician-assessed GMA (AUC=0.79). Given its low cost, our video-based approach may be useful for clinical screening applications, particularly in low-resource settings.
**Corresponding Author:**	Melanie Segado<br>University of Pennsylvania School of Engineering and Applied Science<br>Philadelphia, UNITED STATES
**Corresponding Author Secondary Information:**	
**Corresponding Author's Institution:**	University of Pennsylvania School of Engineering and Applied Science
**Corresponding Author's Secondary Institution:**	
**First Author:**	Melanie Segado, PhD
**First Author Secondary Information:**	
**Order of Authors:**	Melanie Segado, PhD
	Laura Prosser, PT, PhD
	Andrea F Duncan, MD, MS
	Michelle J Johnson, PhD
	Konrad P Kording, PhD
**Order of Authors Secondary Information:**	
**Additional Information:**	
**Question**	**Response**
Are you submitting this manuscript to a special series or article collection?	No
**Experimental design and statistics**<br><br>Full details of the experimental design and	Yes

Powered by Editorial Manager® and ProduXion Manager® from Aries Systems Corporation

statistical methods used should be given in the Methods section, as detailed in our Minimum Standards Reporting Checklist. Information essential to interpreting the data presented should be made available in the figure legends.<br><br>Have you included all the information requested in your manuscript?	
**Resources**<br><br>A description of all resources used, including antibodies, cell lines, animals and software tools, with enough information to allow them to be uniquely identified, should be included in the Methods section. Authors are strongly encouraged to cite Research Resource Identifiers (RRIDs) for antibodies, model organisms and tools, where possible.<br><br>Have you included the information requested as detailed in our Minimum Standards Reporting Checklist?	Yes
**Availability of data and materials**<br><br>All datasets and code on which the conclusions of the paper rely must be either included in your submission or deposited in publicly available repositories (where available and ethically appropriate), referencing such data using a unique identifier in the references and in the "Availability of Data and Materials" section of your manuscript.<br><br>Have you have met the above requirement as detailed in our Minimum Standards Reporting Checklist?	No
If not, please give reasons for any omissions below.	Original dataset and extracted keypoints cannot be made openly available at this time to comply with ethics regulations.

Powered by Editorial Manager® and ProduXion Manager® from Aries Systems Corporation

as follow-up to "**Availability of data and materials**<br><br>All datasets and code on which the conclusions of the paper rely must be either included in your submission or deposited in publicly available repositories (where available and ethically appropriate), referencing such data using a unique identifier in the references and in the "Availability of Data and Materials" section of your manuscript.<br><br>Have you have met the above requirement as detailed in our Minimum Standards Reporting Checklist?<br><br>"	
GigaScience has policies and guidelines in place for the use of generative AI-writing tools such as ChatGPT. If you have used such writing tools to assist with writing the manuscript this must be declared and cited in the text. Authors should not list AI-writing tools and other AI-assisted technologies as an author or co-author and should acknowledge that they are fully responsible for text generated or refined by AI-writing tools.<p><br><br>A summary of use (particularly in the introduction or among methods) needs to be included at the end of the paper, and the outputs should also be included as a supplementary file hosted in GigaDB or other open repositories. Please read our guidelines for more information. <p><br><br>By submitting to GigaScience, you are aware of the journal's AI-writing tools policy, and if you have declared use of such tools below, you have acknowledged this where appropriate in your manuscript and have made a summary of use and outputs available. <p><br><b>AI-assisted writing tools have been used in the preparation of this manuscript?	No

Powered by Editorial Manager® and ProduXion Manager® from Aries Systems Corporation

Manuscript file created from Gigascience latex template
Click here to access/download;Manuscript;tjrntzpfzkndnqpdwtyhvnsjdvrxgcp

GigaScience, 2023, 1–8

doi: xx.xxxx/xxxx
Manuscript in Preparation
Paper

PAPER

Assessing infant risk of cerebral palsy with video-based motion tracking

Melanie Segado, PhD[1,2,5], Laura Prosser, PT, PhD[4,5*], Andrea F. Duncan, MD, MS[4,6], Michelle J. Johnson, PhD[1,7,8,9] and Konrad P. Kording, PhD[1,2]

[1]Department of Bioengineering, University of Pennsylvania, Philadelphia, PA, United States and [2]Department of Neuroscience, University of Pennsylvania, Philadelphia, PA, United State and [3]Department of Physical Therapy, The Children's Hospital of Philadelphia, Philadelphia, PA, USA and [4]Department of Pediatrics, Perelman School of Medicine, University of Pennsylvania, Philadelphia, PA, USA and [5]Division of Rehabilitation Medicine, The Children's Hospital of Philadelphia, Philadelphia, PA, USA and [6]Division of Neonatology and Department of Pediatrics, Children's Hospital of Philadelphia and [7]Department of Physical Medicine and Rehabilitation, University of Pennsylvania, Philadelphia, PA, USA and [8]Department of Mechanical Engineering and Applied Mechanics, University of Pennsylvania, Philadelphia, PA, USA and [9]Rehabilitation Robotics Lab, Perelman School of Medicine, University of Pennsylvania, Philadelphia, PA, USA

*prosserl@chop.edu

Abstract

Cerebral Palsy (CP) is a common (about 1 in 500 children) health condition caused by abnormal brain development that affects the ability to control movement. Early risk assessment happens through the General Movements Assessment (GMA), a test administered by trained clinicians at 3-4 months of age that has high predictive value for CP. With recent improvements in video-based motion tracking, automated risk assessment for CP based on the GMA is being explored. However, studies generally have used small datasets or were limited in terms of methodological rigor. Here we acquired a large dataset (1060 infants) of videos from a clinical population with elevated CP risk. In a preregistered pipeline using a lock-box set that was not used before algorithm submission we find that our machine learning predictions are highly predictive of the clinician-assessed GMA (AUC=0.79). Given its low cost, our video-based approach may be useful for clinical screening applications, particularly in low-resource settings.

Key words: Cerebral palsy; Risk assessment; Infant development; Movement analysis; Machine learning; Video analysis; Computer vision; Motor disorders; Predictive modeling; Pediatrics

Background

Cerebral Palsy (CP) is the most common cause of motor impairment leading to physical disability in children, affecting an estimated 2-3 out of 1000 infants globally [1]. In the USA alone, this results in approximately 1 million people living with impaired mobility due to CP at any given time, many of which have lifelong disability. Early detection and rehabilitation before two years of age is critical, as beginning rehabilitation within this sensitive period for neural plasticity and motor development is associated with functional outcomes [2, 3]. Atypical movement patterns that indicate a high risk of developing CP are reliably detectable through visual observation of movements by a trained physician at or before 10 weeks of age, but many infants are not evaluated by a physician until after severe, overt motor impairments have already developed. In practice, this means that CP is typically diagnosed between 6 to 24 months of age, which is near the end of the optimal window for intervention. There is, therefore, a need to develop automated early pre-screening tools that can detect atypical patterns of motor development before they progress to more severe impairment, allowing for more efficient

Compiled on: November 11, 2024.
Draft manuscript prepared by the author.

Key Points

- Developed a video-based predictive model for early detection of cerebral palsy (CP) in infants, addressing a critical gap in scalable pediatric risk assessment tools.
- Utilized a large, diverse dataset with a pre-registered lock-box test set, ensuring robustness and minimizing the risk of overfitting.
- Demonstrated that video-based movement metrics can reliably predict General Movements Assessment (GMA) scores, validating the model's clinical relevance.
- Achieved high generalizability, with performance sustained across unseen data, supporting potential for broad implementation in various clinical settings.
- Provided an open-source pipeline, facilitating rapid adoption and further development across clinical research and health technology communities.

use of costly medical resources and improved outcomes particularly in low resources settings.

CP risk is routinely assessed by clinicians based on visual observation of movements. One such assessment is the General Movement Assessments (GMA)[4], which is predictive of CP as early as 3 months of age based on the expert classification of spontaneous infant movements. It distinguishes between "typical" and "atypical" general movements (GMs), including the identification of "fidgety movements" (FMs) at 3-4 months, which are a precursor to coordinated, volitional movement. The absence of FMs at this age is 95% predictive of CP when combined with abnormal findings on brain MRI[2]. The GMA assessment is typically scored from video and considers characteristics of movement quality, variability, and complexity. If these relevant movement features can be reliably computed from videos, then algorithmic approaches for predicting infant risk from movement features should work robustly.

Many parallel efforts by various research groups are underway to automate GMA assessment using video-derived skeletal tracking [5, 6, 7, 8, 9, 10, 11, 12, 13, 14, 15, 16, 17, 18]. However, the potential for these approaches to scale beyond the dataset on which they were trained is currently limited. Existing models rely on hand-annotated, or custom fine-tuned models, which are specific to each research group's dataset. The advent of pre-trained vision transformers has enabled better feature extraction and multi-scale information fusion, leading to improved performance on data with occlusions and poses and joint or limb segment occlusions, both of which are common in spontaneous infant movement and cause significant issues for infant pose estimation algorithms[19, 20, 21, 22, 23, 18]. The combined advancements in deep learning, open datasets, and open-source tools have significantly improved the reliability and accuracy of pose estimation and tracking outcomes[24, 25]. Pre-trained vision transformers should be sufficiently good to capture the movement features that are relevant for clinical assessment, without the need for custom models that risk overfitting.

Existing video-based automated risk assessment models often perform well, but are limited either in terms of sample size, generalizability, or methodological rigor. For instance, Gao et al. [6] trained a transformer model on clips of hand-labeled movements and counted the proportion of video clips in their sample labeled as FMs after training6. This approach was highly effective at detecting FMs, however, as they noted this approach cannot be extended without the need for retraining on other hand-labeled segments. Others such as Ihlen et al. [26] found high levels of sensitivity and specificity, comparable to clinician GMA assessments, but the model relied on a backward prediction of over 900 features, raising the concern that the precise featurization may overfit to the specific dataset [26, 27]. Moreover, none of these models with promising results have been made fully openly available, limiting the extent to which generalization to a new dataset can be tested, and none employed a "lock-box" set (ie. held-out data points that were not used at any point during the hyperparameter optimization process), raising the possibility that results are overly optimistic [28]. There is, therefore, still a need to test whether the effects observed in the literature replicate in a large sample, with explainable features, and a pre-registered analysis pipeline.

We addressed these limitations using a large dataset of clinician-labeled videos from our institutions' United States CP Early Detection and Intervention Network site data. To assess risk based on video data we developed a classification pipeline. To compute accurate movement features, we started by selecting an open-source pose estimation algorithm that had high precision on our infant dataset. Out of the 2D pose estimates we, based on clinician feedback, computed 38 features that described posture, velocity, acceleration, left-right symmetry, and complexity of movements. We show that movement features can predict GMA scores in the largest infant dataset used to date, using an automated machine learning approach that limits bias in hyperparameter optimization, and a fully pre-registered pipeline (Figure 1).

Data Description

Collection of a large clinical dataset

Data were collected as part of standard clinical care by team members of the CHOP site of the US CP Early Detection and Intervention Network in a REDcap database between May 2019 and December 2023. This included the secure uploading of iPad- or iPhone-recorded videos, GMA scores and demographic information. Access to this clinical database was restricted to hospital staff. The GMA was administered in accordance with CHOP's participation in the Cerebral Palsy Foundation's Early Detection and Intervention network, who follow the international diagnostic guidelines 2. For all infants who were between 10-20 weeks post-term age (corrected for preterm birth, if applicable) at the time of a clinic visit, and whose parents or legal guardians agreed to video recording for clinical care, clinicians captured a 1-2 minute video of the infant lying in supine. This is the usual age for an infant's first visit with the Neonatal Follow-up Program high-risk infant follow-up clinic. Infants were observed in minimal attire for unobstructed visibility of the trunk, shoulders, and extremities to facilitate the observation of natural movements (typically wearing a diaper only. The use of pacifiers, toys, or engagement in communication with the infant during the assessment was prohibited and other distractions that could potentially influence the outcome were minimized. If patients missed their clinic visit during this time period, parents were instructed how to capture the video and provided a link to upload the video into REDcap.

Clinical evaluation

The evaluation process was characterized by the involvement of over 20 clinicians, including physical and occupational therapists, nurse practitioners and physicians, who had completed training

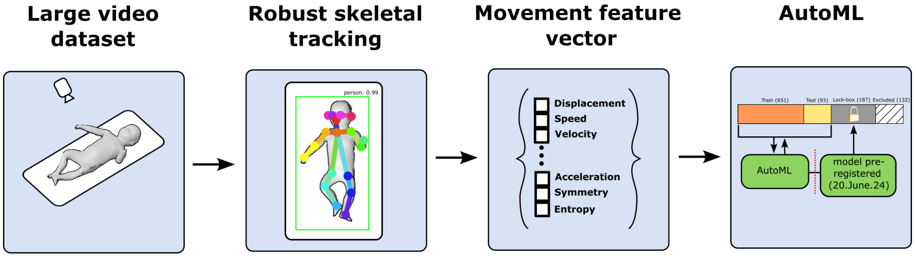

Figure 1. Process for rigorous evaluation of automated clinical score prediction. Each step of the model development process was pre-registered. Lage video dataset had 1060 infants, 132 were excluded from the dataset for meeting one or more medical exclusion criteria prior to any analysis. Training(651)/Test(93) infant videos and "Lock-box" videos (187) were pre-registered prior to pose-tracking algorithm selection and movement feature computation. Features were pre-registered prior to model training. Model was pre-registered prior to testing on lock-box

in GMA assessment by the General Movements Assessment Trust, several with additional advanced training. The GMA score (FMs present, absent or abnormal) was determined after adjudication by two independent clinician reviewers. In instances where disparities in assessment arose, a third evaluator was consulted. Videos eliciting uncertainty were further examined in weekly meetings convened by the site's early detection team.

Patient characteristics

To ask how well we could predict GMA score from clinician-selected movement features in a large sample, we recruited 1060 participants from the Children's Hospital of Pennsylvania. The sample of 1060 infants was sex-balanced, with 55% girls, 45% boys, and 1% Unknown/Unspecified. It also comprised a wide range of race/ethnicities, including White (38%), Black/African American (35%), Other (10%), and all other responses (Multi-Racial, Asian, Indian, American Indian/Alaskan, Native Hawaiian, and Not Reported/Unknown/Other) making up 16% of responses. Of reported ethnicities, 8% were Hispanic/Latino.

Each of the video recordings used for analysis was determined evaluable by the clinical reviewers. In cases where infants were distracted during the recording session, a second video was obtained. Only the final videos used for GMA scoring were considered in this dataset. Average infant age was mean corrected age 14.6 weeks (+/- 2.1 weeks). Of the 931 infants that remained after applying exclusion criteria, 820 were scored as having FMs (normal) and 105 infants were scored with having absent FMs. The remaining six infants were scored as having abnormal movements and were excluded from further analyses.

Inclusion/Exclusion criteria

For children still hospitalized at the time of the fidgety-aged GMA, Early Detection Team members captured the videos in the hospital as part of standard care. Exclusions were applied to intubated patients, those under the influence of sedation medications, within a week post-operative, on ECMO support, or diagnosed with myelomeningocele. The full dataset comprised 1060 infants. 129 were then excluded for meeting one or more exclusion criteria listed above. Six infants with a GMA score of 3 ("atypical fidgety") were also excluded, since there were not enough infants in this group for model training/testing, and movement patterns differ from those of "absent fidgety" infants. The remaining 931 videos were split into an analysis set (744) and a lock box holdout set (187). The analysis set was further split into train/val/test sets (558, 93, 93), each of which had a 12% representation of the "absent fidgety" movement type. The splits were stratified to preserve the ratios of boy/girl infants, as well as age, and race/ethnicity. There was a total recording duration of 60-120 s per infant.

Data Availability

In accordance with ethics guidelines, identifiable data (videos) can only be processed on-site at the Children's Hospital of Pennsylvania. Pose estimate time-series data will be made available upon reasonable request. Researchers wishing to access the data should contact the corresponding author. However, computed movement features have been made available on the OSF Pre-registration site.

Analyses

Developing a pipeline for robust skeletal tracking

Performance and Validation of Skeletal Tracking Pipeline

Infant videos pose unique challenges for pose-estimation algorithms due to frequent irregular body poses, the presence of body-like objects (e.g., toys or cartoons), and high levels of self-occlusion. Conventional algorithms, such as OpenPose[29], often fail in such conditions, leading to unreliable pose estimates [22]. Fine-tuning is often required to improve accuracy on each infant dataset. However, we found that a pre-trained vision transformer, ViTPose-H [19], performed better than fine-tuned alternatives [30, 31, 32, 29, 33], obviating the need for manual finetuning.

Generalizability Testing

The robustness and generalizability of ViTPose-H were validated through iterative review of pose estimates by clinicians trained in the General Movements Assessment (GMA). This model was further tested on two fully out-of-sample infant datasets (n = 300, ages 0–4 months), achieving consistent skeletal tracking performance. This provides a scalable and reliable solution for converting infant videos into skeletal tracking data, facilitating large-scale analysis of movement patterns.

Feature Relevance and Clinical Interpretation

A set of 38 kinematic features was selected based on clinician input [34, 35], designed to capture the displacement, speed, velocity, acceleration, and entropy of key body parts, specifically wrists, ankles, elbows, and knees (Figure 3). These features were chosen as they represent clinically relevant movement patterns necessary for visual scoring of the GMA [4]. By excluding any direct features related to GMA-specific fidgety movements, we aimed to ensure generalizability and avoid overfitting the model to the clinical dataset.

Model performance and validation

Generalizability and robustness of feature vector for risk prediction

Our initial analysis found considerable overlap in the 38 selected features for infants with and without fidgety movements (Figure 3), suggesting that no individual feature alone could distinctly differ-

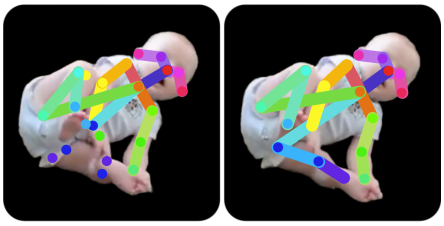

Figure 2. Improvements in skeletal tracking with pre-trained vision transformers. Fine-tuned OpenPose algorithm (Left) still struggles with issues frequently encountered in infant videos such as occlusions and complex poses. Modern approaches, notably those leveraging pretrained vision transformers such as ViTPose-H (Right), are more robust. Occluded keypoints are not included in pose estimate using previous algorithms (Left). Transformer-based approach (Right) learns skeletal structure from adult human data and can infer occluded keypoints even on infants.

entiate high-risk infants. However, the aggregated feature vector was sufficient to provide a predictive signal for GMA scores. Using the feature vector, the model achieved an AUC-ROC of 0.72 on the validation set. Five-fold cross-validation with six random seeds resulted in an average AUC-ROC of 0.73 ± 0.05, verifying model robustness and generalizability within the training dataset.

Rigorous methods to prevent overfitting

To ensure that the model's performance was unbiased and generalizable, a lockbox test set of 187 infants (22 with absent fidgety movements) was randomly selected before model training. This lockbox data was only accessed after pre-registering all features, preprocessing steps, and algorithms. Testing on the lockbox set yielded an AUC-ROC of 0.79 (Figure 4), closely aligned with the cross-validation performance, indicating minimal overfitting. This rigorous validation suggests that the model generalizes well to unseen data, providing a reliable and reproducible approach for identifying infants at high risk for CP.

Discussion

Here we have developed an ML algorithm to predict GMA score (a strong indicator of CP risk) from video-based pose estimates using rigorous methods. We used an exceptionally big sample (training set: 558, overall >1000 infants), a simple and explainable movement-based feature vector, and we pre-registered each step of the process prior to testing on a randomly-selected "lock-box" set of 187 infant videos. We found that our algorithm performs well (AUC-ROC 0.79). We have utilized an AutoML approach to minimize the risk of overfitting. We have further minimized the risk of false positive data using a lock-box set and pre-registered our analysis before running it. We have made data and algorithms publicly available on the OSF pre-registration site and GitHub. Based on our rigorous pre-registered approach with a lock-box set we can be confident that we did not do any overfitting and that it will generalize well to other datasets.

While the GMA has been shown to have a high level of sensitivity and specificity in clinical settings, we did not predict the main important target future outcome – diagnosis of CP – as long-term outcomes were not available at the time of model training. Instead we predicted GMA, a clinician powered risk measure. This is common throughout the automated CP risk prediction literature, with multiple research groups focusing on predicting GMA score, or detecting Fidgety Movements directly, as opposed to predicting CP diagnosis. This approach is not ideal, as it introduces an additional source of noise from potential human error during assessment, in addition to the noise inherent in the GMA assessment itself. FMs, while highly indicative, are still not a perfect biomarker for CP and multiple items are necessary for CP diagnosis (biomarkers, clinical history, functional motor assessment and neurological assessment). Over-reliance on FMs risks missing other, perhaps more indicative features or combinations of features that are not readily apparent. Moreover, the extremely low prevalence of Abnormal FMs makes training a model that captures this movement type infeasible, meaning that some infants at high risk are not accounted for in models trained only to detect FMs (or their absence). Future efforts should focus directly on predicting CP outcomes.

In order to detect subtle movement differences, it is likely that our features are suboptimal. The clinician-selected movement features offer only a coarse description of movement, whereas we know from the clinical literature that the difference between infants whose movements are typically developing and those that are not is often subtle. This is especially true if we push towards early-prediction, before the 3-4 months where the difference between movements is captured by the GMA, or push towards models that work for widespread pre-screening in the general population. Many efforts have been made to identify a precise featurization using machine learning [14, 15], however all of these efforts risk overfitting since the size of the datasets is very small. By contrast, deep learning models trained on very large datasets of infant movements, as well as important context like clinical history and other assessments) promise to give more precise feature vectors that capture these subtle differences, boosting performance of the GMA prediction model and enabling even earlier prediction of CP.

The wide range of ages at which CP is typically diagnosed reflects the fact that less severe movement deficits are often not evident to untrained observers until later in an infant's development when they start missing major milestones, whereas indicators of more severe impairment may be evident to clinicians (and caregivers) much earlier. The infants included in the model all spent time after birth in the Neonatal Intensive Care Unit (NICU), meaning that they were already at an elevated risk of CP. This limitation is prevalent throughout the automated CP detection literature [8, 10, 11, 16], since collecting videos of infants for the purposes of training a ML prediction model is most feasible in a hospital setting. As such the movements that distinguish the two groups in our sample may not be representative of infants from the general infant population. Future work can address this by including infants from the general population as well as those from the higher risk NICU cohort. However, other people have shown that movement features can be used to predict GMA scores in at-home videos of infants that are not at high risk15, so the approach should generalize if trained on the bigger sample. This should be imminently feasible now that we have released a pose estimation and preprocessing pipeline that is open, easy to share, and does not require fine-tuning across videos of infants in different settings and at different ages.

We have shown that a simple movement-based automated prediction approach works in an exceptionally big sample (>1000 infants). This is significant since models are often trained on very small datasets (< 50) which risk overfitting and limit generalizability. Given that the clinician-selected movement features can predict GMA score in such a large sample, we have a strong indication that models that include even more data from a wider sample of infants, and more precise features, should perform even better.

To further minimize the risk of overfitting, we pre-registered all of our analysis pipeline, and our model, prior to testing on a "lock-box" set. Both of these steps minimize the risk of selecting features that are unique to a specific dataset, or selecting features that are biased by the pre-processing steps taken prior to model training. Such bias results in many machine learning models being overfit, and failing when applied to an out-of-sample dataset. Pre-registration and lock-box testing greatly reduce this risk, and should be standard for developing machine learning pipelines in clinical contexts.

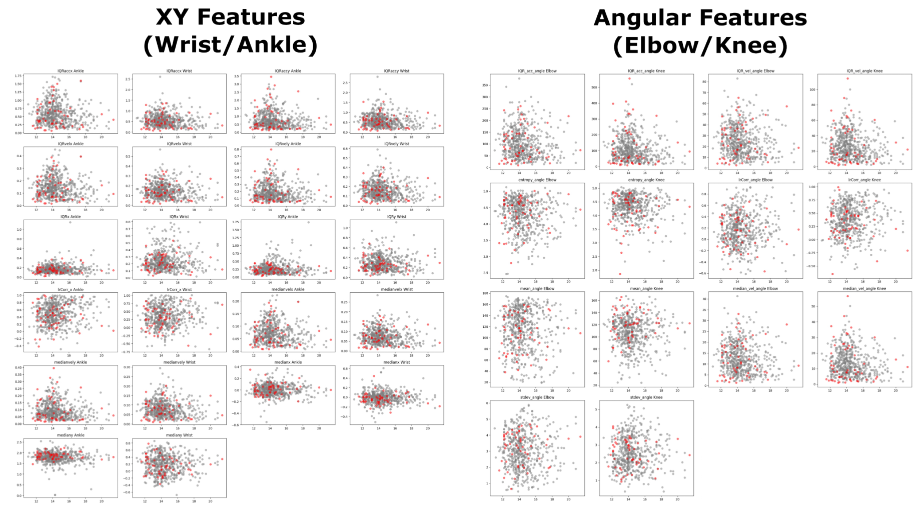

Figure 3. Individual features are highly correlated. Clinician-selected features, including XY features of the wrists/ankles (Left) and angular features of the elbows/knees (Right), which are typically used for human assessment of risk are highly overlapping for Fidgety (Grey) and Absent Fidgety (Red) movement types, with no individual feature clearly predicting GMA score.

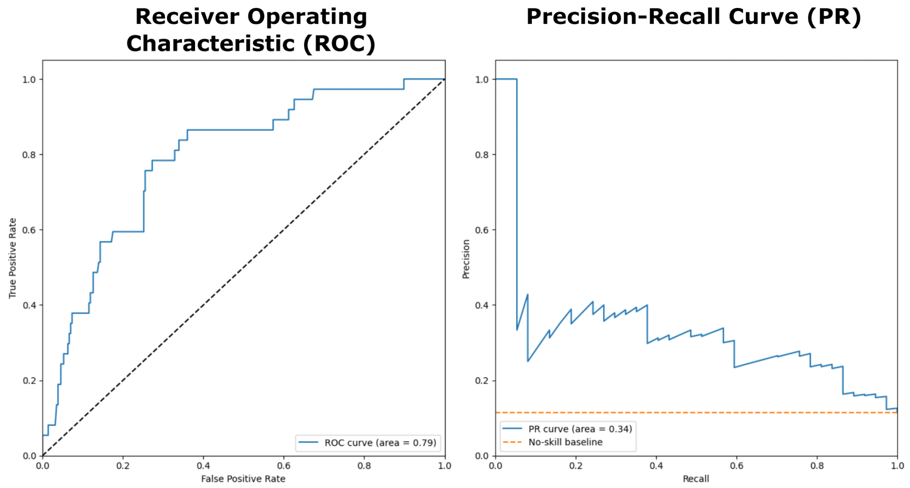

Figure 4. Model generalizes to lock-box set. Classifier trained on clinician-selected features using vanilla auto-sklearn shows a high AUC-ROC of 0.79 (Left) and Precision-Recall of 0.34 (Right) on lock-box set of 187 infants, having 12% representation of absent fidgety movement type. True positive rate is equal to the Sensitivity of the classifier, False positive rate is equal to 1-Specificity.

Potential implications

All of the methods used are ethologically doable on a phone camera. Data collection was done using a hand-held iPad camera, and the pose estimation pipeline was tested both on videos from this dataset, as well as 300 infant videos from other datasets to ensure generalizability across various contexts. While video-based pose estimation for infant movement estimation is common in the automated detection literature, each site typically uses their own custom fine-tuned algorithm with a post-processing pipeline that is tailored to their specific dataset. The pre-trained vision transformer we used was not fine-tuned on any of the infant videos, and as such should work equally well at other clinical sites and on at-home videos. Training on datasets across multiple sites and various contexts should now be possible, and drive towards a globally available at-home pre-screening tool.

Overall we have shown that advances in pose estimation now make it entirely realistic to get precise movements from infant videos without the need for any specialized camera setup or fine-tuning. We have shown that movement features derived from these pose estimates are sufficient for predicting GMA scores in a very large sample, and that our model generalizes well to unseen data. This holds tremendous potential for the creation of a global prescreening tool, especially if we boost performance using deep-learned feature vectors and train on more data, including from many infants across various contexts, and includes CP outcomes as opposed to just clinical scores.

Methods

Developing a Pipeline for Robust Skeletal Tracking

Selecting a pose estimation algorithm

To estimate infant pose from monocular hand-held video, we implemented a top-down 2D pose estimation pipeline using tools from the open-source library OpenMMLab. MMDetection was used for infant detection [24], and MMPose was used for 2D pose estimation [24]. Infant detection was performed using an RTMDet [36] model pre-trained on the Common Objects in Context (COCO) dataset [37]. 2D frame-wise pose estimation was carried out using ViTPose [19], a 10B parameter vision transformer, selected for its domain adaptability.

Processing keypoint timeseries data

In each frame, only the highest-confidence detection was used, and frames with keypoint confidence scores below 0.8 were excluded. As in Chambers et al. [5], missing frames were linearly interpolated, outliers removed with a rolling-median filter (1-second window), and data smoothed with a rolling-mean filter (1-second window).

Dataset Split and Pre-registration

Following pose estimation, infant IDs were divided into training, validation, and lockbox test sets using a stratified split to preserve a 12% representation of the absent fidgety movement type, as well as the ratio of male/female infants and race/ethnicity as described in the dataset. The video IDs corresponding to each split were pre-registered prior to conducting further analyses, ensuring a clear separation between training, validation, and test datasets, and the lockbox test set.

Kinematic Feature Computation

After pre-registration and pre-processing, a set of 38 kinematic features were computed from the smoothed keypoint timeseries using open-source Python code [38], adapted from previous work [5]. These features captured displacement, speed, velocity, acceleration, and entropy of the extremities (wrists and ankles) and joint angles (elbows and knees). No specific features related to General Movements Assessment (GMA) fidgety movements (FMs) were included to minimize the risk of overfitting to the clinical dataset. Pose estimation was conducted on CHOP high-performance computing servers, ensuring compliance with ethics guidelines by restricting access to CHOP staff only.

Model Training

Feature Selection and Pre-registration

A binary classifier was trained to predict infants with a GMA score of "absent fidgety," which indicates a higher risk of developing cerebral palsy (CP) [2, 3, 4, 39, 40]. To reduce the risk of overfitting, feature selection was conducted in consultation with clinicians prior to any data analysis and was pre-registered in 2018 [34, 35]. A set of 38 movement features was derived from expert clinician input to ensure clinical relevance.

Model Selection and Training

Model selection and hyperparameter optimization were carried out using the Auto-sklearn 2.0 package [41, 42], with the "vanilla Auto-sklearn" setting. This configuration limited the ensemble size to one, ensuring that the model with the best validation performance was selected. Balanced accuracy was chosen as the optimization metric due to the class imbalance (approximately 10:1) [43]. A meta-feature-free portfolio was used for efficient meta-learning, and training/validation splits were managed with successive halving. Cross-validation with five folds was employed to validate model generalizability across different training/validation splits, and the resulting model was pre-registered on May 22, 2024, before testing on the lockbox test set [34].

Movement Feature Vector Computation

The feature vector, designed to represent each infant's movement characteristics, was computed from outputs of the skeletal tracking pipeline. This feature vector, based on previously established CP risk-assessment pipelines [34, 35], included 38 movement features. All features were selected and pre-registered before collecting infant video data to eliminate overfitting risks. Feature computation was automated using custom Python code available on GitHub [38].

Availability of source code and requirements

The source code for skeletal tracking, feature computation, and classifier training has been made available on GitHub at https://doi.org/10.5281/zenodo.14042732. The original feature computation code, from which this work is derived, can be found at https://github.com/quietscientist/Infant_movement_assessment.

Data availability

The data set supporting the results of this article is available on the OSF repository and contains participant IDs, data splits, movement features, and clinical scores [34]. In accordance with ethics guidelines, identifiable data (videos) can only be processed onsite at the Children's Hospital of Pennsylvania. De-identified keypoint timeseries may be made available upon reasonable request.

Declarations

List of Abbreviations

AUC-ROC Area Under Receiver Operating Characteristic

CHOP Children's Hospital of Pennsylvania
COCO Common Objects in Context
CP Cerebral Palsy
ECMO Extracorporeal Membrane Oxygenation
FM Fidgety Movement
GM General Movement
GMA General Movements Assessment
ML Machine Learning
MRI Magnetic Resonance Imaging
NICU Neonatal Intensive Care Unit

Ethical Approval

Ethical approval for this study was provided by the University of Pennsylvania (Penn) Institutional Review Board (IRB Protocol Number: 833180), acting as the single IRB or record and a subsequent reliance agreement between Penn and the Children's Hospital of Philadelphia (CHOP) Institutional Review Board (IRB Protocol Number: 19-016641).

Consent for publication

The infant image used in Figure 2 to illustrate algorithm performance is a video frame taken from the YouTube8M dataset, the use of which is permitted under CC BY 4.0

Competing Interests

The author(s) declare that they have no competing interests.

Funding

This work was funded by an NIH-NICHD grant (Project#: 1R01HD097686, PIs: Johnson, Michelle J. and Kording, Konrad P.) and the clinical Early Detection Trial data collection was supported in part by the Cerebral Palsy Foundation.

Author's Contributions

Konrad P. Kording, Michelle J. Johnson, Laura Prosser, and Melanie Segado were responsible for conceptualization of the study aims. Data curation was performed by Andrea F. Duncan, Laura Prosser, and Melanie Segado. Melanie Segado conducted the formal analysis and developed the software. Funding acquisition was led by Konrad P. Kording, Michelle J. Johnson, and Laura Prosser. Data collection and clinical evaluation were carried out by Andrea F. Duncan and Laura Prosser. Methodology was established by Konrad P. Kording and Melanie Segado, with input from Laura Prosser and Michelle J. Johnson. The original draft was written by Melanie Segado and Konrad P. Kording, and all authors contributed to the review and editing of the manuscript.

Acknowledgements

The authors would like to thank Felipe Parodi for help implementing the pose estimation pipeline, and O. Francis Sowande for iterative testing on out-of-sample data. They would also like to thank Julie Skorup, , PT, DPT, PCS and Audrey J Wood, MS, PT, PCS for validation of the skeletal tracking outputs.

References

1. McIntyre S, Goldsmith S, Webb A, et al. Global prevalence of cerebral palsy: A systematic analysis. Dev Med Child Neurol 2022;64(12):1494–1506.
2. Novak I, Morgan C, Adde L, et al. Early, Accurate Diagnosis and Early Intervention in Cerebral Palsy: Advances in Diagnosis and Treatment. JAMA Pediatr 2017;171(9):897–907.
3. Herskind A, Greisen G, Nielsen JB. Early identification and intervention in cerebral palsy. Dev Med Child Neurol 2015;57(1):29–36.
4. Einspieler C, Prechtl HFR. Prechtl's assessment of general movements: A diagnostic tool for the functional assessment of the young nervous system. Ment Retard Dev Disabil Res Rev 2005;11(1):61–67.
5. Chambers C, Seethapathi N, Saluja R, et al. Computer Vision to Automatically Assess Infant Neuromotor Risk. IEEE Trans Neural Syst Rehabil Eng 2020;28(11):2431–2442.
6. Gao Q, Yao S, Tian Y, et al. Automating General Movements Assessment with quantitative deep learning to facilitate early screening of cerebral palsy. Nat Commun 2023;14(1):8294.
7. Adde L, Brown A, van den Broeck C, et al. In-Motion-App for remote General Movement Assessment: a multi-site observational study. BMJ Open 2021;11(3):e042147.
8. Hashimoto Y, Furui A, Shimatani K, et al. Automated Classification of General Movements in Infants Using a Two-stream Spatiotemporal Fusion Network. arXiv 2022;http://arxiv.org/abs/2207.03344, published online July 4, 2022. Accessed September 27, 2023.
9. Groos D, Adde L, Aubert S, et al. Development and Validation of a Deep Learning Method to Predict Cerebral Palsy From Spontaneous Movements in Infants at High Risk. JAMA Netw Open 2022;5(7):e2221325.
10. Irshad MT, Nisar MA, Gouverneur P, Rapp M, Grzegorzek M. AI Approaches towards Prechtl's Assessment of General Movements: A Systematic Literature Review. Sensors 2020;20(18):5321.
11. Kwong AKL, Doyle LW, Olsen JE, et al. Parent-recorded videos of infant spontaneous movement: Comparisons at 3–4 months and relationships with 2-year developmental outcomes in extremely preterm, extremely low birthweight and term-born infants. Paediatr Perinat Epidemiol 2022;36(5):673–682.
12. Morais R, Le V, Morgan C, et al. Robust and Interpretable General Movement Assessment Using Fidgety Movement Detection. IEEE J Biomed Health Inform 2023;p. 1–12.
13. Nguyen-Thai B, Le V, Morgan C, Badawi N, Tran T, Venkatesh S. A Spatio-temporal Attention-based Model for Infant Movement Assessment from Videos. IEEE J Biomed Health Inform 2021;25(10):3911–3920.
14. Passmore E, Kwong AL, Greenstein S, et al. Automated identification of abnormal infant movements from smart phone videos. PLOS Digit Health 2024;3(2):e0000432.
15. Redd CB, Karunanithi M, Boyd RN, Barber LA. Technology-assisted quantification of movement to predict infants at high risk of motor disability: A systematic review. Res Dev Disabil 2021;118:104071.
16. Silva N, Zhang D, Kulvicius T, et al. The future of General Movement Assessment: The role of computer vision and machine learning – A scoping review. Res Dev Disabil 2021;110:103854.
17. Spittle AJ, Olsen J, Kwong A, et al. The Baby Moves prospective cohort study protocol: using a smartphone application with the General Movements Assessment to predict neurodevelopmental outcomes at age 2 years for extremely preterm or extremely low birthweight infants. BMJ Open 2016;6(10):e013446.
18. Ostadabbas S, Fine-tuned Domain-adapted Infant Pose (FiDIP); 2023. https://github.com/ostadabbas/Infant-Pose-Estimation, published online August 17, 2023. Accessed September 6, 2023.

19. Xu Y, Zhang J, Zhang Q, Tao D. ViTPose++: Vision Transformer for Generic Body Pose Estimation. arXiv 2023;Published online December 14, 2023.
20. Liu W, Bao Q, Sun Y, Mei T. Recent Advances of Monocular 2D and 3D Human Pose Estimation: A Deep Learning Perspective. ACM Comput Surv 2022;55(4):80:1–80:41.
21. Wei K, Kording KP. Behavioral tracking gets real. Nat Neurosci 2018;21(9):1146–1147.
22. Seethapathi N, Wang S, Saluja R, Blohm G, Kording KP, Movement science needs different pose tracking algorithms; 2019. Published online July 23, 2019.
23. Hesse N, Bodensteiner C, Arens M, Hofmann UG, Weinberger R, Schroeder AS. Computer Vision for Medical Infant Motion Analysis: State of the Art and RGB-D Data Set. In: Computer Vision - ECCV 2018 Workshops Springer International Publishing; 2018. .
24. Contributors M, OpenMMLab Pose Estimation Toolbox and Benchmark; 2020. https://github.com/open-mmlab/mmpose.
25. Contributors M, OpenMMLab Detection Toolbox and Benchmark; 2018. https://github.com/open-mmlab/mmdetection.
26. Ihlen EAF, Støen R, Boswell L, et al. Machine Learning of Infant Spontaneous Movements for the Early Prediction of Cerebral Palsy: A Multi-Site Cohort Study. J Clin Med 2020;9(1):5.
27. Powell M, Hosseini M, Collins J, et al, I Tried a Bunch of Things: The Dangers of Unexpected Overfitting in Classification; 2020. Published online February 14, 2020.
28. Hosseini M, Powell M, Collins J, et al. I tried a bunch of things: The dangers of unexpected overfitting in classification of brain data. Neurosci Biobehav Rev 2020;119:456–467.
29. Cao Z, Hidalgo G, Simon T, Wei SE, Sheikh Y. OpenPose: Realtime Multi-Person 2D Pose Estimation Using Part Affinity Fields. IEEE Trans Pattern Anal Mach Intell 2021;43(1):172–186.
30. Wang W, Xie E, Li X, et al. PVT v2: Improved baselines with Pyramid Vision Transformer. Comput Vis Media 2022;8(3):415–424.
31. Mathis A, Mamidanna P, Cury KM, et al. DeepLabCut: markerless pose estimation of user-defined body parts with deep learning. Nat Neurosci 2018;21(9):1281–1289.
32. Pereira TD, Tabris N, Matsliah A, et al. SLEAP: A deep learning system for multi-animal pose tracking. Nat Methods 2022;19(4):486–495.
33. Toshev A, Szegedy C. DeepPose: Human Pose Estimation via Deep Neural Networks. In: 2014 IEEE Conference on Computer Vision and Pattern Recognition; 2014. p. 1653–1660.
34. Segado M, Update: Predicting clinical assessments of infants' risk of neuromotor disease from 2-dimensional videos; 2023.
35. Chambers C, Predicting clinical assessments of infants' risk of neuromotor disease from 2-dimensional videos; 2018.
36. Lyu C, Zhang W, Huang H, et al, RTMDet: An Empirical Study of Designing Real-Time Object Detectors; 2022. https://arxiv.org/abs/2212.07784.
37. Lin TY, Maire M, Belongie S, et al, Microsoft COCO: Common Objects in Context; 2015.
38. Segado M, Chambers MJKK, Seethapathi N, Saluja R, Prosser L, Infant Movement Assessment: Update; 2024. https://github.com/quietscientist/Infant_movement_assessment.
39. Ferrari F, Cioni G, Einspieler C, et al. Cramped Synchronized General Movements in Preterm Infants as an Early Marker for Cerebral Palsy. Arch Pediatr Adolesc Med 2002;156(5):460–467.
40. Einspieler C, Yang H, Bartl-Pokorny KD, et al. Are sporadic fidgety movements as clinically relevant as is their absence? Early Hum Dev 2015;91(4):247–252.
41. Feurer M, Klein A, Eggensperger K, Springenberg J, Blum M, Hutter F. Efficient and Robust Automated Machine Learning. In: Advances in Neural Information Processing Systems, vol. 28 Curran Associates, Inc.; 2015. https://proceedings.neurips.cc/paper_files/paper/2015/file/11d0e6287202fced83f79975ec59a3a6-Paper.pdf.
42. Feurer M, Eggensperger K, Falkner S, Lindauer M, Hutter F, Auto-Sklearn 2.0: Hands-free AutoML via Meta-Learning; 2022.
43. Pedregosa F, Varoquaux G, Gramfort A, et al. Scikit-learn: Machine Learning in Python. J Mach Learn Res 2011;12:2825–2830.
