# Supplementary material for: A preregistered, open pipeline for early cerebral palsy risk assessment from infant videos
Source: Gigascience. 2026 Jan 20;15:giag003. doi: 10.1093/gigascience/giag003 (PMC13152017; doi:10.1093/gigascience/giag003)
Supplement: giag003_GIGA-D-24-00511_Revision_1 [file giag003_giga-d-24-00511_revision_1.pdf]

GigaScience

A Pre-Registered, Open Pipeline for Early Cerebral Palsy Risk Assessment from Infant Videos

--Manuscript Draft--

**Manuscript Number:**	GIGA-D-24-00511R1
**Full Title:**	A Pre-Registered, Open Pipeline for Early Cerebral Palsy Risk Assessment from Infant Videos
**Article Type:**	Technical Note
**Funding Information:**	National Institute of Child Health and Human Development (1R01HD097686) — Dr. Konrad P Kording<br>Cerebral Palsy Foundation — Dr. Andrea F Duncan
**Abstract:**	Cerebral Palsy (CP), affecting approximately 1 in 500 children due to abnormal brain development, impacts movement control. Early risk assessment via the General Movements Assessment (GMA) at 3-4 months is highly predictive for CP but relies on trained clinicians. Machine-learning-based approaches for predicting GMA score from video have shown considerable promise, but are not openly available and rely on fine-tuned pre-processing steps, hand-crafted feature sets, and experimenter-driven hyperparameter selection. This, combined with strict privacy constraints on sharing data, limits the extent to which models can be trained and tested across datasets, thus reducing clinical impact. There is therefore a need to develop approaches that will work across different datasets to enable multi-site dataset aggregation and model training. To address this gap, we developed an end-to-end pipeline that uses off-the-shelf pose estimation, general-purpose feature extraction, and automated machine learning—none of which are tuned to a specific dataset. We applied this approach to a newly generated large dataset of 1063 infants (with approximately 12% positive class for adverse GMA outcome, drawn from a high-risk clinical cohort) within a preregistered study design. Model performance was evaluated on a strict "lock-box" validation set, which remained untouched during any phase of model development or pre-processing optimization. The developed model achieved moderate predictive accuracy for clinician-assessed GMA scores (Area Under the Receiver Operating Characteristic Curve, ROC-AUC = 0.79; Area Under the Precision-Recall Curve, PR-AUC = 0.34). The moderate accuracy is noteworthy given the 12% positive class prevalence. By releasing de-identified feature data and open-source code, and simplifying the training pipeline using automated machine learning, our work establishes essential groundwork for future robust, globally relevant CP screening tools suitable for low-resource settings.
**Corresponding Author:**	Melanie Segado<br>University of Pennsylvania School of Engineering and Applied Science<br>Philadelphia, UNITED STATES
**Corresponding Author Secondary Information:**	
**Corresponding Author's Institution:**	University of Pennsylvania School of Engineering and Applied Science
**Corresponding Author's Secondary Institution:**	
**First Author:**	Melanie Segado, PhD
**First Author Secondary Information:**	
**Order of Authors:**	Melanie Segado, PhD<br>Laura A Prosser, PT, PhD<br>Andrea F Duncan, MD, MS<br>Michelle J Johnson, PhD<br>Konrad P Kording, PhD

Powered by Editorial Manager® and ProduXion Manager® from Aries Systems Corporation

Order of Authors Secondary Information:	
Response to Reviewers:	Dear reviewing editor,<br><br>We want to thank the reviewers for taking the time to comment on the manuscript. We have carefully reviewed each of their concerns and made major revisions to the manuscript in response. Notably, we agree with all reviewers that the novelty and value of the manuscript is in the open-source code for video analysis, feature extraction, and model training. We have therefore revised the title of the submission accordingly, and feel that “A Pre-Registered, Open Pipeline for Early Cerebral Palsy Risk Assessment from Infant Videos” better reflects the nature of the work.<br><br>In addition, we have substantially revised the manuscript to:<br>• Better situate our work within the current literature.<br>• Provide considerable new detail regarding the dataset itself, including its acquisition and processing.<br>• Offer a more thorough explanation and interpretation of the model's results.<br>• Clearly articulate the limitations of the current approach and results<br>• Further emphasize the potential for open science methodologies like ours to advance the field of cerebral palsy detection.<br>• Clarified our use of terminology (specifically as it relates to the ‘lock-box’ testing)<br>• Improved all the figures, added several new figures, and carefully checked the text for spelling, grammar, and language mistakes.<br><br>We believe these revisions have significantly strengthened the manuscript and more clearly address the points raised. Detailed responses to each specific comment can be found below. Reviewer comments are included in quotations, with responses inline.<br><br>Thank you for your time and consideration.<br><br>Sincerely,<br><br>Melanie Segado (on behalf of all co-authors)<br><br>------<br>Reviewer 1:<br><br>“The paper is targeting early CP prediction by proposing an automatic method for assessing infant risk from videos using a high-risk cohort of 1 060 infants. The method combines video-based pose estimation with predefined feature extraction and classification of movement pattern through automated GMA. By focusing on material related to my competencies within machine learning for movement analysis and CP prediction, I have the following remarks which the authors should carefully pay attention to in order for the paper to be worthy of publication:”<br><br>We appreciate the thorough feedback, and agree that the paper, if published, will be stronger because of it.<br><br>“The authors have focused on developing a simple yet automatic method for detecting fidgety movements that generalizes beyond the training and validation data by relying on a small set of 38 kinematic features derived from video-based pose estimation. This inherently restricts the level of predictive accuracy that can be achieved by the method but at the same time could make the classifier a lightweight, interpretable alternative to high-accuracy deep learning approaches for automated GMA.”<br><br>We would like to clarify that the goal of our work was specifically to avoid predicting labeled fidgety movements, an approach that we feel may lead to overfitting due to a reliance on manual annotation of specific frames/videos, and datasets trained on samples that are already known to have differing numbers of fidgets due to the nature of the GMA administration. We do not make any claims about having learned to detect fidgets but rather a movement-based prediction that aligns with clinician-determined GMA scores.<br><br>The 38 features are definitely limiting compared to precise featurizations- but that was part of the objective. It is already clear from the literature that precise featurization (like

Powered by Editorial Manager® and ProduXion Manager® from Aries Systems Corporation

deep-learned fidget detection) yields very high ROC predictions of GMA score. Our goal was different - to see if using very simple features we could achieve similar results. The reason being that these simple features will be computable from all videos of infants moving, and ideally generalize better to other datasets, including those before 3-4 months when fidgets are typically diagnostic of CP (an assumption that we hope to test in future work).

However, this goal may not have been clear as written. We've updated the paper to better reflect the objectives of our work, and the performance of the autosklearn classification relative to the work of others.

"The authors have not currently focused on the interpretability of the method, and hence major revision is necessary for this request to be addressed. The desired outcome would be that the paper illustrates how different kinematic features contribute to the prediction of present or absent fidgety movements through the use of feature importance values provided by the sklearn classification model."

We agree that including more detail about the kinematic features would improve the paper, so we've added a section describing them, and also discussing each feature was included in the clinician-driven featurization.

However, we disagree that the specific kinematic features that contribute to the prediction are important to highlight in the work, and in fact intentionally chose to avoid doing so, to avoid having others try and use this as a basis for feature selection in future work.

In reality, the auto-sklearn framework uses all of the features to make a prediction, so even if we try to interpret the top "n" features given by the feature importance plot as more important, they are not actually more important than the remaining features in aggregate. Specifically, what we mean is that we have no real basis to conclude that a small number of features, each with high importance, is more relevant than a larger number, each with smaller importance.

While this was one of the main revisions you requested, we hope that our response provides sufficient justification for why these were excluded, and that you agree with our reasoning. We have added this to the text so it is clear to future readers as well.

"The authors repeatedly make incorrect claims and bold statements without support from results or literature. This concern needs to be properly addressed (see further comments in associated sections below)."

We are sorry for our misreading of the literature which we corrected in the manuscript. We have responded to points inline for each of the sections.

"Abstract: «lock-box» set: Use standard terminology (test set or external validation set)"

Our use of lock-box to distinguish from a regular test or external validation set is intentional, and taken from the recommendations proposed by Hosseini 2020 (https://www.sciencedirect.com/science/article/pii/S0149763420305868) to prevent the risk of "overhyping" in machine learning.

Overhyping here refers to the fact that standard train/test splits are typically done after many consequential decisions in preprocessing, feature computation, and architecture selection have already been made, and are not typically pre-registered, so while none of the data points in the test set are used to train the model, the data does still risk biasing the result towards success.

Conversely, a lock-box refers to "a subset of data that are removed from the analysis pipeline at the very start of optimization and not accessed until all hyperparameter adjustments and training have been completed." It therefore allows for higher confidence in the generalizability of the processing pipeline as a whole, and not just the model.

However, since this terminology has not been widely adopted in the literature, we have

amended the manuscript to include a brief description of the distinction between a standard test/external validation approach and the lock-box approach, clarifying that a lock-box is a more stringent extension of an external validation set.

“Background: "The combined advancements in deep learning, open datasets, and open-source tools have significantly improved the reliability and accuracy of pose estimation and tracking outcomes[24, 25].": This statement should be supported by scientific references, not GitHub links”

We have revised the manuscript to include the relevant links to the literature in addition to the github links; however, in the spirit of reproducibility we feel it is both relevant and important to reference the open-source tools directly.

"Pretrained vision transformers should be sufficiently good to capture the movement features that are relevant for clinical assessment, without the need for custom models that risk overfitting.": Refrain from such statements without references or other evidence”

We based this statement off the truly exceptional performance of vision transformers across all domains of computer vision, including for pose estimation. We have added a line to the manuscript (with reference) indicating why we think this claim is generally true and added additional information detailing the performance we observed on infant data.

"This approach was highly effective at detecting FMs, however, as they noted this approach cannot be extended without the need for retraining on other hand-labeled segments.": Elaborate on this and inform the reviewer about where in the paper (reference 6) this information can be found”

This statement was incorrect as worded and has been removed from the manuscript. The authors did not note this in their manuscript, and we appreciate you pointing this out.

That said, the intent of the statement remains true. The model relies on hand-labelled segments of video with FMs. Training it to detect movement patterns other than exclusively FMs, or retraining on a different dataset would therefore require additional hand labeled segments, and since our goal was to predict risk without relying on FMs we see this as a limitation of their approach particularly as it relates to the generalizability of the method to ages prior to the development of fidgets (0-3 months).

"[…] none employed a "lock-box" set (ie. held-out data points that were not used at any point during the hyperparameter optimization process), raising the possibility that results are overly optimistic [28].": This statement is wrong, there are previous studies that have used a hold-out test set/external validation set for final evaluation (one such example is the study of Gao et al. that the authors refer to in reference 6). The authors should be overly careful when making such claims.”

We agree that it is necessary to be overly careful when making such a claim, and have added a section to the manuscript underscoring what we meant in drawing that distinction. Gao used both an internal (cohort 1) and external validation set (cohort 2), as well as an independent set for FM labelling (cohort 3). Your deep learning work has also used similar splits. and our goal is not to diminish the quality of the work that has been done in the field.

Our goal was to highlight that minimizing the risk of overfitting requires not just an external validation set, which is standard in ML and Medical testing, but a true “lock-box” that has been pre-registered. Hopefully this argument is made more clearly in the revised manuscript.

We have added a line to our manuscript further clarifying why the lock-box/pre-registration are important, and are now in contact with Dr. Lu and Dr. Yu (corresponding authors on Gao 2024) to test how well the MAM model generalizes to our dataset and vice versa. We have not been able to do so yet, but hope to do so in

Powered by Editorial Manager® and ProduXion Manager® from Aries Systems Corporation

the near future.

"To compute accurate movement features, we started by selecting an open-source pose estimation algorithm that had high precision on our infant dataset.": How did the authors verify this? There are no results in the paper backing such a claim."

A section has been added to the manuscript clarifying how we determined that the algorithm was performing sufficiently well on our dataset, and updated the figure to provide additional visual evidence. We have also removed the word "high precision" since we did not measure precision directly, and should have avoided using the term colloquially. Our goal here wasn't to make big claims about the performance of other researchers' algorithms (which we didn't test, largely because the model weights were not available), only our own, and have amended the wording accordingly.

For evaluating the datasets, we did not have an annotated ground-truth dataset, so we relied on the opinions of the clinicians involved in GMA scoring. We first tried to find a publicly available algorithm that had been fine-tuned for use on infants, but did not find any with model weights available. We then tested several publicly available algorithms, including HRNet and PVTV2 that we fine-tuned on 10K frames of infant video, but these still (visually) struggled with accurate bone-length estimates, and introduced a lot of high-frequency jitter in wrists and ankles that the clinicians felt could be diagnostically misleading.

ViTPose-H worked (visually) very well off-the-shelf (on the dataset presented in this work, and several others in our lab), and had the added benefit that other research groups would quickly be able to implement without having to fine-tune on new data. We released a set of keypoints extracted from videos from the YouTube 8M dataset using ViTPose-H on figshare (https://doi.org/10.6084/m9.figshare.25316500.v1), along with the video URLs as a pseudo "benchmark" for others to see how they perform on videos of infants generally.

Interestingly, a paper published just recently (April 9th, 2025) actually did do a systematic benchmarking of pose estimation algorithms for infant pose estimation. While ViTPose-H performed very well relative to other algorithms, their fine-tuned version of ViTPose performed even better. They have released the weights, and we have updated our manuscript to reflect this.

https://www.nature.com/articles/s41598-025-96206-0

"We show that movement features can predict GMA scores in the largest infant dataset used to date": Once again the authors make an incorrect claim (the study of Gao et al. (reference 6) used a larger sample), indicating that they do not have sufficient overview of related studies and even the references they have included."

You're right this was incorrect as Cohort 2 in Gao's study was not a subset of the sample of 1206 videos but rather an additional sample of 283. Thank you for pointing that out!

We have removed references to being the largest sample collected to date, and amended the manuscript to better reflect the sample size of our work in relation to others.

We've also clarified in the manuscript that our only goal in highlighting the sample size is to underscore the need for bigger datasets. Gao has a very large dataset - you also have a very large dataset in your work - and so does our research group. Training on more data is better for all of us.

"Patient characteristics
- Lack statistics on risk factors in the sample of recruited infants
- Inclusion/Exclusion Criteria"

Inclusion exclusion criteria are clearly stated in the manuscript under "Data

	Description” → “inclusion/exclusion criteria”.<br><br>Information regarding the risk factors has been added to “Patient Characteristics”<br><br>“The authors should include a flow diagram that more intuitively illustrates the process of inclusion/exclusion per criterion (including the number of infants excluded at each stage)”<br><br>This is stated in the text, but has been made into a flow diagram for easier visualization.<br><br>“"The remaining 931 videos were split into an analysis set (744) and a lock box holdout set (187). The analysis set was further split into train/val/test sets (558, 93, 93), each of which had a 12% representation of the "absent fidgety" movement type.": Currently, there is confusion related to the datasets in the paper, especially the use of three dataset splits, "train", "val" and "test", in addition to the "lock-box" set. What makes it even more confusing is that "train" and "val" are together referred to as "training set" (e.g., Figure 1). The reviewer proposes to stick with common ML methodology and use three dataset portions, "train" (651), "validation" (93) and "test" (187), with the last being the "lock-box" set.”<br><br>We agree that as worded this was needlessly confusing. It was written for technical correctness in relation to the pre-registered splits, but we have modified it to be more intuitive for the reader. The discrepancy arose because Auto-ML uses successive halving during model training and as such only requires a Train/Test split as opposed to the Train/Validation/Test split typically used (it implicitly uses the validation sets).<br><br>“Analysis<br>- "Infant videos pose unique challenges for pose-estimation algorithms due to frequent irregular body poses, the presence of body-like objects (e.g., toys or cartoons), and high levels of self-occlusion.": The authors should also mention the different anatomical proportions of infants compared to adults, and refer to the study of Sciortino et al. (https://link.springer.com/chapter/10.1007/978-3-319-68548-9_38).<br><br>- "Conventional algorithms, such as OpenPose[29], often fail in such conditions, leading to unreliable pose estimates [22]. Fine-tuning is often required to improve accuracy on each infant dataset.": Lack of infant-specific references, e.g., https://www.sciencedirect.com/science/article/pii/S0895611121001610.”<br><br>Thank you for bringing these references to our attention - we have included them in the revised manuscript. However, it is worth noting that modern vision transformers are not sensitive to the input body proportions of the subject for 2D pose estimation and this is therefore much less of a concern than for other algorithms. Our updated figure now shows additional details highlighting the point.<br><br>"However, we found that a pre-trained vision transformer, ViTPose-H [19], performed better than fine-tuned alternatives [30, 31, 32, 29, 33], obviating the need for manual finetuning.": How was this evaluated? There does not seem to be any results in the paper comparing the accuracy of ViTPose-H to a fine-tuned pose estimation model. Currently, there is only a single example image in Figure 2. For the authors to claim that pre-trained ViTPose-H is better than fine-tuned alternatives, this should be supported by quantitative evidence on a sufficiently large sample of infant images/videos.”<br><br>“Furthermore, the fine-tuning procedure of OpenPose is not mentioned in the text and as an outdated method OpenPose is not representative of the performance that could be achieved through fine-tuning. Hence, it is better to avoid such statements to avoid confusion and invalid conclusions.”<br><br>As clarified in the previous response, a section has been added to the manuscript describing the process that was used to select ViTPose-H as the algorithm for this work. Prior to switching to ViTPose we tested a few different pose estimation algorithms (exclusively on the training set, not the holdout) and reviewed the results

Powered by Editorial Manager® and ProduXion Manager® from Aries Systems Corporation

with clinicians. We found performance was much worse than VitPose-H, especially with regards to the stability of detected keypoints of the wrists/ankles.

Unfortunately none of the videos included in this work were consented for publication or re-distribution, and so releasing the full keypoint time series for public review/algorithm benchmarking is not possible. In lieu of this, we have released a set of key points for a (relatively) large sample of 94 youtube videos (available: https://doi.org/10.6084/m9.figshare.25316500.v1) which can be visualized and directly compared to the performance of a previously used algorithm (openpose finetuned on infants). Additionally, we have replaced the initial figure with an improved version showing examples of the types of improvements we observed with the ViTPose-H algorithm relative to the algorithm our lab was using previously (finetuned Openpose). We have selected frames illustrating 3 specific points where algorithms often fail - lost keypoints on low contrast backgrounds, bone length distortions, and inability to handle self-occlusions.

I should also clarify that we are not making any claims about the performance that could be achieved by fine tuning - fine-tuned ViTPose-H has now been shown to be better than off-the-shelf by a different research group (https://doi.org/10.1038/s41598-025-96206-0). However we specifically chose to go with a pre-trained algorithm that did not require fine tuning to improve the likelihood that performance on datasets beyond what we have collected will be similar to the performance we achieved on ours.

That said, as previously mentioned, a separate research group has actually fine-tuned ViTPose and shown that it works even better on infants in their dataset (especially for correctly estimating the width of hip keypoints), so we’ve amended the manuscript accordingly, and will be testing this on our data in future.

We have amended our wording to better reflect that our conclusion is not based on a published benchmark, nor a direct comparison to the work of others, but rather an internal benchmarking process to select an algorithm whose performance aligned with clinician judgement.

“A set of 38 kinematic features was selected based on clinician input [34, 35], designed to capture the displacement, speed, velocity, acceleration, and entropy of key body parts, specificallywrists, ankles, elbows, and knees (Figure 3).": The authors should provide a list of the specific kinematic features along with their biomechanical definitions.”

The feature descriptions are well documented in previous work, listed on the pre-registration site, and their computation is well-described in the github repository. We have also added a table explaining what the features are, and why they may be relevant for early cerebral palsy risk prediction.

“Figure 3 is way too small for information to be consumed by the user.”

We agree and have updated the figure with something that is easier to read.

“Testing on the lockbox set yielded an AUC-ROC of 0.79 (Figure 4), closely aligned with the cross-validation performance, indicating minimal overfitting.": While the AUC on the test set indicates that the model does not overfit on the training and validation data it should still be addressed that the AUC is still moderate (for example, compared to the study of Gao et al.). However, the authors could argue for the interpretability of the proposed method, but this should then be backed by results on feature importance of the selected set of biomechanical features.”

Yes, the AUC is moderate compared with previous work, and we have added a line to better reflect this comparison in the manuscript.

The interpretability of this approach is in using a simple movement-based vector, not in the specific feature weights selected by the model. While it may feel significant to think of features with higher weights as more important than others, the reality is that the model is using some combination of all features to arrive at the classification result. There is no value in stating that the top 3 features had higher weights than others when

Powered by Editorial Manager® and ProduXion Manager® from Aries Systems Corporation

the distributions of those features do not clearly distinguish between Fidgety/Absent Fidgety infants, and if anything may lead readers down an incorrect path of trying to select their own features based on our feature importance result.

The novelty of this work isn't having the best-performing model, it's having a model with rigorous, fully open methods and a framework for data processing and model training that others can easily replicate on their own data.

“Discussion
"We used an exceptionally big sample (training set: 558, overall >1000 infants)": The authors should stay objective and avoid such exaggeration”

This is an exceptionally large sample size (as are those of Gao et al 2023, and datasets used in your own work). We have modified the text to reflect the fact that others also used exceptionally large sample sizes.

On that note, we are actively seeking collaborators to aggregate data across clinical sites, either through de-identified features, or federated learning.

“We found that our algorithm performs well (AUC-ROC 0.79).": Such a statement should relate to the accuracy achieved in other studies on automated GMA, e.g., https://www.nature.com/articles/s41467-023-44141-x.”

We have included a sentence better situating our model performance within the broader context of the literature. That said, our goal was not to push state of the art performance, and we make no claims about this model performing better than others. Our goal was to determine whether classification could be achieved at all without relying on any of the standard ML techniques that boost performance at the cost of interpretability/generalizability.

“Based on our rigorous pre-registered approach with a lock-box set we can be confident that we did not do any overfitting and that it will generalize well to other datasets.": This is a bold statement, since the model will not generalize beyond the characteristics of the dataset used for training.”

Absolutely, it is never possible to know out of distribution performance of the trained model until someone else tests it on their data. What we meant was that the process would generalize to other datasets, and have made this much more explicit in the wording. That said, we are confident that none of these steps were overfit to our specific data and should generalize to other datasets.

- We didn't optimize the pose estimation method for our videos (and tested them on several other datasets). Others have since tested it and found decent results under more rigorously benchmarked conditions (see: Jahn et al. 2025).
- Our features are general movement descriptions selected years before this dataset was even collected (see: Chambers et al. 2020), as opposed to precise featurizations selected based on our dataset.
- AutoSklearn will optimize hyperparameters for new datasets similarly to how it did for ours.

“The clinician-selected movement features offer only a coarse description of movement, whereas we know from the clinical literature that the difference between infants whose movements are typically developing and those that are not is often subtle.": The authors should include references that support such a claim.”

True, we have added a reference to support this claim and a more nuanced explanation of the point we were aiming to convey. In short, we were thinking of the subtle combination of features that are reflected in fidgets, and other descriptors like “cramped synchronized” or “writhing” movements.
We agree and have addressed this by including a supporting citation and clarifying our intended meaning. Specifically, we aimed to capture the subtle interplay of characteristics present in fidgets, along with other descriptive terms such as “cramped synchronized” or “writhing” movements typically used in quantifying differences

between typical/atypical motor development in infants.

“Given that the clinician-selected movement features can predict GMA score in such a large sample, we have a strong indication that models that include even more data from a wider sample of infants, and more precise features, should perform even better.": How can you be sure about this?”

Our expectation that models incorporating a wider sample of infants and more precise movement features will perform better is based on both empirical evidence and established principles in machine learning and movement analysis. The strong predictive power of clinician-selected features in our study suggests that additional, more nuanced features could further enhance performance by capturing subtle movement patterns. Fidgets themselves are one example of how very subtle differences in wrist and ankle movements can be highly indicative of CP.

Additionally, broader and more diverse datasets typically improve model generalization by reducing biases associated with limited demographic or clinical representation. Similar trends have been observed in other areas of clinical assessment and neurological disorder detection, where increasing data diversity and feature richness leads to better predictive accuracy.

While we acknowledge that more data and refined features do not guarantee improved performance in all cases, prior research and the nature of data-driven modeling strongly support this expectation. Our goal here was really to motivate the idea that we should find ways to pool datasets across sites (including sharing standard sets of de-identified features or implementing federated learning pipelines).

We have clarified this point in the manuscript, and made it much more clear why we are making such a statement to begin with.

Potential implications

“Overall we have shown that advances in pose estimation now make it entirely realistic to get precise movements from infant videos without the need for any specialized camera setup or finetuning.": How can you be such confident without any results on the preciseness of pose estimation?”

The point is less about the specific precision of the pose estimation algorithm, but rather the broader point that achieving performance better than what used to require custom trained algorithms, manual annotation, or considerable fine-tuning can be accomplished with pre-trained models that are widely accessible.

The fact that we were able to obtain high-quality pose estimates from hand-held iphone/ipad videos without any finetuning does really demonstrate that this is entirely possible for other research groups to achieve.

We have added additional details to the manuscript regarding the performance of the algorithm, and in the meantime a separate research group has done an excellent systematic benchmark that we cite.

“Methods
"Model selection and hyperparameter optimization were carried out using the Auto-sklearn 2.0 package [41, 42], with the "vanilla Autosklearn" setting.": The authors should mention which specific machine learning models and hyperparameters that were explored using auto-sklearn.”

We agree that we gave too few details about what “vanilla settings” refers to. This has been expanded upon in the revisions in a new section that discusses AutoSklearn in more depth.

Regarding the model and hyperparameters. We specifically chose not to do this in order to avoid having readers try to pick specific models based on our findings. The hyperparameters and models will change depending on the training data, and over-

Powered by Editorial Manager® and ProduXion Manager® from Aries Systems Corporation

interpreting their significance would be a mistake. The point here was that other researchers should also train AutoML models on their datasets, not necessarily that this model is uniquely good. This has been made much more clear in the manuscript.

“References
- Figure 2 not referred to in main manuscript
- 27 and 28 seem to be duplicate references”

This has been corrected in the new manuscript.

“Typos
- Lack of brackets and parenthesis
E.g., references 6 and 25 mentioned without brackets
"(typically wearing a diaper only"
- "Lage" in Figure 1”

Thank you for catching these - we’ve fixed them in the manuscript and proof-read the revision more carefully.

Reviewer 2:

“The paper describes a pipeline to automatically classify movements based on the General Movement Assessment (GMA) in a relative large dataset (931 infants after application of exclusion criteria; including 105 infants with a positive score on GMA i.e. absence of fidgety movements). This paper contributes to the innovation of the recent years to develop a tool to assess risk of developing cerebral palsy within the first months from simple video recordings. This paper provides an interesting step forward by using a general model for body point tracking from videos and explainable (pre-registered) features for the machine learning step.”

"Overall the paper is well written, however I have comments mainly on the presentation and discussion of the results."

"In general I recommend the authors to align the paper with one of the current guideline and standard frameworks for AI in medicine (e.g. TRIPOD+AI checklist)."

Title: "with video-based tracking" does not fully represent the method applied. I would be more specific in the title."

Thank you for the comments - we agree that the title was not a good representation of the paper and have updated it to “A Pre-Registered, Open Pipeline for Early Cerebral Palsy Risk Assessment from Infant Videos.” We think this title much better reflects the fact that this paper is really describing a set of methods as opposed to a scientific innovation or new state-of-the-art model for CP prediction.

Regarding the reporting frameworks, part of the GigaScience review process required us to report methods using the DOME-ML framework. We have additionally filled the TRIPOD+AI checklist and included it with the supplementary materials.

"Abstract:
All information on the dataset should be provided in the abstract (i.e. cases excluded, distribution of positive/negative classification of GMA). Additional performance metrics of the model should be eventual provided, see comments within result section."

We agree and have updated the abstract accordingly.

"Background:
The last paragraph of the background reads as a summary of the method section, including results. I would expect objectives/research questions and hypothesis there."

We have now included an objective since that was clearly missing in the original submission. Thank you for the suggestion.

Regarding the hypothesis, the article is being submitted as a “Technical Note” as opposed to a research article. Because of this the description of methods/results in the background section is more relevant than the standard hypothesis that would normally be expected.

"Data description:

Data availability: I tried to find the calculated features on OSF but could only find the .csv of the different datasets including patient characteristics and GMA scores within the OSF Storage. Please provide the links for the feature dataset. I appreciate the preregistration of the features. "

Thank you for pointing out the ambiguity of where the features have been uploaded. We have provided a direct link to them in the manuscript, and provided the link here for your reference: https://osf.io/gztmd/files/osfstorage

Features are available averaged over the whole video “features_final_total” and in 2-second windows “features_window_total”

If you have any access issues please let us know and we’ll fix it as soon as possible.

"Analyses:

Was there any converting/preprocessing applied such as cropping, stabilization, rotation, scaling , cutting to the same length to the videos before skeletal tracking? Length and frequency of the videos could matter for some of the features, thus it should be stated how this is handled."

No additional video processing was performed prior to skeletal tracking in an effort to develop a process that is invariant to such manipulations.

A description of the video parameters (including number of frames) has been added to the manuscript. We also ensured that the number of frames did not differ between our fidgety/absent-fidgety videos. These methods and the resulting statistics have been added to the manuscript in a new section called “Video characteristics”

We chose not to standardize the length of videos, even though it could affect IQR, for the following reasons. First, an initial check confirmed that the average video duration (number of frames) did not significantly differ between our positive and negative outcome groups, mitigating concerns that video length could act as a systematic confounder at the group level. More importantly, each video was recorded by clinicians with the explicit aim of capturing a sufficient epoch of movement to reliably perform the General Movements Assessment. Thus, the duration of each recording reflects a clinically determined window deemed adequate for observation. The raw IQR within such a window directly reflects the movement variability pertinent to this clinical judgment. Normalizing by total frame count might obscure true differences, particularly given that the proportion of frames containing active, analyzable movement could vary from one clinically sufficient recording to another.

This information about the decision to not standardize lengths has also been included in the revised manuscript.

"Feature relevance:
I would suggest to mention all 38 features within the paper (within a table or supplement) for the reader's convenience and also how these features relate to the clinical observations (i.e. variability, consistency, symmetry). Within Figure 3 the abbreviation of features are within the headings, however these are very small and abbreviations are not explained within the legend. Also the x and y axis labels are missing in the figure."

We agree and have included a table in the supplementary material describing each of the features. Figure 3 has also been revised for clarity.

Powered by Editorial Manager® and ProduXion Manager® from Aries Systems Corporation

"Performance metric:
The authors should not only report the AUC, especially as this can be misleading in imbalanced dataset which is the case here (931/105). The recall of 0.34, should be mentioned in the main text and abstract and not only in the legend of Figure 4. I would also appreciate a confusion matrix of the locked-box set as this helps to interpret the results. "

This is an important point. The Precision/recall tradeoff has been integrated into the text and a sentence has been added explaining its significance in the context of imbalanced data. The confusion matrix has also been added for clarity.

We have also added a section clarifying that AutoML also optimizes resampling strategy to handle class imbalance so it is clear how this factor was considered.

"The author should also discuss what the metrics mean in the current use case and discuss on setting appropriate classification threshold for risk assessment of cerebral palsy."

We agree and have added this to the discussion. Admittedly our result is not groundbreaking in the field, we have amended the wording in the manuscript to reflect this. For real-world applications, sensitivity and specific would need to be much higher than how our model performed.

"Discussion/potential complications:
To my view, within a risk screening assessment of cerebral palsy - maybe in the future widely applied in a general population- we are willing to accept a higher "false positive" rate with infants being extra checked by a specialist and with the possible consequence that more children get extra intervention/stimulation on motor development by a physiotherapist. I think we are less willing to accept "false negatives". However, this needs to be discussed with stakeholders within the field. "

Yes, this is an important point and a more thorough discussion of the clinical interpretation of the findings has been added to the discussion of the manuscript.

"Efforts should be taken to realize data pooling from different groups working on automated GMA assessment and prevent data silos. I would encourage the authors to make data as far as possible available, as well as the trained models. Alternatively federated learning could be an option to strengthen the models for cerebral palsy risk assessment and should be explored in the future. "

This is by far the most important goal of our work, and should have been better reflected in the manuscript from the beginning. We've modified the conclusion to make this goal explicit. The difficulties in sharing datasets across sites was one of our main motivations for releasing not only the features and model, but also all the code required to replicate the results across clinical sites. None of us can share videos, or even skeletal pose estimates. What we can share are features and model weights, and we think that all of us should.

We're also very interested in working with others to set up systems for federated learning. The ability to train models on all of the thousands of videos researchers have collected around the world (securely) would be a huge step forward for the community. Thank you again.

Reviewer 3:

"Your dataset presents a significant contribution to the field of automated risk assessment for cerebral palsy, particularly given its large sample size and rigorous methodological validation. However, for the benefit of readers and potential users of the dataset, additional details regarding the data acquisition process would be valuable."

Thank you for the review – we've updated the manuscript based on your suggestions,

Powered by Editorial Manager® and ProduXion Manager® from Aries Systems Corporation

and hope that it is now more informative for others that want to build off our work.

"Recording Environment:
1. Were the videos recorded in a controlled clinical setting, or were there variations in background, lighting, and positioning?"

Videos were recorded as part of standard of care through the CHOP neo-natal follow up program. Videos were varied in background, lighting, and while they were taken from a top-down perspective there was some variation in positioning due to the fact that these were handheld cameras. We have added a section called “Video Characteristics” discussing these points.

We didn’t directly quantify the differences in background/lighting, and this is something we hope to do in future - both for data quality control when processing even larger datasets.

"2. Did all infants undergo assessment under similar conditions, or were there differences that might impact model generalizability?"

Most of the videos were collected in a hospital setting under similar conditions, and all of the infants included in this dataset were at elevated risk of cerebral palsy due to hypoxia/injury at birth. This could certainly impact generalizability. Aggregating across different datasets will be essential for developing a model that will generalize at a population level, and we have clarified this point in the revised manuscript.

"Video Quality and Resolution:
3. What was the resolution and frame rate of the recorded videos?"

All of the video information has been added to the manuscript in the new “Video Characteristics” section. details were released in the OSF data repository along with the extracted metrics. Generally, videos were recorded in landscape orientation at 1280x720 resolution and a framerate of ~30fps.

"4. Were there any preprocessing steps applied to standardize video quality across different recordings?
Standardization of Data Collection:"

No, and this is something we hope to explore in future. Video quality was assessed by 2 expert reviewers trained in GMA administration and deemed to be “usable” based on their own criteria. As such, we chose not to apply any pre-processing steps. Our goal was to determine how well algorithms work “off-the-shelf” without applying any specific pre-processing of the videos to improve the likelihood that the approach would generalize. However, optimizing the video pre-processing for feature extraction is something we are actively working on and hope to do in collaboration with other researchers.

"5. Were specific protocols followed to ensure consistency in video capture, such as standardized camera angles or infant positioning?"

Anyone capturing videos (mainly hospital staff, a minority of videos were collected by parents) was instructed to capture them from a top-down perspective with the infant centered in the frame, free of any distractions, and unoccluded. Videos were collected with hand-held cameras, so the perspective may vary slightly, but we have carried out additional analyses to verify that the proportions of the normalized infant poses are consistent with a top-down perspective.

In future we hope to integrate these preprocessing steps, since camera angle can have a huge impact on both the quality of pose estimates and also the derived features (especially measures of Left/Right symmetry).

"6. Were variations in clothing, movement freedom, or recording duration controlled or documented?"

Powered by Editorial Manager® and ProduXion Manager® from Aries Systems Corporation

The conditions for video collection are included in the manuscript under “Collection of a large clinical dataset. ”:

Infants were observed in minimal attire for unobstructed visibility of the trunk, shoulders, and extremities to facilitate the observation of natural movements (typically wearing a diaper only)

In the future we hope to leverage advances in computer vision to be able to quantify these differences automatically, for instance segmenting individual clothing items and documenting them, to ensure that these factors are not influencing model performance at scale.

"Providing these details would enhance the dataset's usability and reproducibility for future studies. Thank you for your work on this important topic."

Thank you for the detailed review. The addition of the Video characteristics section has made this a stronger, more comprehensive manuscript.

Reviewer 4:

"Summary

This paper proposes an automated method for predicting the risk of cerebral palsy in infants based on the General Movements Assessment. Videos of infants are recorded, from which 2D body keypoints are extracted using ViTPose. From the tracked keypoints, a set of features, which has been defined by clinicians, is extracted, which is then used to classify the infant as having present or absent fidgety movements.
The data set used consists of around 1000 videos, which were clinically assessed by trained GM experts. One part of the data set was defined as held out test set, and the remaining data was used to evaluate the method in a cross-validation manner.
Results showed an AUC of 0.79 for the held-out test set, with a true positive rate of around 80% at a false positive rate of around 25%. Cross-validation results were reported to lie in a similar range at an AUC of 0.73.
The code for the pipeline, including keypoint estimation, feature extraction, and classification is publicly available. For the used dataset, the computed features together with clinical scores are published, while the key point data is available on "reasonable request".
This paper tackles a very relevant topic, the automated early detection of CP, which could have a big impact, since it could enable wide-spread screening, and therefore detect more children with CP earlier and therefore lead to an earlier start of treatment and therapy.
The paper is well written and uses one of the largest datasets in automated CP detection literature. The experimental design is valid, and follows a strict process, by registering the method and a test set before running the experiments. The research community could benefit a lot from this large data set, since that is one of the things that has been lacking for a long time - a dataset on which researchers can compare their methods. Up to now, most methods have only been evaluated on private, small datasets, which made the interpretation of results difficult.

Thank you for the detailed review. We hope the code and features are useful to other researchers, and are actively seeking out collaborations to train on larger, multisite data (either through feature dataset aggregation, and/or federated learning).
However, there are several things that should be improved in the paper.
Data set
More information about the data set needs to be added:
-Was the recording process standardized? Did the recorders get specific instructions?
-How was video recorded? From the top of the infant, from side view, at an angle, ...?
-Was the camera position static (on a tripod) or was it a handheld with potential moving?
-Were the infants lying on a table/floor?"

Powered by Editorial Manager® and ProduXion Manager® from Aries Systems Corporation

	We agree - this information was lacking in the initial draft. To address this we have added a new section called “Video characteristics” that provides many details about the videos themselves, as well as additional details regarding how the videos were collected. This information is located under “Collection of a large clinical dataset”<br><br>Specifically:<br>- The recording process was standardized to the extent that videos were to be recorded from a top-down perspective, with the infant supine in minimal attire centred in the frame. The infant was supposed to be free of distractions and occlusions<br>- The camera position was not static, it was hand-held, but we have added additional analysis to show that the aspect ratio for the infant poses is roughly square indicating that the videos were not distorted (as would be expected if they were collected side on). This point is really important since both pose estimation and feature computation suffer if the perspective is not properly accounted for.<br>In case parents filmed the infants at home, was the recording setup the same as in the clinic?<br>Yes, in cases where the parents filmed the infants at home, they followed the same process as infants recorded at the Children’s Hospital of Philadelphia. While we don’t have information about where the videos were collected, all videos were recorded with hand-held devices, following the same instructions, and all videos were deemed “evaluable” by 2 expert reviewers trained in GMA administration. As such, we do not anticipate any differences between videos collected in the two locations.<br>In fact, this is one of the strengths of this dataset since we hope to pursue analysis of at-home videos in the future, towards development of a global pre-screening tool.<br><br>"Information about "two fully out-of-sample infant datasets" - same as above. Are there differences between these datasets and yours?<br>Description of recordings lacking - camera position, handheld, top, front, sid...
**Additional Information:**	
**Question**	**Response**
Are you submitting this manuscript to a special series or article collection?	No
**Experimental design and statistics**<br><br>Full details of the experimental design and statistical methods used should be given in the Methods section, as detailed in our Minimum Standards Reporting Checklist. Information essential to interpreting the data presented should be made available in the figure legends.<br><br>Have you included all the information requested in your manuscript?	Yes
**Resources**<br><br>A description of all resources used, including antibodies, cell lines, animals and software tools, with enough	Yes

information to allow them to be uniquely identified, should be included in the Methods section. Authors are strongly encouraged to cite Research Resource Identifiers (RRIDs) for antibodies, model organisms and tools, where possible.<br><br>Have you included the information requested as detailed in our Minimum Standards Reporting Checklist?	
**Availability of data and materials**<br><br>All datasets and code on which the conclusions of the paper rely must be either included in your submission or deposited in publicly available repositories (where available and ethically appropriate), referencing such data using a unique identifier in the references and in the "Availability of Data and Materials" section of your manuscript.<br><br>Have you have met the above requirement as detailed in our Minimum Standards Reporting Checklist?	No
If not, please give reasons for any omissions below.<br><br>as follow-up to "**Availability of data and materials**<br><br>All datasets and code on which the conclusions of the paper rely must be either included in your submission or deposited in publicly available repositories (where available and ethically appropriate), referencing such data using a unique identifier in the references and in the "Availability of Data and Materials" section of your manuscript.<br><br>Have you have met the above requirement as detailed in our Minimum	Original dataset and extracted keypoints cannot be made openly available at this time to comply with ethics regulations.

Standards Reporting Checklist?<br><br>"	
GigaScience has policies and guidelines in place for the use of generative AI-writing tools such as ChatGPT. If you have used such writing tools to assist with writing the manuscript this must be declared and cited in the text. Authors should not list AI-writing tools and other AI-assisted technologies as an author or co-author and should acknowledge that they are fully responsible for text generated or refined by AI-writing tools.<p><br><br>A summary of use (particularly in the introduction or among methods) needs to be included at the end of the paper, and the outputs should also be included as a supplementary file hosted in GigaDB or other open repositories. Please read our guidelines for more information. <p><br><br>By submitting to GigaScience, you are aware of the journal's AI-writing tools policy, and if you have declared use of such tools below, you have acknowledged this where appropriate in your manuscript and have made a summary of use and outputs available. <p><br><b>AI-assisted writing tools have been used in the preparation of this manuscript?	No

Manuscript

Click here to access/download;Manuscript;qbwgynhrxfsgsdvmcmfwtqfjwfmr

GigaScience, 2023, 1–12

doi: xx.xxxx/xxxx
Manuscript in Preparation
Paper

PAPER

A Pre-Registered, Open Pipeline for Early Cerebral Palsy Risk Assessment from Infant Videos

Melanie Segado, PhD[1,2,5], Laura A. Prosser, PT, PhD[4,5*], Andrea F. Duncan, MD, MS[4,6], Michelle J. Johnson, PhD[1,7,8,9] and Konrad P. Kording, PhD[1,2,10]

[1]Department of Bioengineering, University of Pennsylvania, Philadelphia, PA, United States and [2]Department of Neuroscience, University of Pennsylvania, Philadelphia, PA, United States and [3]Department of Physical Therapy, The Children's Hospital of Philadelphia, Philadelphia, PA, USA and [4]Department of Pediatrics, Perelman School of Medicine, University of Pennsylvania, Philadelphia, PA, USA and [5]Division of Rehabilitation Medicine, The Children's Hospital of Philadelphia, Philadelphia, PA, USA and [6]Division of Neonatology and Department of Pediatrics, Children's Hospital of Philadelphia and [7]Department of Physical Medicine and Rehabilitation, University of Pennsylvania, Philadelphia, PA, USA and [8]Department of Mechanical Engineering and Applied Mechanics, University of Pennsylvania, Philadelphia, PA, USA and [9]Rehabilitation Robotics Lab, Perelman School of Medicine, University of Pennsylvania, Philadelphia, PA, USA and [10]CIFAR Learning in Machines and Brains Program

*prosserl@chop.edu

Abstract

Cerebral Palsy (CP), affecting approximately 1 in 500 children due to abnormal brain development, impacts movement control. Early risk assessment via the General Movements Assessment (GMA) at 3-4 months is highly predictive for CP but relies on trained clinicians. Machine-learning-based approaches for predicting GMA score from video have shown considerable promise, but are not openly available and rely on fine-tuned pre-processing steps, hand-crafted feature sets, and experimenter-driven hyperparameter selection. This, combined with strict privacy constraints on sharing data, limits the extent to which models can be trained and tested across datasets, thus reducing clinical impact. There is therefore a need to develop approaches that will work across different datasets to enable multi-site dataset aggregation and model training. To address this gap, we developed an end-to-end pipeline that uses off-the-shelf pose estimation, general-purpose feature extraction, and automated machine learning—none of which are tuned to a specific dataset. We applied this approach to a newly generated large dataset of 1063 infants (with approximately 12% positive class for adverse GMA outcome, drawn from a high-risk clinical cohort) within a preregistered study design. Model performance was evaluated on a strict "lock-box" validation set, which remained untouched during any phase of model development or pre-processing optimization. The developed model achieved moderate predictive accuracy for clinician-assessed GMA scores (Area Under the Receiver Operating Characteristic Curve, ROC-AUC = 0.79; Area Under the Precision-Recall Curve, PR-AUC = 0.34). The moderate accuracy is noteworthy given the 12% positive class prevalence. By releasing de-identified feature data and open-source code, and simplifying the training pipeline using automated machine learning, our work establishes essential groundwork for future robust, globally relevant CP screening tools suitable for low-resource settings.

Key words: Cerebral palsy; Risk assessment; Infant development; Movement analysis; Machine learning; Computer vision; Movement disorders; Predictive modeling; Pediatrics

Compiled on: May 30, 2025.
Draft manuscript prepared by the author.

Key Points

- Introduced an open, accessible video-based pipeline to predict General Movements Assessment (GMA) scores (a key early indicator of Cerebral Palsy [CP] risk), providing a foundation for more scalable pediatric risk assessment.
- Rigorously validated this pipeline on a large infant cohort (1063 videos), employing a pre-registered design and a "lock-box" test set to ensure robust evaluation and minimize the risk of overly optimistic performance estimates.
- Demonstrated that relatively simple movement features, derived from hand-held camera recordings achieves moderate predictive accuracy for GMA scores (ROC-AUC 0.79, PR-AUC 0.34) even under these stringent validation conditions.
- Designed the pipeline to facilitate broader application and collaborative research, particularly through its use of generalizable pose estimation and by enabling the extraction and sharing of de-identified movement features for aggregated dataset creation across clinical sites.
- Released the entire pipeline as open-source (including data processing, feature computation, and AutoML components) to promote transparency, reproducibility, and empower the research community to collaboratively build and refine more impactful early CP risk assessment tools.

Background

Cerebral Palsy (CP) is the most common cause of motor impairment leading to physical disability in children, affecting an estimated 2-3 out of 1000 infants globally [1]. In the USA alone, this results in approximately 1 million people living with impaired mobility due to CP at any given time, many of whom have lifelong disability. Early detection and rehabilitation before two years of age are critical, as beginning rehabilitation within this sensitive period for neural plasticity and motor development is associated with functional outcomes [2, 3]. Atypical movement patterns that indicate a high risk of developing CP are reliably detectable through a trained physician's visual observation of movements at or before 10 weeks of age, but many infants are not evaluated by a physician until after severe overt motor impairments have developed. In practice, this means that CP is typically diagnosed between 6 and 24 months of age, which is near or beyond the end of the optimal window for intervention. There is, therefore, a need to develop automated early pre-screening tools that can detect atypical patterns of motor development before they progress to more severe impairment, allowing for more efficient use of costly medical resources, and improved outcomes, particularly in low-resource settings.

CP risk is routinely assessed by clinicians based on visual observation of movements. One such assessment is the General Movements Assessment (GMA) [4], which is predictive of CP as early as 3 months of age based on expert classification of spontaneous infant movements. It distinguishes between *typical* and *atypical* General Movements (GMs), including the identification of Fidgety Movements (FMs) at 3-4 months, which are a precursor to coordinated, volitional movement. The absence of FMs at this age is 95% predictive of CP when combined with abnormal findings on brain MRI [2]. The GMA is typically scored from video and considers characteristics of movement quality, variability, and complexity. If these relevant movement features can be reliably computed from videos, then algorithmic approaches for predicting infant risk from movement features should perform robustly.

Numerous efforts by multiple research groups are underway to automate the GMA using computer vision and machine learning [5, 6, 7, 8, 9, 10, 11, 12, 13, 14, 15, 16, 17, 18]. These groups have all shown compelling evidence that GMA assessment, and by extension CP risk, can be predicted from video. However, the potential for these approaches to scale to new, unseen datasets is currently limited. Existing models rely on hand-annotated or custom fine-tuned models, which are specific to each research group's dataset, none of which are publicly available for other research groups to use as an end-to-end pipeline.

The advent of pre-trained vision transformers has enabled better feature extraction and multi-scale information fusion. This advance improves performance on data with joint- or limb-segment occlusions, as well as complex poses, both of which are common in spontaneous infant movement and challenging for infant pose estimation algorithms [19, 20, 21, 22, 23, 18, 24, 9, 25]. The combined advancements in computer vision, availability of human movement datasets, and development of open-source tools [26, 27] have significantly improved the reliability and accuracy of pose estimation and tracking outcomes [24, 19], even in challenging conditions. This raises the exciting possibility that pre-trained vision transformers could be used for infant pose estimation without the need for custom fine-tuned models for specific datasets.

Existing video-based automated risk assessment models often perform exceptionally well on private datasets. However, they are not readily available for testing on new data, have limited generalizability, and use methods that may yield overly optimistic performance estimates. For instance, Gao et al. [6] trained a transformer model on clips of hand-labeled movements and calculated the proportion of video clips labeled as FMs in their sample. This approach was highly effective at detecting FMs, consistently agreeing with expert assessment, but requires retraining on other hand-labeled segments to detect markers beyond FMs. This is an issue in terms of generalizability because FMs are only one marker of CP observable during a limited period of development. Ihlen et al. [28] also found high levels of sensitivity and specificity, comparable to clinician GMA, but the model relied on a backward prediction of over 900 features, raising the concern that the precise featurization may overfit to the specific dataset [28, 29]. Groos et al. [9] showed very high sensitivity and specificity on multi-site data using fine-tuned pose estimation and deep-learned features, but the code to replicate the methods is not available. Additionally, while all adhered to standard ML methods (such as external validation sets), none employed a stricter *lock-box* set (i.e., held-out data points that were not used at any point during the hyperparameter optimization process, and publicly pre-registered), raising the possibility that results may be overly optimistic [30] due to iterative optimization of the analysis pipeline as a whole.

The models cited above and many others provide compelling evidence that GMA and overall CP risk can be predicted from video, but the diversity of methodological approaches and lack of publicly available code currently limit their clinical impact. Moreover, the lack of public datasets makes it difficult to compare across sites and train on multi-site datasets. Sharing videos and even keypoint time-series across clinical sites is prohibitive due to privacy and ethical constraints. In contrast, kinematic features and model weights can be shared publicly without any of these concerns and offer a simple, effective way to combine datasets and train models on more data. This is important given the low prevalence of atypical movement patterns in each dataset, especially when considering additional factors like CP subtypes and severity.

Our objective was to address these limitations by developing

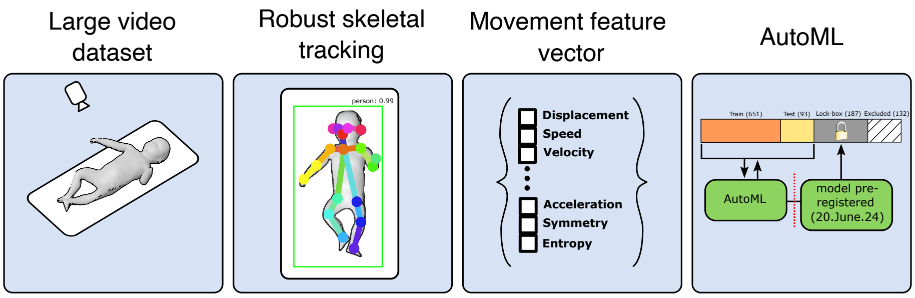

Figure 1. Process for rigorous evaluation of automated clinical score prediction. Each step of the model development process was pre-registered, including subject IDs for each training split, pose-estimation algorithm selection, movement features, and AutoML model. Pose-estimation method was pre-registered prior to feature computation. Features were pre-registered prior to model training. Model was pre-registered prior to testing on lock-box.

a pipeline, testing on a large dataset, and facilitating replication by other researchers. Accordingly, this study introduces and rigorously evaluates this open, generalizable pipeline, showcasing its performance on a large clinical dataset and its potential for enabling more accessible CP research. We assembled a large dataset of clinician-labeled videos from our institutions' United States CP Early Detection and Intervention Network site's data. To compute accurate movement features, we first selected an open-source pose estimation algorithm that performed well on our infant dataset based on clinical expert review. We then computed 38 features from the 2D pose estimates, describing posture, velocity, acceleration, left-right symmetry, and movement complexity. All 38 features were selected based on clinician-determined relevance to movement evaluation and pre-registered prior to this study. To validate the pipeline, we trained a classification algorithm using automated machine learning to predict GMA scores.

We developed a pipeline for predicting infant CP risk from video using an off-the-shelf pose estimation algorithm, simple pre-registered features, and automated machine learning that limits bias during hyperparameter optimization. We demonstrated that these movement features predict GMA scores in one of the largest infant datasets to date, and we released our feature dataset and the code needed for other researchers to process their own data, thus laying the groundwork for dataset sharing and collaborative model training (Figure 1).

Data description

Collection of a large clinical dataset

Data were collected between May 2019 and December 2023 as part of standard clinical care by team members of the CHOP site of the U.S. CP Early Detection and Intervention Network and entered into a REDCap database. This included the secure uploading of videos recorded on iPads or iPhones, GMA scores and demographic information. Access to this clinical database was restricted to hospital staff and authorized researchers. The GMA was administered in accordance with CHOP's participation in the Cerebral Palsy Foundation's Early Detection and Intervention network, which follows international diagnostic guidelines. For all infants who were between 10-20 weeks post-term age (corrected for preterm birth, if applicable) at the time of a clinic visit, and whose parents or legal guardians agreed to video recording for clinical care, clinicians captured a 1-2 minute video of the infant lying supine from a top-down perspective using handheld cameras. 10-20 weeks is the usual age for an infant's first visit with the Neonatal Follow-up Program high-risk infant follow-up clinic, and a 1- to 2- minute video was deemed by two evaluators to be sufficient for GMA administration.

Infants were observed in minimal attire for unobstructed visibility of the trunk, shoulders, and extremities to facilitate the observation of natural movements (typically wearing a diaper only). The use of pacifiers, toys, or engagement in communication with the infant during the assessment was prohibited and other distractions that could potentially influence the outcome were minimized. If patients missed their clinic visit during this time period, parents were instructed on how to capture the video and provided a link to upload the video into REDCap.

Video characteristics

The video dataset included 1063 recordings (one per infant), with a mean frame rate of 29.93 ± 3.28 FPS. There were a few exceptions including videos at 15 FPS (3 videos) and 120 FPS (7 videos). All pose-data processing was normalized to each video's frame rate.

The average number of frames per video was 3,234 ± 762 for infants with a GMA score of 1 and 3,447 ± 1,031 for those with a GMA score of 2. This corresponds to mean video durations of 112 ± 26 seconds for the GMA score 1 videos and 119 ± 36 seconds for GMA score 2 videos. While the GMA score 2 videos had a slightly higher mean (7s) and greater variability in duration (10s), a Kolmogorov–Smirnov test indicated no significant difference between the distributions ($p = 0.05$), suggesting that video length is unlikely to bias downstream comparisons of movement patterns.

Orientation was highly uniform, with 1046 videos in landscape orientation and only 14 in portrait. Video resolutions were also largely consistent, indicating a standardized acquisition protocol suitable for motion quantification. 880 videos were collected at 1280×720 resolution, and a small number had other resolutions such as 480×272, 568×320, 1280×712, and 1920×1080. All video parameters are document on the OSF pre-registration site.

Camera angle can introduce geometric distortion that affects apparent bone lengths and, by extension, the accuracy of 2D joint angle calculations. To evaluate the reliability of joint-based metrics in this dataset, we estimated the average wingspan-to-body-length ratio as a proxy for viewing angle. Across the dataset, this ratio was 0.77 ± 0.12. The ratio was calculated by dividing a 'wingspan' proxy by a 'body length' proxy. The 'wingspan' was defined as the range of x-coordinates (max x - min x) and 'body length' as the range of y-coordinates (max y - min y), both derived from a comprehensive set of body joints (including arms, shoulders, hips, and legs – excluding head keypoints) after rotating each pose to a head-up orientation and normalizing torso length to one unit. While not a perfect metric, significant deviations from expected anatomical ratios would be indicate non-top-down camera positions. The narrow variance in this measure supports the conclusion that the vast majority of recordings were obtained from a largely orthogonal top-down viewpoint, thereby minimizing projection errors and supporting valid kinematic analyses.

Table 1. Demographic and race/ethnicity characteristics across Train, Test, and Lock-box sets

(A) General Demographics

Feature	Train	Test	Lock-box	$p_{\text{T-T}}$	$p_{\text{T-H}}$
Sample Size (N)	648	93	187	—	—
% Female	54.6	55.9	55.1	0.9	0.9
Gestational Age (days)	220.4	224.4	224.6	0.2	1.0
Chronological Age (weeks)	24.6	21.9	22.2	0.2	0.7
% Absent FMs	10.8	10.8	10.2	0.9	1.0

(B) Race and Ethnicity

Category	Train	Test	Lock-box
% White	38.5	39.6	39.0
% Black or African American	33.6	32.3	32.6
% Other or Multiracial	18.7	18.3	19.3
% Not Reported (Race)	9.2	9.8	9.1
% Hispanic/Latino	8.1	7.5	7.5
% Not Reported (Ethnicity)	7.9	8.6	9.1

p-values in (A) are from chi-squared tests (categorical variables) or t-tests (continuous variables). Race and ethnicity reflect self-reported categories. Percentages may not total 100% due to rounding or non-exclusive identification.

Clinical evaluation

The evaluation process was characterized by the involvement of over 20 clinicians, including physical and occupational therapists, nurse practitioners, and physicians; several with additional advanced training. The GMA score (FMs present, absent or abnormal) was determined after adjudication by two independent clinician reviewers. In instances where disparities in assessment arose, a third evaluator was consulted. Videos with uncertain scores were reviewed in weekly meetings convened by the site's team.

This entire clinical scoring process was conducted entirely independently of the feature selection, data processing, and model development pipeline. This strict separation is essential as it ensures that our model's performance was validated against objectively derived clinical labels, thereby minimizing any potential for circular reasoning or bias that could artificially inflate its predictive accuracy.

Patient characteristics

To assess how well we could predict GMA score from clinician-selected movement features in a large sample, we used videos that were collected as part of standard clinical care. In total there were 1063 videos from the Children's Hospital of Philadelphia. The sample of 1063 infants was sex-balanced, with 55% girls, 45% boys, and 1% unknown/unspecified (Table 1 A). It also comprised a wide range of race/ethnicities, including White (38%), Black/African American (35%), 'Other' (10%). The remaining 16% of responses were spread across Multi-Racial, Asian, Indian, American Indian/Alaskan, Native Hawaiian, and Not Reported/Unknown/Other. Of reported ethnicities, 8% were Hispanic/Latino (Table 1 B).

This cohort includes a high proportion of infants with known risk factors for neurodevelopmental delay. Specifically, 70% (n=653) were born preterm (<259 days gestation), 45% (n=419) had very low birth weight (<1500g), and 28% (n=265) met criteria for extremely low birth weight (<1000g) [31].

Each of the video recordings used for analysis was determined evaluable by the clinical reviewers. In cases where infants were distracted during the recording session, a second video was obtained. Only the final videos used for GMA scoring were considered in this dataset. The mean corrected age was 14.6 weeks (+/- 2.1 weeks). Of the 931 infants that remained after applying exclusion criteria (see: Inclusion and exclusion criteria), 820 were scored as having FMs (GMA score 1) and 105 infants were scored as having absent FMs (GMA score 2). The remaining six infants were scored as having abnormal movements (GMA score 3) and were excluded from model training, but still included for pose-estimation and feature computation. The full list of excluded IDs can be found on the OSF pre-registration site (`https://osf.io/gztmd/`).

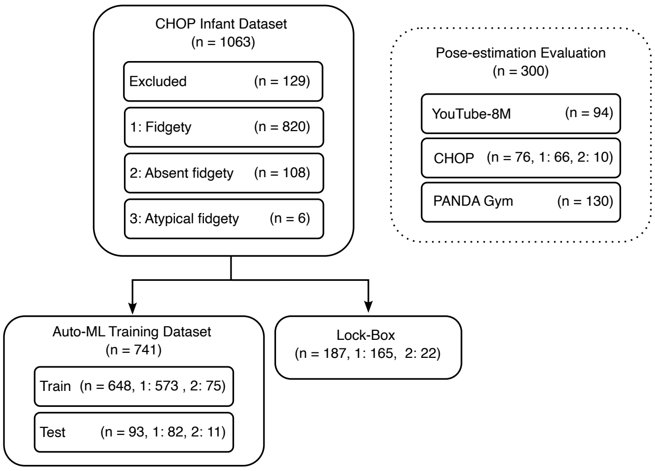

Figure 2. Datasets used for pose-estimation algorithm selection, AutoML training, and lock-box testing. The pose-estimation evaluation dataset included 94 videos from the YouTube-8M dataset, 76 videos from the training subset of the CHOP infant Dataset, and 130 additional videos of infants from ongoing projects in the lab. CHOP Infant Dataset had 1063 infants; 129 were excluded from the dataset for meeting one or more medical exclusion criteria prior to any analysis. training(648), test(93), and lock-box videos (187) each had apx. 12% representation of the positive class (absent fidgety) and were pre-registered prior to pose-tracking algorithm selection and movement feature computation. The 6 atypical fidgety videos were excluded from model training, but are included in the released feature dataset.

Inclusion and exclusion criteria

For children still hospitalized at the time of the fidgety-aged GMA, Early Detection Team members captured the videos in the hospital as part of standard care. Exclusions were applied to intubated patients, those under the influence of sedation medications, within a week post-operative, on ECMO support, or diagnosed with myelomeningocele. The full dataset comprised 1063 infants. 129 were then excluded for meeting one or more exclusion criteria listed above.

Six infants with a GMA score of 3 ("atypical fidgety") were excluded from the AutoML Training and Lock-box datasets, since there were not enough infants in this group for multi-class training and prediction (Figure 2. The remaining 928 videos were split into an analysis set (741) and a lock box holdout set (187). The analysis set was further split into train and test sets (648, 93), each of which had a apx 12% representation of the "absent fidgety" movement type.

The splits were stratified to preserve the ratios of male and female infants, as well as age, and race and ethnicity, and chronological age on upload date (Table 1 A). There was a total recording duration of 60-120 s per infant.

Analyses

Developing a pipeline for robust skeletal tracking

Performance and validation of skeletal tracking pipeline

Infant videos pose unique challenges for pose-estimation algorithms due to frequent irregular body poses, the presence of body-like objects (e.g., toys or cartoons), high levels of self-occlusion, and different body proportions relative to adults [32]. Historically, algorithms such as OpenPose [33] fail in such conditions, leading to unreliable pose estimates [22, 9, 24, 18]. Extensive fine-tuning is often required to improve accuracy on each individual infant dataset [34, 24] making it difficult for researchers without the time and technical skills to do so unable to benefit from custom models. Typically, each research group will annotate a subset of their own data to fine-tune a model that works well for their specific dataset. However, at the time we were selecting an algorithm none of the fine-tuned algorithms referenced in the literature were readily available for testing, and they either required very specific input sizes, needed re-training, or were missing key information like model weights. Increasingly this is no longer the case as open fine-tuned models for infant pose-estimation continue to be released and updated [18, 24].

One of the challenges in fast-moving fields like computer vision is the rate at which new models are developed. The open-source MMPose framework we used for the pose-estimation step of our pipeline simplifies testing multiple algorithms with different weights and adopting new ones as they are released [26]. We first tested various pre-trained algorithms on a diverse infant-video set (Figure 2). We then compared their performance to our lab's previously fine-tuned OpenPose model [5]. As we did not have a ground-truth annotated subset of videos to benchmark against, we relied on feedback from experts trained in scoring the GMA regarding whether or not the skeletal tracking was sufficiently good that a trained human would be able to administer the GMA on animated videos of the keypoint data. We found that ViTPose-H [19] performed better than the alternatives we tested (Openpose, HRNet, PVTv2) [35, 36, 37, 33, 38], obviating the need for manual fine-tuning on our test data (Figure 3).

We have made it as easy as possible for researchers to perform pose estimation on their videos by using a method and algorithm that we provide as a Docker container, built on open-source code that also provides its own Docker container. The weights for ViTPose-H are available on HuggingFace from the ViTPose-H authors. Additionally, another group fine-tuned ViTPose for infant pose estimation. They have published the model weights on Zenodo [24]. Weights can be easily specified in the pose estimation code we provided.

Generalizability testing

The robustness and generalizability of ViTPose-H were validated through iterative review of pose estimates by clinicians trained in the GMA. We relied on expert judgment to determine whether the skeletal tracking was sufficiently robust for a clinician to assess fidgety movements from the keypoints alone.

To evaluate performance on videos beyond our primary dataset, ViTPose-H was also tested on two fully out-of-sample infant datasets from a separate project in the Rehabilitation Robotics Lab ("PANDA Gym", n = 130; ages = 0-6 months, typically and atypically developing, 6 camera angles)[39], as well as on a set of 94 infant videos from the YouTube 8M dataset (total n = 130; ages 0–4 months, presumed typically developing, various camera angles) [5]. The algorithm produced consistent results across datasets (i.e., results that clinicians deemed to be sufficiently smooth), supporting its generalizability. To facilitate further testing and development, we have publicly released the ViTPose-H keypoints for the YouTube-8M videos. We propose that ViTPose-H offers a scalable and reliable solution for converting infant videos into skeletal tracking data without the need for fine-tuning, enabling broader applications in infant movement analysis.

Feature relevance and clinical interpretation

A set of 38 kinematic features was selected based on clinician input [40, 41], designed to capture the displacement, velocity, acceleration, and entropy of key body parts: wrists, ankles, elbows, and knees (Table 2).

Table 2. Summary of position and angular features

**Position Features X & Y (ankles, wrists)**	
Cross-Correlation	Bilateral coordination/symmetry.
Entropy	Movement variability and complexity.
IQR Acceleration	Captures abruptness of movement.
IQR Position	Indicates range of movement in position.
IQR Velocity	Measure of movement smoothness.
Median Position	Captures postural biases or asymmetries.
Median Velocity	Typical speed of movement, reflecting consistency and effort.
**Angular Features (knees, elbows)**	
Cross-Correlation	Bilateral coordination/symmetry.
Entropy	Movement variability and complexity.
IQR Angular Acceleration	Proxy measure of spasticity/stiffness.
IQR Angular Velocity	Measure of movement smoothness.
Mean Joint Angle	Deviations may indicate abnormal tone.
Median Angular Velocity	Measure of rigidity.
Stdev Joint Angle	Reduced variance may signal restricted movement.

GMA-trained clinicians chose these 38 movement features because they are essential components of visual GMA scoring [4]. We excluded any features directly related to GMA-specific FMs to capture general movement patterns that were not specific to the 3-4 month age window, enabling future work on earlier risk prediction and CP subtype classification.

Model performance and validation

Generalizability and robustness of feature vector for risk prediction

Our initial analysis found considerable overlap in the 38 selected features for infants with and without fidgety movements (Figure 4), suggesting that no individual feature could differentiate high-risk infants. However, the aggregated feature vector was sufficient to predict GMA scores. Using the feature vector, the model achieved an ROC-AUC of 0.72 on the validation set, and 0.79 on the holdout set. Five-fold cross-validation, repeated with six random seeds, yielded an average ROC-AUC of 0.73 ± 0.05. This demonstrates the model's robustness and internal generalizability.

We also computed the Precision-Recall Area Under the Curve (PR-AUC), a metric particularly informative for imbalanced datasets typical of clinical screening contexts. Given the positive class prevalence of approximately 12% in this holdout sample (representing infants with absent fidgety movements), the PR-AUC of 0.34 substantially exceeds the random chance baseline and highlights the model's utility in identifying at-risk infants.

The model's output was a continuous probability score indicating the likelihood of absent fidgety movements. For the purposes of classification, we selected the operating threshold on the ROC curve that maximized the difference between the True Positive Rate (TPR) and False Positive Rate (FPR). This threshold is defined as:

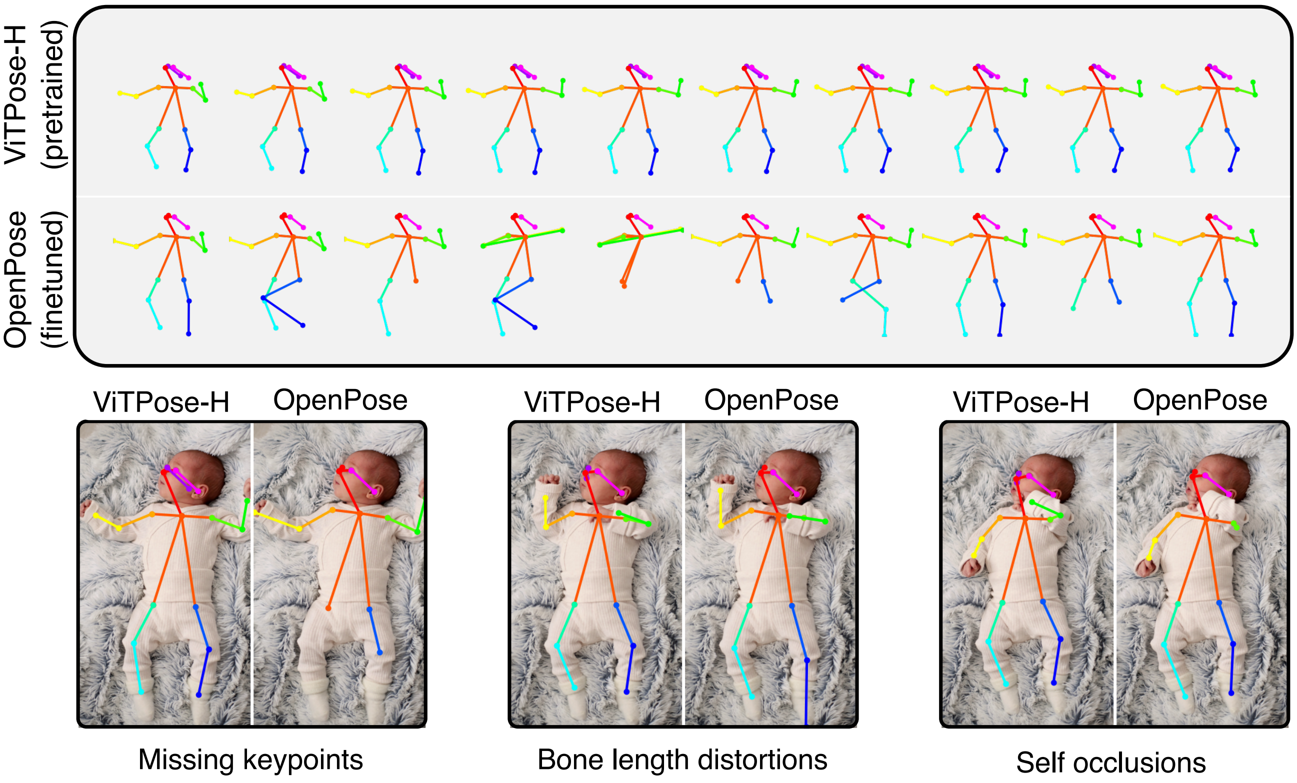

Figure 3. Improvements in skeletal tracking with pre-trained vision transformers. (Top panel) ViTPose-H (top row) produces consistent keypoint detections across frames in contrast to older algorithms like OpenPose (bottom row). (Bottom panel) Transformer-based approaches, such as ViTPose, learn adult skeletal priors and can infer missing keypoints (left), estimate bone lengths (center), and resolve self-occlusions (right) in infants.

$$\text{Threshold}_{\text{balanced}} = \arg\max_{t\in[0,1]} \{\text{TPR}(t) - \text{FPR}(t)\} \tag{1}$$

This corresponds to the threshold that maximizes Youden's J statistic (J = sensitivity + specificity − 1), ensuring balanced sensitivity and specificity in the context of class imbalance. However, alternative thresholds may be more appropriate depending on the clinical context.

A classification threshold of 0.65 on the ROC curve yields a TPR of 81.8% (18/22) and a FPR of 24.2% (40/165). Samples with a model score above this value were classified as positive (GMA score 2), and those below were classified as negative (GMA score 1) (Figure 5).

While it is possible to obtain feature importance scores from the model, we chose not to do so. Because features were chosen based on domain expertise, even if some explained more variance in our sample, this may not hold in other samples. All features contributed to the prediction; even those explaining less variance were, in aggregate, as important as the rest.

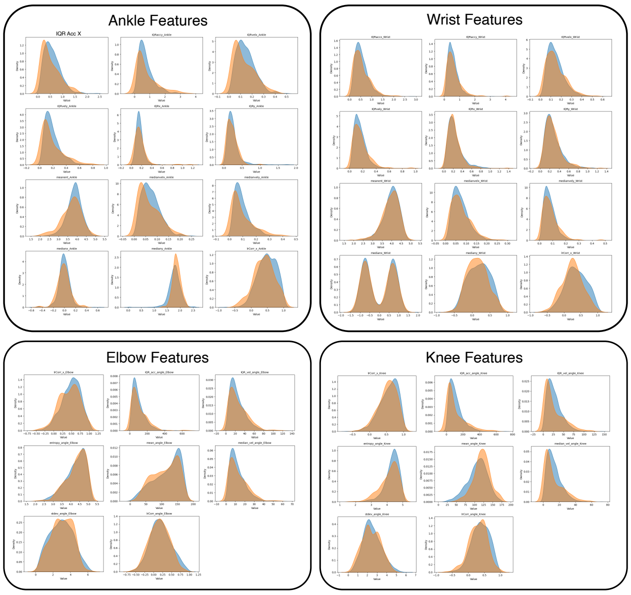

Figure 4. Individual feature distributions are highly correlated. Clinician-selected features, including XY features of the wrists/ankles and angular features of the elbows/knees, which are typically used for human assessment of risk are highly overlapping for Fidgety (blue) and Absent Fidgety (orange) movements, with no individual feature clearly predicting GMA score.

Model training with Auto-sklearn 2.0

Experimenter-driven hyperparameter optimization and model selection are significant sources of bias in machine learning, often leading to models that don't generalize well [30]. Automated Machine Learning (AutoML) frameworks like Auto-sklearn 2.0 address this by abstracting these decisions [42, 43]. Instead of manual tuning, Auto-sklearn 2.0 systematically explores algorithm choices and hyperparameter settings, leveraging meta-learning—insights from previous experiments (i.e., other, fully independent datasets with similar statistical properties) to interpret the characteristics of the input data, such as class imbalance. This allows it to strategically select appropriate handling techniques like SMOTE, class weighting, and appropriate evaluation metrics (e.g., stratified cross-validation).

Auto-sklearn 2.0 uses Bayesian optimization to search model configuration spaces and can automatically construct ensembles of top-performing models. However, under strict "vanilla" settings, training time is restricted to 1 hour and the ensemble size restricted to one [43]. This constraint ensures a highly rigorous evaluation of individual models during the search phase. This approach increases the likelihood that the selected classifiers will generalize effectively

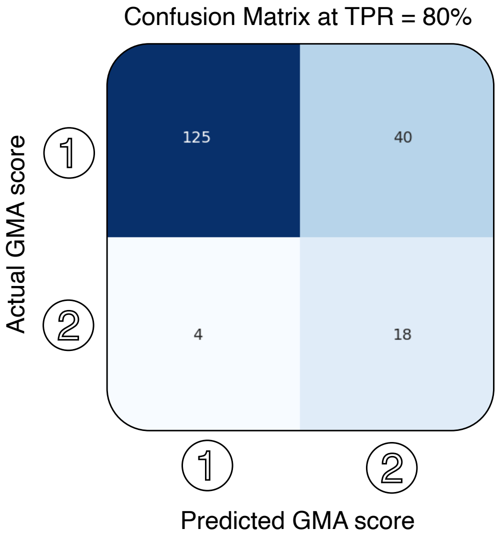

Figure 5. Confusion matrix for model predictions. A decision threshold was selected to achieve a TPR of 80% on the holdout set. GMA score 1 indicates normal fidgety movements, and GMA score 2 indicates absent fidgety movements based on expert GMA scoring. The threshold was optimized to balance sensitivity and specificity, though alternate thresholds can be based on clinical context.

to new, unseen data, ultimately leading to more robust and reliable machine learning solutions.

Rigorous methods to prevent overfitting

To ensure that the model's performance was as unbiased and generalizable as we could achieve using data from only one site, a lock-box test set of 187 infants (22 with absent fidgety movements) was randomly selected before model training and pre-processing optimization. This lock-box dataset was only accessed after pre-registering all features, pre-processing steps, and algorithms. Testing on the lock-box set yielded a ROC-AUC of 0.79, and the precision–recall curve showed an PR-UAC of 0.34 (Figure 6). The ROC-AUC was closely aligned with the cross-validation performance indicating minimal overfitting. While both measures are lower than clinician performance reported in the literature and that of many other ML models, they are notable given the simplicity of the features and the stringent training parameters.

This rigorous validation suggests that the model trained using our developed pipeline generalizes well to unseen data collected under similar conditions. It provides a reliable, reproducible approach for training models that identify infants at high risk for CP.

Discussion

Here we have developed an open, preregistered pipeline for predicting GMA score (a strong indicator of CP risk) from video-based pose estimates using rigorous methods. We used an exceptionally large sample (training set: 648, overall >1000 infants),a simple, explainable movement-based feature vector and pre-registered each step before testing on a lock-box set of 187 infant videos. We found that our algorithm trained using this pipeline performs well (ROC-AUC 0.79, PR-AUC 0.34). We utilized an AutoML approach to minimize the risk of overfitting. We further minimized the risk of overly optimistic reporting by using a lock-box set and pre-registering our analyses and models. We have made our data, code, and algorithms publicly available on the OSF pre-registration site and GitHub. While further external validation is needed, especially regarding the use of pre-trained models for pose estimation, this approach increases confidence in the potential that the pipeline will generalize across datasets, thereby facilitating efforts to share feature datasets and train models across data from multiple sites.

While the GMA has been shown to have a high level of sensitivity and specificity in clinical settings, we did not predict the main important target future outcome – diagnosis of CP – as long-term outcomes were not available at the time of model training. Instead we predicted GMA, a clinician powered risk measure. This is common throughout the automated CP risk prediction literature, with multiple research groups focusing on predicting GMA score, or detecting FMs directly, as opposed to predicting CP diagnosis. This approach is not ideal, as it introduces an additional source of noise from potential human error during assessment, in addition to the noise inherent in the GMA assessment itself. FMs, while highly indicative, are still not a perfect biomarker for CP and multiple items are necessary for CP diagnosis (biomarkers, clinical history,

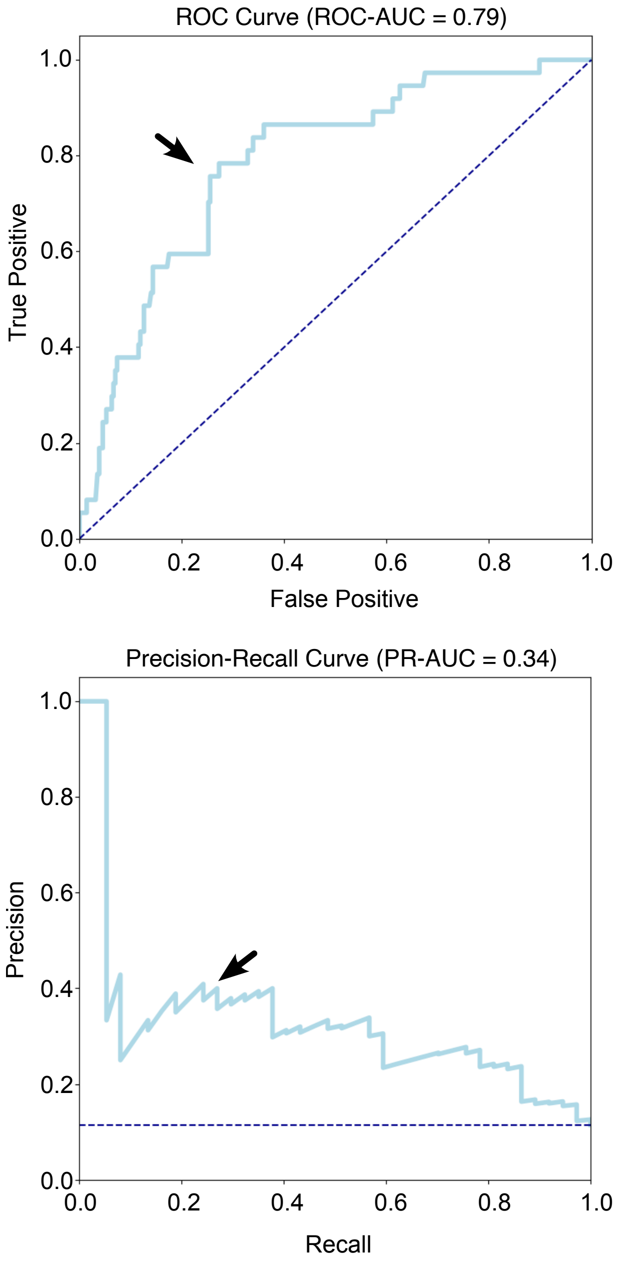

Figure 6. Model generalizes to lock-box set. Classifier trained on clinician-selected features using vanilla Auto-sklearn 2.0 shows a high ROC-AUC of 0.79 (Left) and Precision-Recall of 0.34 (Right) on lock-box set of 187 infants, having 12% representation of absent fidgety movement type. True positive rate is equal to the Sensitivity of the classifier, False positive rate is equal to 1-Specificity.

functional motor assessment, and neurological assessment). Over-reliance on FMs risks missing other, perhaps more indicative features or combinations of features that are not readily apparent, and limits risk analyses to the 3-4 month age window. Moreover, the extremely low prevalence of Abnormal FMs makes training a model that captures this movement type infeasible, meaning that some infants at high risk are often not accounted for in models trained only to detect FMs (or their absence). Future efforts should focus directly on predicting CP outcomes, subtypes, and severity.

Our current movement features are likely suboptimal for detecting subtle movement differences. The reason we say this confidently is that others have obtained much better results using approaches optimized to detect FMs. While our features are capturing some differences between groups, they are clearly missing some of the subtle movements others have captured with the direct FM featurization. The clinician-selected movement features offer only a coarse description of movement averaged over large windows, whereas we know from the clinical literature that the difference between infants whose movements are typically developing and those that are not is often subtle. For instance, infrequent, small amplitude rolls of the wrists and ankles carry significant clinical meaning, but are infrequent and may be smoothed out when averaged over an entire video. This is especially true when aiming for early prediction before the 3–4-month window. It is also a concern for general-population pre-screening, where movement differences may be even more subtle.

Many efforts have been made to identify a precise featurization using machine learning (e.g., [14, 15, 9]). However all of these efforts risk overfitting since the prevalence of absent fidgety infants relatively small (even in large datasets). The prevalence of infants who develop specific subtypes, or specific levels of severity, is even lower. Pooling data across sites—by sharing a standard set of de-identified features and adopting approaches like AutoML—would enable training on much larger datasets. Models trained on these larger datasets may be able to capture more subtle differences, boosting performance, and enabling even earlier, more precise prediction of CP.

Our current work did not investigate several contextual factors critical for the real-world deployment and performance of predictive models. Future validation must assess the pipeline's robustness to variations in video quality—such as those from less optimal recording conditions potentially encountered in under-resourced settings—and systematically evaluate the influence of diverse infant skin tones, lighting conditions, and backgrounds on pose estimation accuracy and subsequent predictions. Furthermore, the clinical heterogeneity of CP, which encompasses multiple subtypes and a broad spectrum of severity, is not fully captured by global risk scores like the GMA. A key future direction is therefore to develop models that not only quantify overall risk but also aim to differentiate CP subtypes, leveraging the hypothesis that these distinctions manifest as unique patterns within the movement feature space. Moving towards more nuanced predictions hinges on collecting larger, more diverse datasets with sufficient CP-subtype representation. Achieving this will require large-scale collaborative efforts.

The wide range of ages at which CP is typically diagnosed reflects the fact that less severe movement deficits are often not evident to untrained observers until later in an infant's development. In contrast, indicators of more severe impairment may be evident to clinicians (and caregivers) much earlier. The infants included in the model all spent time after birth in the Neonatal Intensive Care Unit (NICU), meaning that they were already at an elevated risk of CP. This limitation is prevalent throughout the automated CP detection literature [8, 10, 11, 16], since collecting videos of infants for the purposes of training a ML prediction model is most feasible in a hospital setting. As such the movements that distinguish the two groups in our sample may not be representative of infants from the general infant population. However, other people have shown that movement features can be used to predict GMA scores in at-home videos of infants that are not at high risk [14], so the approach should generalize if trained on the bigger sample that also includes infants from the general population. This should be imminently feasible now that we have released a pose estimation and pre-processing pipeline that is open, easy to share, and does not require fine-tuning.

We have shown that a simple movement-based automated prediction approach works in an very large sample. Our model's performance, with an ROC-AUC of 0.79 and a PR-AUC of 0.34, should be viewed as particularly encouraging given it was achieved under rigorous, pre-registered conditions, indicating a genuine predictive signal from relatively simple features. For any such pre-screening tool, the critical challenge lies in balancing sensitivity and specificity: a high false positive rate can unnecessarily worry parents and overburden healthcare systems with healthy children, whereas low sensitivity risks missing children who need clinical intervention, especially in low-resource settings. While our current model demonstrates that achieving a level of predictive accuracy on a large dataset is feasible even under these strict methodological constraints, it clearly requires enhanced precision for broad application. We contend that the most promising path to such improvements is through substantially increasing data scale and diversity by pooling data across many clinical sites. This becomes truly achievable when we prioritize models designed to generalize effectively across different sites and embrace the sharing of de-identified features, an approach our pipeline is built to facilitate.

Potential implications

One of the biggest limitations in infant CP research is the difficulty in sharing videos due to privacy and safety concerns. There are a growing number of research sites with datasets of over 1000 infant videos, ours is only one example of such a dataset. What's needed is dedicated effort to combine these datasets across sites. While video-based pose estimation for infants is common in the automated detection literature, each site typically uses their own custom, fine-tuned algorithm and a post-processing pipeline tailored to its dataset. This approach hinders broader collaboration and generalizability, though as noted previously, this is increasingly not the case, reflecting a broader shift towards pre-trained models that work across datasets [18, 24].

Here, we present a framework for computing and sharing de-identified features, and training models on the aggregated datasets using AutoML, thereby making it as easy as possible for other researchers to collect large video datasets, compute features, combine across datasets, and train better classifiers. All of the methods used are ethologically doable on a phone camera. All of the videos used for these analyses were collected using hand-held iPhones/iPads. The pre-trained vision-transformer we used was not fine-tuned on any of the infant videos, and was tested on a wide range of different datasets producing stable results across all of them, as assessed by clinicians. As such we expect that it will work equally well at other clinical sites and on at-home videos. Other researchers have since tested ViTPose-H on infant data and found good performance, and they have released fine-tuned weights specific to infants that they show perform even better [24] and can be specified in the pose-estimation pipeline we provide.

We have shown that advances in pose-estimation now make it entirely realistic to get movement features from infant videos without the need for any specialized camera setup or fine-tuning. We have shown that movement features derived from these pose estimates predict GMA scores in a very large sample, and that our model generalizes well to unseen data. The simplified process of obtaining de-identified features means that training on datasets across multiple sites and various contexts should now be possible. This facilitates joint efforts and holds tremendous potential for the creation of a global prescreening tool, especially if we boost per-

formance using deep-learned features (including FMs that others have shown work extremely well at 3-4 months), train on more data including from many infants across sites, and predict CP outcomes as opposed to proxy clinical scores like the GMA.

The capacity to openly share derived movement features (unlike raw videos or detailed keypoints) is crucial. While the development of sophisticated data sharing infrastructures, such as federated learning systems, is a valuable long-term goal, this process often requires considerable time and coordination. To accelerate progress in the interim, our approach leverages features that are not only easy to compute from common video recordings but have also shown predictive power. Their suitability for standardization across multiple clinical sites and developmental stages, combined with their potential for open release, offers an immediate and pragmatic pathway to foster collaboration and build richer aggregated datasets for CP research.

Methods

Developing a Pipeline for Robust Skeletal Tracking

Selecting a pose estimation algorithm

To estimate infant pose from monocular hand-held video, we implemented a top-down 2D pose estimation pipeline using tools from the open-source library OpenMMLab. MMDetection was used for infant detection [26], and MMPose was used for 2D pose estimation [26]. Infant detection was performed using an RTMDet [44] model pre-trained on the Common Objects in Context (COCO) dataset [45]. 2D frame-wise pose estimation was carried out using ViTPose-H [19], a 10B parameter vision transformer, selected for its cross-domain performance.

Processing keypoint timeseries data

Pose estimation was conducted on CHOP high-performance computing servers, ensuring compliance with ethics guidelines by restricting access to CHOP staff and authorized researchers only.

In each frame, only the highest-confidence detection was used, and frames with keypoint confidence scores below 0.8 were excluded. This process still allowed us to retain over 90% of frames. As in Chambers et al. [5], missing frames were linearly interpolated; outliers were removed with a rolling-median filter (1-second window); and data were smoothed with a rolling-mean filter (1-second window). The effect of smoothing can be seen in Figure 7.

Future work will include a systematic evaluation of our preprocessing pipeline, particularly the impact of data smoothing. The application and intensity of smoothing techniques can profoundly influence derived kinematic features, potentially obscuring or altering subtle dynamic characteristics critical for distinguishing between movement patterns. While smoothing is a common step to manage noise from pose estimation, the extent to which it affects diagnostically relevant information, such as small-amplitude variability, is not always clear. Therefore, a key future objective will be to systematically quantify these effects. Understanding how different smoothing strategies modify key kinematic outputs will be crucial for optimizing our preprocessing pipeline. This will help preserve clinically meaningful movement details and maximize the analysis's diagnostic potential.

We chose not to standardize the length of videos, even though it could affect measures of variability (notably IQR), for the following reasons. First, an initial check confirmed that the average video duration did not significantly differ between our positive and negative outcome groups, mitigating concerns that video length could act as a systematic confounder at the group level. More importantly, each video was recorded by clinicians with the explicit aim of capturing a sufficient epoch of movement to reliably perform the General Movements Assessment. Thus, the duration of each recording reflects a clinically determined window deemed adequate for observation.

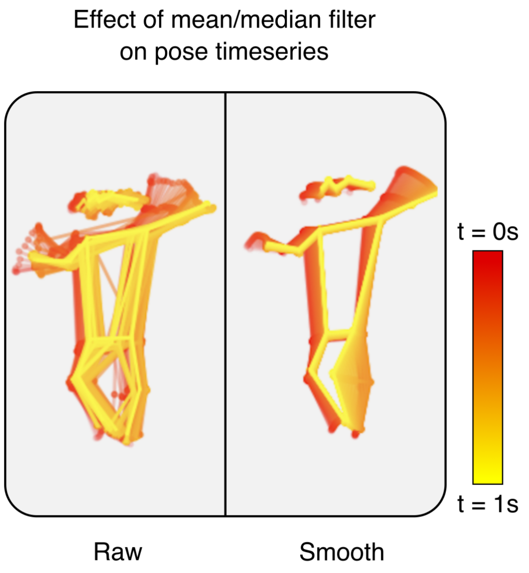

Figure 7. Raw vs smooth pose estimates over a 1s window. Raw timeseries has inter-frame jitter resulting in noisy timeseries (left). The mean and median filters (1s) reduce noise (right), but risks losing variability that may be diagnostically significant, particularly in the wrists/ankles.

The raw IQR within such a window directly reflects the movement variability pertinent to this clinical judgment. Normalizing by total frame count might obscure true differences since the proportion of frames containing active, analyzable movement could vary from one clinically sufficient recording to another.

Dataset Split and Pre-registration

Following pose estimation, infant IDs were divided into training, validation, and lock-box test sets using a stratified split to preserve a 12% representation of the absent fidgety movement type, as well as the ratio of male and female infants, and race and ethnicity as described in Table 1. The video IDs corresponding to each split were pre-registered prior to conducting further analyses, ensuring a clear separation between training, validation, and test datasets, and the lock-box test set.

Kinematic Feature Computation

After pre-registration and pre-processing, a set of 38 kinematic features were computed from the smoothed keypoint timeseries using open-source Python code [46] which was adapted from previous work [5]. These features captured displacement, velocity, acceleration, and entropy of the extremities (wrists and ankles) and joint angles (elbows and knees) (Table 2).

No specific features related to GMA FMs were included to minimize the risk of overfitting to the clinical dataset, but may be integrated into future releases.

Model Training

Feature selection and pre-registration

A binary classifier was trained to predict infants with a GMA score of "absent fidgety," which indicates a higher risk of developing CP [2, 3, 4, 47, 48]. To reduce the risk of overfitting, feature selection was conducted in consultation with clinicians prior to any data analysis and was pre-registered in 2018 [40, 41].Feature computation was automated using custom Python code available on GitHub [46].

Computed features were also pre-registered prior to model training.

We did not perform post-hoc interpretation of feature importance to avoid implying that any one feature on its own was more important for classification. The aggregate of features contributed to the prediction and any iterative feature selection may lead to overfitting.

AutoML framework for model training

Model selection and hyperparameter optimization were carried out using the Auto-sklearn 2.0 package [43, 42], with the "vanilla auto-sklearn" setting (see section: Model training with Auto-sklearn 2.0). This configuration limited the ensemble size to one, ensuring that the model with the best validation performance was selected. Balanced accuracy was chosen as the optimization metric due to the class imbalance (approximately 10:1) [49]. A meta-feature-free portfolio was used for efficient meta-learning, and training/validation splits were managed with successive halving. Cross-validation with five folds was employed to validate model generalizability across different training/validation splits, and the resulting model was pre-registered on May 22, 2024, before testing on the lockbox test set [40].

Availability of source code and requirements

The source code for skeletal tracking, feature computation, and classifier training has been made available on GitHub at https://doi.org/10.5281/zenodo.14674148. The original feature computation code, from which this work is derived, can be found at https://github.com/quietscientist/Infant_movement_assessment.

Data availability

The dataset supporting the results of this article is available on the OSF repository (https://osf.io/gztmd/) and contains participant IDs, data splits, movement features, and clinical scores [40].

In accordance with ethics guidelines and institutional review board (IRB) approvals from the University of Pennsylvania (Penn) and the Children's Hospital of Philadelphia (CHOP), study data containing identifiable information are subject to strict handling protocols. Raw videos can only be processed on-site at CHOP and cannot be shared externally. Similarly, raw pose-estimates are classified as Protected Health Information (PHI) by the Penn and CHOP IRBs and are therefore not publicly available.

Researchers interested in accessing the raw pose estimate time-series data can request to be added to the IRB protocol by contacting the corresponding author (prosserl@chop.edu). This process requires completion of mandatory training in HIPAA regulations and relevant clinical research practices. Additional screening by The University of Pennsylvania or CHOP may also be necessary.

The computed movement features from this study, as well as a dataset computing features over a 2-second sliding window to preserve temporal information, have been made available on the project's OSF Pre-registration site: osf.io/gztmd/files/osfstorage

For methodological transparency, the pose estimate data from the YouTube-8M subset (94 infants), which was used in selecting our pose estimation algorithm are also available on Figshare: doi.org/10.6084/m9.figshare.25316500.

Declarations

List of Abbreviations

ROC-AUC Receiver Operating Characteristic Area Under the Curve
PR-AUC Precision Recall Area Under the Curve
CHOP Children's Hospital of Philadelphia
COCO Common Objects in Context
CP Cerebral Palsy
ECMO Extracorporeal Membrane Oxygenation
FM Fidgety Movement
FPR False Positive Rate
GM General Movement
GMA General Movements Assessment
IQR Inter-Quartile Range
ML Machine Learning
MRI Magnetic Resonance Imaging
NICU Neonatal Intensive Care Unit
TPR True Positive Rate

Ethical Approval

Ethical approval for this study was provided by the University of Pennsylvania (Penn) Institutional Review Board (IRB Protocol Number: 833180), acting as the single IRB or record and a subsequent reliance agreement between Penn and the Children's Hospital of Philadelphia (CHOP) Institutional Review Board (IRB Protocol Number: 19-016641).

Consent for publication

The infant image used in Figure 2 to illustrate algorithm performance is a video frame taken from Adobe Stock Video (ID: #702309262), the use of which is permitted under the Adobe Stock Extended License.

Competing Interests

The author(s) declare that they have no competing interests.

Funding

This work was funded by an NIH-NICHD grant (Project#: 1R01HD097686, PIs: Johnson, Michelle J. and Kording, Konrad P.) and the clinical Early Detection Trial data collection was supported in part by the Cerebral Palsy Foundation.

Author's Contributions

Konrad P. Kording, Michelle J. Johnson, Laura Prosser, and Melanie Segado were responsible for conceptualization of the study aims. Data curation was performed by Andrea F. Duncan, Laura Prosser, and Melanie Segado. Melanie Segado conducted the formal analysis and developed the software. Funding acquisition was led by Konrad P. Kording, Michelle J. Johnson, and Laura Prosser. Data collection and clinical evaluation were carried out by Andrea F. Duncan and Laura Prosser. Methodology was established by Konrad P. Kording and Melanie Segado, with input from Laura Prosser and Michelle J. Johnson. The original draft was written by Melanie Segado and Konrad P. Kording, and all authors contributed to the review and editing of the manuscript.

Acknowledgements

The authors would like to thank Felipe Parodi for help implementing the pose estimation pipeline, and O. Francis Sowande for iterative testing on out-of-sample data. They would also like to thank Julie Skorup, , PT, DPT, PCS and Audrey J Wood, MS, PT, PCS for validation of the skeletal tracking outputs.

References

1. McIntyre S, Goldsmith S, Webb A, et al. Global prevalence of cerebral palsy: A systematic analysis. Dev Med Child Neurol 2022;64(12):1494–1506.
2. Novak I, Morgan C, Adde L, et al. Early, Accurate Diagnosis and Early Intervention in Cerebral Palsy: Advances in Diagnosis and Treatment. JAMA Pediatr 2017;171(9):897–907.
3. Herskind A, Greisen G, Nielsen JB. Early identification and intervention in cerebral palsy. Dev Med Child Neurol 2015;57(1):29–36.
4. Einspieler C, Prechtl HFR. Prechtl's assessment of general movements: A diagnostic tool for the functional assessment of the young nervous system. Ment Retard Dev Disabil Res Rev 2005;11(1):61–67.
5. Chambers C, Seethapathi N, Saluja R, et al. Computer Vision to Automatically Assess Infant Neuromotor Risk. IEEE Trans Neural Syst Rehabil Eng 2020;28(11):2431–2442.
6. Gao Q, Yao S, Tian Y, et al. Automating General Movements Assessment with quantitative deep learning to facilitate early screening of cerebral palsy. Nat Commun 2023;14(1):8294.
7. Adde L, Brown A, van den Broeck C, et al. In-Motion-App for remote General Movement Assessment: a multi-site observational study. BMJ Open 2021;11(3):e042147.
8. Hashimoto Y, Furui A, Shimatani K, et al. Automated Classification of General Movements in Infants Using a Two-stream Spatiotemporal Fusion Network. arXiv 2022;http://arxiv.org/abs/2207.03344, published online July 4, 2022. Accessed September 27, 2023.
9. Groos D, Adde L, Aubert S, et al. Development and Validation of a Deep Learning Method to Predict Cerebral Palsy From Spontaneous Movements in Infants at High Risk. JAMA Netw Open 2022;5(7):e2221325.
10. Irshad MT, Nisar MA, Gouverneur P, Rapp M, Grzegorzek M. AI Approaches towards Prechtl's Assessment of General Movements: A Systematic Literature Review. Sensors 2020;20(18):5321.
11. Kwong AKL, Doyle LW, Olsen JE, et al. Parent-recorded videos of infant spontaneous movement: Comparisons at 3–4 months and relationships with 2-year developmental outcomes in extremely preterm, extremely low birthweight and term-born infants. Paediatr Perinat Epidemiol 2022;36(5):673–682.
12. Morais R, Le V, Morgan C, et al. Robust and Interpretable General Movement Assessment Using Fidgety Movement Detection. IEEE J Biomed Health Inform 2023;p. 1–12.
13. Nguyen-Thai B, Le V, Morgan C, Badawi N, Tran T, Venkatesh S. A Spatio-temporal Attention-based Model for Infant Movement Assessment from Videos. IEEE J Biomed Health Inform 2021;25(10):3911–3920.
14. Passmore E, Kwong AL, Greenstein S, et al. Automated identification of abnormal infant movements from smart phone videos. PLOS Digit Health 2024;3(2):e0000432.
15. Redd CB, Karunanithi M, Boyd RN, Barber LA. Technology-assisted quantification of movement to predict infants at high risk of motor disability: A systematic review. Res Dev Disabil 2021;118:104071.
16. Silva N, Zhang D, Kulvicius T, et al. The future of General Movement Assessment: The role of computer vision and machine learning – A scoping review. Res Dev Disabil 2021;110:103854.
17. Spittle AJ, Olsen J, Kwong A, et al. The Baby Moves prospective cohort study protocol: using a smartphone application with the General Movements Assessment to predict neurodevelopmental outcomes at age 2 years for extremely preterm or extremely low birthweight infants. BMJ Open 2016;6(10):e013446.
18. Ostadabbas S, Fine-tuned Domain-adapted Infant Pose (FiDIP); 2023. https://github.com/ostadabbas/Infant-Pose-Estimation, published online August 17, 2023. Accessed September 6, 2023.
19. Xu Y, Zhang J, Zhang Q, Tao D. ViTPose++: Vision Transformer for Generic Body Pose Estimation. arXiv 2023;Published online December 14, 2023.
20. Liu W, Bao Q, Sun Y, Mei T. Recent Advances of Monocular 2D and 3D Human Pose Estimation: A Deep Learning Perspective. ACM Comput Surv 2022;55(4):80:1–80:41.
21. Wei K, Kording KP. Behavioral tracking gets real. Nat Neurosci 2018;21(9):1146–1147.
22. Seethapathi N, Wang S, Saluja R, Blohm G, Kording KP, Movement science needs different pose tracking algorithms; 2019. Published online July 23, 2019.
23. Hesse N, Bodensteiner C, Arens M, Hofmann UG, Weinberger R, Schroeder AS. Computer Vision for Medical Infant Motion Analysis: State of the Art and RGB-D Data Set. In: Computer Vision - ECCV 2018 Workshops Springer International Publishing; 2018. .
24. Jahn L, Flügge S, Zhang D, Poustka L, Bölte S, Wörgötter F, et al. Comparison of marker-less 2D image-based methods for infant pose estimation. Scientific Reports 2025;15(1):12148.
25. Huang X, Luan L, Hatamimajoumerd E, Wan M, Kakhaki PD, Obeid R, et al. Posture-based infant action recognition in the wild with very limited data. In: Proceedings of the IEEE/CVF Conference on Computer Vision and Pattern Recognition; 2023. p. 4912–4921.
26. Contributors M, OpenMMLab Pose Estimation Toolbox and Benchmark; 2020. https://github.com/open-mmlab/mmpose.
27. Contributors M, OpenMMLab Detection Toolbox and Benchmark; 2018. https://github.com/open-mmlab/mmdetection.
28. Ihlen EAF, Støen R, Boswell L, et al. Machine Learning of Infant Spontaneous Movements for the Early Prediction of Cerebral Palsy: A Multi-Site Cohort Study. J Clin Med 2020;9(1):5.
29. Powell M, Hosseini M, Collins J, et al, I Tried a Bunch of Things: The Dangers of Unexpected Overfitting in Classification; 2020. Published online February 14, 2020.
30. Hosseini M, Powell M, Collins J, et al. I tried a bunch of things: The dangers of unexpected overfitting in classification of brain data. Neurosci Biobehav Rev 2020;119:456–467.
31. Cutland CL, Lackritz EM, Mallett-Moore T, Bardají A, Chandrasekaran R, Lahariya C, et al. Low birth weight: Case definition & guidelines for data collection, analysis, and presentation of maternal immunization safety data. Vaccine 2017;35(48):6492–6500.
32. Sciortino G, Farinella GM, Battiato S, Leo M, Distante C. On the estimation of children's poses. In: Image Analysis and Processing-ICIAP 2017: 19th International Conference, Catania, Italy, September 11-15, 2017, Proceedings, Part II 19 Springer; 2017. p. 410–421.
33. Cao Z, Hidalgo G, Simon T, Wei SE, Sheikh Y. OpenPose: Realtime Multi-Person 2D Pose Estimation Using Part Affinity Fields. IEEE Trans Pattern Anal Mach Intell 2021;43(1):172–186.
34. Groos D, Adde L, Støen R, Ramampiaro H, Ihlen EA. Towards human-level performance on automatic pose estimation of infant spontaneous movements. Computerized Medical Imaging and Graphics 2022;95:102012.
35. Wang W, Xie E, Li X, et al. PVT v2: Improved baselines with Pyramid Vision Transformer. Comput Vis Media 2022;8(3):415–424.
36. Mathis A, Mamidanna P, Cury KM, et al. DeepLabCut: markerless pose estimation of user-defined body parts with deep learning. Nat Neurosci 2018;21(9):1281–1289.
37. Pereira TD, Tabris N, Matsliah A, et al. SLEAP: A deep learning system for multi-animal pose tracking. Nat Methods 2022;19(4):486–495.
38. Toshev A, Szegedy C. DeepPose: Human Pose Estimation via Deep Neural Networks. In: 2014 IEEE Conference on Computer Vision and Pattern Recognition; 2014. p. 1653–1660.
39. Panchal J, Sowande OF, Prosser L, Johnson MJ. Design of

pediatric robot to simulate infant biomechanics for neurodevelopmental assessment in a sensorized gym. In: 2022 9th IEEE RAS/EMBS International Conference for Biomedical Robotics and Biomechatronics (BioRob) IEEE; 2022. p. 1–7.
40. Segado M, Update: Predicting clinical assessments of infants' risk of neuromotor disease from 2-dimensional videos; 2023. 10.17605/OSF.IO/SD6FA.
41. Chambers C, Predicting clinical assessments of infants' risk of neuromotor disease from 2-dimensional videos; 2018.
42. Feurer M, Eggensperger K, Falkner S, Lindauer M, Hutter F, Auto-Sklearn 2.0: Hands-free AutoML via Meta-Learning; 2022.
43. Feurer M, Klein A, Eggensperger K, Springenberg J, Blum M, Hutter F. Efficient and Robust Automated Machine Learning. In: Advances in Neural Information Processing Systems, vol. 28 Curran Associates, Inc.; 2015. https://proceedings.neurips.cc/paper_files/paper/2015/file/11d0e6287202fced83f79975ec59a3a6-Paper.pdf.
44. Lyu C, Zhang W, Huang H, et al, RTMDet: An Empirical Study of Designing Real-Time Object Detectors; 2022. https://arxiv.org/abs/2212.07784.
45. Lin TY, Maire M, Belongie S, et al, Microsoft COCO: Common Objects in Context; 2015.
46. Segado M, Chambers MJKK, Seethapathi N, Saluja R, Prosser L, Infant Movement Assessment: Update; 2024. https://github.com/quietscientist/Infant_movement_assessment.
47. Ferrari F, Cioni G, Einspieler C, et al. Cramped Synchronized General Movements in Preterm Infants as an Early Marker for Cerebral Palsy. Arch Pediatr Adolesc Med 2002;156(5):460–467.
48. Einspieler C, Yang H, Bartl-Pokorny KD, et al. Are sporadic fidgety movements as clinically relevant as is their absence? Early Hum Dev 2015;91(4):247–252.
49. Pedregosa F, Varoquaux G, Gramfort A, et al. Scikit-learn: Machine Learning in Python. J Mach Learn Res 2011;12:2825–2830.

Click here to access/download
Supplementary Material
TRIPODAI_checklist_MSegado.pdf
