# Supplementary material for: A preregistered, open pipeline for early cerebral palsy risk assessment from infant videos
Source: Gigascience. 2026 Jan 20;15:giag003. doi: 10.1093/gigascience/giag003 (PMC13152017; doi:10.1093/gigascience/giag003)
Supplement: giag003_GIGA-D-24-00511_Revision_2 [file giag003_giga-d-24-00511_revision_2.pdf]

GigaScience

A Pre-Registered, Open Pipeline for Early Cerebral Palsy Risk Assessment from Infant Videos

--Manuscript Draft--

**Manuscript Number:**	GIGA-D-24-00511R2
**Full Title:**	A Pre-Registered, Open Pipeline for Early Cerebral Palsy Risk Assessment from Infant Videos
**Article Type:**	Technical Note
**Funding Information:**	National Institute of Child Health and Human Development (1R01HD097686) — Dr. Konrad P Kording<br>Cerebral Palsy Foundation — Dr. Andrea F Duncan
**Abstract:**	Cerebral Palsy (CP), affecting approximately 1 in 500 children due to abnormal brain development, impacts movement control. Early risk assessment via the General Movements Assessment (GMA) at 3-4 months is highly predictive for CP but relies on trained clinicians. Machine-learning-based approaches for predicting GMA score from video have shown considerable promise, but are not openly available and rely on fine-tuned pre-processing steps, hand-crafted feature sets, and experimenter-driven hyperparameter selection. This, combined with strict privacy constraints on sharing data, limits the extent to which models can be trained and tested across datasets, thus reducing clinical impact. There is therefore a need to develop approaches that will work across different datasets to enable multi-site dataset aggregation and model training. To address this gap, we developed an end-to-end pipeline that uses off-the-shelf pose estimation, general-purpose feature extraction, and automated machine learning—none of which are tuned to a specific dataset. We applied this approach to a newly generated large dataset of 1063 infants (with approximately 12% positive class for adverse GMA outcome, drawn from a high-risk clinical cohort) within a preregistered study design. Model performance was evaluated on a strict "lock-box" validation set, which remained untouched during any phase of model development or pre-processing optimization. The developed model achieved moderate predictive accuracy for clinician-assessed GMA scores (Area Under the Receiver Operating Characteristic Curve, ROC-AUC = 0.79; Area Under the Precision-Recall Curve, PR-AUC = 0.34). The moderate accuracy is noteworthy given the 12% positive class prevalence. By releasing de-identified feature data and open-source code, and simplifying the training pipeline using automated machine learning, our work establishes essential groundwork for future robust, globally relevant CP screening tools suitable for low-resource settings.
**Corresponding Author:**	Melanie Segado<br>University of Pennsylvania School of Engineering and Applied Science<br>Philadelphia, UNITED STATES
**Corresponding Author Secondary Information:**	
**Corresponding Author's Institution:**	University of Pennsylvania School of Engineering and Applied Science
**Corresponding Author's Secondary Institution:**	
**First Author:**	Melanie Segado, PhD
**First Author Secondary Information:**	
**Order of Authors:**	Melanie Segado, PhD
	Laura A Prosser, PT, PhD
	Andrea F Duncan, MD, MS
	Michelle J Johnson, PhD
	Konrad P Kording, PhD

Powered by Editorial Manager® and ProduXion Manager® from Aries Systems Corporation

Order of Authors Secondary Information:	
Response to Reviewers:	Dear reviewing editor,<br><br>Thank you for facilitating communication with Dr. Groos, who had major reservations about the manuscript. We hope that this version, that includes all of his requested analyses, addresses his concerns and the minor concerns of Dr. Hesse. Specifically we have:<br><br>1) Revised the language throughout the manuscript to avoid any misunderstandings about what we are claiming. Namely, that we are not claiming to achieve state-of-the-art results relative to deep learning methods, nor are we claiming to have produced a trained model that generalizes to other datasets. We released a comprehensive feature dataset, along with a pre-registered pipeline designed to prevent overfitting, and tested it using rigorous methods on our own data. We believe that this pipeline will generalize to other datasets, and have released everything open source in the hope that it will allow other researchers to test it and ideally release their own feature datasets to facilitate training across sites.<br><br>2) Benchmarked against the STAM model Dr. Groos requested during our email exchange. During review of the code, we identified a statistical error in the implementation which basically resulted in the model training on the test data. We have included the relevant details in our response to Dr. Groos, and in the revised manuscript.<br><br>We were not able to test our own model on a separate dataset because none of the authors we contacted, including Dr. Groos, were willing to do so. We will continue to seek out collaborators and hope to be able to publish work on out-of-sample generalizability in the future.<br><br>3) Added a section on interpretability, including permutation analyses to show the relative effects of permuting different features on the performance of this specific classifier. We have also included an additional scaling analysis to show how aggregating across larger datasets could improve model performance even with this simple feature set.<br><br>This manuscript has already sparked productive scientific debate regarding the value of different ML approaches, benchmarks, and features and we think it would be a valuable contribution to the field despite its limitations.<br><br>Sincerely,<br><br>Melanie (on behalf of all co-authors)<br><br>-----<br><br>Reviewer 1: Nikolas Hesse:<br><br>Thank you for the new round of comments. You raised many interesting points, and we have integrated them into the newest version of the manuscript.<br><br>Comment 1: "Smoothing: The visualization in Fig 7 looks like a lot of information is lost and that it's likely that any fast motions will be smoothed out. This will most probably also affect the features and predictions. You write that you will most likely improve this in the future. Will the released features then be updated accordingly?"<br><br>Our goal is to continue releasing high-quality datasets to the greatest extent permitted by our IRB. Ideally this will include expanded feature sets, and features computed using different pre-processing approaches. We are actively seeking collaborators to expand this to include data from other sites, which will be crucial for developing robust algorithms that work across various conditions.<br><br>Comment 2: "A set of 38 kinematic features was selected based on clinician input [40, 41]," For references 40 and 41 it is unclear what kind of publications these are.

	Searching for them points to the OSF site, but there seems to be no additional information to that presented in the text regarding how the features are actually computed. Some details would be helpful, e.g., median velocity - is the velocity in each frame considered one measurement, and then the median value of all measurements is taken? This would not take into account non-movement periods. To understand this better, it would be good to have all the information on how the features are computed (without having to dig into the code)."<br><br>We agree and have added a few additional lines to the relevant section of the text clarifying how, specifically, the features were computed:<br><br>"All features were computed after smoothing the dataset, with frame-wise calculations of joint angles, velocities, and accelerations, which were then aggregated either over the entire video or within overlapping 2-second sliding windows (including rest periods)."<br><br>Comment 3: "The term „explainable" features (reminds me of „explainable AI"), means to me that it is possible to explain the reasoning why a test sample was scored the way it was. I don't mean to give overall importances for the separate features in the classifier, but explanations to each specific sample. E.g., infant sequence X is predicted as having absent FMs. Is it because of variability/complexity (entropy feature)? Or because of movement smoothness (IQR velocity)? Etc…<br>If it is possible to explain a classification like this, then the GM experts can go back and verify in the videos if these features actually capture what you assume they do. If such explanation is not possible, I would not use the term „explainable" in connection to the classification."<br><br>That's a fair note on terminology and we've amended the manuscript accordingly. We have also included the feature importances for the classifier as a whole, as this was requested by another reviewer.<br><br>Our decision not to include features exclusively indicative of Fidgety Movements (FMs) relates more to our goal of developing a model with broader developmental applicability rather than directly minimizing statistical overfitting for the 3-4 month age window. FMs are powerful predictors within that specific timeframe. However, a model heavily reliant on FM-specific features would be inherently specialized to that narrow age range, limiting its utility for detecting risk at earlier or potentially later stages.<br><br>Comment 4: "You argue that the features are aimed at predicting broad developmental status. However, your clinical scores/class labels are „present FMs" vs. „absent FMs", and hence, your classifier is (supposedly) learning to predict the presence/absence of FMs. I don't think it's reasonable to assume that this approach will generalize to other age ranges/outcomes."<br><br>This is a fair argument, and we are working towards creating a labelled set of videos with fidgets that we can use for inclusion at the 3-4 month timepoint as well. The classifier is, as you said, likely already learning some proxy for fidgets since that is how this specific dataset is split. As such, the classifier itself is very unlikely to generalize beyond this age-range, but we are currently testing whether the same set of generic movement descriptors can also be used at different ages (term age) and also in other movement disorders. This would not be possible if we relied on the detection of fidgets as a core component of the pipeline.<br><br>The language in the manuscript has been clarified throughout to avoid misunderstandings.<br><br>Comment 5: "What we showed is that our model generalized to a sequestered set of data that had never been seen by the processing pipeline, so in that sense it did generalize well to unseen data."<br>If the data hadn't been "unseen" at test time, this would not be a valid evaluation. Since it is drawn from the same distribution as the training set, I would not make a bold claim on the model's generalization abilities.<br>I would consider the recordings done by parents at home to have at least some differences (even if the instructions were the same). So if the results on training with

	the data captured at the clinic and testing with parent videos is the same, this would convince me more that the model generalizes."<br><br>We completely agree. We spent several months trying to coordinate testing on separate dataset from a completely different distribution and so far have not been able to do so (sites either stopped responding, or declined to test the model on their data). What we can say for certain is that the full pipeline, from pose estimation to AutoML model training and evaluation, works end-to-end on any video dataset of people moving with any associated score that can be classified. The model itself likely won't generalize unless it's been trained across many datasets under many different conditions, which we really hope to be able to do soon, but the pipeline itself is robust and generalises well.<br><br>We've toned down the claims about generalizability so as to not overstate the result and rather emphasize the methods. Now reads: "We have shown that movement features derived from these pose estimates predict GMA scores in a very large sample, and that our model performs well on the lock-box test set"<br><br>Comment 6: "In the text, I would find it easier to read if GMA score 1/2/3 was replaced with something else, e.g. FMs present, FMs absent (, abnormal movements), or shorter FM-, FM+."<br><br>This is a great suggestion, we have revised throughout and agree it is much more readable.<br><br>Comment 7: "Not releasing the keypoints will definitely decrease the impact of the data set. I understand that this is not the author's choice, but I don't really understand how (normalized) keypoints are considered personally identifying information. Given the high barriers for getting access, I doubt that many people will actually be able to work with the keypoint data.<br>Gathering a large dataset of the (current) features doesn't seem like a promising avenue to me, due to the limitations that are also discussed in the paper/comments."<br><br>Based on the results of our scaling analysis (now included in the manuscript) aggregating even the current features across a sufficiently large population actually could yield classifiers that work at scale. This is a very low-effort means by which the community could facilitate dataset aggregation, and we hope to go adding feature computations to the pipeline as we develop them for our own data. Other sites sharing their feature computation code would certainly help accelerate this process for the community as a whole.<br><br>The sharing of keypoints is, indeed, a point of debate for IRBs. On the one hand, keypoints may not be identifiable in isolation. On the other hand, if the movement data is biometrically identifying (as we know it can be for gait), that may be a point of concern. In addition, if the dataset was collected within a specific region, it could result in situations like only one individual in the city having a specific subtype of cerebral palsy present in the dataset, thereby making it de-identifiable.<br><br>Comment 8: "Overall, I still think there are enough contributions that justify publication."<br><br>Thank you for all the suggestions and thorough review. It has certainly made the work stronger.<br><br>Reviewer 2: Daniel Groos<br>Thank you for taking the time to continue this discussion despite your reservations about the manuscript.<br>We are sorry that the revisions did not sufficiently address your concerns, and hope that the newer version includes the information you were hoping to see in order to justify publication of the work.<br><br>Comment 1: "The authors have not succeeded to address major concerns of the paper, the most critical aspects including:<br><br>- Although expressing that the objective of the paper was to compare the accuracy of a

simple machine learning method using few predefined features to more complex methods utilizing deep learning, the authors have not performed any experiments investigating this, neither by employing an existing state-of-the-art method on the proposed dataset nor by transferring the proposed method to a different dataset."

This was never the goal of the paper, and we apologize if the wording of any part of it implied as much. Our goal was to present a reproducible framework relying on 1) off-the-shelf pose estimation, 2) simple de-identified features, and 3) AutoML. We felt that there was a gap in the literature because other models often relied on precise featurizations, that were not shared, using methods that may be overfit to the datasets on which they were trained. In the revised manuscript we make it clear that we do think deep-learned features can outperform the simple generic features we're using here but without making the code and features open for others there is no way to scale. To emphasise why this matters, we've included a scaling analysis showing that training on more data, even with simple features, improves classification according to a power-law relationship.

We are happy that you are asking for comparison to benchmarks and we have some unexpected results in this domain. While we can not benchmark against public data because they do not exist, we can run the published code of STAM against our code. On paper the STAM model is better, and when trained on our data the STAM code produces a result that is well better than ours (.86 AUC). However, analyzing their code revealed a fundamental statistical mistake, they do what is called record wise crossvalidation (see Saeb et al. 2017, The need to approximate the use-case in clinical machine learning) - in other words their training set contains snippets of data from the same babies that are in the test set. Correcting for this obvious statistical mistake their AUC drops to 0.6. Our code with the same crossvalidation strategy reaches AUC=.79. This shows how our code benchmarks very favourably against published results. It also highlights how the high standard of rigor we are pioneering in our work is essential.

Comment 2:
"- The authors continue to make bold claims that the proposed pipeline generalize to other datasets without having any evidence supporting this.
- The authors insist that 'the goal of our work was specifically to avoid predicting labeled fidgety movements' and yet there appears contradictory statements in the manuscript such as 'the model's output was a continuous probability score indicating the likelihood of absent fidgety movements'."

We've clarified in the manuscript that we didn't want the pipeline to rely on fidget labels so it could be more general-purpose. We likely did end up learning some proxy for fidgets because the training sample was split on fidgety present/absent labels, but that's unavoidable given the dataset we're using. We're actively working on 1) collecting a sample of videos with fidget labels so we can include them as computable features since they are highly indicative at 3-4 months and also 2) tracking long-term outcomes to be able to train for CP probability instead of FM probability, both at 3-4 months and earlier timepoints as well.

While our method does not outperform published SOTA results for automating the GMA, it outperforms the benchmark code you suggested, and until others have made their code available for running deep-learning based methods we feel that it is still a valuable methodological contribution to the field.

Comment 3:
"- The authors were specifically informed that interpretability should be highlighted as one of the major strengths of the proposed method compared to existing deep learning-based approaches. Nonetheless, the authors neglect to address this concern, and instead continue making statements without evidence such that 'if we try to interpret the top "n" features [..], they are not actually more important than the remaining features in aggregate.'"

While we continue to have reservations regarding the value of interpretability analyses, we have now included them in the revised manuscript and apologize for not doing so in the first place. Including the analyses and results with a discussion of their limitations is

	more useful for the scientific community than omitting them entirely. We hope you agree that productive debate about the value of different approaches is good for the field, and likely to result from this work.
**Additional Information:**	
**Question**	**Response**
Are you submitting this manuscript to a special series or article collection?	No
**Experimental design and statistics**<br><br>Full details of the experimental design and statistical methods used should be given in the Methods section, as detailed in our Minimum Standards Reporting Checklist. Information essential to interpreting the data presented should be made available in the figure legends.<br><br>Have you included all the information requested in your manuscript?	Yes
**Resources**<br><br>A description of all resources used, including antibodies, cell lines, animals and software tools, with enough information to allow them to be uniquely identified, should be included in the Methods section. Authors are strongly encouraged to cite Research Resource Identifiers (RRIDs) for antibodies, model organisms and tools, where possible.<br><br>Have you included the information requested as detailed in our Minimum Standards Reporting Checklist?	Yes
**Availability of data and materials**<br><br>All datasets and code on which the conclusions of the paper rely must be either included in your submission or deposited in publicly available repositories (where available and ethically appropriate), referencing such data using a unique identifier in the references and in	No

the “Availability of Data and Materials” section of your manuscript.<br><br>Have you have met the above requirement as detailed in our Minimum Standards Reporting Checklist?	
If not, please give reasons for any omissions below.<br><br>as follow-up to "**Availability of data and materials**<br><br>All datasets and code on which the conclusions of the paper rely must be either included in your submission or deposited in publicly available repositories (where available and ethically appropriate), referencing such data using a unique identifier in the references and in the “Availability of Data and Materials” section of your manuscript.<br><br>Have you have met the above requirement as detailed in our Minimum Standards Reporting Checklist?<br><br>"	Original dataset and extracted keypoints cannot be made openly available at this time to comply with ethics regulations.
GigaScience has policies and guidelines in place for the use of generative AI-writing tools such as ChatGPT. If you have used such writing tools to assist with writing the manuscript this must be declared and cited in the text. Authors should not list AI-writing tools and other AI-assisted technologies as an author or co-author and should acknowledge that they are fully responsible for text generated or refined by AI-writing tools.<p><br><br>A summary of use (particularly in the introduction or among methods) needs to be included at the end of the paper, and the outputs should also be included as a supplementary file hosted in GigaDB or other open repositories. Please read our guidelines for more information. <p>	No

By submitting to GigaScience, you are aware of the journal's AI-writing tools policy, and if you have declared use of such tools below, you have acknowledged this where appropriate in your manuscript and have made a summary of use and outputs available. <p> <b>AI-assisted writing tools have been used in the preparation of this manuscript?	

Powered by Editorial Manager® and ProduXion Manager® from Aries Systems Corporation

Manuscript

Click here to access/download;Manuscript;GigaScience_Journal_Manuscrip

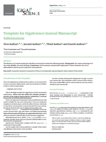

GigaScience, 2023, 1–13

doi: xx.xxxx/xxxx
Manuscript in Preparation
Paper

PAPER

A Pre-Registered, Open Pipeline for Early Cerebral Palsy Risk Assessment from Infant Videos

Melanie Segado, PhD[1], Laura A. Prosser, PT, PhD[3,4*], Andrea F. Duncan, MD, MS[3,5], Michelle J. Johnson, PhD[1,6,7,8] and Konrad P. Kording, PhD[1,2,9]

[1]Department of Bioengineering, University of Pennsylvania, Philadelphia, PA, United States and [2]Department of Neuroscience, University of Pennsylvania, Philadelphia, PA, United States and [3]Department of Pediatrics, Perelman School of Medicine, University of Pennsylvania, Philadelphia, PA, USA and [4]Division of Rehabilitation Medicine, The Children's Hospital of Philadelphia, Philadelphia, PA, USA and [5]Division of Neonatology and Department of Pediatrics, Children's Hospital of Philadelphia and [6]Department of Physical Medicine and Rehabilitation, University of Pennsylvania, Philadelphia, PA, USA and [7]Department of Mechanical Engineering and Applied Mechanics, University of Pennsylvania, Philadelphia, PA, USA and [8]Rehabilitation Robotics Lab, Perelman School of Medicine, University of Pennsylvania, Philadelphia, PA, USA and [9]CIFAR Learning in Machines and Brains Program

*prosserl@chop.edu

Abstract

Cerebral Palsy (CP), affecting approximately 1 in 500 children due to abnormal brain development, impacts movement control. Early risk assessment via the General Movements Assessment (GMA) at 3-4 months is highly predictive for CP but relies on trained clinicians. Machine-learning-based approaches for predicting GMA score from video have shown considerable promise, but are not openly available and rely on fine-tuned pre-processing steps, hand-crafted feature sets, and experimenter-driven hyperparameter selection. This, combined with strict privacy constraints on sharing data, limits the extent to which models can be trained and tested across datasets, thus reducing clinical impact. There is therefore a need to develop approaches that will work across different datasets to enable multi-site dataset aggregation and model training. To address this gap, we developed an end-to-end pipeline that uses off-the-shelf pose estimation, general-purpose feature extraction, and automated machine learning—none of which are tuned to a specific dataset. We applied this approach to a newly generated large dataset of 1063 infants (with approximately 12% positive class for adverse GMA outcome, drawn from a high-risk clinical cohort) within a preregistered study design. Model performance was evaluated on a strict "lock-box" validation set, which remained untouched during any phase of model development or pre-processing optimization. The developed model achieved moderate predictive accuracy for clinician-assessed GMA scores (Area Under the Receiver Operating Characteristic Curve, ROC-AUC = 0.79; Area Under the Precision-Recall Curve, PR-AUC = 0.34). The moderate accuracy is noteworthy given the 12% positive class prevalence. By releasing de-identified feature data and open-source code, and simplifying the training pipeline using automated machine learning, our work establishes essential groundwork for future robust, globally relevant CP screening tools suitable for low-resource settings.

Key words: Cerebral palsy; Risk assessment; Infant development; Movement analysis; Machine learning; Computer vision; Movement disorders; Predictive modeling; Pediatrics

Compiled on: October 6, 2025.
Draft manuscript prepared by the author.

Key Points

- Introduced an open, accessible video-based pipeline, and used it to predict General Movements Assessment (GMA) scores (a key early indicator of Cerebral Palsy [CP] risk).
- Rigorously validated this pipeline on a large infant cohort (1063 videos), employing a pre-registered design and a "lock-box" test set to ensure robust evaluation and minimize the risk of overly optimistic performance estimates.
- Demonstrated that relatively simple movement features, derived from hand-held camera recordings achieves moderate predictive accuracy for GMA scores (ROC-AUC 0.79, PR-AUC 0.34) even under these stringent validation conditions.
- Designed the pipeline to facilitate broader application and collaborative research, particularly through its use of generalizable pose estimation and by enabling the extraction and sharing of de-identified movement features for aggregated dataset creation across clinical sites.
- Released the entire pipeline as open-source (including data processing, feature computation, and AutoML components) to promote transparency and reproducibility.

Background

Cerebral Palsy (CP) is the most common cause of motor impairment leading to physical disability in children, affecting an estimated 2-3 out of 1000 infants globally [1]. In the USA alone, this results in approximately 1 million people living with impaired mobility due to CP at any given time, many of whom have lifelong disability. Early detection and rehabilitation before two years of age are critical, as beginning rehabilitation within this sensitive period for neural plasticity and motor development is associated with functional outcomes [2, 3]. Atypical movement patterns that indicate a high risk of developing CP are reliably detectable through a trained physician's visual observation of movements at or before 10 weeks of age, but many infants are not evaluated by a physician until after severe overt motor impairments have developed. In practice, this means that CP is typically diagnosed between 6 and 24 months of age, which is near or beyond the end of the optimal window for intervention. There is, therefore, a need to develop automated early pre-screening tools that can detect atypical patterns of motor development before they progress to more severe impairment, allowing for more efficient use of costly medical resources, and improved outcomes, particularly in low-resource settings.

CP risk is routinely assessed by clinicians based on visual observation of movements. One such assessment is the General Movements Assessment (GMA) [4], which is predictive of CP as early as 3 months of age based on expert classification of spontaneous infant movements. It distinguishes between *typical* and *atypical* General Movements (GMs), including the identification of Fidgety Movements (FMs) at 3-4 months, which are a precursor to coordinated, volitional movement. The absence of FMs at this age is 95% predictive of CP when combined with abnormal findings on brain MRI [2]. The GMA is typically scored from video and considers characteristics of movement quality, variability, and complexity. If these relevant movement features can be reliably computed from videos, then algorithmic approaches for predicting infant risk from movement features should perform robustly.

Numerous efforts by multiple research groups are underway to automate the GMA using computer vision and machine learning [5, 6, 7, 8, 9, 10, 11, 12, 13, 14, 15, 16, 17, 18]. These groups have all shown compelling evidence that GMA assessment, and by extension CP risk, can be predicted from video. However, the potential for these approaches to scale to new, unseen datasets is currently limited. Existing models rely on hand-annotated or custom fine-tuned models, which are specific to each research group's dataset, none of which are publicly available for other research groups to use as an end-to-end pipeline.

The advent of pre-trained vision transformers has enabled better feature extraction and multi-scale information fusion. This advance improves performance on data with joint- or limb-segment occlusions, as well as complex poses, both of which are common in spontaneous infant movement and challenging for infant pose estimation algorithms [19, 20, 21, 22, 23, 18, 24, 9, 25]. The combined advancements in computer vision, availability of human movement datasets, and development of open-source tools [26, 27] have significantly improved the reliability and accuracy of pose estimation and tracking outcomes [24, 19], even in challenging conditions. This raises the exciting possibility that pre-trained vision transformers could be used for infant pose estimation without the need for custom fine-tuned models for specific datasets.

Existing video-based automated risk assessment models often perform exceptionally well on private datasets. However, they are not readily available for testing on new data, have limited generalizability, and use methods that may yield overly optimistic performance estimates. For instance, Gao et al. [6] trained a transformer model on clips of hand-labeled movements and calculated the proportion of video clips labeled as FMs in their sample. This approach was highly effective at detecting FMs, consistently agreeing with expert assessment, but requires retraining on other hand-labeled segments to detect markers beyond FMs. This is an issue in terms of generalizability because FMs are only one marker of CP observable during a limited period of development. Ihlen et al. [28] also found high levels of sensitivity and specificity, comparable to clinician GMA, but the model relied on a backward prediction of over 900 features, raising the concern that the precise featurization may overfit to the specific dataset [28, 29]. Groos et al. [9] showed very high sensitivity and specificity on multi-site data using fine-tuned pose estimation and deep-learned features, but the code to replicate the methods is not available. Additionally, while all adhered to standard ML methods (such as external validation sets), none employed a stricter *lock-box* set (i.e., held-out data points that were not used at any point during the hyperparameter optimization process, and publicly pre-registered), raising the possibility that results may be overly optimistic [30] due to iterative optimization of the analysis pipeline as a whole.

The models cited above and many others provide compelling evidence that GMA and overall CP risk can be predicted from video, but the diversity of methodological approaches and lack of publicly available code currently limit their clinical impact. Moreover, the lack of public datasets makes it difficult to compare across sites and train on multi-site datasets. Sharing videos and even keypoint time-series across clinical sites is prohibitive due to privacy and ethical constraints. In contrast, kinematic features and model weights can be shared publicly without any of these concerns and offer a simple, effective way to combine datasets and train models on more data. This is important given the low prevalence of atypical movement patterns in each dataset, especially when considering additional factors like CP subtypes and severity.

Our objective was to address these limitations by developing a pipeline, testing on a large dataset, and facilitating replication by other researchers. Accordingly, this study introduces and rig-

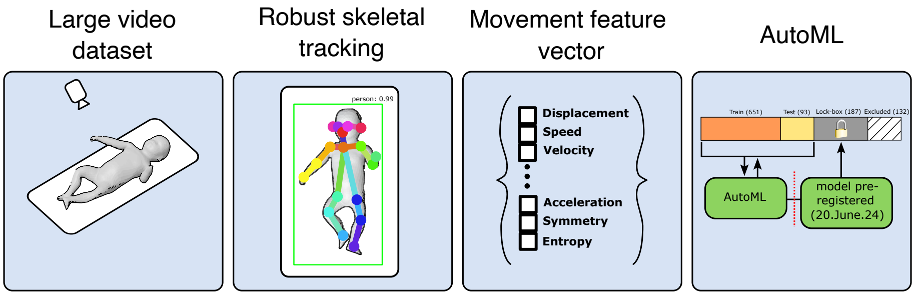

Figure 1. Process for rigorous evaluation of automated clinical score prediction. Each step of the model development process was pre-registered, including subject IDs for each training split, pose-estimation algorithm selection, movement features, and AutoML model. Pose-estimation method was pre-registered prior to feature computation. Features were pre-registered prior to model training. Model was pre-registered prior to testing on lock-box.

orously evaluates this open, generalizable pipeline, showcasing its performance on a large clinical dataset and its potential for enabling more accessible CP research. We assembled a large dataset of clinician-labeled videos from our institutions' United States CP Early Detection and Intervention Network site's data. To compute accurate movement features, we first selected an open-source pose estimation algorithm that performed well on our infant dataset based on clinical expert review. We then computed 38 features from the 2D pose estimates, describing posture, velocity, acceleration, left-right symmetry, and movement complexity. All 38 features were selected based on clinician-determined relevance to movement evaluation and pre-registered prior to this study. To validate the pipeline, we trained a classification algorithm using automated machine learning to predict GMA scores.

We developed a pipeline for predicting infant CP risk from video using an off-the-shelf pose estimation algorithm, simple pre-registered features, and automated machine learning that limits bias during hyperparameter optimization. We demonstrated that these movement features predict GMA scores in one of the largest infant datasets to date, and we released our feature dataset and the code needed for other researchers to process their own data, thus laying the groundwork for dataset sharing and collaborative model training (Figure 1).

Data description

Collection of a large clinical dataset

Data were collected between May 2019 and December 2023 as part of standard clinical care by team members of the CHOP site of the U.S. CP Early Detection and Intervention Network and entered into a REDCap database. This included the secure uploading of videos recorded on iPads or iPhones, GMA scores and demographic information. Access to this clinical database was restricted to hospital staff and authorized researchers. The GMA was administered in accordance with CHOP's participation in the Cerebral Palsy Foundation's Early Detection and Intervention network, which follows international diagnostic guidelines. For all infants who were between 10-20 weeks post-term age (corrected for preterm birth, if applicable) at the time of a clinic visit, and whose parents or legal guardians agreed to video recording for clinical care, clinicians captured a 1-2 minute video of the infant lying supine from a top-down perspective using handheld cameras. 10-20 weeks is the usual age for an infant's first visit with the Neonatal Follow-up Program high-risk infant follow-up clinic, and a 1- to 2- minute video was deemed by two evaluators to be sufficient for GMA administration.

Infants were observed in minimal attire for unobstructed visibility of the trunk, shoulders, and extremities to facilitate the observation of natural movements (typically wearing a diaper only). The use of pacifiers, toys, or engagement in communication with the infant during the assessment was prohibited and other distractions that could potentially influence the outcome were minimized. If patients missed their clinic visit during this time period, parents were instructed on how to capture the video and provided a link to upload the video into REDCap.

Video characteristics

The video dataset included 1063 recordings (one per infant), with a mean frame rate of 29.93 ± 3.28 FPS. There were a few exceptions including videos at 15 FPS (3 videos) and 120 FPS (7 videos). All pose-data processing was normalized to each video's frame rate.

The average number of frames per video was 3,234 ± 762 for FM+ infants and 3,447 ± 1,031 for FM-. This corresponds to mean video durations of 112 ± 26 seconds for the FM+ videos and 119 ± 36 seconds for FM- videos. While the FM- videos had a slightly higher mean (7s) and greater variability in duration (10s), a Kolmogorov–Smirnov test indicated no significant difference between the distributions ($p = 0.05$), suggesting that video length is unlikely to bias downstream comparisons of movement patterns.

Orientation was highly uniform, with 1046 videos in landscape orientation and only 14 in portrait. Video resolutions were also largely consistent, indicating a standardized acquisition protocol suitable for motion quantification. 880 videos were collected at 1280×720 resolution, and a small number had other resolutions such as 480×272, 568×320, 1280×712, and 1920×1080. All video parameters are document on the OSF pre-registration site.

Camera angle can introduce geometric distortion that affects apparent bone lengths and, by extension, the accuracy of 2D joint angle calculations. To evaluate the reliability of joint-based metrics in this dataset, we estimated the average wingspan-to-body-length ratio as a proxy for viewing angle. Across the dataset, this ratio was 0.77 ± 0.12. The ratio was calculated by dividing a 'wingspan' proxy by a 'body length' proxy. The 'wingspan' was defined as the range of x-coordinates (max x - min x) and 'body length' as the range of y-coordinates (max y - min y), both derived from a comprehensive set of body joints (including arms, shoulders, hips, and legs – excluding head keypoints) after rotating each pose to a head-up orientation and normalizing torso length to one unit. While not a perfect metric, significant deviations from expected anatomical ratios would be indicate non-top-down camera positions. The narrow variance in this measure supports the conclusion that the vast majority of recordings were obtained from a largely orthogonal top-down viewpoint, thereby minimizing projection errors and supporting valid kinematic analyses.

Clinical evaluation

The evaluation process was characterized by the involvement of over 20 clinicians, including physical and occupational therapists, nurse practitioners, and physicians; several with additional advanced training. The GMA score (FMs present [FM+], absent [(FM-)] or

Table 1. Demographic and race/ethnicity characteristics across Train, Test, and Lock-box sets

(A) General Demographics

Feature	Train	Test	Lock-box	$p_{T\text{-}T}$	$p_{T\text{-}H}$
Sample Size (N)	648	93	187	—	—
% Female	54.6	55.9	55.1	0.9	0.9
Gestational Age (days)	220.4	224.4	224.6	0.2	1.0
Chronological Age (weeks)	24.6	21.9	22.2	0.2	0.7
% FM-	10.8	10.8	10.2	0.9	1.0

(B) Race and Ethnicity

Category	Train	Test	Lock-box
% White	38.5	39.6	39.0
% Black or African American	33.6	32.3	32.6
% Other or Multiracial	18.7	18.3	19.3
% Not Reported (Race)	9.2	9.8	9.1
% Hispanic/Latino	8.1	7.5	7.5
% Not Reported (Ethnicity)	7.9	8.6	9.1

p-values in (A) are from chi-squared tests (categorical variables) or t-tests (continuous variables). Race and ethnicity reflect self-reported categories. Percentages may not total 100% due to rounding or non-exclusive identification.

abnormal) was determined after adjudication by two independent clinician reviewers. In instances where disparities in assessment arose, a third evaluator was consulted. Videos with uncertain scores were reviewed in weekly meetings convened by the site's team.

This entire clinical scoring process was conducted entirely independently of the feature selection, data processing, and model development pipeline. This strict separation is essential as it ensures that our model's performance was validated against objectively derived clinical labels, thereby minimizing any potential for circular reasoning or bias that could artificially inflate its predictive accuracy.

Patient characteristics

To assess how well we could predict GMA score from clinician-selected movement features in a large sample, we used videos that were collected as part of standard clinical care. In total there were 1063 videos from the Children's Hospital of Philadelphia. The sample of 1063 infants was sex-balanced, with 55% girls, 45% boys, and 1% unknown/unspecified (Table 1 A). It also comprised a wide range of race/ethnicities, including White (38%), Black/African American (35%), 'Other' (10%). The remaining 16% of responses were spread across Multi-Racial, Asian, Indian, American Indian/Alaskan, Native Hawaiian, and Not Reported/Unknown/Other. Of reported ethnicities, 8% were Hispanic/Latino (Table 1 B).

This cohort includes a high proportion of infants with known risk factors for neurodevelopmental delay. Specifically, 70% (n=653) were born preterm (<259 days gestation), 45% (n=419) had very low birth weight (<1500g), and 28% (n=265) met criteria for extremely low birth weight (<1000g) [31].

Each of the video recordings used for analysis was determined evaluable by the clinical reviewers. In cases where infants were distracted during the recording session, a second video was obtained. Only the final videos used for GMA scoring were considered in this dataset. The mean corrected age was 14.6 weeks (+/- 2.1 weeks). Of the 931 infants that remained after applying exclusion criteria (see: Inclusion and exclusion criteria), 820 were scored as having FM+ and 105 infants were scored as FM-. The remaining six infants were scored as having abnormal movements (atypical FM) and were excluded from model training, but still included for pose-estimation and feature computation. The full list of excluded IDs can be found on the OSF pre-registration site (https://osf.io/gztmd/).

Inclusion and exclusion criteria

For children still hospitalized at the time of the fidgety-aged GMA, Early Detection Team members captured the videos in the hospital as part of standard care. Exclusions were applied to intubated patients, those under the influence of sedation medications, within a week post-operative, on ECMO support, or diagnosed with myelomeningocele. The full dataset comprised 1063 infants. 129 were then excluded for meeting one or more exclusion criteria listed

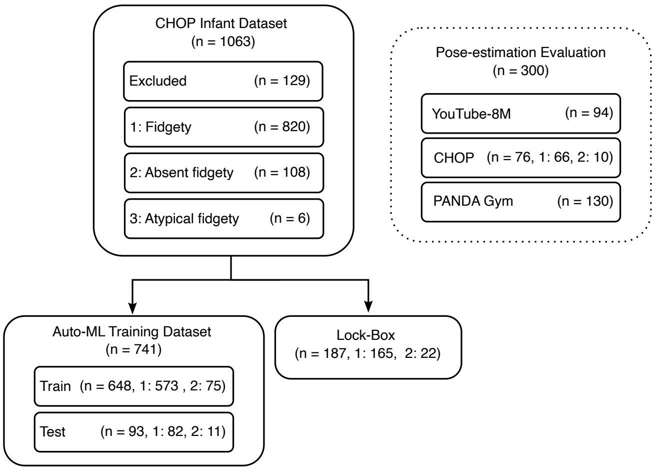

Figure 2. Datasets used for pose-estimation algorithm selection, AutoML training, and lock-box testing. The pose-estimation evaluation dataset included 94 videos from the YouTube-8M dataset, 76 videos from the training subset of the CHOP infant Dataset, and 130 additional videos of infants from ongoing projects in the lab. CHOP Infant Dataset had 1063 infants; 129 were excluded from the dataset for meeting one or more medical exclusion criteria prior to any analysis. training(648), test(93), and lock-box videos (187) each had apx. 12% representation of the positive class (FM-) and were pre-registered prior to pose-tracking algorithm selection and movement feature computation. The 6 atypical fidgety videos were excluded from model training, but are included in the released feature dataset.

above.

Six infants atypical FM were excluded from the AutoML Training and Lock-box datasets, since there were not enough infants in this group for multi-class training and prediction (Figure 2. The remaining 928 videos were split into an analysis set (741) and a lock box holdout set (187). The analysis set was further split into train and test sets (648, 93), each of which had a apx 12% representation of the FM- movement type.

The splits were stratified to preserve the ratios of male and female infants, as well as age, and race and ethnicity, and chronological age on upload date (Table 1 A). There was a total recording duration of 60-120 s per infant.

Analyses

Developing a pipeline for robust skeletal tracking

Performance and validation of skeletal tracking pipeline

Infant videos pose unique challenges for pose-estimation algorithms due to frequent irregular body poses, the presence of body-like objects (e.g., toys or cartoons), high levels of self-occlusion, and different body proportions relative to adults [32]. Historically, algorithms such as OpenPose [33] fail in such conditions, leading to unreliable pose estimates [22, 9, 24, 18]. Extensive fine-tuning

is often required to improve accuracy on each individual infant dataset [34, 24] making it difficult for researchers without the time and technical skills to do so unable to benefit from custom models. Typically, each research group will annotate a subset of their own data to fine-tune a model that works well for their specific dataset. However, at the time we were selecting an algorithm none of the fine-tuned algorithms referenced in the literature were readily available for testing, and they either required very specific input sizes, needed re-training, or were missing key information like model weights. Increasingly this is no longer the case as open fine-tuned models for infant pose-estimation continue to be released and updated [18, 24].

One of the challenges in fast-moving fields like computer vision is the rate at which new models are developed. The open-source MMPose framework we used for the pose-estimation step of our pipeline simplifies testing multiple algorithms with different weights and adopting new ones as they are released [26]. We first tested various pre-trained algorithms on a diverse infant-video set (Figure 2). We then compared their performance to our lab's previously fine-tuned OpenPose model [5]. As we did not have a ground-truth annotated subset of videos to benchmark against, we relied on feedback from experts trained in scoring the GMA regarding whether or not the skeletal tracking was sufficiently good that a trained human would be able to administer the GMA on animated videos of the keypoint data. We found that ViTPose-H [19] performed better than the alternatives we tested (Openpose, HRNet, PVTv2) [35, 36, 37, 33, 38], obviating the need for manual fine-tuning on our test data (Figure 3).

We have made it as easy as possible for researchers to perform pose estimation on their videos by using a method and algorithm that we provide as a Docker container, built on open-source code that also provides its own Docker container. The weights for ViTPose-H are available on HuggingFace from the ViTPose-H authors. Additionally, another group fine-tuned ViTPose for infant pose estimation. They have published the model weights on Zenodo [24]. Weights can be easily specified in the pose estimation code we provided.

Generalizability testing

The robustness and generalizability of ViTPose-H were validated through iterative review of pose estimates by clinicians trained in the GMA. We relied on expert judgment to determine whether the skeletal tracking was sufficiently robust for a clinician to assess fidgety movements from the keypoints alone.

To evaluate performance on videos beyond our primary dataset, ViTPose-H was also tested on two fully out-of-sample infant datasets from a separate project in the Rehabilitation Robotics Lab (”PANDA Gym”, n = 130; ages = 0-6 months, typically and atypically developing, 6 camera angles)[39], as well as on a set of 94 infant videos from the YouTube 8M dataset (total n = 130; ages 0–4 months, presumed typically developing, various camera angles) [5]. The algorithm produced consistent results across datasets (i.e., results that clinicians deemed to be sufficiently smooth), supporting its generalizability. To facilitate further testing and development, we have publicly released the ViTPose-H keypoints for the YouTube-8M videos. We propose that ViTPose-H offers a scalable and reliable solution for converting infant videos into skeletal tracking data without the need for fine-tuning, enabling broader applications in infant movement analysis.

Benchmarking against deep-learning models

To establish a baseline for how simple kinematic features perform relative to deep learning methods, we first surveyed openly available code from recent papers on automated GMA. The vast majority of repositories were incomplete, either lacking sufficient code to fully replicate the published methods or omitting pretrained model weights, preventing direct evaluation on out-of-sample data without re-labeling and re-training. Direct requests to authors for data and models were also largely unsuccessful, with many inquiries going unanswered and others explicitly declining to share resources.

One available implementation (STAM: Spatio-temporal Attention-based Model) [13] was complete and testable, and it initially appeared to show excellent performance (ROC-AUC = 0.86). However, we identified a statistical error in the code, which used record-wise splitting, allowing samples from the same infant to appear in both training and testing sets. This practice is a well-documented source of overfitting in medical data [40]. When we corrected this error, performance collapsed to an ROC-AUC of 0.60. By contrast, our pipeline achieves a substantially stronger ROC-AUC of 0.79 on the identical pre-registered data splits.

This finding underscores a broader point that progress in the field depends on open data and code sharing together with rigorous evaluation practices that minimize overfitting.

Scaling analysis

To demonstrate the effect of aggregating simple features, we performed a scaling analysis. We took the complete feature dataset and split it into training set sizes of 50, 100, 200, 400, and 800 with the same test set of 100 infants (12% FM-). We found that the improvement in ROC followed a power-law relationship, as indicated by the approximately linear trend in the log-log plot (Figure. 4).

Explainability and feature importance

To provide some measure of model explanation, we implemented a standard permutation importance test using the sklearn framework [41, 42]. This approach systematically permutes feature values to assess the extent to which model performance depends on each input. We found that knee and elbow features contributed most strongly to model performance, followed by the wrists and ankles (Table 2). However, features with measurable importance were distributed across all body parts and statistical descriptors (mean, IQR, standard deviation) (Figure 5). This is consistent with established understanding of GMA assessment, which relies on global, whole-body movement patterns rather than isolated features.

**Grouped feature importance (△ROC-AUC)**	
Knees	0.17 ± 0.06
Elbows	0.03 ± 0.02
Ankles	0.08 ± 0.05
Wrists	0.08 ± 0.03

Table 2. Grouped feature importance (△ROC-AUC) across different body joints. Higher values indicate greater effect on model performance when permuted.

At the same time, we emphasize that this type of analysis has important limitations. Because many features are highly correlated, their contributions cannot be cleanly disentangled. Moreover, placing too much weight on individual features, or using them as the basis for feature selection, risks overfitting and may reduce generalizability. As our scaling experiments demonstrate, model performance improves most reliably when aggregating across increasing sample size, rather than prioritizing any one feature set. Thus, while this analysis provides some intuition about which features may be more influential, we caution against overinterpretation and underscore that robust performance derives primarily from data breadth rather than feature selection.

Feature relevance and clinical interpretation

A set of 38 kinematic features was selected based on clinician input [43, 44], designed to capture the displacement, velocity, acceleration, and entropy of key body parts: wrists, ankles, elbows, and knees (Table 3).

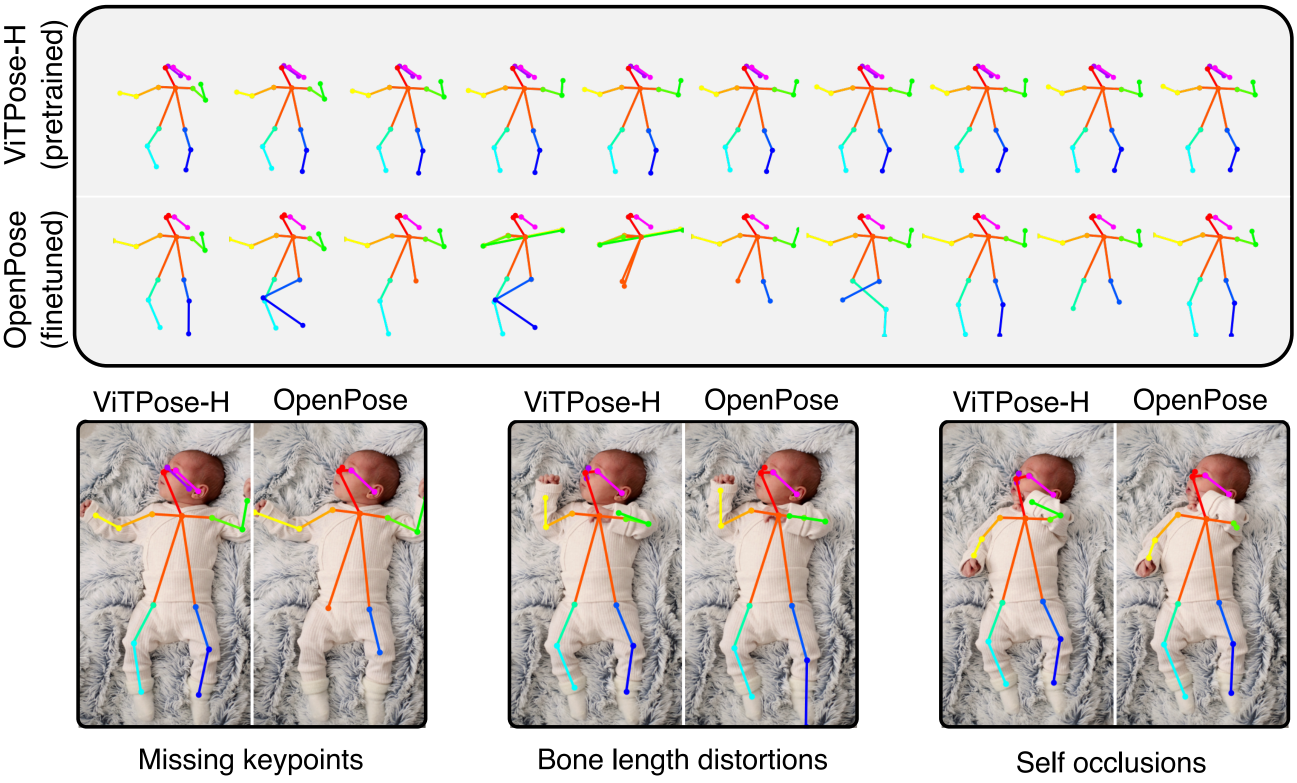

Figure 3. Improvements in skeletal tracking with pre-trained vision transformers. (Top panel) ViTPose-H (top row) produces consistent keypoint detections across frames in contrast to older algorithms like OpenPose (bottom row). (Bottom panel) Transformer-based approaches, such as ViTPose, learn adult skeletal priors and can infer missing keypoints (left), estimate bone lengths (center), and resolve self-occlusions (right) in infants.

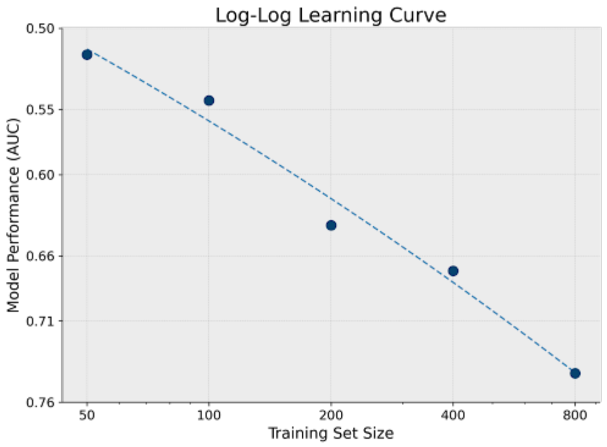

Figure 4. Improvements in model performance as a function of training-set size. More data improves model performance following a power-law relationship.

GMA-trained clinicians chose these 38 movement features because they are essential components of visual GMA scoring [4], though it is important to note that these are general kinematic descriptors that apply across contexts and age groups. We excluded any features directly related to GMA-specific FMs to capture general movement patterns, enabling future work on earlier risk prediction and CP subtype classification, and other movement disorders.

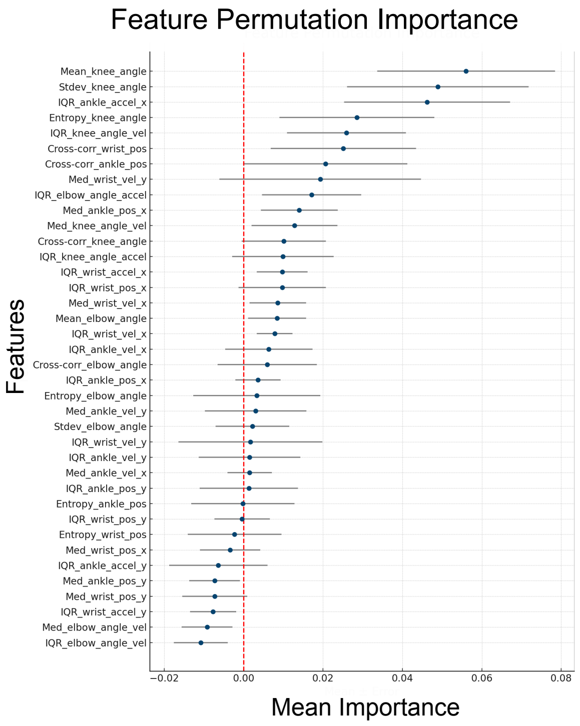

Figure 5. Permutation importance for each feature. Individual feature importance is low and highly variable, indicating that the model is using a combination of all features for predictions.

Model performance and validation

Generalizability and robustness of feature vector for risk prediction

Our initial analysis found considerable overlap in the 38 selected features for infants with and without fidgety movements (Figure 6), suggesting that no individual feature could differentiate high-risk infants. However, the aggregated feature vector was sufficient to predict GMA scores. Using the feature vector, the model achieved an ROC-AUC of 0.72 on the validation set, and 0.79 on the holdout

Table 3. Summary of position and angular features

**Position Features X & Y (ankles, wrists)**	
Cross-Correlation	Bilateral coordination/symmetry.
Entropy	Movement variability and complexity.
IQR Acceleration	Captures abruptness of movement.
IQR Position	Indicates range of movement in position.
IQR Velocity	Measure of movement smoothness.
Median Position	Captures postural biases or asymmetries.
Median Velocity	Typical speed of movement, reflecting consistency and effort.
**Angular Features (knees, elbows)**	
Cross-Correlation	Bilateral coordination/symmetry.
Entropy	Movement variability and complexity.
IQR Angular Acceleration	Proxy measure of spasticity/stiffness.
IQR Angular Velocity	Measure of movement smoothness.
Mean Joint Angle	Deviations may indicate abnormal tone.
Median Angular Velocity	Measure of rigidity.
Stdev Joint Angle	Reduced variance may signal restricted movement.

set. Five-fold cross-validation, repeated with six random seeds, yielded an average ROC-AUC of 0.73 ± 0.05. This demonstrates the model's robustness and internal generalizability.

We also computed the Precision-Recall Area Under the Curve (PR-AUC), a metric particularly informative for imbalanced datasets typical of clinical screening contexts. Given the positive class prevalence of approximately 12% in this holdout sample (FM-), the PR-AUC of 0.34 substantially exceeds the random chance baseline and highlights the model's utility in identifying at-risk infants.

The model's output was a continuous probability score indicating the likelihood of FM-. For the purposes of classification, we selected the operating threshold on the ROC curve that maximized the difference between the True Positive Rate (TPR) and False Positive Rate (FPR). This threshold is defined as:

$$\text{Threshold}_{\text{balanced}} = \arg \max_{t\in[0,1]} \{\text{TPR}(t) - \text{FPR}(t)\} \tag{1}$$

This corresponds to the threshold that maximizes Youden's J statistic (J = sensitivity + specificity − 1), ensuring balanced sensitivity and specificity in the context of class imbalance. However, alternative thresholds may be more appropriate depending on the clinical context.

A classification threshold of 0.65 on the ROC curve yields a TPR of 81.8% (18/22) and a FPR of 24.2% (40/165). Samples with a model score above this value were classified as positive (FM-), and those below were classified as negative (FM+) (Figure 7).

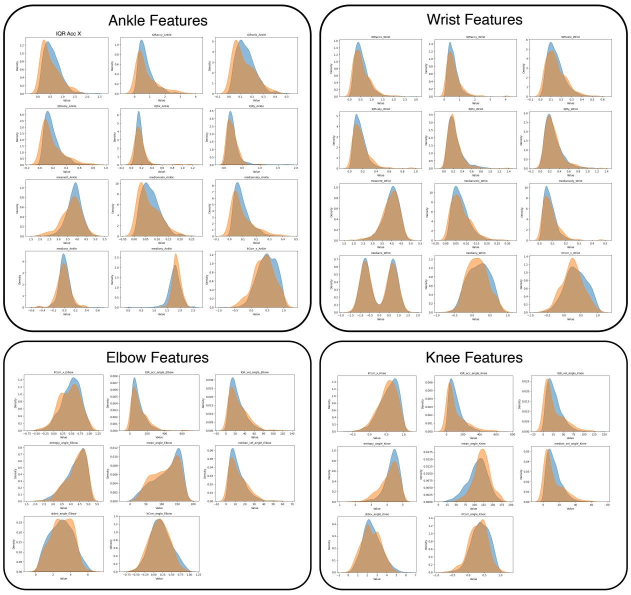

Figure 6. Individual feature distributions are highly correlated. Clinician-selected features, including XY features of the wrists/ankles and angular features of the elbows/knees, which are typically used for human assessment of risk are highly overlapping for FM+ (blue) and FM- (orange) movements, with no individual feature clearly predicting GMA score.

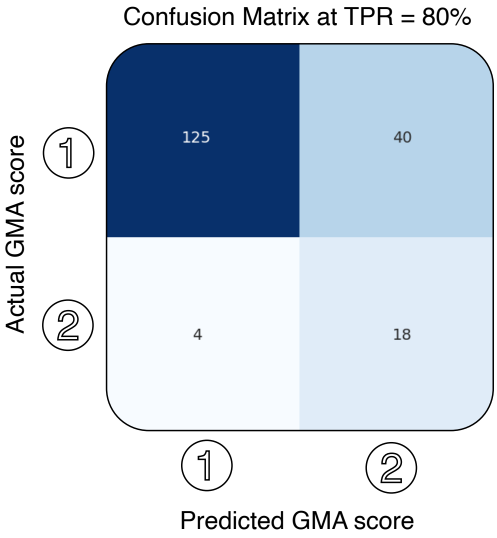

Figure 7. Confusion matrix for model predictions. A decision threshold was selected to achieve a TPR of 80% on the holdout set. GMA score 1 indicates FM+, and GMA score 2 indicates FM- movements based on expert GMA scoring. The threshold was optimized to balance sensitivity and specificity, though alternate thresholds can be based on clinical context.

Model training with Auto-sklearn 2.0

Experimenter-driven hyperparameter optimization and model selection are significant sources of bias in machine learning, often leading to models that don't generalize well [30]. Automated Machine Learning (AutoML) frameworks like Auto-sklearn 2.0 address this by abstracting these decisions [45, 41]. Instead of manual tuning, Auto-sklearn 2.0 systematically explores algorithm choices and hyperparameter settings, leveraging meta-learning—insights from previous experiments (i.e., other, fully independent datasets with similar statistical properties) to interpret the characteristics of the input data, such as class imbalance. This allows it to strategically select appropriate handling techniques like SMOTE, class weighting, and appropriate evaluation metrics (e.g., stratified cross-validation).

Auto-sklearn 2.0 uses Bayesian optimization to search model configuration spaces and can automatically construct ensembles of top-performing models. However, under strict "vanilla" settings, training time is restricted to 1 hour and the ensemble size restricted to one [41]. This constraint ensures a highly rigorous evaluation of individual models during the search phase. This approach increases the likelihood that the selected classifiers will generalize effectively to new, unseen data, ultimately leading to more robust and reliable

machine learning solutions.

Rigorous methods to prevent overfitting

To ensure that the model's performance was as unbiased and generalizable as we could achieve using data from only one site, a lock-box test set of 187 infants (22 FM-) was randomly selected before model training and pre-processing optimization. This lock-box dataset was only accessed after pre-registering all features, pre-processing steps, and algorithms. Testing on the lock-box set yielded a ROC-AUC of 0.79, and the precision–recall curve showed an PR-UAC of 0.34 (Figure 8). The ROC-AUC was closely aligned with the cross-validation performance indicating minimal overfitting. While both measures are lower than clinician performance reported in the literature and that of many other ML models, they are notable given the simplicity of the features and the stringent training parameters.

This rigorous validation suggests that our developed pipeline should generalize well to unseen data collected under similar conditions. It provides a reliable, reproducible approach for training models that identify infants at high risk for CP.

Discussion

Here we have developed an open, preregistered pipeline for predicting GMA score (a strong indicator of CP risk) from video-based pose estimates using rigorous methods. We used an exceptionally large sample (training set: 648, overall >1000 infants),a simple, generic movement-based feature vector and pre-registered each step before testing on a lock-box set of 187 infant videos. We found that our algorithm trained using this pipeline performs well (ROC-AUC 0.79, PR-AUC 0.34). We utilized an AutoML approach to minimize the risk of overfitting. We further minimized the risk of overly optimistic reporting by using a lock-box set and pre-registering our analyses and models. We have made our data, code, and algorithms publicly available on the OSF pre-registration site and GitHub. While further external validation is needed, especially regarding the use of pre-trained models for pose estimation, this approach increases confidence in the potential that the pipeline will generalize across datasets, thereby facilitating efforts to share feature datasets and train models across data from multiple sites.

While the GMA has been shown to have a high level of sensitivity and specificity in clinical settings, we did not predict the main important target future outcome – diagnosis of CP – as long-term outcomes were not available at the time of model training. Instead we predicted GMA, a clinician powered risk measure. This is common throughout the automated CP risk prediction literature, with multiple research groups focusing on predicting GMA score, or detecting FMs directly, as opposed to predicting CP diagnosis. This approach is not ideal, as it introduces an additional source of noise from potential human error during assessment, in addition to the noise inherent in the GMA assessment itself. FMs, while highly indicative, are still not a perfect biomarker for CP and multiple items are necessary for CP diagnosis (biomarkers, clinical history, functional motor assessment, and neurological assessment). Overreliance on FMs risks missing other, perhaps more indicative features or combinations of features that are not readily apparent, and limits risk analyses to the 3-4 month age window. Moreover, the extremely low prevalence of Abnormal FMs makes training a model that captures this movement type infeasible, meaning that some infants at high risk are often not accounted for in models trained only to detect FMs (or their absence). Future efforts should focus directly on predicting CP outcomes, subtypes, and severity.

Our current movement features are likely suboptimal for detecting subtle movement differences. The reason we say this confidently is that others have obtained much better results using approaches optimized to detect FMs. While our features are capturing some differences between groups, they are clearly missing some of the subtle movements others have captured with the direct FM featurization. The clinician-selected movement features offer only a coarse description of movement averaged over large windows, whereas we know from the clinical literature that the difference between infants whose movements are typically developing and those that are not is often subtle. For instance, infrequent, small amplitude rolls of the wrists and ankles carry significant clinical meaning, but are infrequent and may be smoothed out when averaged over an entire video. This is especially true when aiming for early prediction before the 3–4-month window. It is also a concern for general-population pre-screening, where movement differences may be even more subtle.

Many efforts have been made to identify a precise featurization using machine learning (e.g., [14, 15, 9]). However all of these efforts risk overfitting since the prevalence of FM- infants is relatively small (even in large datasets). The prevalence of infants who develop specific subtypes, or specific levels of severity, is even lower.

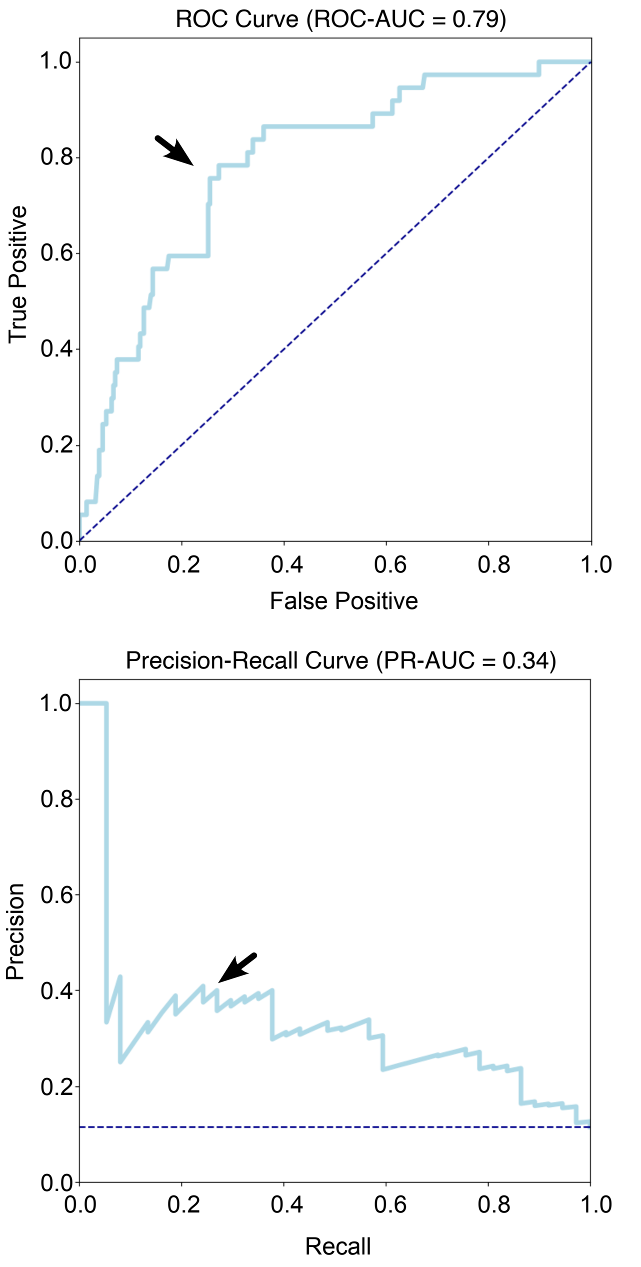

Figure 8. Model generalizes to lock-box set. Classifier trained on clinician-selected features using vanilla Auto-sklearn 2.0 shows a high ROC-AUC of 0.79 (Left) and Precision-Recall of 0.34 (Right) on lock-box set of 187 infants, having 12% representation of FM-. True positive rate is equal to the Sensitivity of the classifier, False positive rate is equal to 1-Specificity.

Pooling data across sites—by sharing a standard set of de-identified features and adopting approaches like AutoML—would enable training on much larger datasets. Models trained on these larger datasets may be able to capture more subtle differences, boosting performance, and enabling even earlier, more precise prediction of CP.

Our current work did not investigate several contextual factors critical for the real-world deployment and performance of predictive models. Future validation must assess the pipeline's robustness to variations in video quality—such as those from less optimal recording conditions potentially encountered in under-resourced settings—and systematically evaluate the influence of diverse infant skin tones, lighting conditions, and backgrounds on pose estimation accuracy and subsequent predictions. Furthermore, the clinical heterogeneity of CP, which encompasses multiple subtypes and a broad spectrum of severity, is not fully captured by global risk scores like the GMA. A key future direction is therefore to develop models that not only quantify overall risk but also aim to differentiate CP subtypes, leveraging the hypothesis that these distinctions manifest as unique patterns within the movement feature space. Moving towards more nuanced predictions hinges on collecting larger, more diverse datasets with sufficient CP-subtype representation. Achieving this will require large-scale collaborative efforts.

The wide range of ages at which CP is typically diagnosed reflects the fact that less severe movement deficits are often not evident to untrained observers until later in an infant's development. In contrast, indicators of more severe impairment may be evident to clinicians (and caregivers) much earlier. The infants included in the model all spent time after birth in the Neonatal Intensive Care Unit (NICU), meaning that they were already at an elevated risk of CP. This limitation is prevalent throughout the automated CP detection literature [8, 10, 11, 16], since collecting videos of infants for the purposes of training a ML prediction model is most feasible in a hospital setting. As such the movements that distinguish the two groups in our sample may not be representative of infants from the general infant population. However, other people have shown that movement features can be used to predict GMA scores in at-home videos of infants that are not at high risk [14], so the approach should generalize if trained on the bigger sample that also includes infants from the general population. This should be imminently feasible now that we have released a pose estimation and pre-processing pipeline that is open, easy to share, and does not require fine-tuning.

We have shown that a simple movement-based automated prediction approach works in an very large sample. Our model's performance, with an ROC-AUC of 0.79 and a PR-AUC of 0.34, should be viewed as particularly encouraging given it was achieved under rigorous, pre-registered conditions, indicating a genuine predictive signal from relatively simple features. For any such pre-screening tool, the critical challenge lies in balancing sensitivity and specificity: a high false positive rate can unnecessarily worry parents and overburden healthcare systems with healthy children, whereas low sensitivity risks missing children who need clinical intervention, especially in low-resource settings. While our current model demonstrates that achieving a level of predictive accuracy on a large dataset is feasible even under these strict methodological constraints, it clearly requires enhanced precision for broad application. We contend that the most promising path to such improvements is through substantially increasing data scale and diversity by pooling data across many clinical sites. This becomes truly achievable when we prioritize models designed to generalize effectively across different sites and embrace the sharing of de-identified features, an approach our pipeline is built to facilitate.

Potential implications

One of the biggest limitations in infant CP research is the difficulty in sharing videos due to privacy and safety concerns. There are a growing number of research sites with datasets of over 1000 infant videos, ours is only one example of such a dataset. What's needed is dedicated effort to combine these datasets across sites. While video-based pose estimation for infants is common in the automated detection literature, each site typically uses their own custom, fine-tuned algorithm and a post-processing pipeline tailored to its dataset. This approach hinders broader collaboration and generalizability, though as noted previously, this is increasingly not the case, reflecting a broader shift towards pre-trained models that work across datasets [18, 24].

Here, we present a framework for computing and sharing de-identified features, and training models on the aggregated datasets using AutoML, thereby making it as easy as possible for other researchers to collect large video datasets, compute features, combine across datasets, and train better classifiers. All of the methods used are ethologically doable on a phone camera. All of the videos used for these analyses were collected using hand-held iPhones/iPads. The pre-trained vision-transformer we used was not fine-tuned on any of the infant videos, and was tested on a wide range of different datasets producing stable results across all of them, as assessed by clinicians. As such we expect that it will work equally well at other clinical sites and on at-home videos. Other researchers have since tested ViTPose-H on infant data and found good performance, and they have released fine-tuned weights specific to infants that they show perform even better [24] and can be specified in the pose-estimation pipeline we provide.

We have shown that advances in pose-estimation now make it entirely realistic to get movement features from infant videos without the need for any specialized camera setup or fine-tuning. We have shown that movement features derived from these pose estimates predict GMA scores in a very large sample, and that our model performs well on the lock-box test set. The simplified process of obtaining de-identified features means that training on datasets across multiple sites and various contexts should now be possible. This facilitates joint efforts and holds tremendous potential for the creation of a global prescreening tool, especially if we boost performance using deep-learned features (including FMs that others have shown work extremely well at 3-4 months), train on more data including from many infants across sites (power-law scaling), and predict CP outcomes as opposed to proxy clinical scores like the GMA.

The capacity to openly share derived movement features (unlike raw videos or detailed keypoints) is crucial. While the development of sophisticated data sharing infrastructures, such as federated learning systems, is a valuable long-term goal, this process often requires considerable time and coordination. To accelerate progress in the interim, our approach leverages features that are not only easy to compute from common video recordings but have also shown predictive power. Their suitability for standardization across multiple clinical sites and developmental stages, combined with their potential for open release, offers an immediate and pragmatic pathway to foster collaboration and build richer aggregated datasets for CP research.

Methods

Developing a Pipeline for Robust Skeletal Tracking

Selecting a pose estimation algorithm

To estimate infant pose from monocular hand-held video, we implemented a top-down 2D pose estimation pipeline using tools from the open-source library OpenMMLab. MMDetection was used for infant detection [26], and MMPose was used for 2D pose estimation [26]. Infant detection was performed using an RTMDet [46] model pre-trained on the Common Objects in Context (COCO) dataset [47]. 2D frame-wise pose estimation was carried out using ViTPose-H [19], a 10B parameter vision transformer, selected for

its cross-domain performance.

Processing keypoint timeseries data

Pose estimation was conducted on CHOP high-performance computing servers, ensuring compliance with ethics guidelines by restricting access to CHOP staff and authorized researchers only.

In each frame, only the highest-confidence detection was used, and frames with keypoint confidence scores below 0.8 were excluded. This process still allowed us to retain over 90% of frames. As in Chambers et al. [5], missing frames were linearly interpolated; outliers were removed with a rolling-median filter (1-second window); and data were smoothed with a rolling-mean filter (1-second window). The effect of smoothing can be seen in Figure 9.

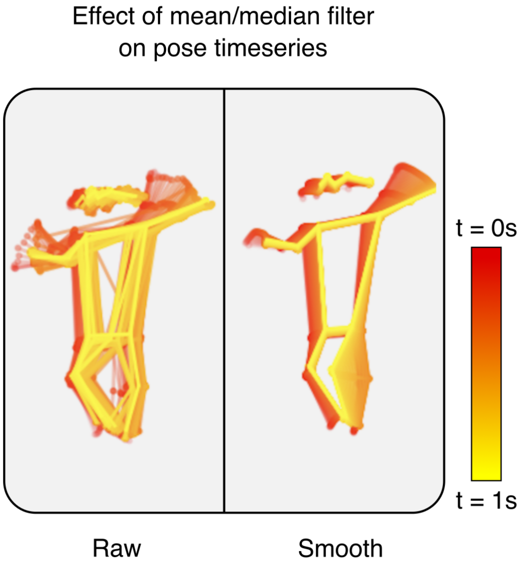

Figure 9. Raw vs smooth pose estimates over a 1s window. Raw timeseries has inter-frame jitter resulting in noisy timeseries (left). The mean and median filters (1s) reduce noise (right), but risks losing variability that may be diagnostically significant, particularly in the wrists/ankles.

Future work will include a systematic evaluation of our preprocessing pipeline, particularly the impact of data smoothing. The application and intensity of smoothing techniques can profoundly influence derived kinematic features, potentially obscuring or altering subtle dynamic characteristics critical for distinguishing between movement patterns. While smoothing is a common step to manage noise from pose estimation, the extent to which it affects diagnostically relevant information, such as small-amplitude variability, is not always clear. Therefore, a key future objective will be to systematically quantify these effects. Understanding how different smoothing strategies modify key kinematic outputs will be crucial for optimizing our preprocessing pipeline. This will help preserve clinically meaningful movement details and maximize the analysis's diagnostic potential.

We chose not to standardize the length of videos, even though it could affect measures of variability (notably standard deviation), for the following reasons. First, an initial check confirmed that the average video duration did not significantly differ between our positive and negative outcome groups, mitigating concerns that video length could act as a systematic confounder at the group level. More importantly, each video was recorded by clinicians with the explicit aim of capturing a sufficient epoch of movement to reliably perform the General Movements Assessment. Thus, the duration of each recording reflects a clinically determined window deemed adequate for observation. The raw standard deviation within such a window directly reflects the movement variability pertinent to this clinical judgment. Normalizing by total frame count might obscure true differences since the proportion of frames containing active, analyzable movement could vary from one clinically sufficient recording to another.

Dataset Split and Pre-registration

Following pose estimation, infant IDs were divided into training, validation, and lock-box test sets using a stratified split to preserve a 12% representation of FM-, as well as the ratio of male and female infants, and race and ethnicity as described in Table 1. The video IDs corresponding to each split were pre-registered prior to conducting further analyses, ensuring a clear separation between training, validation, and test datasets, and the lock-box test set.

Kinematic Feature Computation

After pre-registration and pre-processing, a set of 38 kinematic features were computed from the smoothed keypoint timeseries using open-source Python code [48] which was adapted from previous work [5]. All features were computed after smoothing the dataset, with frame-wise calculations of joint angles, velocities, and accelerations, which were then aggregated either over the entire video or within overlapping 2-second sliding windows (including rest periods). These features captured displacement, velocity, acceleration, and entropy of the extremities (wrists and ankles) and joint angles (elbows and knees) (Table 3).

No specific features related to GMA FMs were included to minimize the risk of overfitting to the clinical dataset, but may be integrated into future releases.

Model Training

Feature selection and pre-registration

A binary classifier was trained to predict FM- infants, which indicates a higher risk of developing CP [2, 3, 4, 49, 50]. To reduce the risk of overfitting, feature selection was conducted in consultation with clinicians prior to any data analysis and was pre-registered in 2018 [43, 44].Feature computation was automated using custom Python code available on GitHub [48]. Computed features were also pre-registered prior to model training.

We did not perform post-hoc iterative feature selection as doing so may lead to overfitting.

AutoML framework for model training

Model selection and hyperparameter optimization were carried out using the Auto-sklearn 2.0 package [41, 45], with the "vanilla auto-sklearn" setting (see section: Model training with Auto-sklearn 2.0). This configuration limited the ensemble size to one, ensuring that the model with the best validation performance was selected. Balanced accuracy was chosen as the optimization metric due to the class imbalance (approximately 10:1) [51]. A meta-feature-free portfolio was used for efficient meta-learning, and training/validation splits were managed with successive halving. Cross-validation with five folds was employed to validate model generalizability across different training/validation splits, and the resulting model was pre-registered on May 22, 2024, before testing on the lockbox test set [43].

Availability of source code and requirements

The source code for skeletal tracking, feature computation, and classifier training has been made available on GitHub at https://doi.org/10.5281/zenodo.14674148. The original feature com-

putation code, from which this work is derived, can be found at https://github.com/quietscientist/Infant_movement_assessment.

Data availability

The dataset supporting the results of this article is available on the OSF repository (https://osf.io/gztmd/) and contains participant IDs, data splits, movement features, and clinical scores [43].

In accordance with ethics guidelines and institutional review board (IRB) approvals from the University of Pennsylvania (Penn) and the Children's Hospital of Philadelphia (CHOP), study data containing identifiable information are subject to strict handling protocols. Raw videos can only be processed on-site at CHOP and cannot be shared externally. Similarly, raw pose-estimates are classified as Protected Health Information (PHI) by the Penn and CHOP IRBs and are therefore not publicly available.

Researchers interested in accessing the raw pose estimate time-series data can request to be added to the IRB protocol by contacting the corresponding author (prosserl@chop.edu). This process requires completion of mandatory training in HIPAA regulations and relevant clinical research practices. Additional screening by The University of Pennsylvania or CHOP may also be necessary.

The computed movement features from this study, as well as a dataset computing features over a 2-second sliding window to preserve temporal information, have been made available on the project's OSF Pre-registration site: osf.io/gztmd/files/osfstorage

For methodological transparency, the pose estimate data from the YouTube-8M subset (94 infants), which was used in selecting our pose estimation algorithm are also available on Figshare: doi.org/10.6084/m9.figshare.25316500.

Declarations

List of Abbreviations

ROC-AUC Receiver Operating Characteristic Area Under the Curve
PR-AUC Precision Recall Area Under the Curve
CHOP Children's Hospital of Philadelphia
COCO Common Objects in Context
CP Cerebral Palsy
ECMO Extracorporeal Membrane Oxygenation
FM Fidgety Movement
FM+ Fidgety Movements Present (GMA Score 1)
FM- Absent Fidgety Movements (GMA Score 2)
FPR False Positive Rate
GM General Movement
GMA General Movements Assessment
IQR Inter-Quartile Range
ML Machine Learning
MRI Magnetic Resonance Imaging
NICU Neonatal Intensive Care Unit
TPR True Positive Rate

Ethical Approval

Ethical approval for this study was provided by the University of Pennsylvania (Penn) Institutional Review Board (IRB Protocol Number: 833180), acting as the single IRB or record and a subsequent reliance agreement between Penn and the Children's Hospital of Philadelphia (CHOP) Institutional Review Board (IRB Protocol Number: 19-016641).

Consent for publication

The infant image used in Figure 3 to illustrate algorithm performance is a video frame taken from Adobe Stock Video (ID: #702309262), the use of which is permitted under the Adobe Stock Extended License.

Competing Interests

The author(s) declare that they have no competing interests.

Funding

This work was funded by an NIH-NICHD grant (Project#: 1R01HD097686, PIs: Johnson, Michelle J. and Kording, Konrad P.) and the clinical Early Detection Trial data collection was supported in part by the Cerebral Palsy Foundation.

Author's Contributions

Konrad P. Kording, Michelle J. Johnson, Laura Prosser, and Melanie Segado were responsible for conceptualization of the study aims. Data curation was performed by Andrea F. Duncan, Laura Prosser, and Melanie Segado. Melanie Segado conducted the formal analysis and developed the software. Funding acquisition was led by Konrad P. Kording, Michelle J. Johnson, and Laura Prosser. Data collection and clinical evaluation were carried out by Andrea F. Duncan and Laura Prosser. Methodology was established by Konrad P. Kording and Melanie Segado, with input from Laura Prosser and Michelle J. Johnson. The original draft was written by Melanie Segado and Konrad P. Kording, and all authors contributed to the review and editing of the manuscript.

Acknowledgements

The authors would like to thank Felipe Parodi for help implementing the pose estimation pipeline, and O. Francis Sowande for iterative testing on out-of-sample data. They would also like to thank Julie Skorup, , PT, DPT, PCS and Audrey J Wood, MS, PT, PCS for validation of the skeletal tracking outputs.

References

1. McIntyre S, Goldsmith S, Webb A, et al. Global prevalence of cerebral palsy: A systematic analysis. Dev Med Child Neurol 2022;64(12):1494–1506.
2. Novak I, Morgan C, Adde L, et al. Early, Accurate Diagnosis and Early Intervention in Cerebral Palsy: Advances in Diagnosis and Treatment. JAMA Pediatr 2017;171(9):897–907.
3. Herskind A, Greisen G, Nielsen JB. Early identification and intervention in cerebral palsy. Dev Med Child Neurol 2015;57(1):29–36.
4. Einspieler C, Prechtl HFR. Prechtl's assessment of general movements: A diagnostic tool for the functional assessment of the young nervous system. Ment Retard Dev Disabil Res Rev 2005;11(1):61–67.
5. Chambers C, Seethapathi N, Saluja R, et al. Computer Vision to Automatically Assess Infant Neuromotor Risk. IEEE Trans Neural Syst Rehabil Eng 2020;28(11):2431–2442.
6. Gao Q, Yao S, Tian Y, et al. Automating General Movements Assessment with quantitative deep learning to facilitate early screening of cerebral palsy. Nat Commun 2023;14(1):8294.
7. Adde L, Brown A, van den Broeck C, et al. In-Motion-App for remote General Movement Assessment: a multi-site observational study. BMJ Open 2021;11(3):e042147.

8. Hashimoto Y, Furui A, Shimatani K, et al. Automated Classification of General Movements in Infants Using a Two-stream Spatiotemporal Fusion Network. arXiv 2022;http://arxiv.org/abs/2207.03344, published online July 4, 2022. Accessed September 27, 2023.
9. Groos D, Adde L, Aubert S, et al. Development and Validation of a Deep Learning Method to Predict Cerebral Palsy From Spontaneous Movements in Infants at High Risk. JAMA Netw Open 2022;5(7):e2221325.
10. Irshad MT, Nisar MA, Gouverneur P, Rapp M, Grzegorzek M. AI Approaches towards Prechtl's Assessment of General Movements: A Systematic Literature Review. Sensors 2020;20(18):5321.
11. Kwong AKL, Doyle LW, Olsen JE, et al. Parent-recorded videos of infant spontaneous movement: Comparisons at 3–4 months and relationships with 2-year developmental outcomes in extremely preterm, extremely low birthweight and term-born infants. Paediatr Perinat Epidemiol 2022;36(5):673–682.
12. Morais R, Le V, Morgan C, et al. Robust and Interpretable General Movement Assessment Using Fidgety Movement Detection. IEEE J Biomed Health Inform 2023;p. 1–12.
13. Nguyen-Thai B, Le V, Morgan C, Badawi N, Tran T, Venkatesh S. A Spatio-temporal Attention-based Model for Infant Movement Assessment from Videos. IEEE J Biomed Health Inform 2021;25(10):3911–3920.
14. Passmore E, Kwong AL, Greenstein S, et al. Automated identification of abnormal infant movements from smart phone videos. PLOS Digit Health 2024;3(2):e0000432.
15. Redd CB, Karunanithi M, Boyd RN, Barber LA. Technology-assisted quantification of movement to predict infants at high risk of motor disability: A systematic review. Res Dev Disabil 2021;118:104071.
16. Silva N, Zhang D, Kulvicius T, et al. The future of General Movement Assessment: The role of computer vision and machine learning – A scoping review. Res Dev Disabil 2021;110:103854.
17. Spittle AJ, Olsen J, Kwong A, et al. The Baby Moves prospective cohort study protocol: using a smartphone application with the General Movements Assessment to predict neurodevelopmental outcomes at age 2 years for extremely preterm or extremely low birthweight infants. BMJ Open 2016;6(10):e013446.
18. Ostadabbas S, Fine-tuned Domain-adapted Infant Pose (FiDIP); 2023. https://github.com/ostadabbas/Infant-Pose-Estimation, published online August 17, 2023. Accessed September 6, 2023.
19. Xu Y, Zhang J, Zhang Q, Tao D. ViTPose++: Vision Transformer for Generic Body Pose Estimation. arXiv 2023;Published online December 14, 2023.
20. Liu W, Bao Q, Sun Y, Mei T. Recent Advances of Monocular 2D and 3D Human Pose Estimation: A Deep Learning Perspective. ACM Comput Surv 2022;55(4):80:1–80:41.
21. Wei K, Kording KP. Behavioral tracking gets real. Nat Neurosci 2018;21(9):1146–1147.
22. Seethapathi N, Wang S, Saluja R, Blohm G, Kording KP, Movement science needs different pose tracking algorithms; 2019. Published online July 23, 2019.
23. Hesse N, Bodensteiner C, Arens M, Hofmann UG, Weinberger R, Schroeder AS. Computer Vision for Medical Infant Motion Analysis: State of the Art and RGB-D Data Set. In: Computer Vision - ECCV 2018 Workshops Springer International Publishing; 2018. .
24. Jahn L, Flügge S, Zhang D, Poustka L, Bölte S, Wörgötter F, et al. Comparison of marker-less 2D image-based methods for infant pose estimation. Scientific Reports 2025;15(1):12148.
25. Huang X, Luan L, Hatamimajoumerd E, Wan M, Kakhaki PD, Obeid R, et al. Posture-based infant action recognition in the wild with very limited data. In: Proceedings of the IEEE/CVF Conference on Computer Vision and Pattern Recognition; 2023. p. 4912–4921.
26. Contributors M, OpenMMLab Pose Estimation Toolbox and Benchmark; 2020. https://github.com/open-mmlab/mmpose.
27. Contributors M, OpenMMLab Detection Toolbox and Benchmark; 2018. https://github.com/open-mmlab/mmdetection.
28. Ihlen EAF, Støen R, Boswell L, et al. Machine Learning of Infant Spontaneous Movements for the Early Prediction of Cerebral Palsy: A Multi-Site Cohort Study. J Clin Med 2020;9(1):5.
29. Powell M, Hosseini M, Collins J, et al, I Tried a Bunch of Things: The Dangers of Unexpected Overfitting in Classification; 2020. Published online February 14, 2020.
30. Hosseini M, Powell M, Collins J, et al. I tried a bunch of things: The dangers of unexpected overfitting in classification of brain data. Neurosci Biobehav Rev 2020;119:456–467.
31. Cutland CL, Lackritz EM, Mallett-Moore T, Bardají A, Chandrasekaran R, Lahariya C, et al. Low birth weight: Case definition & guidelines for data collection, analysis, and presentation of maternal immunization safety data. Vaccine 2017;35(48):6492–6500.
32. Sciortino G, Farinella GM, Battiato S, Leo M, Distante C. On the estimation of children's poses. In: Image Analysis and Processing-ICIAP 2017: 19th International Conference, Catania, Italy, September 11-15, 2017, Proceedings, Part II 19 Springer; 2017. p. 410–421.
33. Cao Z, Hidalgo G, Simon T, Wei SE, Sheikh Y. OpenPose: Realtime Multi-Person 2D Pose Estimation Using Part Affinity Fields. IEEE Trans Pattern Anal Mach Intell 2021;43(1):172–186.
34. Groos D, Adde L, Støen R, Ramampiaro H, Ihlen EA. Towards human-level performance on automatic pose estimation of infant spontaneous movements. Computerized Medical Imaging and Graphics 2022;95:102012.
35. Wang W, Xie E, Li X, et al. PVT v2: Improved baselines with Pyramid Vision Transformer. Comput Vis Media 2022;8(3):415–424.
36. Mathis A, Mamidanna P, Cury KM, et al. DeepLabCut: markerless pose estimation of user-defined body parts with deep learning. Nat Neurosci 2018;21(9):1281–1289.
37. Pereira TD, Tabris N, Matsliah A, et al. SLEAP: A deep learning system for multi-animal pose tracking. Nat Methods 2022;19(4):486–495.
38. Toshev A, Szegedy C. DeepPose: Human Pose Estimation via Deep Neural Networks. In: 2014 IEEE Conference on Computer Vision and Pattern Recognition; 2014. p. 1653–1660.
39. Panchal J, Sowande OF, Prosser L, Johnson MJ. Design of pediatric robot to simulate infant biomechanics for neurodevelopmental assessment in a sensorized gym. In: 2022 9th IEEE RAS/EMBS International Conference for Biomedical Robotics and Biomechatronics (BioRob) IEEE; 2022. p. 1–7.
40. Saeb S, Lonini L, Jayaraman A, Mohr DC, Kording KP. The need to approximate the use-case in clinical machine learning. GigaScience;6(5):gix019. https://doi.org/10.1093/gigascience/gix019, _eprint: https://academic.oup.com/gigascience/article-pdf/6/5/gix019/60708852/gigascience_6_5_gix019.pdf.
41. Feurer M, Klein A, Eggensperger K, Springenberg J, Blum M, Hutter F. Efficient and Robust Automated Machine Learning. In: Advances in Neural Information Processing Systems, vol. 28 Curran Associates, Inc.; 2015. https://proceedings.neurips.cc/paper_files/paper/2015/file/11d0e6287202fced83f79975ec59a3a6-Paper.pdf.
42. Molnar C. Interpretable Machine Learning. 3 ed.; 2025. https://christophm.github.io/interpretable-ml-book.
43. Segado M, Update: Predicting clinical assessments of infants' risk of neuromotor disease from 2-dimensional videos; 2023. 10.17605/OSF.IO/SD6FA.
44. Chambers C, Predicting clinical assessments of infants' risk of neuromotor disease from 2-dimensional videos; 2018.
45. Feurer M, Eggensperger K, Falkner S, Lindauer M, Hutter F, Auto-Sklearn 2.0: Hands-free AutoML via Meta-Learning; 2022.

46. Lyu C, Zhang W, Huang H, et al, RTMDet: An Empirical Study of Designing Real-Time Object Detectors; 2022. https://arxiv.org/abs/2212.07784.
47. Lin TY, Maire M, Belongie S, et al, Microsoft COCO: Common Objects in Context; 2015.
48. Segado M, Chambers MJKK, Seethapathi N, Saluja R, Prosser L, Infant Movement Assessment: Update; 2024. https://github.com/quietscientist/Infant_movement_assessment.
49. Ferrari F, Cioni G, Einspieler C, et al. Cramped Synchronized General Movements in Preterm Infants as an Early Marker for Cerebral Palsy. Arch Pediatr Adolesc Med 2002;156(5):460–467.
50. Einspieler C, Yang H, Bartl-Pokorny KD, et al. Are sporadic fidgety movements as clinically relevant as is their absence? Early Hum Dev 2015;91(4):247–252.
51. Pedregosa F, Varoquaux G, Gramfort A, et al. Scikit-learn: Machine Learning in Python. J Mach Learn Res 2011;12:2825–2830.

Click here to access/download

Supplementary Material

TRIPODAI_checklist_MSegado.pdf
