# Supplementary material for: A preregistered, open pipeline for early cerebral palsy risk assessment from infant videos
Source: Gigascience. 2026 Jan 20;15:giag003. doi: 10.1093/gigascience/giag003 (PMC13152017; doi:10.1093/gigascience/giag003)
Supplement: giag003_GIGA-D-24-00511_Revision_3 [file giag003_giga-d-24-00511_revision_3.pdf]

GigaScience

A Pre-Registered, Open Pipeline for Early Cerebral Palsy Risk Assessment from Infant Videos

--Manuscript Draft--

**Manuscript Number:**	GIGA-D-24-00511R3
**Full Title:**	A Pre-Registered, Open Pipeline for Early Cerebral Palsy Risk Assessment from Infant Videos
**Article Type:**	Technical Note
**Funding Information:**	National Institute of Child Health and Human Development (1R01HD097686) — Dr. Konrad P Kording<br>Cerebral Palsy Foundation — Dr. Andrea F Duncan
**Abstract:**	Cerebral Palsy (CP), affecting approximately 1 in 500 children due to abnormal brain development, impacts movement control. Early risk assessment via the General Movements Assessment (GMA) at 3–4 months is highly predictive for CP but relies on trained clinicians. Machine-learning-based approaches for predicting GMA score from video have shown considerable promise, but typically rely on dataset-specific preprocessing, custom feature sets, and manually designed model pipelines, which make external benchmarking more difficult. This, combined with strict privacy constraints on sharing data, makes it challenging to train and evaluate models across datasets, which is important for assessing clinical utility. There is therefore a need to develop approaches that work across different datasets to enable multi-site dataset aggregation and model training. To address this gap, we developed an end-to-end pipeline that uses off-the-shelf pose estimation, general-purpose feature extraction, and automated machine learning, none of which are tuned to a specific dataset. We applied this approach to a newly generated large dataset of 1063 infants (with approximately 12% positive class for adverse GMA outcome, drawn from a high-risk clinical cohort) within a preregistered study design. Model performance was evaluated on a strict “lock-box” test set, which remained untouched during any phase of model development or preprocessing optimization and was used only once for final evaluation after preregistration. The developed model achieved moderate predictive accuracy for clinician-assessed GMA scores (Area Under the Receiver Operating Characteristic Curve, ROC-AUC = 0.79; Area Under the Precision-Recall Curve, PR-AUC = 0.34). The moderate accuracy is noteworthy given the 12% positive class prevalence and the power-law scaling of ROC-AUC as a function of increasing dataset size. By releasing de-identified feature data and open-source code, and simplifying the training pipeline using AutoML, our work establishes essential groundwork for future robust, globally relevant CP screening tools suitable for low-resource settings.
**Corresponding Author:**	Melanie Segado<br>University of Pennsylvania School of Engineering and Applied Science<br>Philadelphia, UNITED STATES
**Corresponding Author Secondary Information:**	
**Corresponding Author's Institution:**	University of Pennsylvania School of Engineering and Applied Science
**Corresponding Author's Secondary Institution:**	
**First Author:**	Melanie Segado, PhD
**First Author Secondary Information:**	
**Order of Authors:**	Melanie Segado, PhD<br>Laura A Prosser, PT, PhD<br>Andrea F Duncan, MD, MS<br>Michelle J Johnson, PhD

Powered by Editorial Manager® and ProduXion Manager® from Aries Systems Corporation

	Konrad P Kording, PhD
**Order of Authors Secondary Information:**	
**Response to Reviewers:**	Dear Editorial Team,<br><br>Thank you for facilitating this most recent round of reviews and for the guidance throughout the process. We have carefully revised the manuscript and, to the best of our knowledge, addressed all remaining revisions.<br><br>In this revision, we have:<br>- Revised wording throughout to avoid vague or informal phrasing.<br>- Added additional references where appropriate to support key claims.<br>- Updated and corrected all figures, captions, and legends.<br>- Restructured sections to improve logical flow and ensure alignment with the journal's guidelines.<br>- Corrected remaining grammatical and typographic issues.<br><br>In response to the reviewer's comments regarding acknowledgment of prior work, we have substantially revised the relevant section to more clearly and accurately describe the use of external validation sets in this literature.<br>We appreciate the reviewer's thoughtful feedback, which has improved the clarity and precision of the manuscript.<br><br>Thank you again for your time and guidance.<br><br>Sincerely,<br><br>Melanie Segado (on behalf of all co-authors)<br><br>– Direct Response to Reviewer #1 –<br><br>Thank you for the detailed and constructive feedback. We have carefully revised the manuscript to address all of the points raised, with particular attention to clarifying terminology, improving tone and accuracy, and refining the figures, tables, and accompanying descriptions.<br><br>1. Reviewer: "If these relevant movement features can be reliably computed from videos, then algorithmic approaches for predicting infant risk from movement features should perform robustly."<br>This sentence should be made more neutral, e.g., "…this indicates potential for algorithmic approaches..."<br><br>Response: Thank you for this helpful suggestion. The sentence has been revised to: "If these relevant movement features can be reliably computed from videos, it suggests that algorithmic approaches could feasibly be applied to estimate infant risk from movement features."<br><br>This phrasing is more neutral, and written with a more suitable academic tone.<br><br>2. Reviewer: "However, they are not readily available for testing on new data, have limited generalizability, and use methods that may yield overly optimistic performance estimates."<br>The authors should strive to support such statements with references to literature review papers.<br><br>Response: We agree that this statement should be supported by the existing methodological literature. We have therefore added references to work showing how non-independent data splits and flexible model development pipelines can yield overly optimistic performance estimates (Saeb et al., 2017), as well as work on pipeline overfitting and "trying a bunch of things" that specifically motivated our use of a preregistered lock-box evaluation (Hosseini et al., 2020).<br><br>To clarify what we meant by models being "not readily available," we have added a short explanation in the manuscript based on our experience attempting to perform

Powered by Editorial Manager® and ProduXion Manager® from Aries Systems Corporation

external replication. Several studies in this area state that code or data are available upon request. In practice, however, full access to model artifacts can still be difficult. For example, in the case of Gao et al., we contacted the authors multiple times over several months to request the trained model and curated dataset needed for external evaluation. These requests did not result in access, which prevented us from applying their model to our cohort. We fully recognize that there may be administrative, ethical, or logistical reasons that limit responses to such requests, and we do not intend this observation as a criticism of the authors. Our point is simply that, from the perspective of an independent research group, the absence of accessible model artifacts can make external validation challenging, even when studies describe their code or data as available upon request.

We hope this clarification explains why we noted that prior models are not always straightforwardly deployable for independent external testing.

3. Reviewer: "Additionally, while all adhered to standard ML methods (such as external validation sets), none employed a stricter lock-box set (i.e., held-out data points that were not used at any point during the hyperparameter optimization process, and publicly pre-registered), raising the possibility that results may be overly optimistic [30] due to iterative optimization of the analysis pipeline as a whole."
This statement is incorrect and should be significantly rephrased or removed. Although many related studies do not use held-out lock-box/external validation/test set and often are limited to results from leave-one-out or k-fold cross-validation, there exists studies (including studies referenced in the manuscript) that follow similar strict evaluation by testing on a dataset that is separate from development (i.e., training and validation) data (e.g., different set of infants) and that has been isolated from development (including hyperparameter optimization) and used for testing purposes and reporting only. In the current state of the manuscript, the authors seem to be unaware of or neglect this fact. To satisfy the reviewer, the authors need to carefully revise the manuscript to reflect the objective truth.

Response: Thank you for this important clarification. We agree that our original wording did not clearly distinguish between external validation and the more specific preregistered lock-box framework we intended to describe. We also agree that this ambiguity could be read as implying that earlier studies did not use fully independent test data. This was not our intention, and we apologize for the error.

As you note, several prior studies, including Gao et al. and others, evaluated their models on cohorts that were completely held out from training and hyperparameter optimization (the statement in our previous draft has been fully removed). These studies follow established best practices for rigorous model evaluation, and we have revised the manuscript to clearly acknowledge and credit this work.

The distinction we aim to draw is narrower and relates specifically to prospective preregistration. In our study, the evaluation dataset was specified in advance of any model development. The test set was therefore not only independent but also defined prospectively, with the preprocessing steps, model choices, and code frozen and publicly pre-registered before any evaluation. You are correct that it makes more sense to describe this as an extension of existing external validation practices rather than a contrast with them.

We revised the paragraph discussing prior work and our lock box design to more clearly acknowledge that several earlier studies used fully held out external test cohorts, and that in practice they may have not even looked at their test set more than once. The new text explicitly credits these studies for following standard machine learning evaluation practices and removes any phrasing that could be interpreted as suggesting otherwise.

We further clarified that our preregistered lock-box is intended as an extension of these practices. The revised paragraph now explains that the lock-box was prospectively specified, evaluated only once, and paired with a preregistered preprocessing and analysis plan. We also emphasized that this distinguishes our approach by adding preregistration and prospective specification, rather than by implying a lack of rigor in prior work.

Powered by Editorial Manager® and ProduXion Manager® from Aries Systems Corporation

Finally, we added language noting that preregistered, prospectively isolated test sets are still relatively uncommon in this literature and that we provide our implementation, including code and data, for others to validate the result and test on their own datasets.

4. Reviewer: Mention in the caption what T and H is referring to.
Response: Thank you for this helpful suggestion. We have added explicit definitions of pT−V and pT−T in the Table 1 caption, indicating comparisons between Train vs. Validation and Train vs. Test (lock-box) sets, respectively. This is in accordance with the new naming convention we have adopted to better align with published conventions.
5. Reviewer: Adhere to standard machine learning terminology and write validation instead of test set for the AutoML dataset throughout figure, figure caption and manuscript text. The lock-box set can alternatively be referred to as test set.

Response: Thank you for this suggestion; we agree that clear and standardized terminology is essential. In line with your recommendation, we have revised the figures, figure captions, and manuscript text to use Train / Validation / Test (lock-box) consistently. Specifically, we now refer to the AutoML evaluation dataset as the Validation set, and to the preregistered lock-box as the Test (lock-box) set. We will update the filenames on OSF, and our code, to reflect this.

In our implementation, Auto-sklearn performs internal validation through successive halving and cross-validation within the training data during model selection and hyperparameter optimization. On top of this, the Validation set is held out from training and used for performance estimation and model comparison during development.
By contrast, the Test (lock-box) set is completely isolated from the outset: its participant IDs, preprocessing pipeline, and analysis plan are preregistered in advance, and it is evaluated only once after all preprocessing decisions and modeling choices, including the final trained model, have been finalized and preregistered. This design adheres to standard ML terminology while adding a preregistered lock-box layer that, we believe, improves transparency.

6. Reviewer: "…ViTPose-H [19] performed better than the alternatives…"
Specify instead "…pretrained ViTPose-H [19] performed better than the fine-tuned alternatives..."

Response: Thank you for pointing out this potential point of confusion. We have revised the sentence to read:
“We found that the pretrained ViTPose-H performed better than other pretrained models (HRNet, PVTv2) and our previously fine-tuned OpenPose model.”
This revision clarifies that only OpenPose was fine-tuned on our prior infant dataset, while HRNet and PVTv2 were evaluated in their pretrained (COCO-trained) forms.

We also added a brief explanation that our methodological goal was to evaluate off-the-shelf models likely to generalize across datasets without requiring fine-tuning, in keeping with the reproducibility and scalability aims of the project. However, we acknowledge that fine-tuned infant-specific models are increasingly becoming available, and we plan to benchmark ViTPose-H against these newly released fine-tuned versions in future work. Should such models demonstrate equal or superior performance, they can be readily adopted within the same open-source pipeline we provide.

7. Reviewer: The three subsections "Benchmarking against deep learning-models", "Scaling analysis", and "Explainability and feature importance" describe features and predictive models and should be moved to an associated manuscript section(s) (i.e., they are misplaced in the section "Developing a pipeline for robust skeletal tracking").

Response: We have moved these subsections into Model Performance and Validation for better logical structure and consistency with the paper’s organization.

8. Figure 4 Reviewer: It would be more intuitive to have higher AUC with increased value of y-axis.

Powered by Editorial Manager® and ProduXion Manager® from Aries Systems Corporation

	Response: We absolutely agree with this point. The figure has been modified accordingly, with the y-axis orientation adjusted so that higher AUC values correspond to higher y values. This change makes the figure much easier to interpret at a glance and aligns the figure with standard conventions for performance curves.<br><br>9. Figure 7 Reviewer: "A decision threshold was selected to achieve a TPR of 80% on the holdout set."<br>This suggests that the threshold was set to optimize performance on the lock-box set and contradicts the authors' rationale that all decisions were made prior to testing on the lock-box set. Mention this as a limitation and also include performance on the lock-box set if the threshold instead was optimized on the training/validation data.<br><br>Response: Thank you for raising this important point. We agree that our original phrasing could be interpreted as selecting the decision threshold based on the lock box data, which would contradict the preregistered one-time evaluation design. We apologize for this ambiguity and have revised the manuscript to prevent this interpretation.<br>In our preregistered plan, we committed only to computing ROC curves on the lock box in order to characterize performance across thresholds, and we did not preregister the selection of any specific operating point. The confusion matrix shown in Figure 7 corresponds to a point on the lock box ROC curve that was requested by another reviewer, and is therefore presented only as an illustration of possible operating characteristics rather than as a preregistered threshold.<br><br>To address your concern, we now explicitly describe this figure as illustrative, and we have added a clear statement explaining that selecting a threshold after inspecting the lock box ROC curve can introduce optimistic bias. We have also added the performance obtained when the threshold is chosen using the training and validation data instead. This revision makes it explicit that threshold selection was not part of the preregistered plan and that the lock box was not used to optimize classification decisions. In future work we will aim to select a threshold prior to evaluation as an additional step towards generalizability.<br><br>We hope this resolves the concern and improves clarity regarding the preregistered evaluation design.<br><br>10. Reviewer: "However, under strict 'vanilla' settings, training time is restricted to 1 hour and the ensemble size restricted to one [41]. This constraint ensures a highly rigorous evaluation of individual models during the search phase. This approach increases the likelihood that the selected classifiers will generalize effectively to new, unseen data..."<br><br>This statement is counterintuitive as an ensemble of (several) machine learning models usually improves robustness attainable by individual models, by ensembles being less sensitive to noise that often negatively influences individual models.<br><br>Response: We agree and have rephrased to acknowledge that while ensembles can improve robustness, repeated validation-based adaptation in AutoML can induce overfitting. We clarify that single-model training was used to produce conservative results.<br><br>Overall:<br><br>Thank you again for your careful and thoughtful feedback. The suggested changes have strengthened the manuscript's accuracy, clarity, and consistency. We have implemented every requested modification, including the grammar and consistency errors, and hope the manuscript now fully addresses all remaining concerns.
**Additional Information:**	
**Question**	**Response**
Are you submitting this manuscript to a special series or article collection?	No

Powered by Editorial Manager® and ProduXion Manager® from Aries Systems Corporation

**Experimental design and statistics**<br><br>Full details of the experimental design and statistical methods used should be given in the Methods section, as detailed in our Minimum Standards Reporting Checklist. Information essential to interpreting the data presented should be made available in the figure legends.<br><br>Have you included all the information requested in your manuscript?	Yes
**Resources**<br><br>A description of all resources used, including antibodies, cell lines, animals and software tools, with enough information to allow them to be uniquely identified, should be included in the Methods section. Authors are strongly encouraged to cite Research Resource Identifiers (RRIDs) for antibodies, model organisms and tools, where possible.<br><br>Have you included the information requested as detailed in our Minimum Standards Reporting Checklist?	Yes
**Availability of data and materials**<br><br>All datasets and code on which the conclusions of the paper rely must be either included in your submission or deposited in publicly available repositories (where available and ethically appropriate), referencing such data using a unique identifier in the references and in the “Availability of Data and Materials” section of your manuscript.<br><br>Have you have met the above requirement as detailed in our Minimum Standards Reporting Checklist?	No

Powered by Editorial Manager® and ProduXion Manager® from Aries Systems Corporation

If not, please give reasons for any omissions below.<br><br>as follow-up to "**Availability of data and materials**<br><br>All datasets and code on which the conclusions of the paper rely must be either included in your submission or deposited in publicly available repositories (where available and ethically appropriate), referencing such data using a unique identifier in the references and in the "Availability of Data and Materials" section of your manuscript.<br><br>Have you have met the above requirement as detailed in our Minimum Standards Reporting Checklist?<br><br>"	Original dataset and extracted keypoints cannot be made openly available at this time to comply with ethics regulations.
GigaScience has policies and guidelines in place for the use of generative AI-writing tools such as ChatGPT. If you have used such writing tools to assist with writing the manuscript this must be declared and cited in the text. Authors should not list AI-writing tools and other AI-assisted technologies as an author or co-author and should acknowledge that they are fully responsible for text generated or refined by AI-writing tools.<p><br><br>A summary of use (particularly in the introduction or among methods) needs to be included at the end of the paper, and the outputs should also be included as a supplementary file hosted in GigaDB or other open repositories. Please read our guidelines for more information. <p><br><br>By submitting to GigaScience, you are aware of the journal's AI-writing tools policy, and if you have declared use of such tools below, you have acknowledged this where appropriate in your manuscript and have made a summary of use and outputs available. <p><br><b>AI-assisted writing tools have been used in the preparation of this manuscript?	No

Powered by Editorial Manager® and ProduXion Manager® from Aries Systems Corporation

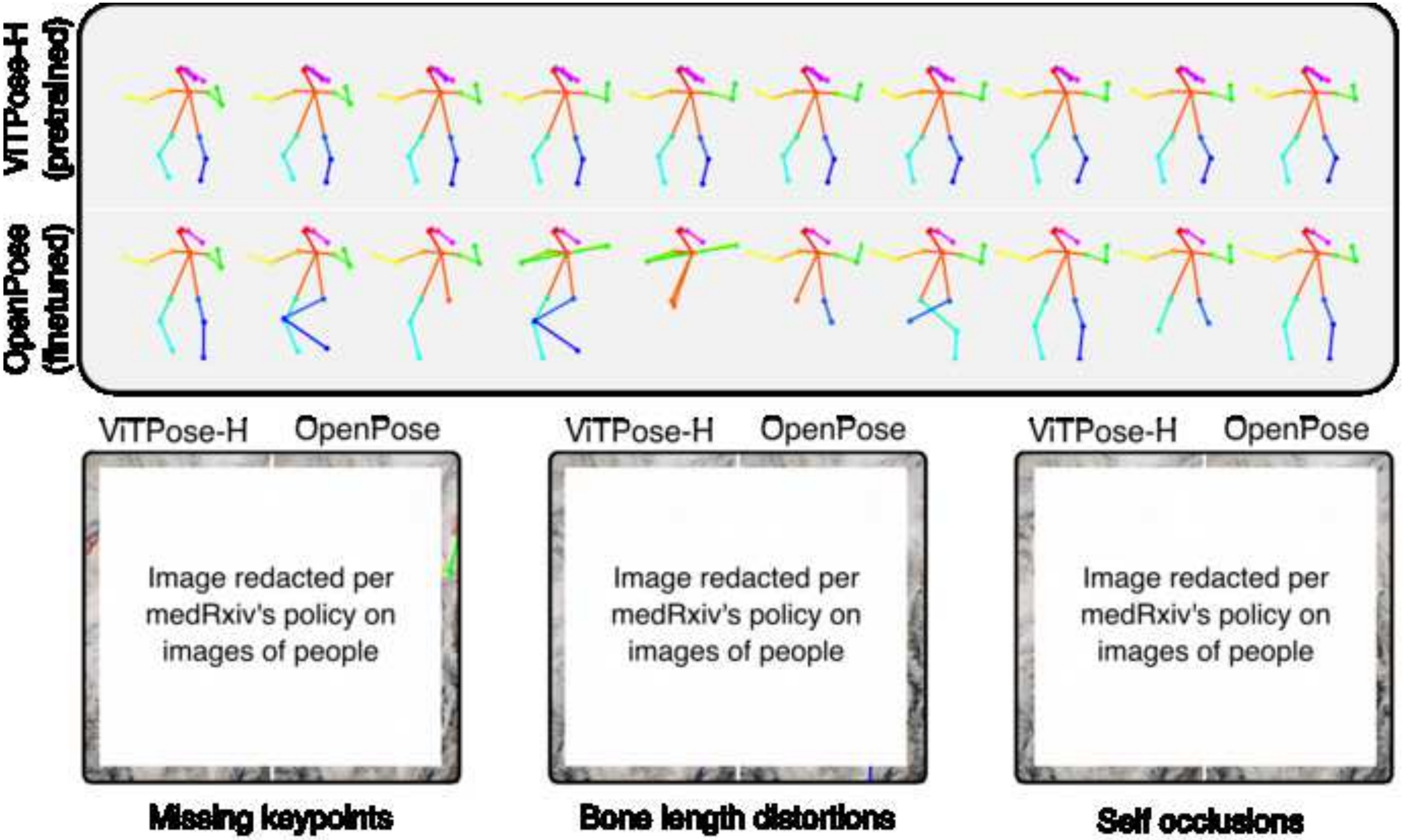
ViTPose-H (pretrained)
OpenPose (finetuned)
ViTPose-H
OpenPose
Image redacted per medRxiv's policy on images of people
Missing keypoints
Bone length distortions
Self occlusions

Manuscript Click here to access/download;Manuscript;main.tex

```
This is pdfTeX, Version 3.141592653-2.6-1.40.26 (TeX Live 2024)
(preloaded format=pdflatex 2024.8.2)  7 JAN 2026 00:52
entering extended mode
 restricted \write18 enabled.
 %&-line parsing enabled.
**main.tex
(./main.tex
LaTeX2e <2024-06-01> patch level 2
L3 programming layer <2024-05-27>
(./oup-contemporary.cls
Document Class: oup-contemporary 2023/06/12, v1.2
(c:/texlive/2024/texmf-dist/tex/latex/base/article.cls
Document Class: article 2024/02/08 v1.4n Standard LaTeX document class
(c:/texlive/2024/texmf-dist/tex/latex/base/size10.clo
File: size10.clo 2024/02/08 v1.4n Standard LaTeX file (size option)
)
\c@part=\count194
\c@section=\count195
\c@subsection=\count196
\c@subsubsection=\count197
\c@paragraph=\count198
\c@subparagraph=\count199
\c@figure=\count266
\c@table=\count267
\abovecaptionskip=\skip49
\belowcaptionskip=\skip50
\bibindent=\dimen141
) (c:/texlive/2024/texmf-dist/tex/latex/base/inputenc.sty
Package: inputenc 2024/02/08 v1.3d Input encoding file
\inpenc@prehook=\toks17
\inpenc@posthook=\toks18
) (c:/texlive/2024/texmf-dist/tex/latex/base/fontenc.sty
Package: fontenc 2021/04/29 v2.0v Standard LaTeX package
) (c:/texlive/2024/texmf-dist/tex/generic/iftex/ifpdf.sty
Package: ifpdf 2019/10/25 v3.4 ifpdf legacy package. Use iftex instead.
(c:/texlive/2024/texmf-dist/tex/generic/iftex/iftex.sty
Package: iftex 2022/02/03 v1.0f TeX engine tests
)) (c:/texlive/2024/texmf-dist/tex/latex/microtype/microtype.sty
Package: microtype 2024/03/29 v3.1b Micro-typographical refinements (RS)
(c:/texlive/2024/texmf-dist/tex/latex/graphics/keyval.sty
Package: keyval 2022/05/29 v1.15 key=value parser (DPC)
\KV@toks@=\toks19
) (c:/texlive/2024/texmf-dist/tex/latex/etoolbox/etoolbox.sty
Package: etoolbox 2020/10/05 v2.5k e-TeX tools for LaTeX (JAW)
\etb@tempcnta=\count268
)
\MT@toks=\toks20
\MT@tempbox=\box52
\MT@count=\count269
LaTeX Info: Redefining \noprotrusionifhmode on input line 1061.
LaTeX Info: Redefining \leftprotrusion on input line 1062.
\MT@prot@toks=\toks21
LaTeX Info: Redefining \rightprotrusion on input line 1081.
LaTeX Info: Redefining \textls on input line 1392.
```

```
\MT@outer@kern=\dimen142
LaTeX Info: Redefining \textmicrotypecontext on input line 2013.
\MT@listname@count=\count270
(c:/texlive/2024/texmf-dist/tex/latex/microtype/microtype-pdftex.def
File: microtype-pdftex.def 2024/03/29 v3.1b Definitions specific to
pdftex (RS)

LaTeX Info: Redefining \lsstyle on input line 902.
LaTeX Info: Redefining \lslig on input line 902.
\MT@outer@space=\skip51
)
Package microtype Info: Loading configuration file microtype.cfg.
(c:/texlive/2024/texmf-dist/tex/latex/microtype/microtype.cfg
File: microtype.cfg 2024/03/29 v3.1b microtype main configuration file
(RS)
)) (c:/texlive/2024/texmf-dist/tex/latex/euler/euler.sty
Package: euler 1995/03/05 v2.5
Package: `euler' v2.5 <1995/03/05> (FJ and FMi)
LaTeX Font Info:    Redeclaring symbol font `letters' on input line 35.
LaTeX Font Info:    Encoding `OML' has changed to `U' for symbol font
(Font)              `letters' in the math version `normal' on input line
35.
LaTeX Font Info:    Overwriting symbol font `letters' in version `normal'
(Font)                  OML/cmm/m/it --> U/eur/m/n on input line 35.
LaTeX Font Info:    Encoding `OML' has changed to `U' for symbol font
(Font)              `letters' in the math version `bold' on input line
35.
LaTeX Font Info:    Overwriting symbol font `letters' in version `bold'
(Font)                  OML/cmm/b/it --> U/eur/m/n on input line 35.
LaTeX Font Info:    Overwriting symbol font `letters' in version `bold'
(Font)                  U/eur/m/n --> U/eur/b/n on input line 36.
LaTeX Font Info:    Redeclaring math symbol \Gamma on input line 47.
LaTeX Font Info:    Redeclaring math symbol \Delta on input line 48.
LaTeX Font Info:    Redeclaring math symbol \Theta on input line 49.
LaTeX Font Info:    Redeclaring math symbol \Lambda on input line 50.
LaTeX Font Info:    Redeclaring math symbol \Xi on input line 51.
LaTeX Font Info:    Redeclaring math symbol \Pi on input line 52.
LaTeX Font Info:    Redeclaring math symbol \Sigma on input line 53.
LaTeX Font Info:    Redeclaring math symbol \Upsilon on input line 54.
LaTeX Font Info:    Redeclaring math symbol \Phi on input line 55.
LaTeX Font Info:    Redeclaring math symbol \Psi on input line 56.
LaTeX Font Info:    Redeclaring math symbol \Omega on input line 57.
\symEulerFraktur=\mathgroup4
LaTeX Font Info:    Overwriting symbol font `EulerFraktur' in version
`bold'
(Font)                  U/euf/m/n --> U/euf/b/n on input line 63.
LaTeX Info: Redefining \oldstylenums on input line 85.
\symEulerScript=\mathgroup5
LaTeX Font Info:    Overwriting symbol font `EulerScript' in version
`bold'
(Font)                  U/eus/m/n --> U/eus/b/n on input line 93.
LaTeX Font Info:    Redeclaring math symbol \aleph on input line 97.
LaTeX Font Info:    Redeclaring math symbol \Re on input line 98.
LaTeX Font Info:    Redeclaring math symbol \Im on input line 99.
```

```
LaTeX Font Info:    Redeclaring math delimiter \vert on input line 101.
LaTeX Font Info:    Redeclaring math delimiter \backslash on input line
103.
LaTeX Font Info:    Redeclaring math symbol \neg on input line 106.
LaTeX Font Info:    Redeclaring math symbol \wedge on input line 108.
LaTeX Font Info:    Redeclaring math symbol \vee on input line 110.
LaTeX Font Info:    Redeclaring math symbol \setminus on input line 112.
LaTeX Font Info:    Redeclaring math symbol \sim on input line 113.
LaTeX Font Info:    Redeclaring math symbol \mid on input line 114.
LaTeX Font Info:    Redeclaring math delimiter \arrowvert on input line
116.
LaTeX Font Info:    Redeclaring math symbol \mathsection on input line
117.
\symEulerExtension=\mathgroup6
LaTeX Font Info:    Redeclaring math symbol \coprod on input line 125.
LaTeX Font Info:    Redeclaring math symbol \prod on input line 125.
LaTeX Font Info:    Redeclaring math symbol \sum on input line 125.
LaTeX Font Info:    Redeclaring math symbol \intop on input line 130.
LaTeX Font Info:    Redeclaring math symbol \ointop on input line 131.
LaTeX Font Info:    Redeclaring math symbol \braceld on input line 132.
LaTeX Font Info:    Redeclaring math symbol \bracerd on input line 133.
LaTeX Font Info:    Redeclaring math symbol \bracelu on input line 134.
LaTeX Font Info:    Redeclaring math symbol \braceru on input line 135.
LaTeX Font Info:    Redeclaring math symbol \infty on input line 136.
LaTeX Font Info:    Redeclaring math symbol \nearrow on input line 153.
LaTeX Font Info:    Redeclaring math symbol \searrow on input line 154.
LaTeX Font Info:    Redeclaring math symbol \nwarrow on input line 155.
LaTeX Font Info:    Redeclaring math symbol \swarrow on input line 156.
LaTeX Font Info:    Redeclaring math symbol \Leftrightarrow on input line
157.
LaTeX Font Info:    Redeclaring math symbol \Leftarrow on input line 158.
LaTeX Font Info:    Redeclaring math symbol \Rightarrow on input line
159.
LaTeX Font Info:    Redeclaring math symbol \leftrightarrow on input line
160.
LaTeX Font Info:    Redeclaring math symbol \leftarrow on input line 161.
LaTeX Font Info:    Redeclaring math symbol \rightarrow on input line
163.
LaTeX Font Info:    Redeclaring math delimiter \uparrow on input line
166.
LaTeX Font Info:    Redeclaring math delimiter \downarrow on input line
168.
LaTeX Font Info:    Redeclaring math delimiter \updownarrow on input line
170.
LaTeX Font Info:    Redeclaring math delimiter \Uparrow on input line
172.
LaTeX Font Info:    Redeclaring math delimiter \Downarrow on input line
174.
LaTeX Font Info:    Redeclaring math delimiter \Updownarrow on input line
176.
LaTeX Font Info:    Redeclaring math symbol \leftharpoonup on input line
177.
LaTeX Font Info:    Redeclaring math symbol \leftharpoondown on input
line 178.
```

```
LaTeX Font Info:    Redeclaring math symbol \rightharpoonup on input line
179.
LaTeX Font Info:    Redeclaring math symbol \rightharpoondown on input
line 180
.
LaTeX Font Info:    Redeclaring math delimiter \lbrace on input line 182.
LaTeX Font Info:    Redeclaring math delimiter \rbrace on input line 184.
\symcmmigroup=\mathgroup7
LaTeX Font Info:    Overwriting symbol font `cmmigroup' in version `bold'
(Font)                  OML/cmm/m/it --> OML/cmm/b/it on input line 200.
LaTeX Font Info:    Redeclaring math accent \vec on input line 201.
LaTeX Font Info:    Redeclaring math symbol \triangleleft on input line
202.
LaTeX Font Info:    Redeclaring math symbol \triangleright on input line
203.
LaTeX Font Info:    Redeclaring math symbol \star on input line 204.
LaTeX Font Info:    Redeclaring math symbol \lhook on input line 205.
LaTeX Font Info:    Redeclaring math symbol \rhook on input line 206.
LaTeX Font Info:    Redeclaring math symbol \flat on input line 207.
LaTeX Font Info:    Redeclaring math symbol \natural on input line 208.
LaTeX Font Info:    Redeclaring math symbol \sharp on input line 209.
LaTeX Font Info:    Redeclaring math symbol \smile on input line 210.
LaTeX Font Info:    Redeclaring math symbol \frown on input line 211.
LaTeX Font Info:    Redeclaring math accent \grave on input line 245.
LaTeX Font Info:    Redeclaring math accent \acute on input line 246.
LaTeX Font Info:    Redeclaring math accent \tilde on input line 247.
LaTeX Font Info:    Redeclaring math accent \ddot on input line 248.
LaTeX Font Info:    Redeclaring math accent \check on input line 249.
LaTeX Font Info:    Redeclaring math accent \breve on input line 250.
LaTeX Font Info:    Redeclaring math accent \bar on input line 251.
LaTeX Font Info:    Redeclaring math accent \dot on input line 252.
LaTeX Font Info:    Redeclaring math accent \hat on input line 254.
) (c:/texlive/2024/texmf-dist/tex/latex/merriweather/merriweather.sty
Package: merriweather 2022/09/20 (Bob Tennent) Supports
Merriweather(Sans) font
s for all LaTeX engines.
(c:/texlive/2024/texmf-dist/tex/generic/iftex/ifxetex.sty
Package: ifxetex 2019/10/25 v0.7 ifxetex legacy package. Use iftex
instead.
) (c:/texlive/2024/texmf-dist/tex/generic/iftex/ifluatex.sty
Package: ifluatex 2019/10/25 v1.5 ifluatex legacy package. Use iftex
instead.
) (c:/texlive/2024/texmf-dist/tex/latex/base/textcomp.sty
Package: textcomp 2024/04/24 v2.1b Standard LaTeX package
) (c:/texlive/2024/texmf-dist/tex/latex/xkeyval/xkeyval.sty
Package: xkeyval 2022/06/16 v2.9 package option processing (HA)
(c:/texlive/2024/texmf-dist/tex/generic/xkeyval/xkeyval.tex
(c:/texlive/2024/te
xmf-dist/tex/generic/xkeyval/xkvutils.tex
\XKV@toks=\toks22
\XKV@tempa@toks=\toks23
)
\XKV@depth=\count271
```

```
File: xkeyval.tex 2014/12/03 v2.7a key=value parser (HA)
)) (c:/texlive/2024/texmf-dist/tex/latex/base/fontenc.sty
Package: fontenc 2021/04/29 v2.0v Standard LaTeX package
) (c:/texlive/2024/texmf-dist/tex/latex/fontaxes/fontaxes.sty
Package: fontaxes 2020/07/21 v1.0e Font selection axes
LaTeX Info: Redefining \upshape on input line 29.
LaTeX Info: Redefining \itshape on input line 31.
LaTeX Info: Redefining \slshape on input line 33.
LaTeX Info: Redefining \swshape on input line 35.
LaTeX Info: Redefining \scshape on input line 37.
LaTeX Info: Redefining \sscshape on input line 39.
LaTeX Info: Redefining \ulcshape on input line 41.
LaTeX Info: Redefining \textsw on input line 47.
LaTeX Info: Redefining \textssc on input line 48.
LaTeX Info: Redefining \textulc on input line 49.
)) (c:/texlive/2024/texmf-dist/tex/latex/mathastext/mathastext.sty
Package: mathastext 2024/07/27 v1.4b Use the text font in math mode (JFB)

Package mathastext Info: Starting the math mode configuration.
\mst@exists@muskip=\muskip17
\mst@forall@muskip=\muskip18
\mst@prime@muskip=\muskip19
\mst@do@nonletters=\toks24
\mst@undo@nonletters=\toks25
\mst@do@easynonletters=\toks26
\mst@undo@easynonletters=\toks27
\symmtoperatorfont=\mathgroup8
\symmtletterfont=\mathgroup9
(   mathastext:   ) ! and ?
(   mathastext:   ) punctuation: , . : ; and \colon
LaTeX Info: Redefining \relbar on input line 1201.
LaTeX Info: Redefining \rightarrowfill on input line 1202.
LaTeX Info: Redefining \leftarrowfill on input line 1205.
(   mathastext:   ) + and =
LaTeX Info: Redefining \Relbar on input line 1298.
(   mathastext:   ) adding = ; and + to \nfss@catcodes
(   mathastext:   ) parentheses ( ) [ ] and slash /
(   mathastext:   ) alldelims: < > \backslash \setminus | \vert \mid \{
\}
LaTeX Font Info:    Redeclaring math symbol \setminus on input line 1364.
LaTeX Info: Redefining \models on input line 1383.
(   mathastext:   ) \# \mathdollar \% \&
(   mathastext:   ) \imath and \jmath
LaTeX Font Info:    Overwriting math alphabet `\Mathnormalbold' in
version `nor
mal'
(Font)                  T1/Merriwthr-OsF/b/it --> T1/Merriwthr-OsF/b/it
on inpu
t line 2863.
LaTeX Font Info:    Overwriting math alphabet `\Mathnormalbold' in
version `bol
d'
(Font)                  T1/Merriwthr-OsF/b/it --> T1/Merriwthr-OsF/b/it
on inpu
```

```
t line 2863.
LaTeX Font Info:    Overwriting symbol font `mtletterfont' in version
`normal'
(Font)                  T1/Merriwthr-OsF/m/it --> T1/Merriwthr-OsF/m/it
on inpu
t line 2863.
LaTeX Font Info:    Overwriting symbol font `mtletterfont' in version
`bold'
(Font)                  T1/Merriwthr-OsF/m/it --> T1/Merriwthr-OsF/b/it
on inpu
t line 2863.
LaTeX Font Info:    Overwriting symbol font `mtoperatorfont' in version
`normal
'
(Font)                  T1/Merriwthr-OsF/m/n --> T1/Merriwthr-OsF/m/n on
input
line 2863.
LaTeX Font Info:    Overwriting symbol font `mtoperatorfont' in version
`bold'
(Font)                  T1/Merriwthr-OsF/m/n --> T1/Merriwthr-OsF/b/n on
input
line 2863.
LaTeX Font Info:    Overwriting math alphabet `\Mathbf' in version
`normal'
(Font)                  T1/Merriwthr-OsF/b/n --> T1/Merriwthr-OsF/b/n on
input
line 2863.
LaTeX Font Info:    Overwriting math alphabet `\Mathbf' in version `bold'
(Font)                  T1/Merriwthr-OsF/b/n --> T1/Merriwthr-OsF/b/n on
input
line 2863.
LaTeX Font Info:    Overwriting math alphabet `\Mathit' in version
`normal'
(Font)                  T1/Merriwthr-OsF/m/it --> T1/Merriwthr-OsF/m/it
on inpu
t line 2863.
LaTeX Font Info:    Overwriting math alphabet `\Mathit' in version `bold'
(Font)                  T1/Merriwthr-OsF/m/it --> T1/Merriwthr-OsF/b/it
on inpu
t line 2863.
LaTeX Font Info:    Overwriting math alphabet `\Mathsf' in version
`normal'
(Font)                  T1/MerriwthrSans-OsF/m/n --> T1/MerriwthrSans-
OsF/m/n o
n input line 2863.
LaTeX Font Info:    Overwriting math alphabet `\Mathsf' in version `bold'
(Font)                  T1/MerriwthrSans-OsF/m/n --> T1/MerriwthrSans-
OsF/b/n o
n input line 2863.
LaTeX Font Info:    Overwriting math alphabet `\Mathtt' in version
`normal'
(Font)                  T1/lmtt/m/n --> T1/lmtt/m/n on input line 2863.
LaTeX Font Info:    Overwriting math alphabet `\Mathtt' in version `bold'
(Font)                  T1/lmtt/m/n --> T1/lmtt/b/n on input line 2863.
```

```
(   mathastext:   ) Latin letters in the `normal', resp. `bold',
(   mathastext:   ) math versions are now set up to use the fonts
(   mathastext:   ) T1/Merriwthr-OsF/m/it, resp. T1/Merriwthr-OsF/b/it.
(   mathastext:   ) Other characters (digits, ...) and \log-like names
will be
(   mathastext:   ) typeset with the n shape.
(   mathastext:   ) \hbar
(   mathastext:   ) minus as endash
(   mathastext:   ) The italic option is in effect.
(   mathastext:   ) \HUGE has been (re)-defined.
(   mathastext:   ) mathastext has declared larger sizes for subscripts.
(   mathastext:   ) To keep LaTeX defaults, use option
`defaultmathsizes'.

Package mathastext Info: Loading is complete.  You can now use
\Mathastext to
(mathastext)             modify the normal and bold math versions.  Use
it
(mathastext)             with optional argument or use \MTDeclareVersion
to
(mathastext)             declare additional math versions.
) (c:/texlive/2024/texmf-dist/tex/latex/relsize/relsize.sty
Package: relsize 2013/03/29 ver 4.1
) (c:/texlive/2024/texmf-dist/tex/latex/ragged2e/ragged2e.sty
Package: ragged2e 2023/06/22 v3.6 ragged2e Package
\CenteringLeftskip=\skip52
\RaggedLeftLeftskip=\skip53
\RaggedRightLeftskip=\skip54
\CenteringRightskip=\skip55
\RaggedLeftRightskip=\skip56
\RaggedRightRightskip=\skip57
\CenteringParfillskip=\skip58
\RaggedLeftParfillskip=\skip59
\RaggedRightParfillskip=\skip60
\JustifyingParfillskip=\skip61
\CenteringParindent=\skip62
\RaggedLeftParindent=\skip63
\RaggedRightParindent=\skip64
\JustifyingParindent=\skip65
) (c:/texlive/2024/texmf-dist/tex/latex/xcolor/xcolor.sty
Package: xcolor 2023/11/15 v3.01 LaTeX color extensions (UK)
(c:/texlive/2024/texmf-dist/tex/latex/graphics-cfg/color.cfg
File: color.cfg 2016/01/02 v1.6 sample color configuration
)
Package xcolor Info: Driver file: pdftex.def on input line 274.
(c:/texlive/2024/texmf-dist/tex/latex/graphics-def/pdftex.def
File: pdftex.def 2024/04/13 v1.2c Graphics/color driver for pdftex
) (c:/texlive/2024/texmf-dist/tex/latex/graphics/mathcolor.ltx)
Package xcolor Info: Model `cmy' substituted by `cmy0' on input line
1350.
Package xcolor Info: Model `hsb' substituted by `rgb' on input line 1354.
Package xcolor Info: Model `RGB' extended on input line 1366.
Package xcolor Info: Model `HTML' substituted by `rgb' on input line
1368.
```

```
Package xcolor Info: Model `Hsb' substituted by `hsb' on input line 1369.
Package xcolor Info: Model `tHsb' substituted by `hsb' on input line
1370.
Package xcolor Info: Model `HSB' substituted by `hsb' on input line 1371.
Package xcolor Info: Model `Gray' substituted by `gray' on input line
1372.
Package xcolor Info: Model `wave' substituted by `hsb' on input line
1373.
) (c:/texlive/2024/texmf-dist/tex/latex/colortbl/colortbl.sty
Package: colortbl 2024/07/06 v1.0i Color table columns (DPC)
(c:/texlive/2024/texmf-dist/tex/latex/tools/array.sty
Package: array 2024/06/14 v2.6d Tabular extension package (FMi)
\col@sep=\dimen143
\ar@mcellbox=\box53
\extrarowheight=\dimen144
\NC@list=\toks28
\extratabsurround=\skip66
\backup@length=\skip67
\ar@cellbox=\box54
)
\everycr=\toks29
\minrowclearance=\skip68
\rownum=\count272
) (c:/texlive/2024/texmf-dist/tex/latex/graphics/graphicx.sty
Package: graphicx 2021/09/16 v1.2d Enhanced LaTeX Graphics (DPC,SPQR)
(c:/texlive/2024/texmf-dist/tex/latex/graphics/graphics.sty
Package: graphics 2024/05/23 v1.4g Standard LaTeX Graphics (DPC,SPQR)
(c:/texlive/2024/texmf-dist/tex/latex/graphics/trig.sty
Package: trig 2023/12/02 v1.11 sin cos tan (DPC)
) (c:/texlive/2024/texmf-dist/tex/latex/graphics-cfg/graphics.cfg
File: graphics.cfg 2016/06/04 v1.11 sample graphics configuration
)
Package graphics Info: Driver file: pdftex.def on input line 106.
)
\Gin@req@height=\dimen145
\Gin@req@width=\dimen146
) (c:/texlive/2024/texmf-dist/tex/latex/xpatch/xpatch.sty
(c:/texlive/2024/texm
f-dist/tex/latex/l3kernel/expl3.sty
Package: expl3 2024-05-27 L3 programming layer (loader)
(c:/texlive/2024/texmf-dist/tex/latex/l3backend/l3backend-pdftex.def
File: l3backend-pdftex.def 2024-05-08 L3 backend support: PDF output
(pdfTeX)
\l__color_backend_stack_int=\count273
\l__pdf_internal_box=\box55
))
Package: xpatch 2020/03/25 v0.3a Extending etoolbox patching commands
(c:/texlive/2024/texmf-dist/tex/latex/l3packages/xparse/xparse.sty
Package: xparse 2024-05-08 L3 Experimental document command parser
)) (c:/texlive/2024/texmf-dist/tex/latex/environ/environ.sty
Package: environ 2014/05/04 v0.3 A new way to define environments
(c:/texlive/2024/texmf-dist/tex/latex/trimspaces/trimspaces.sty
Package: trimspaces 2009/09/17 v1.1 Trim spaces around a token list
)
```

```
\@envbody=\toks30
) (c:/texlive/2024/texmf-dist/tex/latex/lastpage/lastpage.sty
Package: lastpage 2024/07/07 v2.1c lastpage: 2.09 or 2e? (HMM)
(c:/texlive/2024/texmf-dist/tex/latex/lastpage/lastpage2e.sty
Package: lastpage2e 2024/07/07 v2.1c Decide which 2e lastpage version to
use (H
MM)
(c:/texlive/2024/texmf-dist/tex/latex/lastpage/lastpagemodern.sty
Package: lastpagemodern 2024-07-07 v2.1c Refers to last page's name (HMM;
JPG)
\c@lastpagecount=\count274
)
)) (c:/texlive/2024/texmf-dist/tex/latex/graphics/rotating.sty
Package: rotating 2016/08/11 v2.16d rotated objects in LaTeX
(c:/texlive/2024/texmf-dist/tex/latex/base/ifthen.sty
Package: ifthen 2024/03/16 v1.1e Standard LaTeX ifthen package (DPC)
)
\c@r@tfl@t=\count275
\rotFPtop=\skip69
\rotFPbot=\skip70
\rot@float@box=\box56
\rot@mess@toks=\toks31
) (c:/texlive/2024/texmf-dist/tex/latex/graphics/lscape.sty
Package: lscape 2020/05/28 v3.02 Landscape Pages (DPC)
) (c:/texlive/2024/texmf-dist/tex/latex/tools/afterpage.sty
Package: afterpage 2023/07/04 v1.08 After-Page Package (DPC)
\AP@output=\toks32
\AP@partial=\box57
\AP@footins=\box58
) (c:/texlive/2024/texmf-dist/tex/latex/textpos/textpos.sty
Package: textpos 2022/07/23 v1.10.1
Package textpos Info: choosing support for LaTeX3 on input line 60.
\TP@textbox=\box59
\TP@holdbox=\box60
\TPHorizModule=\dimen147
\TPVertModule=\dimen148
\TP@margin=\dimen149
\TP@absmargin=\dimen150
Grid set 16 x 16 = 37.34424pt x 52.81541pt
\TPboxrulesize=\dimen151
\TP@ox=\dimen152
\TP@oy=\dimen153
\TP@tbargs=\toks33
TextBlockOrigin set to 0pt x 0pt
) (c:/texlive/2024/texmf-dist/tex/latex/url/url.sty
\Urlmuskip=\muskip20
Package: url 2013/09/16  ver 3.4  Verb mode for urls, etc.
) (c:/texlive/2024/texmf-dist/tex/latex/newfloat/newfloat.sty
Package: newfloat 2023/10/01 v1.2 Defining new floating environments (AR)
Package newfloat Info: `rotating' package detected.
) (c:/texlive/2024/texmf-dist/tex/latex/mdframed/mdframed.sty
Package: mdframed 2013/07/01 1.9b: mdframed
(c:/texlive/2024/texmf-dist/tex/latex/kvoptions/kvoptions.sty
```

```
Package: kvoptions 2022-06-15 v3.15 Key value format for package options
(HO)
(c:/texlive/2024/texmf-dist/tex/generic/ltxcmds/ltxcmds.sty
Package: ltxcmds 2023-12-04 v1.26 LaTeX kernel commands for general use
(HO)
) (c:/texlive/2024/texmf-dist/tex/latex/kvsetkeys/kvsetkeys.sty
Package: kvsetkeys 2022-10-05 v1.19 Key value parser (HO)
)) (c:/texlive/2024/texmf-dist/tex/latex/zref/zref-abspage.sty
Package: zref-abspage 2023-09-14 v2.35 Module abspage for zref (HO)
(c:/texlive/2024/texmf-dist/tex/latex/zref/zref-base.sty
Package: zref-base 2023-09-14 v2.35 Module base for zref (HO)
(c:/texlive/2024/texmf-dist/tex/generic/infwarerr/infwarerr.sty
Package: infwarerr 2019/12/03 v1.5 Providing info/warning/error messages
(HO)
) (c:/texlive/2024/texmf-dist/tex/generic/kvdefinekeys/kvdefinekeys.sty
Package: kvdefinekeys 2019-12-19 v1.6 Define keys (HO)
) (c:/texlive/2024/texmf-dist/tex/generic/pdftexcmds/pdftexcmds.sty
Package: pdftexcmds 2020-06-27 v0.33 Utility functions of pdfTeX for
LuaTeX (HO
)
Package pdftexcmds Info: \pdf@primitive is available.
Package pdftexcmds Info: \pdf@ifprimitive is available.
Package pdftexcmds Info: \pdfdraftmode found.
) (c:/texlive/2024/texmf-dist/tex/generic/etexcmds/etexcmds.sty
Package: etexcmds 2019/12/15 v1.7 Avoid name clashes with e-TeX commands
(HO)
) (c:/texlive/2024/texmf-dist/tex/latex/auxhook/auxhook.sty
Package: auxhook 2019-12-17 v1.6 Hooks for auxiliary files (HO)
)
Package zref Info: New property list: main on input line 767.
Package zref Info: New property: default on input line 768.
Package zref Info: New property: page on input line 769.
)
\c@abspage=\count276
Package zref Info: New property: abspage on input line 67.
) (c:/texlive/2024/texmf-dist/tex/latex/needspace/needspace.sty
Package: needspace 2010/09/12 v1.3d reserve vertical space
)
\mdf@templength=\skip71
\c@mdf@globalstyle@cnt=\count277
\mdf@skipabove@length=\skip72
\mdf@skipbelow@length=\skip73
\mdf@leftmargin@length=\skip74
\mdf@rightmargin@length=\skip75
\mdf@innerleftmargin@length=\skip76
\mdf@innerrightmargin@length=\skip77
\mdf@innertopmargin@length=\skip78
\mdf@innerbottommargin@length=\skip79
\mdf@splittopskip@length=\skip80
\mdf@splitbottomskip@length=\skip81
\mdf@outermargin@length=\skip82
\mdf@innermargin@length=\skip83
\mdf@linewidth@length=\skip84
\mdf@innerlinewidth@length=\skip85
```

```
\mdf@middlelinewidth@length=\skip86
\mdf@outerlinewidth@length=\skip87
\mdf@roundcorner@length=\skip88
\mdf@footenotedistance@length=\skip89
\mdf@userdefinedwidth@length=\skip90
\mdf@needspace@length=\skip91
\mdf@frametitleaboveskip@length=\skip92
\mdf@frametitlebelowskip@length=\skip93
\mdf@frametitlerulewidth@length=\skip94
\mdf@frametitleleftmargin@length=\skip95
\mdf@frametitlerightmargin@length=\skip96
\mdf@shadowsize@length=\skip97
\mdf@extratopheight@length=\skip98
\mdf@subtitleabovelinewidth@length=\skip99
\mdf@subtitlebelowlinewidth@length=\skip100
\mdf@subtitleaboveskip@length=\skip101
\mdf@subtitlebelowskip@length=\skip102
\mdf@subtitleinneraboveskip@length=\skip103
\mdf@subtitleinnerbelowskip@length=\skip104
\mdf@subsubtitleabovelinewidth@length=\skip105
\mdf@subsubtitlebelowlinewidth@length=\skip106
\mdf@subsubtitleaboveskip@length=\skip107
\mdf@subsubtitlebelowskip@length=\skip108
\mdf@subsubtitleinneraboveskip@length=\skip109
\mdf@subsubtitleinnerbelowskip@length=\skip110
(c:/texlive/2024/texmf-dist/tex/latex/mdframed/md-frame-0.mdf
File: md-frame-0.mdf 2013/07/01\ 1.9b: md-frame-0
)
\mdf@frametitlebox=\box61
\mdf@footnotebox=\box62
\mdf@splitbox@one=\box63
\mdf@splitbox@two=\box64
\mdf@splitbox@save=\box65
\mdfsplitboxwidth=\skip111
\mdfsplitboxtotalwidth=\skip112
\mdfsplitboxheight=\skip113
\mdfsplitboxdepth=\skip114
\mdfsplitboxtotalheight=\skip115
\mdfframetitleboxwidth=\skip116
\mdfframetitleboxtotalwidth=\skip117
\mdfframetitleboxheight=\skip118
\mdfframetitleboxdepth=\skip119
\mdfframetitleboxtotalheight=\skip120
\mdffootnoteboxwidth=\skip121
\mdffootnoteboxtotalwidth=\skip122
\mdffootnoteboxheight=\skip123
\mdffootnoteboxdepth=\skip124
\mdffootnoteboxtotalheight=\skip125
\mdftotallinewidth=\skip126
\mdfboundingboxwidth=\skip127
\mdfboundingboxtotalwidth=\skip128
\mdfboundingboxheight=\skip129
\mdfboundingboxdepth=\skip130
\mdfboundingboxtotalheight=\skip131
```

```
\mdf@freevspace@length=\skip132
\mdf@horizontalwidthofbox@length=\skip133
\mdf@verticalmarginwhole@length=\skip134
\mdf@horizontalspaceofbox=\skip135
\mdfsubtitleheight=\skip136
\mdfsubsubtitleheight=\skip137
\c@mdfcountframes=\count278

****** mdframed patching \endmdf@trivlist

****** -- success******

\mdf@envdepth=\count279
\c@mdf@env@i=\count280
\c@mdf@env@ii=\count281
\c@mdf@zref@counter=\count282
Package zref Info: New property: mdf@pagevalue on input line 895.
) (c:/texlive/2024/texmf-dist/tex/latex/titlesec/titlesec.sty
Package: titlesec 2023/10/27 v2.16 Sectioning titles
\ttl@box=\box66
\beforetitleunit=\skip138
\aftertitleunit=\skip139
\ttl@plus=\dimen154
\ttl@minus=\dimen155
\ttl@toksa=\toks34
\titlewidth=\dimen156
\titlewidthlast=\dimen157
\titlewidthfirst=\dimen158
) (c:/texlive/2024/texmf-dist/tex/latex/koma-script/scrextend.sty
Package: scrextend 2023/07/07 v3.41 KOMA-Script package (extend other
classes w
ith features of KOMA-Script classes)
(c:/texlive/2024/texmf-dist/tex/latex/koma-script/scrkbase.sty
Package: scrkbase 2023/07/07 v3.41 KOMA-Script package (KOMA-Script-
dependent b
asics and keyval usage)
(c:/texlive/2024/texmf-dist/tex/latex/koma-script/scrbase.sty
Package: scrbase 2023/07/07 v3.41 KOMA-Script package (KOMA-Script-
independent
basics and keyval usage)
(c:/texlive/2024/texmf-dist/tex/latex/koma-script/scrlfile.sty
Package: scrlfile 2023/07/07 v3.41 KOMA-Script package (file load hooks)
(c:/texlive/2024/texmf-dist/tex/latex/koma-script/scrlfile-hook.sty
Package: scrlfile-hook 2023/07/07 v3.41 KOMA-Script package (using LaTeX
hooks)

(c:/texlive/2024/texmf-dist/tex/latex/koma-script/scrlogo.sty
Package: scrlogo 2023/07/07 v3.41 KOMA-Script package (logo)
)))
Applying: [2021/05/01] Usage of raw or classic option list on input line
252.
Already applied: [0000/00/00] Usage of raw or classic option list on
input line
 368.
```

```
))
Package scrextend Info: unexpected definition of `\@makefnmark'.
(scrextend)             Trying to patch it on input line 1762.
Package scrextend Info: patch seems to be successfull on input line 1762.
)

LaTeX Font Warning: Font shape `T1/cmr/m/n' in size <7.5> not available
(Font)              size <7> substituted on input line 69.

(c:/texlive/2024/texmf-dist/tex/latex/tools/calc.sty
Package: calc 2023/07/08 v4.3 Infix arithmetic (KKT,FJ)
\calc@Acount=\count283
\calc@Bcount=\count284
\calc@Adimen=\dimen159
\calc@Bdimen=\dimen160
\calc@Askip=\skip140
\calc@Bskip=\skip141
LaTeX Info: Redefining \setlength on input line 80.
LaTeX Info: Redefining \addtolength on input line 81.
\calc@Ccount=\count285
\calc@Cskip=\skip142
) (c:/texlive/2024/texmf-dist/tex/latex/geometry/geometry.sty
Package: geometry 2020/01/02 v5.9 Page Geometry
(c:/texlive/2024/texmf-dist/tex/generic/iftex/ifvtex.sty
Package: ifvtex 2019/10/25 v1.7 ifvtex legacy package. Use iftex instead.
)
\Gm@cnth=\count286
\Gm@cntv=\count287
\c@Gm@tempcnt=\count288
\Gm@bindingoffset=\dimen161
\Gm@wd@mp=\dimen162
\Gm@odd@mp=\dimen163
\Gm@even@mp=\dimen164
\Gm@layoutwidth=\dimen165
\Gm@layoutheight=\dimen166
\Gm@layouthoffset=\dimen167
\Gm@layoutvoffset=\dimen168
\Gm@dimlist=\toks35
) (c:/texlive/2024/texmf-dist/tex/latex/preprint/authblk.sty
Package: authblk 2001/02/27 1.3 (PWD)
\affilsep=\skip143
\@affilsep=\skip144
\c@Maxaffil=\count289
\c@authors=\count290
\c@affil=\count291
) (c:/texlive/2024/texmf-dist/tex/latex/footmisc/footmisc.sty
Package: footmisc 2023/07/05 v6.0f a miscellany of footnote facilities
\FN@temptoken=\toks36
\footnotemargin=\dimen169
\@outputbox@depth=\dimen170
Package footmisc Info: Declaring symbol style bringhurst on input line
696.
Package footmisc Info: Declaring symbol style chicago on input line 704.
Package footmisc Info: Declaring symbol style wiley on input line 713.
```

```
Package footmisc Info: Declaring symbol style lamport-robust on input
line 724.

Package footmisc Info: Declaring symbol style lamport* on input line 744.
Package footmisc Info: Declaring symbol style lamport*-robust on input
line 765
.
) (c:/texlive/2024/texmf-dist/tex/latex/fancyhdr/fancyhdr.sty
Package: fancyhdr 2024/07/23 v4.3.1 Extensive control of page headers and
foote
rs
\f@nch@headwidth=\skip145
\f@nch@O@elh=\skip146
\f@nch@O@erh=\skip147
\f@nch@O@olh=\skip148
\f@nch@O@orh=\skip149
\f@nch@O@elf=\skip150
\f@nch@O@erf=\skip151
\f@nch@O@olf=\skip152
\f@nch@O@orf=\skip153
) (c:/texlive/2024/texmf-dist/tex/generic/alphalph/alphalph.sty
Package: alphalph 2019/12/09 v2.6 Convert numbers to letters (HO)
(c:/texlive/2024/texmf-dist/tex/generic/intcalc/intcalc.sty
Package: intcalc 2019/12/15 v1.3 Expandable calculations with integers
(HO)
))
\c@authorfn=\count292
(c:/texlive/2024/texmf-dist/tex/latex/abstract/abstract.sty
Package: abstract 2009/06/08 v1.2a configurable abstracts
\abstitleskip=\skip154
\absleftindent=\skip155
\absrightindent=\skip156
\absparindent=\skip157
\absparsep=\skip158
)
Package newfloat Info: New float `keypoints' with options
`placement=t!,name=kp
t' on input line 291.
\c@keypoints=\count293
\newfloat@ftype=\count294
Package newfloat Info: float type `keypoints'=8 on input line 291.
(c:/texlive/2024/texmf-dist/tex/latex/enumitem/enumitem.sty
Package: enumitem 2019/06/20 v3.9 Customized lists
\labelindent=\skip159
\enit@outerparindent=\dimen171
\enit@toks=\toks37
\enit@inbox=\box67
\enit@count@id=\count295
\enitdp@description=\count296
) (c:/texlive/2024/texmf-dist/tex/latex/quoting/quoting.sty
Package: quoting 2014/01/28 v0.1c Consolidated environment for displayed
text
\quo@toppartop=\skip160
) (c:/texlive/2024/texmf-dist/tex/latex/sttools/stfloats.sty
```

```
Package: stfloats 2017/03/27 v3.3 Improve float mechanism and
baselineskip sett
ings
\@dblbotnum=\count297
\c@dblbotnumber=\count298
) (c:/texlive/2024/texmf-dist/tex/latex/booktabs/booktabs.sty
Package: booktabs 2020/01/12 v1.61803398 Publication quality tables
\heavyrulewidth=\dimen172
\lightrulewidth=\dimen173
\cmidrulewidth=\dimen174
\belowrulesep=\dimen175
\belowbottomsep=\dimen176
\aboverulesep=\dimen177
\abovetopsep=\dimen178
\cmidrulesep=\dimen179
\cmidrulekern=\dimen180
\defaultaddspace=\dimen181
\@cmidla=\count299
\@cmidlb=\count300
\@aboverulesep=\dimen182
\@belowrulesep=\dimen183
\@thisruleclass=\count301
\@lastruleclass=\count302
\@thisrulewidth=\dimen184
) (c:/texlive/2024/texmf-dist/tex/latex/tools/tabularx.sty
Package: tabularx 2023/12/11 v2.12a `tabularx' package (DPC)
\TX@col@width=\dimen185
\TX@old@table=\dimen186
\TX@old@col=\dimen187
\TX@target=\dimen188
\TX@delta=\dimen189
\TX@cols=\count303
\TX@ftn=\toks38
)
\enitdp@tablenotes=\count304
(c:/texlive/2024/texmf-dist/tex/latex/caption/caption.sty
Package: caption 2023/08/05 v3.6o Customizing captions (AR)
(c:/texlive/2024/texmf-dist/tex/latex/caption/caption3.sty
Package: caption3 2023/07/31 v2.4d caption3 kernel (AR)
\caption@tempdima=\dimen190
\captionmargin=\dimen191
\caption@leftmargin=\dimen192
\caption@rightmargin=\dimen193
\caption@width=\dimen194
\caption@indent=\dimen195
\caption@parindent=\dimen196
\caption@hangindent=\dimen197
Package caption Info: Standard document class detected.
)
\c@caption@flags=\count305
\c@continuedfloat=\count306
Package caption Info: rotating package is loaded.
Package caption Info: scrextend package is loaded.
\caption@addmargin@hsize=\dimen198
```

```
\caption@addmargin@linewidth=\dimen199
) (c:/texlive/2024/texmf-dist/tex/latex/natbib/natbib.sty
Package: natbib 2010/09/13 8.31b (PWD, AO)
\bibhang=\skip161
\bibsep=\skip162
LaTeX Info: Redefining \cite on input line 694.
\c@NAT@ctr=\count307
)) (c:/texlive/2024/texmf-dist/tex/latex/siunitx/siunitx.sty
Package: siunitx 2024-06-24 v3.3.19 A comprehensive (SI) units package
\l__siunitx_number_uncert_offset_int=\count308
\l__siunitx_number_exponent_fixed_int=\count309
\l__siunitx_number_min_decimal_int=\count310
\l__siunitx_number_min_integer_int=\count311
\l__siunitx_number_round_precision_int=\count312
\l__siunitx_number_lower_threshold_int=\count313
\l__siunitx_number_upper_threshold_int=\count314
\l__siunitx_number_group_first_int=\count315
\l__siunitx_number_group_size_int=\count316
\l__siunitx_number_group_minimum_int=\count317
\l__siunitx_angle_tmp_dim=\dimen256
\l__siunitx_angle_marker_box=\box68
\l__siunitx_angle_unit_box=\box69
\l__siunitx_compound_count_int=\count318
(c:/texlive/2024/texmf-dist/tex/latex/translations/translations.sty
Package: translations 2022/02/05 v1.12 internationalization of LaTeX2e
packages
 (CN)
) (c:/texlive/2024/texmf-dist/tex/latex/amsmath/amstext.sty
Package: amstext 2021/08/26 v2.01 AMS text
(c:/texlive/2024/texmf-dist/tex/latex/amsmath/amsgen.sty
File: amsgen.sty 1999/11/30 v2.0 generic functions
\@emptytoks=\toks39
\ex@=\dimen257
))
\l__siunitx_table_tmp_box=\box70
\l__siunitx_table_tmp_dim=\dimen258
\l__siunitx_table_column_width_dim=\dimen259
\l__siunitx_table_integer_box=\box71
\l__siunitx_table_decimal_box=\box72
\l__siunitx_table_uncert_box=\box73
\l__siunitx_table_before_box=\box74
\l__siunitx_table_after_box=\box75
\l__siunitx_table_before_dim=\dimen260
\l__siunitx_table_carry_dim=\dimen261
\l__siunitx_unit_tmp_int=\count319
\l__siunitx_unit_position_int=\count320
\l__siunitx_unit_total_int=\count321
) (c:/texlive/2024/texmf-dist/tex/latex/tools/longtable.sty
Package: longtable 2024-04-26 v4.20 Multi-page Table package (DPC)
\LTleft=\skip163
\LTright=\skip164
\LTpre=\skip165
\LTpost=\skip166
\LTchunksize=\count322
```

```
\LTcapwidth=\dimen262
\LT@head=\box76
\LT@firsthead=\box77
\LT@foot=\box78
\LT@lastfoot=\box79
\LT@gbox=\box80
\LT@cols=\count323
\LT@rows=\count324
\c@LT@tables=\count325
\c@LT@chunks=\count326
\LT@p@ftn=\toks40
) (c:/texlive/2024/texmf-dist/tex/latex/amsmath/amsmath.sty
Package: amsmath 2024/05/23 v2.17q AMS math features
\@mathmargin=\skip167
For additional information on amsmath, use the `?' option.
(c:/texlive/2024/texmf-dist/tex/latex/amsmath/amsbsy.sty
Package: amsbsy 1999/11/29 v1.2d Bold Symbols
\pmbraise@=\dimen263
) (c:/texlive/2024/texmf-dist/tex/latex/amsmath/amsopn.sty
Package: amsopn 2022/04/08 v2.04 operator names
)
\inf@bad=\count327
LaTeX Info: Redefining \frac on input line 233.
\uproot@=\count328
\leftroot@=\count329
LaTeX Info: Redefining \overline on input line 398.
LaTeX Info: Redefining \colon on input line 409.
\classnum@=\count330
\DOTSCASE@=\count331
LaTeX Info: Redefining \ldots on input line 495.
LaTeX Info: Redefining \dots on input line 498.
LaTeX Info: Redefining \cdots on input line 619.
\Mathstrutbox@=\box81
\strutbox@=\box82
LaTeX Info: Redefining \big on input line 721.
LaTeX Info: Redefining \Big on input line 722.
LaTeX Info: Redefining \bigg on input line 723.
LaTeX Info: Redefining \Bigg on input line 724.
\big@size=\dimen264
LaTeX Font Info:    Redeclaring font encoding OML on input line 742.
LaTeX Font Info:    Redeclaring font encoding OMS on input line 743.
\macc@depth=\count332
LaTeX Info: Redefining \bmod on input line 904.
LaTeX Info: Redefining \pmod on input line 909.
LaTeX Info: Redefining \smash on input line 939.
LaTeX Info: Redefining \relbar on input line 969.
LaTeX Info: Redefining \Relbar on input line 970.
\c@MaxMatrixCols=\count333
\dotsspace@=\muskip21
\c@parentequation=\count334
\dspbrk@lvl=\count335
\tag@help=\toks41
\row@=\count336
\column@=\count337
```

```
\maxfields@=\count338
\andhelp@=\toks42
\eqnshift@=\dimen265
\alignsep@=\dimen266
\tagshift@=\dimen267
\tagwidth@=\dimen268
\totwidth@=\dimen269
\lineht@=\dimen270
\@envbody=\toks43
\multlinegap=\skip168
\multlinetaggap=\skip169
\mathdisplay@stack=\toks44
LaTeX Info: Redefining \[ on input line 2953.
LaTeX Info: Redefining \] on input line 2954.
) (c:/texlive/2024/texmf-dist/tex/latex/hyperref/nameref.sty
Package: nameref 2023-11-26 v2.56 Cross-referencing by name of section
(c:/texlive/2024/texmf-dist/tex/latex/refcount/refcount.sty
Package: refcount 2019/12/15 v3.6 Data extraction from label references
(HO)
) (c:/texlive/2024/texmf-
dist/tex/generic/gettitlestring/gettitlestring.sty
Package: gettitlestring 2019/12/15 v1.6 Cleanup title references (HO)
)
\c@section@level=\count339
) (c:/texlive/2024/texmf-dist/tex/latex/hyperref/hyperref.sty
Package: hyperref 2024-07-10 v7.01j Hypertext links for LaTeX
(c:/texlive/2024/texmf-dist/tex/generic/pdfescape/pdfescape.sty
Package: pdfescape 2019/12/09 v1.15 Implements pdfTeX's escape features
(HO)
) (c:/texlive/2024/texmf-dist/tex/latex/hycolor/hycolor.sty
Package: hycolor 2020-01-27 v1.10 Color options for hyperref/bookmark
(HO)
) (c:/texlive/2024/texmf-dist/tex/generic/stringenc/stringenc.sty
Package: stringenc 2019/11/29 v1.12 Convert strings between diff.
encodings (HO
)
)
\@linkdim=\dimen271
\Hy@linkcounter=\count340
\Hy@pagecounter=\count341
(c:/texlive/2024/texmf-dist/tex/latex/hyperref/pd1enc.def
File: pd1enc.def 2024-07-10 v7.01j Hyperref: PDFDocEncoding definition
(HO)
Now handling font encoding PD1 ...
... no UTF-8 mapping file for font encoding PD1
)
\Hy@SavedSpaceFactor=\count342
(c:/texlive/2024/texmf-dist/tex/latex/hyperref/puenc.def
File: puenc.def 2024-07-10 v7.01j Hyperref: PDF Unicode definition (HO)
Now handling font encoding PU ...
... no UTF-8 mapping file for font encoding PU
)
Package hyperref Info: Option `colorlinks' set `true' on input line 4040.
Package hyperref Info: Hyper figures OFF on input line 4157.
```

```
Package hyperref Info: Link nesting OFF on input line 4162.
Package hyperref Info: Hyper index ON on input line 4165.
Package hyperref Info: Plain pages OFF on input line 4172.
Package hyperref Info: Backreferencing OFF on input line 4177.
Package hyperref Info: Implicit mode ON; LaTeX internals redefined.
Package hyperref Info: Bookmarks ON on input line 4424.
\c@Hy@tempcnt=\count343
LaTeX Info: Redefining \url on input line 4763.
\XeTeXLinkMargin=\dimen272
(c:/texlive/2024/texmf-dist/tex/generic/bitset/bitset.sty
Package: bitset 2019/12/09 v1.3 Handle bit-vector datatype (HO)
(c:/texlive/2024/texmf-dist/tex/generic/bigintcalc/bigintcalc.sty
Package: bigintcalc 2019/12/15 v1.5 Expandable calculations on big
integers (HO
)
))
\Fld@menulength=\count344
\Field@Width=\dimen273
\Fld@charsize=\dimen274
Package hyperref Info: Hyper figures OFF on input line 6042.
Package hyperref Info: Link nesting OFF on input line 6047.
Package hyperref Info: Hyper index ON on input line 6050.
Package hyperref Info: backreferencing OFF on input line 6057.
Package hyperref Info: Link coloring ON on input line 6060.
Package hyperref Info: Link coloring with OCG OFF on input line 6067.
Package hyperref Info: PDF/A mode OFF on input line 6072.
(c:/texlive/2024/texmf-dist/tex/latex/base/atbegshi-ltx.sty
Package: atbegshi-ltx 2021/01/10 v1.0c Emulation of the original atbegshi
package with kernel methods
)
\Hy@abspage=\count345
\c@Item=\count346
\c@Hfootnote=\count347
)
Package hyperref Info: Driver (autodetected): hpdftex.
(c:/texlive/2024/texmf-dist/tex/latex/hyperref/hpdftex.def
File: hpdftex.def 2024-07-10 v7.01j Hyperref driver for pdfTeX
(c:/texlive/2024/texmf-dist/tex/latex/base/atveryend-ltx.sty
Package: atveryend-ltx 2020/08/19 v1.0a Emulation of the original
atveryend pac
kage
with kernel methods
)
\HyAnn@Count=\count348
\Fld@listcount=\count349
\c@bookmark@seq@number=\count350
(c:/texlive/2024/texmf-dist/tex/latex/rerunfilecheck/rerunfilecheck.sty
Package: rerunfilecheck 2022-07-10 v1.10 Rerun checks for auxiliary files
(HO)
(c:/texlive/2024/texmf-dist/tex/generic/uniquecounter/uniquecounter.sty
Package: uniquecounter 2019/12/15 v1.4 Provide unlimited unique counter
(HO)
)
```

```
Package uniquecounter Info: New unique counter `rerunfilecheck' on input
line 2
85.
)
\Hy@SectionHShift=\skip170
)
Package translations Info: No language package found. I am going to use
`englis
h' as default language. on input line 63.
LaTeX Font Info:    Trying to load font information for T1+Merriwthr-OsF
on inp
ut line 63.
(c:/texlive/2024/texmf-dist/tex/latex/merriweather/T1Merriwthr-OsF.fd
File: T1Merriwthr-OsF.fd 2020/08/30 (autoinst) Font definitions for
T1/Merriwth
r-OsF.
)
LaTeX Font Info:    Font shape `T1/Merriwthr-OsF/m/n' will be
(Font)              scaled to size 7.5pt on input line 63.
(./main.aux)
\openout1 = `main.aux'.

LaTeX Font Info:    Checking defaults for OML/cmm/m/it on input line 63.
LaTeX Font Info:    ... okay on input line 63.
LaTeX Font Info:    Checking defaults for OMS/cmsy/m/n on input line 63.
LaTeX Font Info:    ... okay on input line 63.
LaTeX Font Info:    Checking defaults for OT1/cmr/m/n on input line 63.
LaTeX Font Info:    ... okay on input line 63.
LaTeX Font Info:    Checking defaults for T1/cmr/m/n on input line 63.
LaTeX Font Info:    ... okay on input line 63.
LaTeX Font Info:    Checking defaults for TS1/cmr/m/n on input line 63.
LaTeX Font Info:    ... okay on input line 63.
LaTeX Font Info:    Checking defaults for OMX/cmex/m/n on input line 63.
LaTeX Font Info:    ... okay on input line 63.
LaTeX Font Info:    Checking defaults for U/cmr/m/n on input line 63.
LaTeX Font Info:    ... okay on input line 63.
LaTeX Font Info:    Checking defaults for PD1/pdf/m/n on input line 63.
LaTeX Font Info:    ... okay on input line 63.
LaTeX Font Info:    Checking defaults for PU/pdf/m/n on input line 63.
LaTeX Font Info:    ... okay on input line 63.
LaTeX Info: Redefining \microtypecontext on input line 63.
Package microtype Info: Applying patch `item' on input line 63.
Package microtype Info: Applying patch `toc' on input line 63.
Package microtype Info: Applying patch `eqnum' on input line 63.
Package microtype Info: Applying patch `footnote' on input line 63.
Package microtype Info: Applying patch `verbatim' on input line 63.
Package microtype Info: Generating PDF output.
Package microtype Info: Character protrusion enabled (level 2).
Package microtype Info: Using default protrusion set `alltext'.
Package microtype Info: Automatic font expansion enabled (level 2),
(microtype)             stretch: 20, shrink: 20, step: 1, non-selected.
Package microtype Info: Using default expansion set `alltext-nott'.
LaTeX Info: Redefining \showhyphens on input line 63.
Package microtype Info: No adjustment of tracking.
```

```
Package microtype Info: No adjustment of interword spacing.
Package microtype Info: No adjustment of character kerning.
Package microtype Info: Loading generic protrusion settings for font
family
(microtype)                `Merriwthr-OsF' (encoding: T1).
(microtype)                For optimal results, create family-specific
settings.
(microtype)                See the microtype manual for details.
LaTeX Font Info:    Redeclaring symbol font `operators' on input line 63.
LaTeX Font Info:    Encoding `OT1' has changed to `T1' for symbol font
(Font)              `operators' in the math version `normal' on input
line 63.
LaTeX Font Info:    Overwriting symbol font `operators' in version
`normal'
(Font)                  OT1/cmr/m/n --> T1/Merriwthr-OsF/m/up on input
line 63.

LaTeX Font Info:    Encoding `OT1' has changed to `T1' for symbol font
(Font)              `operators' in the math version `bold' on input line
63.
LaTeX Font Info:    Overwriting symbol font `operators' in version `bold'
(Font)                  OT1/cmr/bx/n --> T1/Merriwthr-OsF/m/up on input
line 63
.
LaTeX Font Info:    Overwriting symbol font `operators' in version `bold'
(Font)                  T1/Merriwthr-OsF/m/up --> T1/Merriwthr-OsF/b/up
on inpu
t line 63.
LaTeX Font Info:    Redeclaring math alphabet \mathbf on input line 63.
LaTeX Font Info:    Overwriting math alphabet `\mathbf' in version
`normal'
(Font)                  OT1/cmr/bx/n --> T1/Merriwthr-OsF/b/up on input
line 63
.
LaTeX Font Info:    Overwriting math alphabet `\mathbf' in version `bold'
(Font)                  OT1/cmr/bx/n --> T1/Merriwthr-OsF/b/up on input
line 63
.
LaTeX Font Info:    Redeclaring math alphabet \mathsf on input line 63.
LaTeX Font Info:    Overwriting math alphabet `\mathsf' in version
`normal'
(Font)                  OT1/cmss/m/n --> T1/MerriwthrSans-OsF/m/up on
input lin
e 63.
LaTeX Font Info:    Overwriting math alphabet `\mathsf' in version `bold'
(Font)                  OT1/cmss/bx/n --> T1/MerriwthrSans-OsF/m/up on
input li
ne 63.
LaTeX Font Info:    Redeclaring math alphabet \mathit on input line 63.
LaTeX Font Info:    Overwriting math alphabet `\mathit' in version
`normal'
(Font)                  OT1/cmr/m/it --> T1/Merriwthr-OsF/m/it on input
line 63
.
```

```
LaTeX Font Info:    Overwriting math alphabet `\mathit' in version `bold'
(Font)                  OT1/cmr/bx/it --> T1/Merriwthr-OsF/m/it on input
line 6
3.
LaTeX Font Info:    Redeclaring math alphabet \mathtt on input line 63.
LaTeX Font Info:    Overwriting math alphabet `\mathtt' in version
`normal'
(Font)                  OT1/cmtt/m/n --> T1/lmtt/m/up on input line 63.
LaTeX Font Info:    Overwriting math alphabet `\mathtt' in version `bold'
(Font)                  OT1/cmtt/m/n --> T1/lmtt/m/up on input line 63.
LaTeX Font Info:    Overwriting math alphabet `\mathsf' in version `bold'
(Font)                  T1/MerriwthrSans-OsF/m/up --> T1/MerriwthrSans-
OsF/b/up
 on input line 63.
LaTeX Font Info:    Overwriting math alphabet `\mathit' in version `bold'
(Font)                  T1/Merriwthr-OsF/m/it --> T1/Merriwthr-OsF/b/it
on inpu
t line 63.
\c@mv@tabular=\count351
\c@mv@boldtabular=\count352
(c:/texlive/2024/texmf-dist/tex/context/base/mkii/supp-pdf.mkii
[Loading MPS to PDF converter (version 2006.09.02).]
\scratchcounter=\count353
\scratchdimen=\dimen275
\scratchbox=\box83
\nofMPsegments=\count354
\nofMParguments=\count355
\everyMPshowfont=\toks45
\MPscratchCnt=\count356
\MPscratchDim=\dimen276
\MPnumerator=\count357
\makeMPintoPDFobject=\count358
\everyMPtoPDFconversion=\toks46
) (c:/texlive/2024/texmf-dist/tex/latex/epstopdf-pkg/epstopdf-base.sty
Package: epstopdf-base 2020-01-24 v2.11 Base part for package epstopdf
Package epstopdf-base Info: Redefining graphics rule for `.eps' on input
line 4
85.
(c:/texlive/2024/texmf-dist/tex/latex/latexconfig/epstopdf-sys.cfg
File: epstopdf-sys.cfg 2010/07/13 v1.3 Configuration of (r)epstopdf for
TeX Liv
e
))
*geometry* driver: auto-detecting
*geometry* detected driver: pdftex
*geometry* verbose mode - [ preamble ] result:
* driver: pdftex
* paper: a4paper
* layout: <same size as paper>
* layoutoffset:(h,v)=(0.0pt,0.0pt)
* modes: includefoot twoside
* h-part:(L,W,R)=(54.64pt, 488.22787pt, 54.64pt)
* v-part:(T,H,B)=(66.0pt, 745.04684pt, 34.0pt)
* \paperwidth=597.50787pt
```

```
* \paperheight=845.04684pt
* \textwidth=488.22787pt
* \textheight=715.04684pt
* \oddsidemargin=-17.62999pt
* \evensidemargin=-17.62999pt
* \topmargin=-47.76999pt
* \headheight=17.5pt
* \headsep=24.0pt
* \topskip=10.0pt
* \footskip=30.0pt
* \marginparwidth=48.0pt
* \marginparsep=10.0pt
* \columnsep=18.0pt
* \skip\footins=22.0pt plus 2.0pt
* \hoffset=0.0pt
* \voffset=0.0pt
* \mag=1000
* \@twocolumntrue
* \@twosidetrue
* \@mparswitchtrue
* \@reversemarginfalse
* (1in=72.27pt=25.4mm, 1cm=28.453pt)

Package caption Info: Begin \AtBeginDocument code.
Package caption Info: hyperref package is loaded.
Package caption Info: longtable package is loaded.
(c:/texlive/2024/texmf-dist/tex/latex/caption/ltcaption.sty
Package: ltcaption 2021/01/08 v1.4c longtable captions (AR)
)
Package caption Info: End \AtBeginDocument code.

(c:/texlive/2024/texmf-dist/tex/latex/translations/translations-basic-
dictionar
y-english.trsl
File: translations-basic-dictionary-english.trsl (english translation
file `tra
nslations-basic-dictionary')
)
Package translations Info: loading dictionary `translations-basic-
dictionary' f
or `english'. on input line 63.
Package hyperref Info: Link coloring ON on input line 63.
(./main.out) (./main.out)
\@outlinefile=\write3
\openout3 = `main.out'.

\@gscitedetails=\box84
\@gscitedetailsheight=\skip171
\@gsheadbox=\box85
\@gsheadboxheight=\skip172
LaTeX Font Info:    Font shape `T1/Merriwthr-OsF/b/n' will be
(Font)              scaled to size 6.5pt on input line 63.
LaTeX Font Info:    Calculating math sizes for size <7.5> on input line
63.
```

```
LaTeX Font Warning: Font shape `T1/Merriwthr-OsF/m/up' undefined
(Font)              using `T1/Merriwthr-OsF/m/n' instead on input line
63.

LaTeX Font Info:    Font shape `T1/Merriwthr-OsF/m/up' will be
(Font)              scaled to size 6.24973pt on input line 63.
LaTeX Font Info:    Font shape `T1/Merriwthr-OsF/m/up' will be
(Font)              scaled to size 5.24997pt on input line 63.
LaTeX Font Info:    Trying to load font information for U+eur on input
line 63.

(c:/texlive/2024/texmf-dist/tex/latex/amsfonts/ueur.fd
File: ueur.fd 2013/01/14 v3.01 Euler Roman
) (c:/texlive/2024/texmf-dist/tex/latex/microtype/mt-eur.cfg
File: mt-eur.cfg 2006/07/31 v1.1 microtype config. file: AMS Euler Roman
(RS)
)

LaTeX Font Warning: Font shape `OMS/cmsy/m/n' in size <7.5> not available
(Font)              size <7> substituted on input line 63.

LaTeX Font Info:    Trying to load font information for U+euf on input
line 63.

(c:/texlive/2024/texmf-dist/tex/latex/amsfonts/ueuf.fd
File: ueuf.fd 2013/01/14 v3.01 Euler Fraktur
) (c:/texlive/2024/texmf-dist/tex/latex/microtype/mt-euf.cfg
File: mt-euf.cfg 2006/07/03 v1.1 microtype config. file: AMS Euler
Fraktur (RS)

)
LaTeX Font Info:    Trying to load font information for U+eus on input
line 63.

(c:/texlive/2024/texmf-dist/tex/latex/amsfonts/ueus.fd
File: ueus.fd 2013/01/14 v3.01 Euler Script
) (c:/texlive/2024/texmf-dist/tex/latex/microtype/mt-eus.cfg
File: mt-eus.cfg 2006/07/28 v1.2 microtype config. file: AMS Euler Script
(RS)
)
LaTeX Font Info:    Trying to load font information for U+euex on input
line 63
.
(c:/texlive/2024/texmf-dist/tex/latex/amsfonts/ueuex.fd
File: ueuex.fd 2013/01/14 v3.01 Euler extra symbols
)

LaTeX Font Warning: Font shape `OML/cmm/m/it' in size <7.5> not available
(Font)              size <7> substituted on input line 63.

LaTeX Font Info:    Font shape `T1/Merriwthr-OsF/m/n' will be
(Font)              scaled to size 6.24973pt on input line 63.
LaTeX Font Info:    Font shape `T1/Merriwthr-OsF/m/n' will be
```

```
(Font)              scaled to size 5.24997pt on input line 63.
LaTeX Font Info:    Font shape `T1/Merriwthr-OsF/m/it' will be
(Font)              scaled to size 7.5pt on input line 63.
LaTeX Font Info:    Font shape `T1/Merriwthr-OsF/m/it' will be
(Font)              scaled to size 6.24973pt on input line 63.
LaTeX Font Info:    Font shape `T1/Merriwthr-OsF/m/it' will be
(Font)              scaled to size 5.24997pt on input line 63.
LaTeX Font Info:    Font shape `T1/Merriwthr-OsF/m/n' will be
(Font)              scaled to size 8.0pt on input line 63.
LaTeX Font Info:    Font shape `T1/Merriwthr-OsF/m/it' will be
(Font)              scaled to size 8.0pt on input line 63.
LaTeX Font Info:    Font shape `T1/Merriwthr-OsF/b/it' will be
(Font)              scaled to size 8.0pt on input line 63.
TextBlockOrigin set to 4pc+6.64pt x 4pc+6pt
<oup.pdf, id=169, 597.50829pt x 845.0471pt>
File: oup.pdf Graphic file (type pdf)
<use oup.pdf>
Package pdftex.def Info: oup.pdf  used on input line 78.
(pdftex.def)             Requested size: 41.03665pt x 58.038pt.

Overfull \hbox (54.64pt too wide) in paragraph at lines 78--78
[][]
 []

LaTeX Font Info:    Font shape `T1/Merriwthr-OsF/m/n' will be
(Font)              scaled to size 14.0pt on input line 78.
LaTeX Font Info:    Font shape `T1/Merriwthr-OsF/m/n' will be
(Font)              scaled to size 8.99997pt on input line 78.
LaTeX Font Info:    Calculating math sizes for size <14> on input line
78.
LaTeX Font Info:    Font shape `T1/Merriwthr-OsF/m/up' will be
(Font)              scaled to size 14.0pt on input line 78.
LaTeX Font Info:    Font shape `T1/Merriwthr-OsF/m/up' will be
(Font)              scaled to size 11.66617pt on input line 78.
LaTeX Font Info:    Font shape `T1/Merriwthr-OsF/m/up' will be
(Font)              scaled to size 9.79996pt on input line 78.
LaTeX Font Info:    Font shape `T1/Merriwthr-OsF/m/n' will be
(Font)              scaled to size 11.66617pt on input line 78.
LaTeX Font Info:    Font shape `T1/Merriwthr-OsF/m/n' will be
(Font)              scaled to size 9.79996pt on input line 78.
LaTeX Font Info:    Font shape `T1/Merriwthr-OsF/m/it' will be
(Font)              scaled to size 14.0pt on input line 78.
LaTeX Font Info:    Font shape `T1/Merriwthr-OsF/m/it' will be
(Font)              scaled to size 11.66617pt on input line 78.
LaTeX Font Info:    Font shape `T1/Merriwthr-OsF/m/it' will be
(Font)              scaled to size 9.79996pt on input line 78.
LaTeX Font Info:    Font shape `T1/Merriwthr-OsF/b/n' will be
(Font)              scaled to size 18.0pt on input line 78.
LaTeX Font Info:    Font shape `T1/Merriwthr-OsF/m/n' will be
(Font)              scaled to size 13.0pt on input line 78.
LaTeX Font Info:    Calculating math sizes for size <13> on input line
78.
LaTeX Font Info:    Font shape `T1/Merriwthr-OsF/m/up' will be
(Font)              scaled to size 13.0pt on input line 78.
```

```
LaTeX Font Info:    Font shape `T1/Merriwthr-OsF/m/up' will be
(Font)              scaled to size 10.83287pt on input line 78.
LaTeX Font Info:    Font shape `T1/Merriwthr-OsF/m/up' will be
(Font)              scaled to size 9.09996pt on input line 78.

LaTeX Font Warning: Font shape `OMS/cmsy/m/n' in size <13> not available
(Font)              size <12> substituted on input line 78.


LaTeX Font Warning: Font shape `OMX/cmex/m/n' in size <13> not available
(Font)              size <12> substituted on input line 78.


LaTeX Font Warning: Font shape `OML/cmm/m/it' in size <13> not available
(Font)              size <12> substituted on input line 78.

LaTeX Font Info:    Font shape `T1/Merriwthr-OsF/m/n' will be
(Font)              scaled to size 10.83287pt on input line 78.
LaTeX Font Info:    Font shape `T1/Merriwthr-OsF/m/n' will be
(Font)              scaled to size 9.09996pt on input line 78.
LaTeX Font Info:    Font shape `T1/Merriwthr-OsF/m/it' will be
(Font)              scaled to size 13.0pt on input line 78.
LaTeX Font Info:    Font shape `T1/Merriwthr-OsF/m/it' will be
(Font)              scaled to size 10.83287pt on input line 78.
LaTeX Font Info:    Font shape `T1/Merriwthr-OsF/m/it' will be
(Font)              scaled to size 9.09996pt on input line 78.
LaTeX Font Info:    Trying to load font information for TS1+Merriwthr-OsF
on in
put line 78.
(c:/texlive/2024/texmf-dist/tex/latex/merriweather/TS1Merriwthr-OsF.fd
File: TS1Merriwthr-OsF.fd 2020/08/30 (autoinst) Font definitions for
TS1/Merriw
thr-OsF.
)
LaTeX Font Info:    Font shape `TS1/Merriwthr-OsF/m/n' will be
(Font)              scaled to size 10.83287pt on input line 78.
Package microtype Info: Loading generic protrusion settings for font
family
(microtype)             `Merriwthr-OsF' (encoding: TS1).
(microtype)             For optimal results, create family-specific
settings.
(microtype)             See the microtype manual for details.
LaTeX Font Info:    Font shape `T1/Merriwthr-OsF/m/n' will be
(Font)              scaled to size 9.0pt on input line 78.
LaTeX Font Info:    Font shape `T1/Merriwthr-OsF/m/up' will be
(Font)              scaled to size 9.0pt on input line 78.
LaTeX Font Info:    Font shape `T1/Merriwthr-OsF/m/up' will be
(Font)              scaled to size 7.0pt on input line 78.
LaTeX Font Info:    Font shape `T1/Merriwthr-OsF/m/up' will be
(Font)              scaled to size 5.0pt on input line 78.
LaTeX Font Info:    Font shape `T1/Merriwthr-OsF/m/n' will be
(Font)              scaled to size 7.0pt on input line 78.
LaTeX Font Info:    Font shape `T1/Merriwthr-OsF/m/n' will be
(Font)              scaled to size 5.0pt on input line 78.
```

```
LaTeX Font Info:    Font shape `T1/Merriwthr-OsF/m/it' will be
(Font)              scaled to size 9.0pt on input line 78.
LaTeX Font Info:    Font shape `T1/Merriwthr-OsF/m/it' will be
(Font)              scaled to size 7.0pt on input line 78.
LaTeX Font Info:    Font shape `T1/Merriwthr-OsF/m/it' will be
(Font)              scaled to size 5.0pt on input line 78.
LaTeX Font Info:    Font shape `T1/Merriwthr-OsF/m/n' will be
(Font)              scaled to size 6.5pt on input line 78.
LaTeX Font Info:    Calculating math sizes for size <6.5> on input line
78.
LaTeX Font Info:    Font shape `T1/Merriwthr-OsF/m/up' will be
(Font)              scaled to size 6.5pt on input line 78.
LaTeX Font Info:    Font shape `T1/Merriwthr-OsF/m/up' will be
(Font)              scaled to size 5.41643pt on input line 78.
LaTeX Font Info:    Font shape `T1/Merriwthr-OsF/m/up' will be
(Font)              scaled to size 4.54997pt on input line 78.

LaTeX Font Warning: Font shape `OMS/cmsy/m/n' in size <6.5> not available
(Font)              size <6> substituted on input line 78.


LaTeX Font Warning: Font shape `OMS/cmsy/m/n' in size <5.41643> not
available
(Font)              size <5> substituted on input line 78.


LaTeX Font Warning: Font shape `OMS/cmsy/m/n' in size <4.54997> not
available
(Font)              size <5> substituted on input line 78.


LaTeX Font Warning: Font shape `OML/cmm/m/it' in size <6.5> not available
(Font)              size <6> substituted on input line 78.


LaTeX Font Warning: Font shape `OML/cmm/m/it' in size <5.41643> not
available
(Font)              size <5> substituted on input line 78.


LaTeX Font Warning: Font shape `OML/cmm/m/it' in size <4.54997> not
available
(Font)              size <5> substituted on input line 78.

LaTeX Font Info:    Font shape `T1/Merriwthr-OsF/m/n' will be
(Font)              scaled to size 5.41643pt on input line 78.
LaTeX Font Info:    Font shape `T1/Merriwthr-OsF/m/n' will be
(Font)              scaled to size 4.54997pt on input line 78.
LaTeX Font Info:    Font shape `T1/Merriwthr-OsF/m/it' will be
(Font)              scaled to size 6.5pt on input line 78.
LaTeX Font Info:    Font shape `T1/Merriwthr-OsF/m/it' will be
(Font)              scaled to size 5.41643pt on input line 78.
LaTeX Font Info:    Font shape `T1/Merriwthr-OsF/m/it' will be
(Font)              scaled to size 4.54997pt on input line 78.
```

```
LaTeX Font Info:    Font shape `TS1/Merriwthr-OsF/m/n' will be
(Font)              scaled to size 5.41643pt on input line 78.

Overfull \hbox (54.64pt too wide) in paragraph at lines 78--78
[][][]
 []

LaTeX Font Info:    Font shape `T1/Merriwthr-OsF/b/n' will be
(Font)              scaled to size 10.0pt on input line 78.
LaTeX Font Info:    Font shape `T1/Merriwthr-OsF/b/n' will be
(Font)              scaled to size 8.0pt on input line 78.

Overfull \hbox (54.64pt too wide) in paragraph at lines 78--78
[][][]
 []


LaTeX Warning: Text page 1 contains only floats.


Underfull \vbox (badness 10000) has occurred while \output is active []




LaTeX Warning: Text page 1 contains only floats.


Underfull \vbox (badness 10000) has occurred while \output is active []



LaTeX Font Info:    Font shape `T1/Merriwthr-OsF/m/n' will be
(Font)              scaled to size 7.8pt on input line 78.
LaTeX Font Info:    Font shape `T1/Merriwthr-OsF/b/n' will be
(Font)              scaled to size 7.8pt on input line 78.
[1{c:/texlive/2024/texmf-
var/fonts/map/pdftex/updmap/pdftex.map}{c:/texlive/202
4/texmf-
dist/fonts/enc/dvips/merriweather/merriwthr_posqbl.enc}{c:/texlive/2024
/texmf-dist/fonts/enc/dvips/merriweather/merriwthr_owzwzj.enc}


 <./oup.pdf>]
Package mdframed Info: mdframed works in twoside mode on input line 82.
LaTeX Font Info:    Font shape `T1/Merriwthr-OsF/b/n' will be
(Font)              scaled to size 8.2pt on input line 82.
LaTeX Font Info:    Font shape `TS1/Merriwthr-OsF/m/n' will be
(Font)              scaled to size 7.5pt on input line 84.
Package mdframed Info: mdframed inside float
 mdframed uses option nobreak mdframed on input line 90.
Package mdframed Info: mdframed inside a box
 mdframed uses option nobreak mdframed on input line 90.
LaTeX Font Info:    Font shape `T1/Merriwthr-OsF/b/n' will be
```

```
(Font)              scaled to size 7.5pt on input line 100.

Package natbib Warning: Citation `huang2023posture' on page 2 undefined on inpu
t line 100.




Underfull \hbox (badness 2150) in paragraph at lines 102--105
[]\T1/Merriwthr-OsF/m/n/7.5 (+20) Most video-based au-to-mated risk as-sess-men
t mod-els are
 []


Underfull \vbox (badness 3471) has occurred while \output is active []



LaTeX Font Info:    Font shape `T1/Merriwthr-OsF/m/it' will be
(Font)              scaled to size 7.8pt on input line 107.
[2

]
<Figure1.png, id=307, 542.2659pt x 147.9126pt>
File: Figure1.png Graphic file (type png)
<use Figure1.png>
Package pdftex.def Info: Figure1.png  used on input line 116.
(pdftex.def)             Requested size: 341.75801pt x 93.22153pt.
LaTeX Font Info:    Font shape `T1/Merriwthr-OsF/m/n' will be
(Font)              scaled to size 6.0pt on input line 118.
LaTeX Font Info:    Font shape `T1/Merriwthr-OsF/b/n' will be
(Font)              scaled to size 6.0pt on input line 118.
LaTeX Font Info:    Font shape `T1/Merriwthr-OsF/b/n' will be
(Font)              scaled to size 8.5pt on input line 123.



! LaTeX Error: Unicode character − (U+2212)
               not set up for use with LaTeX.

See the LaTeX manual or LaTeX Companion for explanation.
Type  H <return>  for immediate help.
 ...

l.132 ...or FM+ infants and 3447 ± 1031 for FM−
                                                  . This corresponds to
mean...

You may provide a definition with
\DeclareUnicodeCharacter
```

```
! LaTeX Error: Unicode character − (U+2212)
               not set up for use with LaTeX.

See the LaTeX manual or LaTeX Companion for explanation.
Type  H <return>  for immediate help.
 ...

l.132 ...M+ videos and 119 ± 36 seconds for FM−
                                                   videos. While the FM−
v...

You may provide a definition with
\DeclareUnicodeCharacter


! LaTeX Error: Unicode character − (U+2212)
               not set up for use with LaTeX.

See the LaTeX manual or LaTeX Companion for explanation.
Type  H <return>  for immediate help.
 ...

l.132 ...seconds for FM− videos. While the FM−
                                                   videos had a slightly
hig...

You may provide a definition with
\DeclareUnicodeCharacter



[3 <./Figure1.png>]

! LaTeX Error: Unicode character − (U+2212)
               not set up for use with LaTeX.

See the LaTeX manual or LaTeX Companion for explanation.
Type  H <return>  for immediate help.
 ...

l.139 ... score (FMs present [FM+], absent [(FM−
                                                  )] or abnormal) was
determ...

You may provide a definition with
\DeclareUnicodeCharacter

LaTeX Font Info:    Font shape `T1/Merriwthr-OsF/b/n' will be
(Font)              scaled to size 7.0pt on input line 149.
LaTeX Font Info:    Font shape `T1/Merriwthr-OsF/b/n' will be
(Font)              scaled to size 5.0pt on input line 157.

! LaTeX Error: Unicode character − (U+2212)
               not set up for use with LaTeX.
```

```
See the LaTeX manual or LaTeX Companion for explanation.
Type  H <return>  for immediate help.
 ...

l.164 \% FM-
                           & 10.8    & 10.8   & 10.2\hspace{10pt}    &
0.9  ...

You may provide a definition with
\DeclareUnicodeCharacter


Overfull \hbox (18.93364pt too wide) in paragraph at lines 153--167
 []
 []

LaTeX Font Info:    Font shape `T1/Merriwthr-OsF/m/n' will be
(Font)              scaled to size 6.8438pt on input line 188.
LaTeX Font Info:    Calculating math sizes for size <6.8438> on input
line 192.

LaTeX Font Info:    Font shape `T1/Merriwthr-OsF/m/up' will be
(Font)              scaled to size 6.8438pt on input line 192.
LaTeX Font Info:    Font shape `T1/Merriwthr-OsF/m/up' will be
(Font)              scaled to size 5.70291pt on input line 192.
LaTeX Font Info:    Font shape `T1/Merriwthr-OsF/m/up' will be
(Font)              scaled to size 4.79063pt on input line 192.
LaTeX Font Info:    Font shape `T1/Merriwthr-OsF/m/n' will be
(Font)              scaled to size 5.70291pt on input line 192.
LaTeX Font Info:    Font shape `T1/Merriwthr-OsF/m/n' will be
(Font)              scaled to size 4.79063pt on input line 192.
LaTeX Font Info:    Font shape `T1/Merriwthr-OsF/m/it' will be
(Font)              scaled to size 6.8438pt on input line 192.
LaTeX Font Info:    Font shape `T1/Merriwthr-OsF/m/it' will be
(Font)              scaled to size 5.70291pt on input line 192.
LaTeX Font Info:    Font shape `T1/Merriwthr-OsF/m/it' will be
(Font)              scaled to size 4.79063pt on input line 192.

Underfull \hbox (badness 1394) in paragraph at lines 197--198
\T1/Merriwthr-OsF/m/n/7.5 (+20) risk fac-tors for neu-rode-vel-op-men-tal
de-la
y. Specif-i-cally, 70%
 []



LaTeX Font Info:    Font shape `T1/Merriwthr-OsF/m/up' will be
(Font)              scaled to size 7.5pt on input line 199.

! LaTeX Error: Unicode character − (U+2212)
               not set up for use with LaTeX.

See the LaTeX manual or LaTeX Companion for explanation.
```

```
Type  H <return>  for immediate help.
 ...

l.199 ...g FM+ and 99 infants were scored as FM−
                                                  . The remaining six
infant...

You may provide a definition with
\DeclareUnicodeCharacter

LaTeX Font Info:    Trying to load font information for T1+lmtt on input
line 1
99.
(c:/texlive/2024/texmf-dist/tex/latex/lm/t1lmtt.fd
File: t1lmtt.fd 2015/05/01 v1.6.1 Font defs for Latin Modern
)
Package microtype Info: Loading generic protrusion settings for font
family
(microtype)             `lmtt' (encoding: T1).
(microtype)             For optimal results, create family-specific
settings.
(microtype)             See the microtype manual for details.
<Figure5.png, id=324, 555.0336pt x 394.8351pt>
File: Figure5.png Graphic file (type png)
<use Figure5.png>
Package pdftex.def Info: Figure5.png  used on input line 213.
(pdftex.def)             Requested size: 244.11394pt x 173.65567pt.

Overfull \hbox (9.0pt too wide) in paragraph at lines 213--214
 [][]
 []

LaTeX Font Info:    Font shape `T1/Merriwthr-OsF/b/sl' in size <7.5> not
availa
ble
(Font)              Font shape `T1/Merriwthr-OsF/b/it' tried instead on
input l
ine 221.
LaTeX Font Info:    Font shape `T1/Merriwthr-OsF/b/it' will be
(Font)              scaled to size 7.5pt on input line 221.


[4{c:/texlive/2024/texmf-dist/fonts/enc/dvips/lm/lm-ec.enc}]
Underfull \vbox (badness 4084) has occurred while \output is active []



<Figure2.pdf, id=360, 532.21234pt x 316.1187pt>
File: Figure2.pdf Graphic file (type pdf)
<use Figure2.pdf>
Package pdftex.def Info: Figure2.pdf  used on input line 230.
(pdftex.def)             Requested size: 488.22787pt x 290.00305pt.

Underfull \hbox (badness 10000) in paragraph at lines 246--247
```

```
[]\T1/Merriwthr-OsF/m/up/7.5 (+20) One avail-able im-ple-men-ta-tion
(STAM: Spa
tio-temporal
 []


Underfull \hbox (badness 2027) in paragraph at lines 281--282
[]|\T1/Merriwthr-OsF/m/n/7 (+20) Reduced vari-ance may sig-nal re-
stricted
 []


Overfull \hbox (4.5081pt too wide) in paragraph at lines 259--285
 [][]
 []

<Figure3.pdf, id=369, 494.60985pt x 469.28755pt>
File: Figure3.pdf Graphic file (type pdf)
<use Figure3.pdf>
Package pdftex.def Info: Figure3.pdf  used on input line 294.
(pdftex.def)             Requested size: 235.11394pt x 223.07858pt.

! LaTeX Error: Unicode character − (U+2212)
               not set up for use with LaTeX.

See the LaTeX manual or LaTeX Companion for explanation.
Type  H <return>  for immediate help.
 ...

l.295 ...al feature clearly predicting GMA score.}
                                                  \label{fig:Figure3}
You may provide a definition with
\DeclareUnicodeCharacter



[5 <./Figure5.png>]
<Figure4.png, id=390, 569.4876pt x 857.604pt>
File: Figure4.png Graphic file (type png)
<use Figure4.png>
Package pdftex.def Info: Figure4.png  used on input line 306.
(pdftex.def)             Requested size: 235.11394pt x 354.05391pt.

! LaTeX Error: Unicode character − (U+2212)
               not set up for use with LaTeX.

See the LaTeX manual or LaTeX Companion for explanation.
Type  H <return>  for immediate help.
 ...

l.307 ...positive rate is equal to 1-Specificity.}
                                                  \label{fig:Figure4}
You may provide a definition with
\DeclareUnicodeCharacter
```

```
! LaTeX Error: Unicode character − (U+2212)
               not set up for use with LaTeX.

See the LaTeX manual or LaTeX Companion for explanation.
Type  H <return>  for immediate help.
 ...

l.311 ... 10–12\% in this lock-box sample (FM−
                                                  ), the PR-AUC of 0.41
subs...

You may provide a definition with
\DeclareUnicodeCharacter


Underfull \hbox (badness 4120) in paragraph at lines 311--312
\T1/Merriwthr-OsF/m/up/7.5 (+20) (PR-AUC), a met-ric par-tic-u-larly in-
for-ma-
tive for im-bal-anced
 []


Underfull \vbox (badness 10000) has occurred while \output is active []




Underfull \vbox (badness 10000) has occurred while \output is active []



[6 <./Figure2.pdf> <./Figure3.pdf

pdfTeX warning: pdflatex.exe (file ./Figure3.pdf): PDF inclusion:
multiple pdfs
 with page group included in a single page
>]

! LaTeX Error: Unicode character − (U+2212)
               not set up for use with LaTeX.

See the LaTeX manual or LaTeX Companion for explanation.
Type  H <return>  for immediate help.
 ...

l.323 ... value were classified as positive (FM−
                                                  ), and those below were
cl...

You may provide a definition with
\DeclareUnicodeCharacter
```

```
<Figure7.png, id=438, 405.6756pt x 424.4658pt>
File: Figure7.png Graphic file (type png)
<use Figure7.png>
Package pdftex.def Info: Figure7.png  used on input line 330.
(pdftex.def)             Requested size: 235.11394pt x 246.0025pt.

Package natbib Warning: Citation `Feurer2015' on page 7 undefined on
input line
 335.


Package natbib Warning: Citation `Feurer2015' on page 7 undefined on
input line
 337.


Package natbib Warning: Citation `Feurer2015' on page 7 undefined on
input line
 337.


! LaTeX Error: Unicode character − (U+2212)
               not set up for use with LaTeX.

See the LaTeX manual or LaTeX Companion for explanation.
Type  H <return>  for immediate help.
 ...

l.340 ...ock-box test set of 186 infants (19 FM−
                                                  ) was randomly selected
be...

You may provide a definition with
\DeclareUnicodeCharacter


Underfull \vbox (badness 10000) has occurred while \output is active []



[7 <./Figure4.png> <./Figure7.png>]
<Figure8.png, id=456, 278.7213pt x 235.6002pt>
File: Figure8.png Graphic file (type png)
<use Figure8.png>
Package pdftex.def Info: Figure8.png  used on input line 350.
(pdftex.def)             Requested size: 235.11394pt x 198.74043pt.
LaTeX Font Info:    Font shape `T1/Merriwthr-OsF/m/up' will be
(Font)              scaled to size 6.0pt on input line 353.
LaTeX Font Info:    Font shape `T1/Merriwthr-OsF/m/it' will be
(Font)              scaled to size 6.0pt on input line 353.

! LaTeX Error: Unicode character − (U+2212)
```

```
               not set up for use with LaTeX.

See the LaTeX manual or LaTeX Companion for explanation.
Type  H <return>  for immediate help.
 ...

l.353 ...-folds cross-validated training results.}
                                                  \label{fig:Figure8}
You may provide a definition with
\DeclareUnicodeCharacter


! LaTeX Error: Unicode character − (U+2212)
               not set up for use with LaTeX.

See the LaTeX manual or LaTeX Companion for explanation.
Type  H <return>  for immediate help.
 ...

l.353 ...-folds cross-validated training results.}
                                                  \label{fig:Figure8}
You may provide a definition with
\DeclareUnicodeCharacter


Package natbib Warning: Citation `Feurer2015' on page 8 undefined on
input line
 358.


LaTeX Warning: `h' float specifier changed to `ht'.



<Figure9.png, id=460, 714.9912pt x 860.7357pt>
File: Figure9.png Graphic file (type png)
<use Figure9.png>
Package pdftex.def Info: Figure9.png  used on input line 382.
(pdftex.def)             Requested size: 235.11394pt x 283.03236pt.

Underfull \vbox (badness 6001) has occurred while \output is active []



[8 <./Figure8.png> <./Figure9.png>]

! LaTeX Error: Unicode character − (U+2212)
               not set up for use with LaTeX.

See the LaTeX manual or LaTeX Companion for explanation.
Type  H <return>  for immediate help.
 ...

l.395 ...allenges due to the small number of FM−
```

```
                                                    infants available for
tra...

You may provide a definition with
\DeclareUnicodeCharacter





[9]

<Figure6.png, id=494, 302.3295pt x 299.6796pt>
File: Figure6.png Graphic file (type png)
<use Figure6.png>
Package pdftex.def Info: Figure6.png  used on input line 428.
(pdftex.def)             Requested size: 235.11394pt x 233.0495pt.

Underfull \vbox (badness 1014) has occurred while \output is active []



[10 <./Figure6.png>]

! LaTeX Error: Unicode character − (U+2212)
               not set up for use with LaTeX.

See the LaTeX manual or LaTeX Companion for explanation.
Type  H <return>  for immediate help.
 ...

l.448 ...y classifier was trained to predict FM−
                                                    infants, which
indicates ...

You may provide a definition with
\DeclareUnicodeCharacter


Package natbib Warning: Citation `Feurer2015' on page 11 undefined on
input lin
e 454.





! LaTeX Error: Unicode character − (U+2212)
               not set up for use with LaTeX.

See the LaTeX manual or LaTeX Companion for explanation.
Type  H <return>  for immediate help.
 ...

l.494     \item[FM−]
```

```
                        Absent Fidgety Movements (GMA Score 2)
You may provide a definition with
\DeclareUnicodeCharacter




[11]
Underfull \hbox (badness 5403) in paragraph at lines 519--520
\T1/Merriwthr-OsF/m/up/7.5 (+20) This work was funded by an NIH-NICHD
grant (Pr
oject#:
 []

(./main.bbl
Underfull \hbox (badness 1803) in paragraph at lines 19--23
[]\T1/Merriwthr-OsF/m/up/7.5 (+20) Herskind A, Greisen G, Nielsen JB.
Early id
en-ti-fi-ca-tion
 []




Underfull \vbox (badness 10000) has occurred while \output is active []




[12]
Underfull \hbox (badness 10000) in paragraph at lines 204--206
[]\T1/Merriwthr-OsF/m/up/7.5 (+20) Ostadabbas S, Fine-tuned Domain-
adapted In-f
ant
 []


Underfull \hbox (badness 10000) in paragraph at lines 204--206
\T1/Merriwthr-OsF/m/up/7.5 (+20) Pose (FiDIP); 2023.
[][]$\T1/lmtt/m/n/7.5 htt
ps : / / github . com / ostadabbas /
 []


Overfull \hbox (0.60785pt too wide) in paragraph at lines 208--212
\T1/Merriwthr-OsF/m/up/7.5 (-20) Pyramid Vision Transformer.  Comput Vis
Media
2022;8(3):415--
 []


Underfull \hbox (badness 1337) in paragraph at lines 220--224
\T1/Merriwthr-OsF/m/up/7.5 (+20) learn-ing.  Nat Neu-rosci
2018;21(9):1281--128
9.  [][]$\T1/lmtt/m/n/7.5 10 . 1038 /
 []
```

```
Underfull \vbox (badness 10000) has occurred while \output is active []



)

Package natbib Warning: There were undefined citations.



[13]
enddocument/afterlastpage: lastpage setting LastPage.
(./main.aux)
 ***********
LaTeX2e <2024-06-01> patch level 2
L3 programming layer <2020/03/25>
 ***********

LaTeX Font Warning: Size substitutions with differences
(Font)              up to 1.0pt have occurred.


LaTeX Font Warning: Some font shapes were not available, defaults
substituted.

Package rerunfilecheck Info: File `main.out' has not changed.
(rerunfilecheck)             Checksum:
73A4E33B676E07C670C65C3F55AF9D7B;8601.
 )
Here is how much of TeX's memory you used:
 25391 strings out of 473583
 491132 string characters out of 5732343
 1979908 words of memory out of 5000000
 47108 multiletter control sequences out of 15000+600000
 2048494 words of font info for 614 fonts, out of 8000000 for 9000
 1141 hyphenation exceptions out of 8191
 123i,16n,131p,2187b,1106s stack positions out of
10000i,1000n,20000p,200000b,200000s
<c:/texlive/2024/texmf-dist/fonts/type1/sorkin/merriweather/Merriwthr-
Bold.pf
b><c:/texlive/2024/texmf-dist/fonts/type1/sorkin/merriweather/Merriwthr-
BoldIta
lic.pfb><c:/texlive/2024/texmf-
dist/fonts/type1/sorkin/merriweather/Merriwthr-I
talic.pfb><c:/texlive/2024/texmf-
dist/fonts/type1/sorkin/merriweather/Merriwthr
-Regular.pfb><c:/texlive/2024/texmf-
dist/fonts/type1/public/amsfonts/cmextra/cm
ex7.pfb><c:/texlive/2024/texmf-
dist/fonts/type1/public/amsfonts/cm/cmsy6.pfb><c
:/texlive/2024/texmf-
dist/fonts/type1/public/amsfonts/cm/cmsy7.pfb><c:/texlive/
```

2024/texmf-
dist/fonts/type1/public/amsfonts/euler/eurm7.pfb><c:/texlive/2024/te
xmf-dist/fonts/type1/public/lm/lmtt8.pfb>
Output written on main.pdf (13 pages, 4016498 bytes).
PDF statistics:
 684 PDF objects out of 1000 (max. 8388607)
 601 compressed objects within 7 object streams
 122 named destinations out of 1000 (max. 500000)
 238459 words of extra memory for PDF output out of 266212 (max.
10000000)

Click here to access/download
Supplementary Material
TRIPODAI_checklist_MSegado.pdf
