# Supplementary material for: A preregistered, open pipeline for early cerebral palsy risk assessment from infant videos
Source: Gigascience. 2026 Jan 20;15:giag003. doi: 10.1093/gigascience/giag003 (PMC13152017; doi:10.1093/gigascience/giag003)
Supplement: giag003_Authors_Response_To_Reviewer_Comments_original_submission [file giag003_authors_response_to_reviewer_comments_original_submission.pdf]

# Author's Response To Reviewer Comments

Dear reviewing editor,

We want to thank the reviewers for taking the time to comment on the manuscript. We have carefully reviewed each of their concerns and made major revisions to the manuscript in response. Notably, we agree with all reviewers that the novelty and value of the manuscript is in the open-source code for video analysis, feature extraction, and model training. We have therefore revised the title of the submission accordingly, and feel that “A Pre-Registered, Open Pipeline for Early Cerebral Palsy Risk Assessment from Infant Videos” better reflects the nature of the work.

In addition, we have substantially revised the manuscript to:
• Better situate our work within the current literature.
• Provide considerable new detail regarding the dataset itself, including its acquisition and processing.
• Offer a more thorough explanation and interpretation of the model's results.
• Clearly articulate the limitations of the current approach and results
• Further emphasize the potential for open science methodologies like ours to advance the field of cerebral palsy detection.
• Clarified our use of terminology (specifically as it relates to the ‘lock-box’ testing)
• Improved all the figures, added several new figures, and carefully checked the text for spelling, grammar, and language mistakes.

We believe these revisions have significantly strengthened the manuscript and more clearly address the points raised. Detailed responses to each specific comment can be found below. Reviewer comments are included in quotations, with responses inline.

Thank you for your time and consideration.

Sincerely,

Melanie Segado (on behalf of all co-authors)

------
Reviewer 1:

“The paper is targeting early CP prediction by proposing an automatic method for assessing infant risk from videos using a high-risk cohort of 1 060 infants. The method combines video-based pose estimation with predefined feature extraction and classification of movement pattern through automated GMA. By focusing on material related to my competencies within machine learning for movement analysis and CP prediction, I have the following remarks which the authors should carefully pay attention to in order for the paper to be worthy of publication:”

We appreciate the thorough feedback, and agree that the paper, if published, will be stronger because of it.

“The authors have focused on developing a simple yet automatic method for detecting fidgety movements that generalizes beyond the training and validation data by relying on a small set of 38 kinematic features derived from video-based pose estimation. This inherently restricts the level of predictive accuracy that can be achieved by the method but at the same time could make the classifier a lightweight, interpretable alternative to high-accuracy deep learning approaches for automated GMA.”

We would like to clarify that the goal of our work was specifically to avoid predicting labeled fidgety movements, an approach that we feel may lead to overfitting due to a reliance on manual annotation of specific frames/videos, and datasets trained on samples that are already known to have differing numbers of fidgets due to the nature of the GMA administration. We do not make any claims about having learned to detect fidgets but rather a movement-based prediction that aligns with clinician-determined GMA scores.

The 38 features are definitely limiting compared to precise featurizations- but that was part of the objective. It is already clear from the literature that precise featurization (like deep-learned fidget detection) yields very high ROC predictions of GMA score. Our goal was different - to see if using very simple features we could achieve similar results. The reason being that these simple features will be computable from all videos of infants moving, and ideally generalize better to other datasets, including those before 3-4 months when fidgets are typically diagnostic of CP (an assumption that we hope to test in future work).

However, this goal may not have been clear as written. We've updated the paper to better reflect the objectives of our work, and the performance of the autosklearn classification relative to the work of others.

“The authors have not currently focused on the interpretability of the method, and hence major revision is necessary for this request to be addressed. The desired outcome would be that the paper illustrates how different kinematic features contribute to the prediction of present or absent fidgety movements through the use of feature importance values provided by the sklearn classification model.”

We agree that including more detail about the kinematic features would improve the paper, so we've added a section describing them, and also discussing each feature was included in the clinician-driven featurization.

However, we disagree that the specific kinematic features that contribute to the prediction are important to highlight in the work, and in fact intentionally chose to avoid doing so, to avoid having others try and use this as a basis for feature selection in future work.

In reality, the auto-sklearn framework uses all of the features to make a prediction, so even if we try to interpret the top “n” features given by the feature importance plot as more important, they are not actually more important than the remaining features in aggregate. Specifically, what we mean is that we have no real basis to conclude that a small number of features, each with high importance, is more relevant than a larger number, each with smaller importance.

While this was one of the main revisions you requested, we hope that our response provides sufficient justification for why these were excluded, and that you agree with our reasoning. We have added this to the text so it is clear to future readers as well.

“The authors repeatedly make incorrect claims and bold statements without support from results or literature. This concern needs to be properly addressed (see further comments in associated sections below).”

We are sorry for our misreading of the literature which we corrected in the manuscript. We have responded to points inline for each of the sections.

“Abstract: «lock-box» set: Use standard terminology (test set or external validation set)”

Our use of lock-box to distinguish from a regular test or external validation set is intentional, and taken from the recommendations proposed by Hosseini 2020 (https://www.sciencedirect.com/science/article/pii/S0149763420305868) to prevent the risk of “overhyping” in machine learning.

Overhyping here refers to the fact that standard train/test splits are typically done after many consequential decisions in preprocessing, feature computation, and architecture selection have already been made, and are not typically pre-registered, so while none of the data points in the test set are used to train the model, the data does still risk biasing the result towards success.

Conversely, a lock-box refers to “a subset of data that are removed from the analysis pipeline at the very start of optimization and not accessed until all hyperparameter adjustments and training have been completed.” It therefore allows for higher confidence in the generalizability of the processing pipeline as a

whole, and not just the model.

However, since this terminology has not been widely adopted in the literature, we have amended the manuscript to include a brief description of the distinction between a standard test/external validation approach and the lock-box approach, clarifying that a lock-box is a more stringent extension of an external validation set.

“Background: "The combined advancements in deep learning, open datasets, and open-source tools have significantly improved the reliability and accuracy of pose estimation and tracking outcomes[24, 25].": This statement should be supported by scientific references, not GitHub links”

We have revised the manuscript to include the relevant links to the literature in addition to the github links; however, in the spirit of reproducibility we feel it is both relevant and important to reference the open-source tools directly.

"Pretrained vision transformers should be sufficiently good to capture the movement features that are relevant for clinical assessment, without the need for custom models that risk overfitting.": Refrain from such statements without references or other evidence”

We based this statement off the truly exceptional performance of vision transformers across all domains of computer vision, including for pose estimation. We have added a line to the manuscript (with reference) indicating why we think this claim is generally true and added additional information detailing the performance we observed on infant data.

"This approach was highly effective at detecting FMs, however, as they noted this approach cannot be extended without the need for retraining on other hand-labeled segments.": Elaborate on this and inform the reviewer about where in the paper (reference 6) this information can be found”

This statement was incorrect as worded and has been removed from the manuscript. The authors did not note this in their manuscript, and we appreciate you pointing this out.

That said, the intent of the statement remains true. The model relies on hand-labelled segments of video with FMs. Training it to detect movement patterns other than exclusively FMs, or retraining on a different dataset would therefore require additional hand labeled segments, and since our goal was to predict risk without relying on FMs we see this as a limitation of their approach particularly as it relates to the generalizability of the method to ages prior to the development of fidgets (0-3 months).

"[…] none employed a "lock-box" set (ie. held-out data points that were not used at any point during the hyperparameter optimization process), raising the possibility that results are overly optimistic [28].": This statement is wrong, there are previous studies that have used a hold-out test set/external validation set for final evaluation (one such example is the study of Gao et al. that the authors refer to in reference 6). The authors should be overly careful when making such claims.”

We agree that it is necessary to be overly careful when making such a claim, and have added a section to the manuscript underscoring what we meant in drawing that distinction. Gao used both an internal (cohort 1) and external validation set (cohort 2), as well as an independent set for FM labelling (cohort 3). Your deep learning work has also used similar splits. and our goal is not to diminish the quality of the work that has been done in the field.

Our goal was to highlight that minimizing the risk of overfitting requires not just an external validation set, which is standard in ML and Medical testing, but a true “lock-box” that has been pre-registered. Hopefully this argument is made more clearly in the revised manuscript.

We have added a line to our manuscript further clarifying why the lock-box/pre-registration are important, and are now in contact with Dr. Lu and Dr. Yu (corresponding authors on Gao 2024) to test how well the MAM model generalizes to our dataset and vice versa. We have not been able to do so yet, but hope to do

so in the near future.

"To compute accurate movement features, we started by selecting an open-source pose estimation algorithm that had high precision on our infant dataset.": How did the authors verify this? There are no results in the paper backing such a claim.”

A section has been added to the manuscript clarifying how we determined that the algorithm was performing sufficiently well on our dataset, and updated the figure to provide additional visual evidence. We have also removed the word “high precision” since we did not measure precision directly, and should have avoided using the term colloquially. Our goal here wasn’t to make big claims about the performance of other researchers' algorithms (which we didn’t test, largely because the model weights were not available), only our own, and have amended the wording accordingly.

For evaluating the datasets, we did not have an annotated ground-truth dataset, so we relied on the opinions of the clinicians involved in GMA scoring. We first tried to find a publicly available algorithm that had been fine-tuned for use on infants, but did not find any with model weights available. We then tested several publicly available algorithms, including HRNet and PVTV2 that we fine-tuned on 10K frames of infant video, but these still (visually) struggled with accurate bone-length estimates, and introduced a lot of high-frequency jitter in wrists and ankles that the clinicians felt could be diagnostically misleading.

ViTPose-H worked (visually) very well off-the-shelf (on the dataset presented in this work, and several others in our lab), and had the added benefit that other research groups would quickly be able to implement without having to fine-tune on new data. We released a set of keypoints extracted from videos from the YouTube 8M dataset using ViTPose-H on figshare (https://doi.org/10.6084/m9.figshare.25316500.v1 ), along with the video URLs as a pseudo “benchmark” for others to see how they perform on videos of infants generally.

Interestingly, a paper published just recently (April 9th, 2025) actually did do a systematic benchmarking of pose estimation algorithms for infant pose estimation. While ViTPose-H performed very well relative to other algorithms, their fine-tuned version of ViTPose performed even better. They have released the weights, and we have updated our manuscript to reflect this.

https://www.nature.com/articles/s41598-025-96206-0

"We show that movement features can predict GMA scores in the largest infant dataset used to date": Once again the authors make an incorrect claim (the study of Gao et al. (reference 6) used a larger sample), indicating that they do not have sufficient overview of related studies and even the references they have included.”

You're right this was incorrect as Cohort 2 in Gao’s study was not a subset of the sample of 1206 videos but rather an additional sample of 283. Thank you for pointing that out!

We have removed references to being the largest sample collected to date, and amended the manuscript to better reflect the sample size of our work in relation to others.

We’ve also clarified in the manuscript that our only goal in highlighting the sample size is to underscore the need for bigger datasets. Gao has a very large dataset - you also have a very large dataset in your work - and so does our research group. Training on more data is better for all of us.

“Patient characteristics
- Lack statistics on risk factors in the sample of recruited infants
- Inclusion/Exclusion Criteria”

Inclusion exclusion criteria are clearly stated in the manuscript under “Data Description” → “inclusion/exclusion criteria”.

Information regarding the risk factors has been added to “Patient Characteristics”

“The authors should include a flow diagram that more intuitively illustrates the process of inclusion/exclusion per criterion (including the number of infants excluded at each stage)”

This is stated in the text, but has been made into a flow diagram for easier visualization.

“"The remaining 931 videos were split into an analysis set (744) and a lock box holdout set (187). The analysis set was further split into train/val/test sets (558, 93, 93), each of which had a 12% representation of the "absent fidgety" movement type.": Currently, there is confusion related to the datasets in the paper, especially the use of three dataset splits, "train", "val" and "test", in addition to the "lock-box" set. What makes it even more confusing is that "train" and "val" are together referred to as "training set" (e.g., Figure 1). The reviewer proposes to stick with common ML methodology and use three dataset portions, "train" (651), "validation" (93) and "test" (187), with the last being the "lock-box" set.”

We agree that as worded this was needlessly confusing. It was written for technical correctness in relation to the pre-registered splits, but we have modified it to be more intuitive for the reader. The discrepancy arose because Auto-ML uses successive halving during model training and as such only requires a Train/Test split as opposed to the Train/Validation/Test split typically used (it implicitly uses the validation sets).

“Analysis
- "Infant videos pose unique challenges for pose-estimation algorithms due to frequent irregular body poses, the presence of body-like objects (e.g., toys or cartoons), and high levels of self-occlusion.": The authors should also mention the different anatomical proportions of infants compared to adults, and refer to the study of Sciortino et al. (https://link.springer.com/chapter/10.1007/978-3-319-68548-9_38).

- "Conventional algorithms, such as OpenPose[29], often fail in such conditions, leading to unreliable pose estimates [22]. Fine-tuning is often required to improve accuracy on each infant dataset.": Lack of infant-specific references, e.g., https://www.sciencedirect.com/science/article/pii/S0895611121001610.”

Thank you for bringing these references to our attention - we have included them in the revised manuscript. However, it is worth noting that modern vision transformers are not sensitive to the input body proportions of the subject for 2D pose estimation and this is therefore much less of a concern than for other algorithms. Our updated figure now shows additional details highlighting the point.

"However, we found that a pre-trained vision transformer, ViTPose-H [19], performed better than fine-tuned alternatives [30, 31, 32, 29, 33], obviating the need for manual finetuning.": How was this evaluated? There does not seem to be any results in the paper comparing the accuracy of ViTPose-H to a fine-tuned pose estimation model. Currently, there is only a single example image in Figure 2. For the authors to claim that pre-trained ViTPose-H is better than fine-tuned alternatives, this should be supported by quantitative evidence on a sufficiently large sample of infant images/videos.”

“Furthermore, the fine-tuning procedure of OpenPose is not mentioned in the text and as an outdated method OpenPose is not representative of the performance that could be achieved through fine-tuning. Hence, it is better to avoid such statements to avoid confusion and invalid conclusions.”

As clarified in the previous response, a section has been added to the manuscript describing the process that was used to select ViTPose-H as the algorithm for this work. Prior to switching to ViTPose we tested a few different pose estimation algorithms (exclusively on the training set, not the holdout) and reviewed the results with clinicians. We found performance was much worse than VitPose-H, especially with regards to the stability of detected keypoints of the wrists/ankles.

Unfortunately none of the videos included in this work were consented for publication or re-distribution, and so releasing the full keypoint time series for public review/algorithm benchmarking is not possible. In

lieu of this, we have released a set of key points for a (relatively) large sample of 94 youtube videos (available: https://doi.org/10.6084/m9.figshare.25316500.v1 ) which can be visualized and directly compared to the performance of a previously used algorithm (openpose finetuned on infants). Additionally, we have replaced the initial figure with an improved version showing examples of the types of improvements we observed with the ViTPose-H algorithm relative to the algorithm our lab was using previously (finetuned Openpose). We have selected frames illustrating 3 specific points where algorithms often fail - lost keypoints on low contrast backgrounds, bone length distortions, and inability to handle self-occlusions.

I should also clarify that we are not making any claims about the performance that could be achieved by fine tuning - fine-tuned ViTPose-H has now been shown to be better than off-the-shelf by a different research group (https://doi.org/10.1038/s41598-025-96206-0). However we specifically chose to go with a pre-trained algorithm that did not require fine tuning to improve the likelihood that performance on datasets beyond what we have collected will be similar to the performance we achieved on ours.

That said, as previously mentioned, a separate research group has actually fine-tuned ViTPose and shown that it works even better on infants in their dataset (especially for correctly estimating the width of hip keypoints), so we’ve amended the manuscript accordingly, and will be testing this on our data in future.

We have amended our wording to better reflect that our conclusion is not based on a published benchmark, nor a direct comparison to the work of others, but rather an internal benchmarking process to select an algorithm whose performance aligned with clinician judgement.

“A set of 38 kinematic features was selected based on clinician input [34, 35], designed to capture the displacement, speed, velocity, acceleration, and entropy of key body parts, specificallywrists, ankles, elbows, and knees (Figure 3).": The authors should provide a list of the specific kinematic features along with their biomechanical definitions.”

The feature descriptions are well documented in previous work, listed on the pre-registration site, and their computation is well-described in the github repository. We have also added a table explaining what the features are, and why they may be relevant for early cerebral palsy risk prediction.

“Figure 3 is way too small for information to be consumed by the user.”

We agree and have updated the figure with something that is easier to read.

“Testing on the lockbox set yielded an AUC-ROC of 0.79 (Figure 4), closely aligned with the cross-validation performance, indicating minimal overfitting.": While the AUC on the test set indicates that the model does not overfit on the training and validation data it should still be addressed that the AUC is still moderate (for example, compared to the study of Gao et al.). However, the authors could argue for the interpretability of the proposed method, but this should then be backed by results on feature importance of the selected set of biomechanical features.”

Yes, the AUC is moderate compared with previous work, and we have added a line to better reflect this comparison in the manuscript.

The interpretability of this approach is in using a simple movement-based vector, not in the specific feature weights selected by the model. While it may feel significant to think of features with higher weights as more important than others, the reality is that the model is using some combination of all features to arrive at the classification result. There is no value in stating that the top 3 features had higher weights than others when the distributions of those features do not clearly distinguish between Fidgety/Absent Fidgety infants, and if anything may lead readers down an incorrect path of trying to select their own features based on our feature importance result.

The novelty of this work isn’t having the best-performing model, it’s having a model with rigorous, fully open methods and a framework for data processing and model training that others can easily replicate on their own data.

“Discussion
"We used an exceptionally big sample (training set: 558, overall >1000 infants)": The authors should stay objective and avoid such exaggeration”

This is an exceptionally large sample size (as are those of Gao et al 2023, and datasets used in your own work). We have modified the text to reflect the fact that others also used exceptionally large sample sizes.

On that note, we are actively seeking collaborators to aggregate data across clinical sites, either through de-identified features, or federated learning.

“We found that our algorithm performs well (AUC-ROC 0.79).": Such a statement should relate to the accuracy achieved in other studies on automated GMA, e.g., https://www.nature.com/articles/s41467-023-44141-x.”

We have included a sentence better situating our model performance within the broader context of the literature. That said, our goal was not to push state of the art performance, and we make no claims about this model performing better than others. Our goal was to determine whether classification could be achieved at all without relying on any of the standard ML techniques that boost performance at the cost of interpretability/generalizability.

“Based on our rigorous pre-registered approach with a lock-box set we can be confident that we did not do any overfitting and that it will generalize well to other datasets.": This is a bold statement, since the model will not generalize beyond the characteristics of the dataset used for training.”

Absolutely, it is never possible to know out of distribution performance of the trained model until someone else tests it on their data. What we meant was that the process would generalize to other datasets, and have made this much more explicit in the wording. That said, we are confident that none of these steps were overfit to our specific data and should generalize to other datasets.

- We didn’t optimize the pose estimation method for our videos (and tested them on several other datasets). Others have since tested it and found decent results under more rigorously benchmarked conditions (see: Jahn et al. 2025).
- Our features are general movement descriptions selected years before this dataset was even collected (see: Chambers et al. 2020), as opposed to precise featurizations selected based on our dataset.
- AutoSklearn will optimize hyperparameters for new datasets similarly to how it did for ours.

“The clinician-selected movement features offer only a coarse description of movement, whereas we know from the clinical literature that the difference between infants whose movements are typically developing and those that are not is often subtle.": The authors should include references that support such a claim.”

True, we have added a reference to support this claim and a more nuanced explanation of the point we were aiming to convey. In short, we were thinking of the subtle combination of features that are reflected in fidgets, and other descriptors like “cramped synchronized” or “writhing” movements.
We agree and have addressed this by including a supporting citation and clarifying our intended meaning. Specifically, we aimed to capture the subtle interplay of characteristics present in fidgets, along with other descriptive terms such as “cramped synchronized” or “writhing” movements typically used in quantifying differences between typical/atypical motor development in infants.

“Given that the clinician-selected movement features can predict GMA score in such a large sample, we have a strong indication that models that include even more data from a wider sample of infants, and more precise features, should perform even better.": How can you be sure about this?”

Our expectation that models incorporating a wider sample of infants and more precise movement features will perform better is based on both empirical evidence and established principles in machine learning and movement analysis. The strong predictive power of clinician-selected features in our study suggests that

additional, more nuanced features could further enhance performance by capturing subtle movement patterns. Fidgets themselves are one example of how very subtle differences in wrist and ankle movements can be highly indicative of CP.

Additionally, broader and more diverse datasets typically improve model generalization by reducing biases associated with limited demographic or clinical representation. Similar trends have been observed in other areas of clinical assessment and neurological disorder detection, where increasing data diversity and feature richness leads to better predictive accuracy.

While we acknowledge that more data and refined features do not guarantee improved performance in all cases, prior research and the nature of data-driven modeling strongly support this expectation. Our goal here was really to motivate the idea that we should find ways to pool datasets across sites (including sharing standard sets of de-identified features or implementing federated learning pipelines).

We have clarified this point in the manuscript, and made it much more clear why we are making such a statement to begin with.

Potential implications

“Overall we have shown that advances in pose estimation now make it entirely realistic to get precise movements from infant videos without the need for any specialized camera setup or finetuning.": How can you be such confident without any results on the preciseness of pose estimation?”

The point is less about the specific precision of the pose estimation algorithm, but rather the broader point that achieving performance better than what used to require custom trained algorithms, manual annotation, or considerable fine-tuning can be accomplished with pre-trained models that are widely accessible.

The fact that we were able to obtain high-quality pose estimates from hand-held iphone/ipad videos without any finetuning does really demonstrate that this is entirely possible for other research groups to achieve.

We have added additional details to the manuscript regarding the performance of the algorithm, and in the meantime a separate research group has done an excellent systematic benchmark that we cite.

“Methods
"Model selection and hyperparameter optimization were carried out using the Auto-sklearn 2.0 package [41, 42], with the "vanilla Autosklearn" setting.": The authors should mention which specific machine learning models and hyperparameters that were explored using auto-sklearn.”

We agree that we gave too few details about what “vanilla settings” refers to. This has been expanded upon in the revisions in a new section that discusses AutoSklearn in more depth.

Regarding the model and hyperparameters. We specifically chose not to do this in order to avoid having readers try to pick specific models based on our findings. The hyperparameters and models will change depending on the training data, and over-interpreting their significance would be a mistake. The point here was that other researchers should also train AutoML models on their datasets, not necessarily that this model is uniquely good. This has been made much more clear in the manuscript.

“References
- Figure 2 not referred to in main manuscript
- 27 and 28 seem to be duplicate references”

This has been corrected in the new manuscript.

“Typos

- Lack of brackets and parenthesis
E.g., references 6 and 25 mentioned without brackets
"(typically wearing a diaper only"
- "Lage" in Figure 1”

Thank you for catching these - we’ve fixed them in the manuscript and proof-read the revision more carefully.

------
Reviewer 2:

“The paper describes a pipeline to automatically classify movements based on the General Movement Assessment (GMA) in a relative large dataset (931 infants after application of exclusion criteria; including 105 infants with a positive score on GMA i.e. absence of fidgety movements). This paper contributes to the innovation of the recent years to develop a tool to assess risk of developing cerebral palsy within the first months from simple video recordings. This paper provides an interesting step forward by using a general model for body point tracking from videos and explainable (pre-registered) features for the machine learning step.”

"Overall the paper is well written, however I have comments mainly on the presentation and discussion of the results."

"In general I recommend the authors to align the paper with one of the current guideline and standard frameworks for AI in medicine (e.g. TRIPOD+AI checklist)."

Title: "with video-based tracking" does not fully represent the method applied. I would be more specific in the title."

Thank you for the comments - we agree that the title was not a good representation of the paper and have updated it to “A Pre-Registered, Open Pipeline for Early Cerebral Palsy Risk Assessment from Infant Videos.” We think this title much better reflects the fact that this paper is really describing a set of methods as opposed to a scientific innovation or new state-of-the-art model for CP prediction.

Regarding the reporting frameworks, part of the GigaScience review process required us to report methods using the DOME-ML framework. We have additionally filled the TRIPOD+AI checklist and included it with the supplementary materials.

"Abstract:
All information on the dataset should be provided in the abstract (i.e. cases excluded, distribution of positive/negative classification of GMA). Additional performance metrics of the model should be eventual provided, see comments within result section."

We agree and have updated the abstract accordingly.

"Background:
The last paragraph of the background reads as a summary of the method section, including results. I would expect objectives/research questions and hypothesis there."

We have now included an objective since that was clearly missing in the original submission. Thank you for the suggestion.

Regarding the hypothesis, the article is being submitted as a “Technical Note” as opposed to a research article. Because of this the description of methods/results in the background section is more relevant than the standard hypothesis that would normally be expected.

"Data description:

Data availability: I tried to find the calculated features on OSF but could only find the .csv of the different datasets including patient characteristics and GMA scores within the OSF Storage. Please provide the links for the feature dataset. I appreciate the preregistration of the features. "

Thank you for pointing out the ambiguity of where the features have been uploaded. We have provided a direct link to them in the manuscript, and provided the link here for your reference: https://osf.io/gztmd/files/osfstorage

Features are available averaged over the whole video "features_final_total" and in 2-second windows "features_window_total"

If you have any access issues please let us know and we'll fix it as soon as possible.

"Analyses:

Was there any converting/preprocessing applied such as cropping, stabilization, rotation, scaling , cutting to the same length to the videos before skeletal tracking? Length and frequency of the videos could matter for some of the features, thus it should be stated how this is handled."

No additional video processing was performed prior to skeletal tracking in an effort to develop a process that is invariant to such manipulations.

A description of the video parameters (including number of frames) has been added to the manuscript. We also ensured that the number of frames did not differ between our fidgety/absent-fidgety videos. These methods and the resulting statistics have been added to the manuscript in a new section called "Video characteristics"

We chose not to standardize the length of videos, even though it could affect IQR, for the following reasons. First, an initial check confirmed that the average video duration (number of frames) did not significantly differ between our positive and negative outcome groups, mitigating concerns that video length could act as a systematic confounder at the group level. More importantly, each video was recorded by clinicians with the explicit aim of capturing a sufficient epoch of movement to reliably perform the General Movements Assessment. Thus, the duration of each recording reflects a clinically determined window deemed adequate for observation. The raw IQR within such a window directly reflects the movement variability pertinent to this clinical judgment. Normalizing by total frame count might obscure true differences, particularly given that the proportion of frames containing active, analyzable movement could vary from one clinically sufficient recording to another.

This information about the decision to not standardize lengths has also been included in the revised manuscript.

"Feature relevance:
I would suggest to mention all 38 features within the paper (within a table or supplement) for the reader's convenience and also how these features relate to the clinical observations (i.e. variability, consistency, symmetry). Within Figure 3 the abbreviation of features are within the headings, however these are very small and abbreviations are not explained within the legend. Also the x and y axis labels are missing in the figure."

We agree and have included a table in the supplementary material describing each of the features. Figure 3 has also been revised for clarity.

"Performance metric:
The authors should not only report the AUC, especially as this can be misleading in imbalanced dataset which is the case here (931/105). The recall of 0.34, should be mentioned in the main text and abstract and not only in the legend of Figure 4. I would also appreciate a confusion matrix of the locked-box set as

this helps to interpret the results. "

This is an important point. The Precision/recall tradeoff has been integrated into the text and a sentence has been added explaining its significance in the context of imbalanced data. The confusion matrix has also been added for clarity.

We have also added a section clarifying that AutoML also optimizes resampling strategy to handle class imbalance so it is clear how this factor was considered.

"The author should also discuss what the metrics mean in the current use case and discuss on setting appropriate classification threshold for risk assessment of cerebral palsy."

We agree and have added this to the discussion. Admittedly our result is not groundbreaking in the field, we have amended the wording in the manuscript to reflect this. For real-world applications, sensitivity and specific would need to be much higher than how our model performed.

"Discussion/potential complications:
To my view, within a risk screening assessment of cerebral palsy - maybe in the future widely applied in a general population- we are willing to accept a higher "false positive" rate with infants being extra checked by a specialist and with the possible consequence that more children get extra intervention/stimulation on motor development by a physiotherapist. I think we are less willing to accept "false negatives". However, this needs to be discussed with stakeholders within the field. "

Yes, this is an important point and a more thorough discussion of the clinical interpretation of the findings has been added to the discussion of the manuscript.

"Efforts should be taken to realize data pooling from different groups working on automated GMA assessment and prevent data silos. I would encourage the authors to make data as far as possible available, as well as the trained models. Alternatively federated learning could be an option to strengthen the models for cerebral palsy risk assessment and should be explored in the future. "

This is by far the most important goal of our work, and should have been better reflected in the manuscript from the beginning. We've modified the conclusion to make this goal explicit. The difficulties in sharing datasets across sites was one of our main motivations for releasing not only the features and model, but also all the code required to replicate the results across clinical sites. None of us can share videos, or even skeletal pose estimates. What we can share are features and model weights, and we think that all of us should.

We're also very interested in working with others to set up systems for federated learning. The ability to train models on all of the thousands of videos researchers have collected around the world (securely) would be a huge step forward for the community. Thank you again.

-----
Reviewer 3:

"Your dataset presents a significant contribution to the field of automated risk assessment for cerebral palsy, particularly given its large sample size and rigorous methodological validation. However, for the benefit of readers and potential users of the dataset, additional details regarding the data acquisition process would be valuable."

Thank you for the review – we've updated the manuscript based on your suggestions, and hope that it is now more informative for others that want to build off our work.

"Recording Environment:
1. Were the videos recorded in a controlled clinical setting, or were there variations in background, lighting, and positioning?"

Videos were recorded as part of standard of care through the CHOP neo-natal follow up program. Videos were varied in background, lighting, and while they were taken from a top-down perspective there was some variation in positioning due to the fact that these were handheld cameras. We have added a section called “Video Characteristics” discussing these points.

We didn’t directly quantify the differences in background/lighting, and this is something we hope to do in future - both for data quality control when processing even larger datasets.

"2. Did all infants undergo assessment under similar conditions, or were there differences that might impact model generalizability?"

Most of the videos were collected in a hospital setting under similar conditions, and all of the infants included in this dataset were at elevated risk of cerebral palsy due to hypoxia/injury at birth. This could certainly impact generalizability. Aggregating across different datasets will be essential for developing a model that will generalize at a population level, and we have clarified this point in the revised manuscript.

"Video Quality and Resolution:
3. What was the resolution and frame rate of the recorded videos?"

All of the video information has been added to the manuscript in the new “Video Characteristics” section. details were released in the OSF data repository along with the extracted metrics. Generally, videos were recorded in landscape orientation at 1280x720 resolution and a framerate of ~30fps.

"4. Were there any preprocessing steps applied to standardize video quality across different recordings? Standardization of Data Collection:"

No, and this is something we hope to explore in future. Video quality was assessed by 2 expert reviewers trained in GMA administration and deemed to be “usable” based on their own criteria. As such, we chose not to apply any pre-processing steps. Our goal was to determine how well algorithms work “off-the-shelf” without applying any specific pre-processing of the videos to improve the likelihood that the approach would generalize. However, optimizing the video pre-processing for feature extraction is something we are actively working on and hope to do in collaboration with other researchers.

"5. Were specific protocols followed to ensure consistency in video capture, such as standardized camera angles or infant positioning?"

Anyone capturing videos (mainly hospital staff, a minority of videos were collected by parents) was instructed to capture them from a top-down perspective with the infant centered in the frame, free of any distractions, and unoccluded. Videos were collected with hand-held cameras, so the perspective may vary slightly, but we have carried out additional analyses to verify that the proportions of the normalized infant poses are consistent with a top-down perspective.

In future we hope to integrate these preprocessing steps, since camera angle can have a huge impact on both the quality of pose estimates and also the derived features (especially measures of Left/Right symmetry).

"6. Were variations in clothing, movement freedom, or recording duration controlled or documented?"

The conditions for video collection are included in the manuscript under “Collection of a large clinical dataset. ”:

Infants were observed in minimal attire for unobstructed visibility of the trunk, shoulders, and extremities to facilitate the observation of natural movements (typically wearing a diaper only)

In the future we hope to leverage advances in computer vision to be able to quantify these differences

automatically, for instance segmenting individual clothing items and documenting them, to ensure that these factors are not influencing model performance at scale.

"Providing these details would enhance the dataset's usability and reproducibility for future studies. Thank you for your work on this important topic."

Thank you for the detailed review. The addition of the Video characteristics section has made this a stronger, more comprehensive manuscript.

-----
Reviewer 4:

"Summary

This paper proposes an automated method for predicting the risk of cerebral palsy in infants based on the General Movements Assessment. Videos of infants are recorded, from which 2D body keypoints are extracted using ViTPose. From the tracked keypoints, a set of features, which has been defined by clinicians, is extracted, which is then used to classify the infant as having present or absent fidgety movements.
The data set used consists of around 1000 videos, which were clinically assessed by trained GM experts. One part of the data set was defined as held out test set, and the remaining data was used to evaluate the method in a cross-validation manner.
Results showed an AUC of 0.79 for the held-out test set, with a true positive rate of around 80% at a false positive rate of around 25%. Cross-validation results were reported to lie in a similar range at an AUC of 0.73.
The code for the pipeline, including keypoint estimation, feature extraction, and classification is publicly available. For the used dataset, the computed features together with clinical scores are published, while the key point data is available on "reasonable request".
This paper tackles a very relevant topic, the automated early detection of CP, which could have a big impact, since it could enable wide-spread screening, and therefore detect more children with CP earlier and therefore lead to an earlier start of treatment and therapy.
The paper is well written and uses one of the largest datasets in automated CP detection literature. The experimental design is valid, and follows a strict process, by registering the method and a test set before running the experiments. The research community could benefit a lot from this large data set, since that is one of the things that has been lacking for a long time - a dataset on which researchers can compare their methods. Up to now, most methods have only been evaluated on private, small datasets, which made the interpretation of results difficult.

Thank you for the detailed review. We hope the code and features are useful to other researchers, and are actively seeking out collaborations to train on larger, multisite data (either through feature dataset aggregation, and/or federated learning).
However, there are several things that should be improved in the paper.
Data set
More information about the data set needs to be added:
- Was the recording process standardized? Did the recorders get specific instructions?
- How was video recorded? From the top of the infant, from side view, at an angle, ...?
- Was the camera position static (on a tripod) or was it a handheld with potential moving?
- Were the infants lying on a table/floor?"

We agree - this information was lacking in the initial draft. To address this we have added a new section called “Video characteristics” that provides many details about the videos themselves, as well as additional details regarding how the videos were collected. This information is located under “Collection of a large clinical dataset”

Specifically:
- The recording process was standardized to the extent that videos were to be recorded from a top-down perspective, with the infant supine in minimal attire centred in the frame. The infant was supposed to be free of distractions and occlusions
- The camera position was not static, it was hand-held, but we have added additional analysis to show that the aspect ratio for the infant poses is roughly square indicating that the videos were not distorted (as would be expected if they were collected side on). This point is really important since both pose estimation and feature computation suffer if the perspective is not properly accounted for.
In case parents filmed the infants at home, was the recording setup the same as in the clinic?
Yes, in cases where the parents filmed the infants at home, they followed the same process as infants recorded at the Children's Hospital of Philadelphia. While we don't have information about where the videos were collected, all videos were recorded with hand-held devices, following the same instructions, and all videos were deemed "evaluable" by 2 expert reviewers trained in GMA administration. As such, we do not anticipate any differences between videos collected in the two locations.
In fact, this is one of the strengths of this dataset since we hope to pursue analysis of at-home videos in the future, towards development of a global pre-screening tool.

"Information about "two fully out-of-sample infant datasets" - same as above. Are there differences between these datasets and yours?
Description of recordings lacking - camera position, handheld, top, front, side? Very relevant for 2D → generalization ability (lock-box set), description of other datasets used??"

The recording parameters (top down, handheld camera, supine infant) were already described in the text ("Collection of a large clinical dataset") and we have added additional details regarding the video collection in a new section called "Video Characteristics".

We have also added additional details regarding the other dataset used to select the pose estimation algorithm.

"ViTPose
The claim that ViTPose has better accuracy than other models is made several times. However, there are no quantitative results backing up this claim. There is one example image, but I don't think this is enough to convince the reader.
"...selecting an open-source pose estimation algorithm that had high precision on our infant dataset."
"ViTPose-H [19] performed better than fine-tuned alternatives"

We agree that the direct comparison with other algorithms would require additional quantification, which we did not perform. However, in the interim, another research group has actually performed a comprehensive quantification of various methods (see: Jahn et al. 2025). They found that ViTPose did perform very well out-of the box, and also released a set of fine-tuned weights specifically for infants that we hope to evaluate in the next iteration of our work.

We added more details regarding the pose estimation selection process to the "Selection of a pose estimation algorithm section." We tested several algorithms and compared them (visually) with the algorithm that was previously being used to analyze this dataset (fine-tuned openpose). During the algorithm selection process we tested both 2D and 3D pose estimation algorithms (PVTV2 and HRNet, fine-tuned on infants). We did not have a ground-truth annotated test set to benchmark against, so we iteratively discussed the resulting pose estimates with expert clinicians to evaluate whether or not they would consider the pose estimates stable enough for pose estimation.

While our claims regarding ViTPose are somewhat subjective due to the lack of systematic benchmarking, we have released a full set of de-identified keypoints for a separate set of youtube videos of infants for direct comparison with the original fine-tuned openpose algorithm used by our group in previous work (available here : https://doi.org/10.6084/m9.figshare.25316500.v1 ).

Additionally, we have improved the figure to show side-by-side examples of poses across several frames, highlighting common challenges in infant pose estimation like occlusions and incorrect bone-length estimates.

"The robustness and generalizability of ViTPose-H were validated through iterative review of pose estimates by clinicians trained in the General Movements Assessment (GMA). "
"This model was further tested on two fully out-of-sample infant datasets (n = 300, ages 0-4months), achieving consistent skeletal tracking performance."
"Overall we have shown that advances in pose estimation now make it entirely realistic to get precise movements from infant videos without the need for any specialized camera setup or finetuning."
- It sounds like evaluations have been made, but the experiments and their results need to be reported in detail.
- What does the "iterative review of pose estimates" mean? Did the clinicians perform GMA on visualized keypoints only? "

We did aim to do a key-points only GMA evaluation on a previously selected candidate algorithm; however, the clinicians could not evaluate the GMA as the smoothing algorithm we selected for that iteration had too much small-variability jitter making all videos look fidgety. As the evaluation was very time-intensive, we used this feedback to guide our choice of algorithm/smoothing parameters, and consulted with the same clinicians to determine whether the new parameters addressed the limitations of the previous one, but did not fully re-evaluate.

We have added a section to the manuscript further elaborating on how the algorithm for keypoint estimation was selected. We have also added additional details regarding the other datasets used for evaluation of the algorithm (summarized in a new figure: Figure 2) and the expanded “Selecting a pose estimation algorithm” section.

"
- What did the "out-of-sample" infant datasets look like? What is different to your dataset?

Yes, the additional out-of-sample evaluation of ViTPose was done on a completely different dataset of infants from a different experiment, as well as 94 videos from the YouTube-8M dataset.We hope that the figures and descriptions added regarding the algorithm selection address concerns.

More detail should be added to the processing of the videos. E.g. if there was some kind of video/keypoint normalization to handle different video resolutions or different distances to the camera (= different number of pixels per infant/different "size" in images), camera rotations, zooming or camera movement.

We absolutely agree and have added two sections to address this. First, the “Video characteristics” section now contains more information about the videos themselves. Second, the section regarding “Processing the keypoint time series” contains several additional paragraphs where we describe how pose estimates were rotated, smoothed, and normalized prior to feature computation.
In brief, we rotated all frames so the center of the infant was aligned with the vertical axis, and scaled to a torso length of 1. By doing this we unfortunately remove a lot of interesting information about the size of the infant and the torso movements, but allows for feature computations to be much more standard across infants and removes variability that could be introduced by camera movement.
In future we hope to use features that are more invariant to camera position so that less pre-processing is required and we can preserve more of the interesting variability.

"The post-processing sounds like it could significantly change the input data (interpolation, smoothing). Could you add some statistics on how many frames were excluded, the percentage of interpolated frames/joints, and how many outliers were removed? It could be also interesting to show a plot of a raw sequence of keypoint values vs. a post-processed one, so the reader can better understand how big the

actual changes are."

Absolutely. We have added a section about the number of frames that were retained, as well as the distribution of frames across videos. Only 10% of frames had keypoints below our strict cutoff of 0.8, and none had frames below 0.7 across all keypoints (excluding the face).

We have also added an additional figure that shows the effect of the mean/median filter that was used in lieu of threshold based outlier removal.

"Smoothing will always come with a loss of information. As is mentioned before, subtle differences could be relevant to distinguish between typically developing and high-risk infants. Could you comment on the effect of smoothing with respect to this? Is it really necessary to apply smoothing? Is there a lot of "jitter" in the keypoints?"

Yes, smoothing will always come with a loss of information and we did aim to minimize the extent to which we pre- and post processed the data for this reason. We initially tried smoothing the data with a much less aggressive one-euro smoothing to preserve the small-amplitude variability while smoothing the more ballistic trajectories (our initial attempts are documented on the pre-registration as well). However, an in-house evaluation by clinicians found that this produced far too much noise on the same amplitude as the expected fidgets. The more aggressive smoothing (used previously by the Kording lab for Chambers et al 2020) was determined to be a better alternative.

We have added an additional figure to the manuscript (Figure 6) showing the effect of smoothing on the keypoints.

We agree, however, that this is certainly not the optimal smoothing parameters and that future work should focus on preserving more of the raw data wherever possible. One of our key motivators for releasing the processing pipeline in its entirety is to encourage other clinical sites to build on our work using fully independent datasets to ensure that the pre-processing parameters do not end up working for our videos only.

Features
"A set of 38 movement features was derived from expert clinician input to ensure clinical relevance."
I don't think this statement is necessarily true. If the features selected by clinicians do not lead to good predictions, there is no clinical relevance. If some features, e.g., deep-learned ones without any connection to the clinician's features, perfectly solve the problem, then this is clinically relevant."

We absolutely agree with this point. Our features were determined in close collaboration with clinicians based on what they thought would be relevant for distinguishing between low vs high risk infants to align them with clinical expertise (see: Chambers et al. 2020). "Ensure relevance to clinicians" is a clearer wording to capture this and so we've amended the wording in the manuscript accordingly.
We have also added a table to the manuscript listing the features, as well as the rationale behind their inclusion. This should clarify what we meant by "relevance."
That said, we agree that these features are simple, course movement descriptors and many more relevant features are likely to exist. This includes, for instance, fidgets which at 3-4 months have been shown to work with very high accuracy (see: Gao 2023).

In future work we hope to include more of these precise features as well, especially as we establish multisite collaborations to avoid overfitting to any one dataset.

"By excluding any direct features related to GMA-specific fidgety movements, we aimed to ensure generalizability and avoid overfitting the model to the clinical dataset."
I don't really understand this reasoning. If FM-specific features work best, why not use them? You can still test for overfitting. It would be great to find features that work extremely well for the given dataset (using cross-validation). If you show that they do, and then show that they don't generalize to other data sets,

then you can go back to other features. But if you have features you think are best for the job, you should evaluate them.

Detecting FMs directly is one promising avenue for CP risk prediction, and one that other groups have already explored. However, evaluating FMs at 3-4 months is, in our opinion, rather restrictive and biased towards success in a dataset where videos have already been curated to capture the fidgety/absent fidgety split.

Nevertheless, we definitely hope to integrate these additional features in future. As mentioned previously, a key step towards this will be establishing multisite collaborations, and implementing strategies like federated learning, so we can add precise features without risking overfitting to a specific dataset.

"It is mentioned that the (aggregated) feature vector that is used for classification is explainable, but it seems to me that the classifier is a black-box, and there is no way to tell based on which features/weights the classification was made. If this is possible, there should be an analysis of the predictions, regarding which combination of features is most important/can be trusted most."

The feature vector is explainable precisely because each of the features was determined in discussion with clinicians and no feature selection was performed based on factors like feature importance.
Analyzing the weights/importance of specific features is, in a sense, irrelevant and likely to lead us, and others, to overfit their results in the future. While it feels more explainable to conclude that the top 3 features that explain the most variance are more important than the others, the reality is that the remaining features with small weights, together, explain as much variance as the top 3. This is why we placed such emphasis on only including features that we could justify based on clinical expertise, and not performing any feature selection based on preliminary results.

"The AutoML method to find a good classifier should be explained in more detail. From what I understand, it tests many different classifiers, preprocessing methods and different hyperparameters, and selects those that work best. It should be described in more detail which model and settings were selected for the experiments."

We agree and have added an entire section discussing the AutoML framework, including the settings we used to train the model "AutoSklearn-2.0 settings"
AutoML selects models and settings tailored to the unique properties of the training data, reporting these specifics could mislead readers into thinking they are universally optimal, which is unlikely to be the case. Instead, we emphasize the process and its contribution to model generalizability, rather than any one particular model configuration. The idea is to iteratively train this model on increasingly large datasets across multiple sites, and the fact that the training framework adapts itself to new input statistics makes this much more realistic than a model with hyperparameters tuned to one specific dataset from one specific site.
Although we think it is likely that the method will generalize well to other datasets from similar domains, and one of the central claims of our paper is that using AutoML enables better generalization than hand-curated models, we have intentionally not included detailed descriptions of the final model architecture or hyperparameters (stochastic gradient descent with a modified huber loss function). This is because our aim is to highlight the value of the AutoML-based approach in identifying an effective classifier, rather than to recommend a fixed model or parameter set for broader use.

"Results/discussion
It is mentioned several times, that the risk of overfitting is minimized by
* using a lock-box test set
* doing feature selection based on clinicians' opinion
* not using FM-related features
I think using a separate test set will not prevent overfitting, it will only make it visible. And I don't really see why the last two should minimize overfitting. Please elaborate."

These are important points, so I'll try to address each of them on its own.

Lock-box set: While any test set ultimately serves to make overfitting visible, our pre-registered 'lock-box' approach plays a more active role in minimizing the risk of developing and reporting an overfit model, precisely through the accountability it enforces. By pre-registering the specific sample IDs for the lock-box set and committing to not using this data for any aspect of model training, feature selection, or even optimization of the pre-processing pipeline, researchers are held accountable to a truly unseen evaluation. This process actively discourages conscious or unconscious decisions during model development that might incrementally bias the model towards apparent success on data that is, even if notionally separate, still part of the iterative development loop.

That said, it is a limitation of our work that our lock-box videos were still drawn from the same overall dataset. While this methodology strengthens claims about generalization to unseen samples from this specific source population and data acquisition context, true out-of-sample generalization across entirely different datasets, potentially varying in populations or recording conditions, is not something we can claim from this step alone. Testing on such completely independent external datasets is essential for that broader validation, and this is an objective we are actively working towards.

Feature selection based on clinician’s opinions: Our approach to feature selection, by relying on 'clinician-selected' general movement descriptors pre-defined and validated in prior research (e.g., Chambers et al., 2020), aims to minimize overfitting in a different way than data-driven feature selection might. Instead of applying algorithms to our current training data to identify features that maximally predict the outcome (a process that risks amplifying chance correlations specific to that sample), we used a set of features whose relevance is based on established clinical knowledge and prior empirical evidence. This reduces the likelihood that the chosen features are specifically tailored to our specific training sample's noise or particular distribution, thereby promoting a model based on more robust and generalizable movement characteristics.
Not using FMs: Our decision not to include features exclusively indicative of Fidgety Movements (FMs) relates more to our goal of developing a model with broader developmental applicability rather than directly minimizing statistical overfitting for the 3-4 month age window. FMs are powerful predictors within that specific timeframe. However, a model heavily reliant on FM-specific features would be inherently specialized to that narrow age range, limiting its utility for detecting risk at earlier or potentially later stages. By employing more general movement descriptors, our goal was to train a model that is not constrained to a single, age-specific biomarker.
This is a direction we are actively working towards by aggregating data from different ages (including term-age videos from our cohort) to uncover even earlier indicators of risk.

We can be confident that [...] it will generalize well to other datasets."
"...suggests that the model generalizes well to unseen data"
"We have shown [...] that our model generalizes well to unseen data."
There is not enough detail presented about the data sets to assess such statement. If, e.g., the camera angles of the used data sets are all similar, and the method is tested on a new video that was filmed from a completely different angle, I have doubts that it will actually generalize.

What we showed is that our model generalized to a sequestered set of data that had never been seen by the processing pipeline, so in that sense it did generalize well to unseen data.
That said, you’re absolutely correct that this statement is only true within a specific set of constraints. Camera angles specifically have a big impact on the quality of pose estimates, and many of the features we used in our feature vector (e.g., for measures of left/right symmetry). Ideally a pipeline would either have a more robust pre-processing approach (e.g., unwarping camera angles, or at least detecting them), features that are invariant to camera angle (e.g., learned through contrastive pretraining), or ideally some combination of the two.
We are in the process of testing this question at the moment on a separate dataset where videos were collected from 6 different camera angles and, as more datasets come online, we hope to continue re-training the model on increasingly large datestes that vary along many of these dimensions.

"If you can show that the datasets are very diverse and cover all/most possible settings, this will be more

convincing.
It would be interesting to check the results for such connections - e.g., if samples filmed from a specific angle are misclassified more often than others."

Both fortunately (and unfortunately), what we showed was that the samples were all filmed from a roughly top-down perspective. We analyzed the wingspan-to-body length ratio across all infants and the results were consistent with what would be expected for a top-down perspective (~0.78 ratio). This is slightly less than the anthropomorphically expected ratio of 1, but explained by the fact that these are 2D projections of 3D pose and infants very rarely extend their arms fully parallel to the ground. The perspective will therefore cause the 2D estimate of wingspan to be shorter than anticipated.

"The paper puts a lot of emphasis on the "lock-box" test set (mentioned 11 times in text and figure captions). Could you elaborate more on how this set is different from the main data set? If there is no real difference (same population, same camera angles, same distribution of other properties) and it is just a randomly selected subset of the main data set (keeping the GM label ratio), how is it different from simply being an additional cross-validation split?"

Yes, this is one of the elements of the pipeline that we feel is extremely important for methodological rigor. Unlike a cross-validation split, which is standard ML practice, a lock-box set goes one step further ensuring that no element of the pipeline (including consequential decisions like pose estimation algorithm, video pre-processing, etc) are optimized on the videos in the lock-box. So by showing that our process and ML algorithm generalize to the lock-box, we can be more confident that the process will work on other datasets as well. Due to the strict limitations of sharing infant data between clinical sites, we feel that the most direct way to allow others to test this claim is to release the full processing/inference pipeline so it can be tested on new data. We are currently in contact with a few different sites (notably the authors of Gao 2023) to perform out-of-sample testing in their data, and also test their MAM model on ours.

"It is mentioned that "the difference between infants whose movements are typically developing and those that are not is often subtle." - still, a binary classifier is used, which does not account for any subtle differences. I think it would be beneficial to aim for a more fine-grained classification, so that the prediction could also provide some kind of confidence level. This will be much more valuable to clinicians, to know which predictions can be really trusted, and which are uncertain."

Yes, we fully agree with that statement and it is the direction we plan to take for future work. This is a limitation of approaches that rely on GMA score prediction in general, and this has now been added to the discussion of the paper.
Testing whether using strict criteria with AutoML we could predict the two GMA score classes was just a first step towards the bigger goal of models that can be useful for global pre-screening (which could require different risk level assessments, not just binary classification), and development of decision support algorithms for clinicians (which would include things like confidence, and rationale for predictions).
Future work will also emphasize predicting scores that are more nuanced, for instance the HINE which relates not only to the presence/absence of fidgets but also to the quality and tone of movements, as well as the GMFM that gives an index of severity.

"Given that the clinician-selected movement features can predict GMA score in such a large sample, we have a strong indication that models that include even more data from a wider sample of infants, and more precise features, should perform even better."
Could you elaborate what you mean by "more precise features"? In literature, many people have used all kinds of features for nearly 20 years, and nobody has found the best combination of features, yet. It is not clear to me why more data should improve the results. Particularly if the data is from a lower-risk population."

Given that the clinician-selected movement features can predict GMA score moderately well in such a large sample, we have a strong indication that models leveraging even more data from a wider sample of infants, and incorporating more precise features, should perform significantly better.

Part of why we say this is that other groups have obtained much better results using, for instance, direct featurizations of fidgets. But mainly what we meant was achieving greater precision by moving beyond traditional feature engineering. It builds upon the growing body of evidence, across diverse domains, demonstrating that features learned directly from vast datasets via self-supervised learning (SSL) often outperform hand-curated counterparts.
The core idea is that by pre-training a model on large-scale, diverse infant movement data, including data from lower-risk or typically developing populations, across different contexts and camera angles etc, the SSL approach enables the model to learn a rich, nuanced understanding of general infant motor patterns. Then this pre-trained model can be fine-tuned on more specific, labeled datasets (e.g., infants with known outcomes) to detect subtle deviations indicative of CP risk.
'More precise features,' in this context, refer to these data-driven, latent representations that can capture complex patterns and subtle anomalies potentially missed by pre-defined, coarse kinematic measures. Our future work aims to adopt such self-supervised learning approaches to discover these more discriminative features, with the hypothesis that they will lead to more accurate and robust early CP risk assessment.
We have elaborated on this point in the discussion since, as you pointed out, our initial statement was much less nuanced.

"we find that our machine learning predictions are highly predictive of the clinician-assessed GMA (AUC=0.79). Given its low cost, our video-based approach may be useful for clinical screening applications..."
From Fig. 4, selecting a true positive rate of roughly 0.8 will give a false positive rate of roughly 0.25. Calculating from the numbers in the dataset (820 samples FMs, 105 samples absent FMs), the model predicts ~289 infants with absent FMs, of which 84 are true positives and 205 are false positives. This seems far from being usable in a screening application, particularly given that in a low-risk population the number of infants with absent FMs will be even lower."

We have done additional analysis looking at the trade-off between sensitivity and specificity for different thresholds, and also included a more thorough description of the precision-recall curve and limitations of the findings.
The false positive rate is, indeed, very high at the selected AUC threshold, and any clinically relevant tool would require careful consideration of the sensitivity/specificity trade off. We have added a section to the discussion presenting a more nuanced interpretation of these findings.

That said, our goal in this work was to not optimize for clinical utility but rather to develop a pipeline that is as likely as possible to work on other clinical datasets. The results of the classifier are moderate, which we feel is very good considering the constraints that were placed on feature selection and model training to prevent overfitting.
Other research groups have published work showing much higher precision/recall, but without a clear process for other researchers to validate and expand on their results. We feel that a model with medium precision/recall that is open is still a meaningful contribution to the field.

It's also worth noting that, in low-resource settings where infants do not have access to pediatric care, capturing the ~20% of infants that are at highest risk (i.e. minimizing false negatives) and referring them to clinical screening while reassuring ~80% of parents of high risk kids that they do not need additional screening would still be of value. The manuscript now discusses this nuance in greater depth.

"I think it would be interesting to dive deeper into the results analysis - do any specific patient characteristics correlate with the correctness of predictions? Or camera angles, recording length, etc... There is a lot more to find out, but this will be probably out of scope for this paper."

We agree wholeheartedly. More work is needed both to understand how different recording parameters and different patient characteristics relate to CP risk prediction, and whether nuanced patterns can help predict

not just CP as a whole, but also subtype and severity. We look forward to building on the results presented here, and hope that by releasing our code others will be able to advance their research as well.
Also, as mentioned previously, cross-site collaboration will be really important for this. There are many many features that may be relevant, but only small numbers of infants within each dataset for each of those features, and therefore a huge risk of overfitting if we try to look at all of them.

"Additional comments/questions
GMA standard recording length is recommended to be 3-5 minutes to my knowledge, why are only 1-2 minutes recorded? Is this considered enough by the clinicians to perform GMA?"

1-2 minutes is the length of time that clinicians needed to administer the GMA in this sample. Each video was evaluated by 2 reviewers (3 if the reviewers did not agree), and all of the videos included were deemed "usable" by both reviewers. Some videos were collected multiple times until the clinicians determined they were usable - we only analyzed the final video that was actually used for GMA administration.

"Fig. 4: Precision-Recall Curve is not mentioned in the text."

Apologies for the oversight, it has now been mentioned (and discussed) in the text.

"computed movement features have been made available on the OSF Pre-registration site." - Please add a link to the website where this can be found.

Absolutely: https://osf.io/gztmd/files/osfstorage

We have also updated the manuscript so it's easier for readers to find:

"Could you please state clearly the criteria for requests for the data set (keypoint data) to be considered as "reasonable"? The sentence "De-identified keypoint timeseries may be made available upon reasonable request." sounds like this is an arbitrary choice of the authors whether or not someone can get access. If the keypoint data is not really made available to the research community, this will greatly reduce the impact that this paper/data set can have for the field of automated CP detection."

Absolutely - this is now much clearer in the text. Not releasing the key points was not an arbitrary choice but rather a strict limitation imposed by the IRB. Keypoints are still considered personally identifying information and, as such, can only be analyzed by researchers that have been added to the IRB protocol. We've added a line to the manuscript explaining this more clearly in the spirit of full-transparency.

Additionally, we have released a set of features computed over a two-second sliding window for other researchers to be able to maximize the utility of this dataset for their own work (available here: https://osf.io/gztmd/files/osfstorage )

The inability to readily share/release data is an unsolved problem in the field as a whole, not just for our work. While our dataset is one of the largest, there are multiple research sites across the globe working to solve the same question – how can we detect CP from video – and all are actively trying to find a solution to aggregate data sources while respecting regulatory considerations and maintaining participant trust in the research groups.

We hope that by releasing code that is easy for researchers to use they will be able to build off of our work, and that this will help move the field forward even though we cannot openly release the keypoint dataset at this time.

We are also very interested in collaborating with other labs and working together to pool data using privacy preserving methods like federated learning.

"Review Conclusion
This paper can have a significant impact on research on automated CP detection. In my opinion, several changes need to be made.
My suggestion would be to put the focus of the paper on the data set that will be made available. This is a strong contribution and can have a big impact on the field. (I would also change the title accordingly.)
I would put less emphasis on the feature extraction and classification method. There have been many methods using hand-crafted features, and the results do not seem like a major breakthrough. The method can serve a good baseline for future comparisons, particularly because it is also open-sourced."

Thank you for the thorough review. We agree that the framing of the paper should be shifted and have modified the title to reflect it. We've taken a hybrid approach - emphasizing the dataset, but also specifically emphasizing that the methods we propose would facilitate data sharing.

The features we compute are certainly not the most precise, but they're easy to compute across datasets, and sufficiently de-identified that all researchers should be able to compute them and share them openly. We're actively working with other researchers to add more precise features (e.g., the fidget detection method from Gao et. al 2024) and plan to update the code and dataset as we do so.

Our goal is to allow for a rapid accumulation of feature datasets across the thousands of videos that the CP prediction community has access to in aggregate.
The AutoML method has a similar purpose - any researcher can train a model using this approach on their specific dataset easily and quickly to test for replication, or test the pre-trained model we released.

We're also looking for partners to implement federated learning to facilitate video-based approaches across sites.
Really our goal is to make it easier to share data and train models on as much of that data as possible, and this has now (hopefully) been made much clearer in the revised manuscript.

"In many parts, more detail needs to be added as mentioned above.
Overall, I think this paper can be an important step towards solving automated early detection of CP!"

We completely agree, and hope that the additional details provided help clarify why we cannot release the full keypoint dataset, but feel that providing the features, code, and AutoML model are still a meaningful contribution to the field.

em Editorial Manager®

## Cookie Preference Center

We use cookies which are necessary to make our site work. We may also use additional cookies to analyze, improve and personalize our content and your digital experience. For

more information, see our Cookie Policy.

You may choose not to allow some types of cookies. However, blocking some types may impact your experience of our site and the services we are able to offer. See the different category headings below to find out more or change your settings.

Allow all

## Manage Consent Preferences

### Strictly Necessary Cookies

**Always active**

These cookies are necessary for the website to function and cannot be switched off in our systems. They are usually only set in response to actions made by you which amount to a request for services, such as setting your privacy preferences, logging in or filling in forms. You can set your browser to block or alert you about these cookies, but some parts of the site will not then work.

### Functional Cookies

These cookies enable the website to provide enhanced functionality and personalisation. They may be set by us or by third party providers whose services we have added to our pages. If you do not allow these cookies then some or all of these services may not function properly.

### Performance Cookies

These cookies allow us to count visits and traffic sources so we can measure and improve the performance of our site. They help us to know which pages are the most and least popular and see how visitors move around the site.

### Targeting Cookies

These cookies may be set through our site by our advertising partners. They may be used by those companies to build a profile of your interests and show you relevant adverts on other sites. If you do not allow these cookies, you will experience less targeted advertising.

### Cookie List

Clear

Apply Cancel

Consent Leg.Interest

Confirm my choices

Power
