# Supplementary material for: A preregistered, open pipeline for early cerebral palsy risk assessment from infant videos
Source: Gigascience. 2026 Jan 20;15:giag003. doi: 10.1093/gigascience/giag003 (PMC13152017; doi:10.1093/gigascience/giag003)
Supplement: giag003_Authors_Response_To_Reviewer_Comments_revision_1 [file giag003_authors_response_to_reviewer_comments_revision_1.pdf]

Author's Response To Reviewer Comments

Dear reviewing editor,

Thank you for facilitating communication with Dr. Groos, who had major reservations about the manuscript. We hope that this version, that includes all of his requested analyses, addresses his concerns and the minor concerns of Dr. Hesse. Specifically we have:

1) Revised the language throughout the manuscript to avoid any misunderstandings about what we are claiming. Namely, that we are not claiming to achieve state-of-the-art results relative to deep learning methods, nor are we claiming to have produced a trained model that generalizes to other datasets. We released a comprehensive feature dataset, along with a pre-registered pipeline designed to prevent overfitting, and tested it using rigorous methods on our own data. We believe that this pipeline will generalize to other datasets, and have released everything open source in the hope that it will allow other researchers to test it and ideally release their own feature datasets to facilitate training across sites.

2) Benchmarked against the STAM model Dr. Groos requested during our email exchange. During review of the code, we identified a statistical error in the implementation which basically resulted in the model training on the test data. We have included the relevant details in our response to Dr. Groos, and in the revised manuscript.

We were not able to test our own model on a separate dataset because none of the authors we contacted, including Dr. Groos, were willing to do so. We will continue to seek out collaborators and hope to be able to publish work on out-of-sample generalizability in the future.

3) Added a section on interpretability, including permutation analyses to show the relative effects of permuting different features on the performance of this specific classifier. We have also included an additional scaling analysis to show how aggregating across larger datasets could improve model performance even with this simple feature set.

This manuscript has already sparked productive scientific debate regarding the value of different ML approaches, benchmarks, and features and we think it would be a valuable contribution to the field despite its limitations.

Sincerely,

Melanie (on behalf of all co-authors)

Reviewer 1: Nikolas Hesse:

Thank you for the new round of comments. You raised many interesting points, and we have integrated them into the newest version of the manuscript.

Comment 1: "Smoothing: The visualization in Fig 7 looks like a lot of information is lost and that it's likely that any fast motions will be smoothed out. This will most probably also affect the features and predictions. You write that you will most likely improve this in the future. Will the released features then be updated accordingly?"

Our goal is to continue releasing high-quality datasets to the greatest extent permitted by our IRB. Ideally this will include expanded feature sets, and features computed using different pre-processing approaches. We are actively seeking collaborators to expand this to include data from other sites, which will be crucial for developing robust algorithms that work across various conditions.

Comment 2: "A set of 38 kinematic features was selected based on clinician input [40, 41]," For references 40 and 41 it is unclear what kind of publications these are. Searching for them points to the OSF site, but there seems to be no additional information to that presented in the text regarding how the features are actually computed. Some details would be helpful, e.g., median velocity - is the velocity in each frame considered one measurement, and then the median value of all measurements is taken? This would not take into account non-movement periods. To understand this better, it would be good to have all the information on how the features are computed (without having to dig into the code)."

We agree and have added a few additional lines to the relevant section of the text clarifying how, specifically, the features were computed:

“All features were computed after smoothing the dataset, with frame-wise calculations of joint angles, velocities, and accelerations, which were then aggregated either over the entire video or within overlapping 2-second sliding windows (including rest periods).”

Comment 3: "The term „explainable" features (reminds me of „explainable AI"), means to me that it is possible to explain the reasoning why a test sample was scored the way it was. I don't mean to give overall importances for the separate features in the classifier, but explanations to each specific sample. E.g., infant sequence X is predicted as having absent FMs. Is it because of variability/complexity (entropy feature)? Or because of movement smoothness (IQR velocity)? Etc…
If it is possible to explain a classification like this, then the GM experts can go back and verify in the videos if these features actually capture what you assume they do. If such explanation is not possible, I would not use the term „explainable" in connection to the classification."

That’s a fair note on terminology and we’ve amended the manuscript accordingly. We have also included the feature importances for the classifier as a whole, as this was requested by another reviewer.

Our decision not to include features exclusively indicative of Fidgety Movements (FMs) relates more to our goal of developing a model with broader developmental applicability rather than directly minimizing statistical overfitting for the 3-4 month age window. FMs are powerful predictors within that specific timeframe. However, a model heavily reliant on FM-specific features would be inherently specialized to that narrow age range, limiting its utility for detecting risk at earlier or potentially later stages.

Comment 4: "You argue that the features are aimed at predicting broad developmental status. However, your clinical scores/class labels are „present FMs" vs. „absent FMs", and hence, your classifier is (supposedly) learning to predict the presence/absence of FMs. I don't think it's reasonable to assume that this approach will generalize to other age ranges/outcomes."

This is a fair argument, and we are working towards creating a labelled set of videos with fidgets that we can use for inclusion at the 3-4 month timepoint as well. The classifier is, as you said, likely already learning some proxy for fidgets since that is how this specific dataset is split. As such, the classifier itself is very unlikely to generalize beyond this age-range, but we are currently testing whether the same set of generic movement descriptors can also be used at different ages (term age) and also in other movement disorders. This would not be possible if we relied on the detection of fidgets as a core component of the pipeline.

The language in the manuscript has been clarified throughout to avoid misunderstandings.

Comment 5: "What we showed is that our model generalized to a sequestered set of data that had never been seen by the processing pipeline, so in that sense it did generalize well to unseen data."
If the data hadn't been "unseen" at test time, this would not be a valid evaluation. Since it is drawn from the same distribution as the training set, I would not make a bold claim on the model's generalization abilities.
I would consider the recordings done by parents at home to have at least some differences (even if the instructions were the same). So if the results on training with the data captured at the clinic and testing with parent videos is the same, this would convince me more that the model generalizes."

We completely agree. We spent several months trying to coordinate testing on separate dataset from a completely different distribution and so far have not been able to do so (sites either stopped responding, or declined to test the model on their data). What we can say for certain is that the full pipeline, from pose estimation to AutoML model training and evaluation, works end-to-end on any video dataset of people moving with any associated score that can be classified. The model itself likely won't generalize unless it's been trained across many datasets under many different conditions, which we really hope to be able to do soon, but the pipeline itself is robust and generalises well.

We've toned down the claims about generalizability so as to not overstate the result and rather emphasize the methods. Now reads: "We have shown that movement features derived from these pose estimates predict GMA scores in a very large sample, and that our model performs well on the lock-box test set"

Comment 6: "In the text, I would find it easier to read if GMA score 1/2/3 was replaced with something else, e.g. FMs present, FMs absent (, abnormal movements), or shorter FM-, FM+."

This is a great suggestion, we have revised throughout and agree it is much more readable.

Comment 7: "Not releasing the keypoints will definitely decrease the impact of the data set. I understand that this is not the author's choice, but I don't really understand how (normalized) keypoints are considered personally identifying information. Given the high barriers for getting access, I doubt that many people will actually be able to work with the keypoint data.
Gathering a large dataset of the (current) features doesn't seem like a promising avenue to me, due to the limitations that are also discussed in the paper/comments."

Based on the results of our scaling analysis (now included in the manuscript) aggregating even the current features across a sufficiently large population actually could yield classifiers that work at scale. This is a very low-effort means by which the community could facilitate dataset aggregation, and we hope to go adding feature computations to the pipeline as we develop them for our own data. Other sites sharing their feature computation code would certainly help accelerate this process for the community as a whole.

The sharing of keypoints is, indeed, a point of debate for IRBs. On the one hand, keypoints may not be identifiable in isolation. On the other hand, if the movement data is biometrically identifying (as we know it can be for gait), that may be a point of concern. In addition, if the dataset was collected within a specific region, it could result in situations like only one individual in the city having a specific subtype of cerebral palsy present in the dataset, thereby making it de-identifiable.

Comment 8: "Overall, I still think there are enough contributions that justify publication."

Thank you for all the suggestions and thorough review. It has certainly made the work stronger.

Reviewer 2: Daniel Groos
Thank you for taking the time to continue this discussion despite your reservations about the manuscript. We are sorry that the revisions did not sufficiently address your concerns, and hope that the newer version includes the information you were hoping to see in order to justify publication of the work.

Comment 1: "The authors have not succeeded to address major concerns of the paper, the most critical aspects including:

- Although expressing that the objective of the paper was to compare the accuracy of a simple machine learning method using few predefined features to more complex methods utilizing deep learning, the authors have not performed any experiments investigating this, neither by employing an existing state-of-the-art method on the proposed dataset nor by transferring the proposed method to a different dataset."

This was never the goal of the paper, and we apologize if the wording of any part of it implied as much. Our goal was to present a reproducible framework relying on 1) off-the-shelf pose estimation, 2) simple de-identified features, and 3) AutoML. We felt that there was a gap in the literature because other models

often relied on precise featurizations, that were not shared, using methods that may be overfit to the datasets on which they were trained. In the revised manuscript we make it clear that we do think deep-learned features can outperform the simple generic features we're using here but without making the code and features open for others there is no way to scale. To emphasise why this matters, we've included a scaling analysis showing that training on more data, even with simple features, improves classification according to a power-law relationship.

We are happy that you are asking for comparison to benchmarks and we have some unexpected results in this domain. While we can not benchmark against public data because they do not exist, we can run the published code of STAM against our code. On paper the STAM model is better, and when trained on our data the STAM code produces a result that is well better than ours (.86 AUC). However, analyzing their code revealed a fundamental statistical mistake, they do what is called record wise crossvalidation (see Saeb et al. 2017, The need to approximate the use-case in clinical machine learning) - in other words their training set contains snippets of data from the same babies that are in the test set. Correcting for this obvious statistical mistake their AUC drops to 0.6. Our code with the same crossvalidation strategy reaches AUC=.79. This shows how our code benchmarks very favourably against published results. It also highlights how the high standard of rigor we are pioneering in our work is essential.

Comment 2:
"- The authors continue to make bold claims that the proposed pipeline generalize to other datasets without having any evidence supporting this.
- The authors insist that 'the goal of our work was specifically to avoid predicting labeled fidgety movements' and yet there appears contradictory statements in the manuscript such as 'the model's output was a continuous probability score indicating the likelihood of absent fidgety movements'."

We've clarified in the manuscript that we didn't want the pipeline to rely on fidget labels so it could be more general-purpose. We likely did end up learning some proxy for fidgets because the training sample was split on fidgety present/absent labels, but that's unavoidable given the dataset we're using. We're actively working on 1) collecting a sample of videos with fidget labels so we can include them as computable features since they are highly indicative at 3-4 months and also 2) tracking long-term outcomes to be able to train for CP probability instead of FM probability, both at 3-4 months and earlier timepoints as well.

While our method does not outperform published SOTA results for automating the GMA, it outperforms the benchmark code you suggested, and until others have made their code available for running deep-learning based methods we feel that it is still a valuable methodological contribution to the field.

Comment 3:
"- The authors were specifically informed that interpretability should be highlighted as one of the major strengths of the proposed method compared to existing deep learning-based approaches. Nonetheless, the authors neglect to address this concern, and instead continue making statements without evidence such that 'if we try to interpret the top "n" features [..], they are not actually more important than the remaining features in aggregate.'"

While we continue to have reservations regarding the value of interpretability analyses, we have now included them in the revised manuscript and apologize for not doing so in the first place. Including the analyses and results with a discussion of their limitations is more useful for the scientific community than omitting them entirely. We hope you agree that productive debate about the value of different approaches is good for the field, and likely to result from this work.

Editorial Manager®

Cookie Preference Center

We use cookies which are necessary to make our site work. We may also use additional cookies to analyze, improve and personalize our content and your digital experience. For more information, see our Cookie Policy.

You may choose not to allow some types of cookies. However, blocking some types may impact your experience of our site and the services we are able to offer. See the different category headings below to find out more or change your settings.

Allow all

Manage Consent Preferences

Strictly Necessary Cookies

Always active

These cookies are necessary for the website to function and cannot be switched off in our systems. They are usually only set in response to actions made by you which amount to a request for services, such as setting your privacy preferences, logging in or filling in forms. You can set your browser to block or alert you about these cookies, but some parts of the site will not then work.

Functional Cookies

These cookies enable the website to provide enhanced functionality and personalisation. They may be set by us or by third party providers whose services we have added to our pages. If you do not allow these cookies then some or all of these services may not function properly.

Performance Cookies

These cookies allow us to count visits and traffic sources so we can measure and improve the performance of our site. They help us to know which pages are the most and least popular and see how visitors move around the site.

Targeting Cookies

These cookies may be set through our site by our advertising partners. They may be used by those companies to build a profile of your interests and show you relevant adverts on other sites. If you do not allow these cookies, you will experience less targeted advertising.

Cookie List

Clear

Apply Cancel

Consent Leg.Interest

Confirm my choices

Power
