# Supplementary material for: A preregistered, open pipeline for early cerebral palsy risk assessment from infant videos
Source: Gigascience. 2026 Jan 20;15:giag003. doi: 10.1093/gigascience/giag003 (PMC13152017; doi:10.1093/gigascience/giag003)
Supplement: giag003_Authors_Response_To_Reviewer_Comments_revision_2 [file giag003_authors_response_to_reviewer_comments_revision_2.pdf]

# Author's Response To Reviewer Comments

Dear Editorial Team,

Thank you for facilitating this most recent round of reviews and for the guidance throughout the process. We have carefully revised the manuscript and, to the best of our knowledge, addressed all remaining revisions.

In this revision, we have:
- Revised wording throughout to avoid vague or informal phrasing.
- Added additional references where appropriate to support key claims.
- Updated and corrected all figures, captions, and legends.
- Restructured sections to improve logical flow and ensure alignment with the journal's guidelines.
- Corrected remaining grammatical and typographic issues.

In response to the reviewer's comments regarding acknowledgment of prior work, we have substantially revised the relevant section to more clearly and accurately describe the use of external validation sets in this literature.
We appreciate the reviewer's thoughtful feedback, which has improved the clarity and precision of the manuscript.

Thank you again for your time and guidance.

Sincerely,

Melanie Segado (on behalf of all co-authors)

– Direct Response to Reviewer #1 –

Thank you for the detailed and constructive feedback. We have carefully revised the manuscript to address all of the points raised, with particular attention to clarifying terminology, improving tone and accuracy, and refining the figures, tables, and accompanying descriptions.

1. Reviewer: "If these relevant movement features can be reliably computed from videos, then algorithmic approaches for predicting infant risk from movement features should perform robustly."
This sentence should be made more neutral, e.g., "…this indicates potential for algorithmic approaches..."

Response: Thank you for this helpful suggestion. The sentence has been revised to:
"If these relevant movement features can be reliably computed from videos, it suggests that algorithmic approaches could feasibly be applied to estimate infant risk from movement features."

This phrasing is more neutral, and written with a more suitable academic tone.

2. Reviewer: "However, they are not readily available for testing on new data, have limited generalizability, and use methods that may yield overly optimistic performance estimates."
The authors should strive to support such statements with references to literature review papers.

Response: We agree that this statement should be supported by the existing methodological literature. We have therefore added references to work showing how non-independent data splits and flexible model development pipelines can yield overly optimistic performance estimates (Saeb et al., 2017), as well as work on pipeline overfitting and "trying a bunch of things" that specifically motivated our use of a preregistered lock-box evaluation (Hosseini et al., 2020).

To clarify what we meant by models being "not readily available," we have added a short explanation in the manuscript based on our experience attempting to perform external replication. Several studies in this area

state that code or data are available upon request. In practice, however, full access to model artifacts can still be difficult. For example, in the case of Gao et al., we contacted the authors multiple times over several months to request the trained model and curated dataset needed for external evaluation. These requests did not result in access, which prevented us from applying their model to our cohort. We fully recognize that there may be administrative, ethical, or logistical reasons that limit responses to such requests, and we do not intend this observation as a criticism of the authors. Our point is simply that, from the perspective of an independent research group, the absence of accessible model artifacts can make external validation challenging, even when studies describe their code or data as available upon request.

We hope this clarification explains why we noted that prior models are not always straightforwardly deployable for independent external testing.

3. Reviewer: "Additionally, while all adhered to standard ML methods (such as external validation sets), none employed a stricter lock-box set (i.e., held-out data points that were not used at any point during the hyperparameter optimization process, and publicly pre-registered), raising the possibility that results may be overly optimistic [30] due to iterative optimization of the analysis pipeline as a whole."
This statement is incorrect and should be significantly rephrased or removed. Although many related studies do not use held-out lock-box/external validation/test set and often are limited to results from leave-one-out or k-fold cross-validation, there exists studies (including studies referenced in the manuscript) that follow similar strict evaluation by testing on a dataset that is separate from development (i.e., training and validation) data (e.g., different set of infants) and that has been isolated from development (including hyperparameter optimization) and used for testing purposes and reporting only. In the current state of the manuscript, the authors seem to be unaware of or neglect this fact. To satisfy the reviewer, the authors need to carefully revise the manuscript to reflect the objective truth.

Response: Thank you for this important clarification. We agree that our original wording did not clearly distinguish between external validation and the more specific preregistered lock-box framework we intended to describe. We also agree that this ambiguity could be read as implying that earlier studies did not use fully independent test data. This was not our intention, and we apologize for the error.

As you note, several prior studies, including Gao et al. and others, evaluated their models on cohorts that were completely held out from training and hyperparameter optimization (the statement in our previous draft has been fully removed). These studies follow established best practices for rigorous model evaluation, and we have revised the manuscript to clearly acknowledge and credit this work.

The distinction we aim to draw is narrower and relates specifically to prospective preregistration. In our study, the evaluation dataset was specified in advance of any model development. The test set was therefore not only independent but also defined prospectively, with the preprocessing steps, model choices, and code frozen and publicly pre-registered before any evaluation. You are correct that it makes more sense to describe this as an extension of existing external validation practices rather than a contrast with them.

We revised the paragraph discussing prior work and our lock box design to more clearly acknowledge that several earlier studies used fully held out external test cohorts, and that in practice they may have not even looked at their test set more than once. The new text explicitly credits these studies for following standard machine learning evaluation practices and removes any phrasing that could be interpreted as suggesting otherwise.

We further clarified that our preregistered lock-box is intended as an extension of these practices. The revised paragraph now explains that the lock-box was prospectively specified, evaluated only once, and paired with a preregistered preprocessing and analysis plan. We also emphasized that this distinguishes our approach by adding preregistration and prospective specification, rather than by implying a lack of rigor in prior work.
Finally, we added language noting that preregistered, prospectively isolated test sets are still relatively uncommon in this literature and that we provide our implementation, including code and data, for others to validate the result and test on their own datasets.

4. Reviewer: Mention in the caption what T and H is referring to.
Response: Thank you for this helpful suggestion. We have added explicit definitions of pT−V and pT−T in the Table 1 caption, indicating comparisons between Train vs. Validation and Train vs. Test (lock-box) sets, respectively. This is in accordance with the new naming convention we have adopted to better align with published conventions.
5. Reviewer: Adhere to standard machine learning terminology and write validation instead of test set for the AutoML dataset throughout figure, figure caption and manuscript text. The lock-box set can alternatively be referred to as test set.

Response: Thank you for this suggestion; we agree that clear and standardized terminology is essential. In line with your recommendation, we have revised the figures, figure captions, and manuscript text to use Train / Validation / Test (lock-box) consistently. Specifically, we now refer to the AutoML evaluation dataset as the Validation set, and to the preregistered lock-box as the Test (lock-box) set. We will update the filenames on OSF, and our code, to reflect this.

In our implementation, Auto-sklearn performs internal validation through successive halving and cross-validation within the training data during model selection and hyperparameter optimization. On top of this, the Validation set is held out from training and used for performance estimation and model comparison during development.
By contrast, the Test (lock-box) set is completely isolated from the outset: its participant IDs, preprocessing pipeline, and analysis plan are preregistered in advance, and it is evaluated only once after all preprocessing decisions and modeling choices, including the final trained model, have been finalized and preregistered. This design adheres to standard ML terminology while adding a preregistered lock-box layer that, we believe, improves transparency.

6. Reviewer: "…ViTPose-H [19] performed better than the alternatives…"
Specify instead "…pretrained ViTPose-H [19] performed better than the fine-tuned alternatives..."

Response: Thank you for pointing out this potential point of confusion. We have revised the sentence to read:
"We found that the pretrained ViTPose-H performed better than other pretrained models (HRNet, PVTv2) and our previously fine-tuned OpenPose model."
This revision clarifies that only OpenPose was fine-tuned on our prior infant dataset, while HRNet and PVTv2 were evaluated in their pretrained (COCO-trained) forms.

We also added a brief explanation that our methodological goal was to evaluate off-the-shelf models likely to generalize across datasets without requiring fine-tuning, in keeping with the reproducibility and scalability aims of the project. However, we acknowledge that fine-tuned infant-specific models are increasingly becoming available, and we plan to benchmark ViTPose-H against these newly released fine-tuned versions in future work. Should such models demonstrate equal or superior performance, they can be readily adopted within the same open-source pipeline we provide.

7. Reviewer: The three subsections "Benchmarking against deep learning-models", "Scaling analysis", and "Explainability and feature importance" describe features and predictive models and should be moved to an associated manuscript section(s) (i.e., they are misplaced in the section "Developing a pipeline for robust skeletal tracking").

Response: We have moved these subsections into Model Performance and Validation for better logical structure and consistency with the paper's organization.

8. Figure 4 Reviewer: It would be more intuitive to have higher AUC with increased value of y-axis.

Response: We absolutely agree with this point. The figure has been modified accordingly, with the y-axis orientation adjusted so that higher AUC values correspond to higher y values. This change makes the figure much easier to interpret at a glance and aligns the figure with standard conventions for performance curves.

9. Figure 7 Reviewer: "A decision threshold was selected to achieve a TPR of 80% on the holdout set." This suggests that the threshold was set to optimize performance on the lock-box set and contradicts the authors' rationale that all decisions were made prior to testing on the lock-box set. Mention this as a limitation and also include performance on the lock-box set if the threshold instead was optimized on the training/validation data.

Response: Thank you for raising this important point. We agree that our original phrasing could be interpreted as selecting the decision threshold based on the lock box data, which would contradict the preregistered one-time evaluation design. We apologize for this ambiguity and have revised the manuscript to prevent this interpretation.
In our preregistered plan, we committed only to computing ROC curves on the lock box in order to characterize performance across thresholds, and we did not preregister the selection of any specific operating point. The confusion matrix shown in Figure 7 corresponds to a point on the lock box ROC curve that was requested by another reviewer, and is therefore presented only as an illustration of possible operating characteristics rather than as a preregistered threshold.

To address your concern, we now explicitly describe this figure as illustrative, and we have added a clear statement explaining that selecting a threshold after inspecting the lock box ROC curve can introduce optimistic bias. We have also added the performance obtained when the threshold is chosen using the training and validation data instead. This revision makes it explicit that threshold selection was not part of the preregistered plan and that the lock box was not used to optimize classification decisions. In future work we will aim to select a threshold prior to evaluation as an additional step towards generalizability.

We hope this resolves the concern and improves clarity regarding the preregistered evaluation design.

10. Reviewer: "However, under strict 'vanilla' settings, training time is restricted to 1 hour and the ensemble size restricted to one [41]. This constraint ensures a highly rigorous evaluation of individual models during the search phase. This approach increases the likelihood that the selected classifiers will generalize effectively to new, unseen data..."

This statement is counterintuitive as an ensemble of (several) machine learning models usually improves robustness attainable by individual models, by ensembles being less sensitive to noise that often negatively influences individual models.

Response: We agree and have rephrased to acknowledge that while ensembles can improve robustness, repeated validation-based adaptation in AutoML can induce overfitting. We clarify that single-model training was used to produce conservative results.

Overall:

Thank you again for your careful and thoughtful feedback. The suggested changes have strengthened the manuscript’s accuracy, clarity, and consistency. We have implemented every requested modification, including the grammar and consistency errors, and hope the manuscript now fully addresses all remaining concerns.

## Cookie Preference Center

We use cookies which are necessary to make our site work. We may also use additional cookies to analyze, improve and personalize our content and your digital experience. For more information, see our Cookie Policy.

You may choose not to allow some types of cookies. However, blocking some types may impact your experience of our site and the services we are able to offer. See the different category headings below to find out more or change your settings.

Allow all

## Manage Consent Preferences

### Strictly Necessary Cookies

**Always active**

These cookies are necessary for the website to function and cannot be switched off in our systems. They are usually only set in response to actions made by you which amount to a request for services, such as setting your privacy preferences, logging in or filling in forms. You can set your browser to block or alert you about these cookies, but some parts of the site will not then work.

### Functional Cookies

These cookies enable the website to provide enhanced functionality and personalisation. They may be set by us or by third party providers whose services we have added to our pages. If you do not allow these cookies then some or all of these services may not function properly.

### Performance Cookies

These cookies allow us to count visits and traffic sources so we can measure and improve the performance of our site. They help us to know which pages are the most and least popular and see how visitors move around the site.

**Targeting Cookies**

These cookies may be set through our site by our advertising partners. They may be used by those companies to build a profile of your interests and show you relevant adverts on other sites. If you do not allow these cookies, you will experience less targeted advertising.

**Cookie List**

Clear

Apply Cancel

Consent Leg.Interest

Confirm my choices

Power
