# Supplementary material for: A preregistered, open pipeline for early cerebral palsy risk assessment from infant videos
Source: Gigascience. 2026 Jan 20;15:giag003. doi: 10.1093/gigascience/giag003 (PMC13152017; doi:10.1093/gigascience/giag003)
Supplement: giag003_Reviewer_1_Report_original_submission — Reviewer 1 -- 1/26/2025 [file giag003_reviewer_1_report_original_submission.pdf]

Reviewer Report

Title: A Pre-Registered, Open Pipeline for Early Cerebral Palsy Risk Assessment from Infant Videos

Version: Original Submission **Date:** 1/26/2025

Reviewer name: Reviewer 1

Reviewer Comments to Author:

The paper is targeting early CP prediction by proposing an automatic method for assessing infant risk from videos using a high-risk cohort of 1 060 infants. The method combines video-based pose estimation with predefined feature extraction and classification of movement pattern through automated GMA. By focusing on material related to my competencies within machine learning for movement analysis and CP prediction, I have the following remarks which the authors should carefully pay attention to in order for the paper to be worthy of publication:

- General

o The authors have focused on developing a simple yet automatic method for detecting fidgety movements that generalizes beyond the training and validation data by relying on a small set of 38 kinematic features derived from video-based pose estimation. This inherently restricts the level of predictive accuracy that can be achieved by the method but at the same time could make the classifier a lightweight, interpretable alternative to high-accuracy deep learning approaches for automated GMA. However, the authors have not currently focused on the interpretability of the method, and hence major revision is necessary for this request to be addressed. The desired outcome would be that the paper illustrates how different kinematic features contribute to the prediction of present or absent fidgety movements through the use of feature importance values provided by the sklearn classification model.

o The authors repeatedly make incorrect claims and bold statements without support from results or literature. This concern needs to be properly addressed (see further comments in associated sections below).

- Abstract

o Â«lock-boxÂ» set: Use standard terminology (test set or external validation set)

- Background

o "The combined advancements in deep learning, open datasets, and open-source tools have significantly improved the reliability and accuracy of pose estimation and tracking outcomes[24, 25].": This statement should be supported by scientific references, not GitHub links

o "Pretrained vision transformers should be sufficiently good to capture the movement features that are relevant for clinical assessment, without the need for custom models that risk overfitting.": Refrain from such statements without references or other evidence

o "This approach was highly effective at detecting FMs, however, as they noted this approach cannot be extended without the need for retraining on other hand-labeled segments.": Elaborate on this and inform the reviewer about where in the paper (reference 6) this information can be found

o "[…] none employed a "lock-box" set (ie. held-out data points that were not used at any point during

the hyperparameter optimization process), raising the possibility that results are overly optimistic [28].": This statement is wrong, there are previous studies that have used a hold-out test set/external validation set for final evaluation (one such example is the study of Gao et al. that the authors refer to in reference 6). The authors should be overly careful when making such claims.
o "To compute accurate movement features, we started by selecting an open-source pose estimation algorithm that had high precision on our infant dataset.": How did the authors verify this? There are no results in the paper backing such a claim.
o "We show that movement features can predict GMA scores in the largest infant dataset used to date": Once again the authors make an incorrect claim (the study of Gao et al. (reference 6) used a larger sample), indicating that they do not have sufficient overview of related studies and even the references they have included.
- Patient characteristics
o Lack statistics on risk factors in the sample of recruited infants
- Inclusion/Exclusion Criteria
o The authors should include a flow diagram that more intuitively illustrates the process of inclusion/exclusion per criterion (including the number of infants excluded at each stage)
o "The remaining 931 videos were split into an analysis set (744) and a lock box holdout set (187). The analysis set was further split into train/val/test sets (558, 93, 93), each of which had a 12% representation of the "absent fidgety" movement type.": Currently, there is confusion related to the datasets in the paper, especially the use of three dataset splits, "train", "val" and "test", in addition to the "lock-box" set. What makes it even more confusing is that "train" and "val" are together referred to as "training set" (e.g., Figure 1). The reviewer proposes to stick with common ML methodology and use three dataset portions, "train" (651), "validation" (93) and "test" (187), with the last being the "lock-box" set.
- Analysis
o "Infant videos pose unique challenges for pose-estimation algorithms due to frequent irregular body poses, the presence of body-like objects (e.g., toys or cartoons), and high levels of self-occlusion.": The authors should also mention the different anatomical proportions of infants compared to adults, and refer to the study of Sciortino et al. (https://link.springer.com/chapter/10.1007/978-3-319-68548-9_38).
o "Conventional algorithms, such as OpenPose[29], often fail in such conditions, leading to unreliable pose estimates [22]. Fine-tuning is often required to improve accuracy on each infant dataset.": Lack of infant-specific references, e.g., https://www.sciencedirect.com/science/article/pii/S0895611121001610.
o "However, we found that a pre-trained vision transformer, ViTPose-H [19], performed better than fine-tuned alternatives [30, 31, 32, 29, 33], obviating the need for manual finetuning.": How was this evaluated? There does not seem to be any results in the paper comparing the accuracy of ViTPose-H to a fine-tuned pose estimation model. Currently, there is only a single example image in Figure 2. For the authors to claim that pre-trained ViTPose-H is better than fine-tuned alternatives, this should be supported by quantitative evidence on a sufficiently large sample of infant images/videos. Furthermore, the fine-tuning procedure of OpenPose is not mentioned in the text and as an outdated method OpenPose is not representative of the performance that could be achieved through fine-tuning. Hence, it is better to avoid such statements to avoid confusion and invalid conclusions.
o "A set of 38 kinematic features was selected based on clinician input [34, 35], designed to capture the

displacement, speed, velocity, acceleration, and entropy of key body parts, specificallywrists, ankles, elbows, and knees (Figure 3).": The authors should provide a list of the specific kinematic features along with their biomechanical definitions.

o Figure 3 is way too small for information to be consumed by the user.

o "Testing on the lockbox set yielded an AUC-ROC of 0.79 (Figure 4), closely aligned with the cross-validation performance, indicating minimal overfitting.": While the AUC on the test set indicates that the model does not overfit on the training and validation data it should still be addressed that the AUC is still moderate (for example, compared to the study of Gao et al.). However, the authors could argue for the interpretability of the proposed method, but this should then be backed by results on feature importance of the selected set of biomechanical features.

- Discussion

o "We used an exceptionally big sample (training set: 558, overall >1000 infants)": The authors should stay objective and avoid such exaggeration

o "We found that our algorithm performs well (AUC-ROC 0.79).": Such a statement should relate to the accuracy achieved in other studies on automated GMA, e.g., https://www.nature.com/articles/s41467-023-44141-x.

o "Based on our rigorous pre-registered approach with a lock-box set we can be confident that we did not do any overfitting and that it will generalize well to other datasets.": This is a bold statement, since the model will not generalize beyond the characteristics of the dataset used for training.

o "The clinician-selected movement features offer only a coarse description of movement, whereas we know from the clinical literature that the difference between infants whose movements are typically developing and those that are not is often subtle.": The authors should include references that support such a claim.

o "Given that the clinician-selected movement features can predict GMA score in such a large sample, we have a strong indication that models that include even more data from a wider sample of infants, and more precise features, should perform even better.": How can you be sure about this?

- Potential implications

o "Overall we have shown that advances in pose estimation now make it entirely realistic to get precise movements from infant videos without the need for any specialized camera setup or finetuning.": How can you be such confident without any results on the preciseness of pose estimation?

- Methods

o "Model selection and hyperparameter optimization were carried out using the Auto-sklearn 2.0 package [41, 42], with the "vanilla Autosklearn" setting.": The authors should mention which specific machine learning models and hyperparameters that were explored using auto-sklearn.

- References

o Figure 2 not referred to in main manuscript

o 27 and 28 seem to be duplicate references

- Typos

o Lack of brackets and parenthesis

ï,§ E.g., references 6 and 25 mentioned without brackets

ï,§ "(typically wearing a diaper only"

o "Lage" in Figure 1

Methods

Are the methods appropriate to the aims of the study, are they well described, and are necessary controls included? Choose an item.

Conclusions

Are the conclusions adequately supported by the data shown? Choose an item.

Reporting Standards

Does the manuscript adhere to the journal's guidelines on minimum standards of reporting? Choose an item.

Choose an item.

Statistics

Are you able to assess all statistics in the manuscript, including the appropriateness of statistical tests used? Choose an item.

Quality of Written English

Please indicate the quality of language in the manuscript: Choose an item.

Declaration of Competing Interests

Please complete a declaration of competing interests, considering the following questions:

- Have you in the past five years received reimbursements, fees, funding, or salary from an organisation that may in any way gain or lose financially from the publication of this manuscript, either now or in the future?
- Do you hold any stocks or shares in an organisation that may in any way gain or lose financially from the publication of this manuscript, either now or in the future?
- Do you hold or are you currently applying for any patents relating to the content of the manuscript?
- Have you received reimbursements, fees, funding, or salary from an organization that holds or has applied for patents relating to the content of the manuscript?
- Do you have any other financial competing interests?
- Do you have any non-financial competing interests in relation to this paper?

If you can answer no to all of the above, write 'I declare that I have no competing interests' below. If your reply is yes to any, please give details below.

I declare that I have no competing interests

I agree to the open peer review policy of the journal. I understand that my name will be included on my report to the authors and, if the manuscript is accepted for publication, my named report including any attachments I upload will be posted on the website along with the authors' responses. I agree for my report to be made available under an Open Access Creative Commons CC-BY license (http://creativecommons.org/licenses/by/4.0/). I understand that any comments which I do not wish to be included in my named report can be included as confidential comments to the editors, which will not be published.

Choose an item.

To further support our reviewers, we have joined with Publons, where you can gain additional credit to further highlight your hard work (see: https://publons.com/journal/530/gigascience). On publication of this paper, your review will be automatically added to Publons, you can then choose whether or not to claim your Publons credit. I understand this statement.

Yes Choose an item.
