# Supplementary material for: A preregistered, open pipeline for early cerebral palsy risk assessment from infant videos
Source: Gigascience. 2026 Jan 20;15:giag003. doi: 10.1093/gigascience/giag003 (PMC13152017; doi:10.1093/gigascience/giag003)
Supplement: giag003_Reviewer_1_Report_revision_1 — Reviewer 1 -- 6/9/2025 [file giag003_reviewer_1_report_revision_1.pdf]

Reviewer Report

Title: A Pre-Registered, Open Pipeline for Early Cerebral Palsy Risk Assessment from Infant Videos

Version: Revision 1 **Date:** 6/9/2025

Reviewer name: Reviewer 1

Reviewer Comments to Author:

The authors have not succeeded to address major concerns of the paper, the most critical aspects including:

- Although expressing that the objective of the paper was to compare the accuracy of a simple machine learning method using few predefined features to more complex methods utilizing deep learning, the authors have not performed any experiments investigating this, neither by employing an existing state-of-the-art method on the proposed dataset nor by transferring the proposed method to a different dataset.
- The authors continue to make bold claims that the proposed pipeline generalize to other datasets without having any evidence supporting this.
- The authors insist that 'the goal of our work was specifically to avoid predicting labeled fidgety movements' and yet there appears contradictory statements in the manuscript such as 'the model's output was a continuous probability score indicating the likelihood of absent fidgety movements'.
- The authors were specifically informed that interpretability should be highlighted as one of the major strengths of the proposed method compared to existing deep learning-based approaches. Nonetheless, the authors neglect to address this concern, and instead continue making statements without evidence such that 'if we try to interpret the top "n" features [..], they are not actually more important than the remaining features in aggregate.'

Methods

Are the methods appropriate to the aims of the study, are they well described, and are necessary controls included? Choose an item.

Conclusions

Are the conclusions adequately supported by the data shown? Choose an item.

Reporting Standards

Does the manuscript adhere to the journal’s guidelines on minimum standards of reporting? Choose an item.

Choose an item.

Statistics

Are you able to assess all statistics in the manuscript, including the appropriateness of statistical tests used? Choose an item.

Quality of Written English

Please indicate the quality of language in the manuscript: Choose an item.

Declaration of Competing Interests

Please complete a declaration of competing interests, considering the following questions:

- Have you in the past five years received reimbursements, fees, funding, or salary from an organisation that may in any way gain or lose financially from the publication of this manuscript, either now or in the future?
- Do you hold any stocks or shares in an organisation that may in any way gain or lose financially from the publication of this manuscript, either now or in the future?
- Do you hold or are you currently applying for any patents relating to the content of the manuscript?
- Have you received reimbursements, fees, funding, or salary from an organization that holds or has applied for patents relating to the content of the manuscript?
- Do you have any other financial competing interests?
- Do you have any non-financial competing interests in relation to this paper?

If you can answer no to all of the above, write 'I declare that I have no competing interests' below. If your reply is yes to any, please give details below.

I declare that I have no competing interests

I agree to the open peer review policy of the journal. I understand that my name will be included on my report to the authors and, if the manuscript is accepted for publication, my named report including any attachments I upload will be posted on the website along with the authors' responses. I agree for my report to be made available under an Open Access Creative Commons CC-BY license (http://creativecommons.org/licenses/by/4.0/). I understand that any comments which I do not wish to be included in my named report can be included as confidential comments to the editors, which will not be published.

Choose an item.

To further support our reviewers, we have joined with Publons, where you can gain additional credit to further highlight your hard work (see: https://publons.com/journal/530/gigascience). On publication of this paper, your review will be automatically added to Publons, you can then choose whether or not to claim your Publons credit. I understand this statement.

Yes Choose an item.
