# Supplementary material for: A preregistered, open pipeline for early cerebral palsy risk assessment from infant videos
Source: Gigascience. 2026 Jan 20;15:giag003. doi: 10.1093/gigascience/giag003 (PMC13152017; doi:10.1093/gigascience/giag003)
Supplement: giag003_Reviewer_1_Report_revision_2 — Reviewer 1 -- 10/26/2025 [file giag003_reviewer_1_report_revision_2.pdf]

Reviewer Report

Title: A Pre-Registered, Open Pipeline for Early Cerebral Palsy Risk Assessment from Infant Videos

Version: Revision 2 **Date:** 10/26/2025

Reviewer name: Reviewer 1

Reviewer Comments to Author:

In the revised manuscript version, the authors have addressed the reviewer's main concerns. If the authors appropriately refine the manuscript further according to the following minor considerations, the reviewer is positive for publication:

- Background (p. 2): "If these relevant movement features can be reliably computed from videos, then algorithmic approaches for predicting infant risk from movement features should perform robustly." This sentence should be made more neutral, e.g., "…this indicates potential for algorithmic approaches..."
- Background (p. 2): "However, they are not readily available for testing on new data, have limited generalizability, and use methods that may yield overly optimistic performance estimates." The authors should strive to support such statements with references to literature review papers.
- Background (p. 2): "Additionally, while all adhered to standard ML methods (such as external validation sets), none employed a stricter lock-box set (i.e., held-out data points that were not used at any point during the hyperparameter optimization process, and publicly pre-registered), raising the possibility that results may be overly optimistic [30] due to iterative optimization of the analysis pipeline as a whole." This statement is incorrect and should be significantly rephrased or removed. Although many related studies do not use held-out lock-box/external validation/test set and often are limited to results from leave-one-out or k-fold cross-validation, there exists studies (including studies referenced in the manuscript) that follow similar strict evaluation by testing on a dataset that is separate from development (i.e., training and validation) data (e.g., different set of infants) and that has been isolated from development (including hyperparameter optimization) and used for testing purposes and reporting only. In the current state of the manuscript, the authors seem to be unaware of or neglect this fact. To satisfy the reviewer, the authors need to carefully revise the manuscript to reflect the objective truth.
- Table 1 (p. 4): Mention in the caption what T and H is referring to.
- Figure 2 (p. 4): Adhere to standard machine learning terminology and write validation instead of test set for the Auto-ML dataset throughout figure, figure caption and manuscript text. The lock-box set can alternatively be referred to as test set.
- Analyses (p. 5): "…ViTPose-H [19] performed better than the alternatives…" Specify instead "…pretrained ViTPose-H [19] performed better than the fine-tuned alternatives..."
- Analyses (p. 5): The three subsections "Benchmarking against deep learning-models", "Scaling analysis" and "Explainability and feature importance" describe features and predictive models and should be moved to an associated manuscript section(s) (i.e., they are misplaced in the section "Developing a pipeline for robust skeletal tracking")
- Figure 4 (p. 6): It would be more intuitive to have higher AUC with increased value of y-axis.

- Figure 7 (p. 7): "A decision threshold was selected to achieve a TPR of 80% on the holdout set." This suggests that the threshold was set to optimize performance on the lock-box set and contradicts the authors' rationale that all decisions were made prior to testing on the lock-box set. Mention this as a limitation and also include performance on the lock-box set if the threshold instead was optimized on the training/validation data.
- Analyses (p. 7): "However, under strict "vanilla" settings, training time is restricted to 1 hour and the ensemble size restricted to one [41]. This constraint ensures a highly rigorous evaluation of individual models during the search phase. This approach increases the likelihood that the selected classifiers will generalize effectively to new, unseen data..." This statement is counterintuitive as an ensemble of (several) machine learning models usually improves robustness attainable by individual models, by ensembles being less sensitive to noise that often negatively influences individual models.
Additionally, the authors should review the paper for inconsistencies and writing errors (-word(s)- indicates insert, :word(s): indicates remove), for example on page 4 "Six infants -with- atypical FM were excluded…", on page 5 "making it difficult for researchers without the time and technical skills :to do so unable :to benefit from custom models." and "We took the complete feature dataset and split it into training set sizes of 50, 100, 200, 400, and 800 -infants-…", in caption of Figure 8 on page 8 "Classifier trained on clinician-selected features using vanilla Auto-sklearn 2.0 shows a high ROC-AUC of 0.79 (-Top- :Left:) and Precision-Recall of 0.34 (-Bottom-:Right:) on lock-box set of 187 infants…" and on page 10 "…ensuring a clear separation between training, validation, :and test datasets, :and the lock-box test set."

Methods

Are the methods appropriate to the aims of the study, are they well described, and are necessary controls included? Choose an item.

Conclusions

Are the conclusions adequately supported by the data shown? Choose an item.

Reporting Standards

Does the manuscript adhere to the journal's guidelines on [minimum standards of reporting?]() Choose an item.

Choose an item.

Statistics

Are you able to assess all statistics in the manuscript, including the appropriateness of statistical tests used? Choose an item.

Quality of Written English

Please indicate the quality of language in the manuscript: Choose an item.

Declaration of Competing Interests

Please complete a declaration of competing interests, considering the following questions:

- Have you in the past five years received reimbursements, fees, funding, or salary from an organisation that may in any way gain or lose financially from the publication of this manuscript, either now or in the future?
- Do you hold any stocks or shares in an organisation that may in any way gain or lose financially from the publication of this manuscript, either now or in the future?
- Do you hold or are you currently applying for any patents relating to the content of the manuscript?
- Have you received reimbursements, fees, funding, or salary from an organization that holds or has applied for patents relating to the content of the manuscript?
- Do you have any other financial competing interests?
- Do you have any non-financial competing interests in relation to this paper?

If you can answer no to all of the above, write 'I declare that I have no competing interests' below. If your reply is yes to any, please give details below.

I declare that I have no competing interests

I agree to the open peer review policy of the journal. I understand that my name will be included on my report to the authors and, if the manuscript is accepted for publication, my named report including any attachments I upload will be posted on the website along with the authors' responses. I agree for my report to be made available under an Open Access Creative Commons CC-BY license (http://creativecommons.org/licenses/by/4.0/). I understand that any comments which I do not wish to be included in my named report can be included as confidential comments to the editors, which will not be published.

Choose an item.

To further support our reviewers, we have joined with Publons, where you can gain additional credit to further highlight your hard work (see: https://publons.com/journal/530/gigascience). On publication of this paper, your review will be automatically added to Publons, you can then choose whether or not to claim your Publons credit. I understand this statement.

Yes Choose an item.
