# Supplementary material for: A preregistered, open pipeline for early cerebral palsy risk assessment from infant videos
Source: Gigascience. 2026 Jan 20;15:giag003. doi: 10.1093/gigascience/giag003 (PMC13152017; doi:10.1093/gigascience/giag003)
Supplement: giag003_Reviewer_2_Report_original_submission — Reviewer 2 -- 2/3/2025 [file giag003_reviewer_2_report_original_submission.pdf]

Reviewer Report

Title: A Pre-Registered, Open Pipeline for Early Cerebral Palsy Risk Assessment from Infant Videos

Version: Original Submission **Date:** 2/3/2025

Reviewer name: Reviewer 2

Reviewer Comments to Author:

Review: "Assessing infant risk of cerebral palsy with video-based motion tracking".
The paper describes a pipeline to automatically classify movements based on the General Movement Assessment (GMA) in a relative large dataset (931 infants after application of exclusion criteria; including 105 infants with a positive score on GMA i.e. absence of fidgety movements). This paper contributes to the innovation of the recent years to develop a tool to assess risk of developing cerebral palsy within the first months from simple video recordings. This paper provides an interesting step forward by using a general model for body point tracking from videos and explainable (pre-registered) features for the machine learning step.
Overall the paper is well written, however I have comments mainly on the presentation and discussion of the results.
In general I recommend the authors to align the paper with one of the current guideline and standard frameworks for AI in medicine (e.g. TRIPOD+AI checklist).
Title: "with video-based tracking" does not fully represent the method applied. I would be more specific in the title.
Abstract:
All information on the dataset should be provided in the abstract (i.e. cases excluded, distribution of positive/negative classification of GMA). Additional performance metrics of the model should be eventual provided, see comments within result section.
Background:
The last paragraph of the background reads as a summary of the method section, including results. I would expect objectives/research questions and hypothesis there.
Data description:
Data availability: I tried to find the calculated features on OSF but could only find the .csv of the different datasets including patient characteristics and GMA scores within the OSF Storage. Please provide the links for the feature dataset. I appreciate the preregistration of the features.
Analyses:
Was there any converting/preprocessing applied such as cropping, stabilization, rotation, scaling , cutting to the same length to the videos before skeletal tracking? Length and frequency of the videos could matter for some of the features, thus it should be stated how this is handled.
Feature relevance:
I would suggest to mention all 38 features within the paper (within a table or supplement) for the reader's convenience and also how these features relate to the clinical observations (i.e. variability, consistency, symmetry). Within Figure 3 the abbreviation of features are within the headings, however

these are very small and abbreviations are not explained within the legend. Also the x and y axis labels are missing in the figure.
Performance metric:
The authors should not only report the AUC, especially as this can be misleading in imbalanced dataset which is the case here (931/105). The recall of 0.34, should be mentioned in the main text and abstract and not only in the legend of Figure 4. I would also appreciate a confusion matrix of the locked-box set as this helps to interpret the results. The author should also discuss what the metrics mean in the current use case and discuss on setting appropriate classification threshold for risk assessment of cerebral palsy.
Discussion/potential complications:
To my view, within a risk screening assessment of cerebral palsy - maybe in the future widely applied in a general population- we are willing to accept a higher "false positive" rate with infants being extra checked by a specialist and with the possible consequence that more children get extra intervention/stimulation on motor development by a physiotherapist. I think we are less willing to accept "false negatives". However, this needs to be discussed with stakeholders within the field.
Efforts should be taken to realize data pooling from different groups working on automated GMA assessment and prevent data silos. I would encourage the authors to make data as far as possible available, as well as the trained models. Alternatively federated learning could be an option to strengthen the models for cerebral palsy risk assessment and should be explored in the future.

Methods

Are the methods appropriate to the aims of the study, are they well described, and are necessary controls included? Choose an item.

Conclusions

Are the conclusions adequately supported by the data shown? Choose an item.

Reporting Standards

Does the manuscript adhere to the journal's guidelines on minimum standards of reporting? Choose an item.

Choose an item.

Statistics

Are you able to assess all statistics in the manuscript, including the appropriateness of statistical tests used? Choose an item.

Quality of Written English

Please indicate the quality of language in the manuscript: Choose an item.

Declaration of Competing Interests

Please complete a declaration of competing interests, considering the following questions:

- Have you in the past five years received reimbursements, fees, funding, or salary from an organisation that may in any way gain or lose financially from the publication of this manuscript, either now or in the future?
- Do you hold any stocks or shares in an organisation that may in any way gain or lose financially from the publication of this manuscript, either now or in the future?
- Do you hold or are you currently applying for any patents relating to the content of the manuscript?
- Have you received reimbursements, fees, funding, or salary from an organization that holds or has applied for patents relating to the content of the manuscript?
- Do you have any other financial competing interests?
- Do you have any non-financial competing interests in relation to this paper?

If you can answer no to all of the above, write 'I declare that I have no competing interests' below. If your reply is yes to any, please give details below.

I declare that I have no competing interests

I agree to the open peer review policy of the journal. I understand that my name will be included on my report to the authors and, if the manuscript is accepted for publication, my named report including any attachments I upload will be posted on the website along with the authors' responses. I agree for my report to be made available under an Open Access Creative Commons CC-BY license (http://creativecommons.org/licenses/by/4.0/). I understand that any comments which I do not wish to be included in my named report can be included as confidential comments to the editors, which will not be published.

Choose an item.

To further support our reviewers, we have joined with Publons, where you can gain additional credit to further highlight your hard work (see: https://publons.com/journal/530/gigascience). On publication of this paper, your review will be automatically added to Publons, you can then choose whether or not to claim your Publons credit. I understand this statement.

Yes Choose an item.
