# Supplementary material for: A preregistered, open pipeline for early cerebral palsy risk assessment from infant videos
Source: Gigascience. 2026 Jan 20;15:giag003. doi: 10.1093/gigascience/giag003 (PMC13152017; doi:10.1093/gigascience/giag003)
Supplement: giag003_Reviewer_4_Report_original_submission — Reviewer 4 -- 2/8/2025 [file giag003_reviewer_4_report_original_submission.pdf]

Reviewer Report

Title: A Pre-Registered, Open Pipeline for Early Cerebral Palsy Risk Assessment from Infant Videos

Version: Original Submission **Date:** 2/8/2025

Reviewer name: Reviewer 4

Reviewer Comments to Author:

Summary
This paper proposes an automated method for predicting the risk of cerebral palsy in infants based on the General Movements Assessment. Videos of infants are recorded, from which 2D body keypoints are extracted using ViTPose. From the tracked keypoints, a set of features, which has been defined by clinicians, is extracted, which is then used to classify the infant as having present or absent fidgety movements.
The data set used consists of around 1000 videos, which were clinically assessed by trained GM experts. One part of the data set was defined as held out test set, and the remaining data was used to evaluate the method in a cross-validation manner.
Results showed an AUC of 0.79 for the held-out test set, with a true positive rate of around 80% at a false positive rate of around 25%. Cross-validation results were reported to lie in a similar range at an AUC of 0.73.
The code for the pipeline, including keypoint estimation, feature extraction, and classification is publicly available. For the used dataset, the computed features together with clinical scores are published, while the key point data is available on "reasonable request".
This paper tackles a very relevant topic, the automated early detection of CP, which could have a big impact, since it could enable wide-spread screening, and therefore detect more children with CP earlier and therefore lead to an earlier start of treatment and therapy.
The paper is well written and uses one of the largest datasets in automated CP detection literature. The experimental design is valid, and follows a strict process, by registering the method and a test set before running the experiments. The research community could benefit a lot from this large data set, since that is one of the things that has been lacking for a long time - a dataset on which researchers can compare their methods. Up to now, most methods have only been evaluated on private, small datasets, which made the interpretation of results difficult.
However, there are several things that should be improved in the paper.
Data set
More information about the data set needs to be added:
- Was the recording process standardized? Did the recorders get specific instructions?
- How was video recorded? From the top of the infant, from side view, at an angle, ...?
- Was the camera position static (on a tripod) or was it a handheld with potential moving?
- Were the infants lying on a table/floor?
- In case parents filmed the infants at home, was the recording setup the same as in the clinic?
Information about "two fully out-of-sample infant datasets" - same as above. Are there differences

between these datasets and yours?
Description of recordings lacking - camera position, handheld, top, front, side? Very relevant for 2D â†' generalization ability (lock-box set), description of other datasets used??
ViTPose
The claim that ViTPose has better accuracy than other models is made several times. However, there are no quantitative results backing up this claim. There is one example image, but I don't think this is enough to convince the reader.
"...selecting an open-source pose estimation algorithm that had high precision on our infant dataset."
"ViTPose-H [19] performed better than fine-tuned alternatives"
"The robustness and generalizability of ViTPose-H were validated through iterative review of pose estimates by clinicians trained in the General Movements Assessment (GMA). "
"This model was further tested on two fully out-of-sample infant datasets (n = 300, ages 0-4months), achieving consistent skeletal tracking performance."
"Overall we have shown that advances in pose estimation now make it entirely realistic to get precise movements from infant videos without the need for any specialized camera setup or finetuning."
- It sounds like evaluations have been made, but the experiments and their results need to be reported in detail.
- What does the "iterative review of pose estimates" mean? Did the clinicians perform GMA on visualized keypoints only?
- What did the "out-of-sample" infant datasets look like? What is different to your dataset?
More detail should be added to the processing of the videos. E.g. if there was some kind of video/keypoint normalization to handle different video resolutions or different distances to the camera (= different number of pixels per infant/different "size" in images), camera rotations, zooming or camera movement.
The post-processing sounds like it could significantly change the input data (interpolation, smoothing). Could you add some statistics on how many frames were excluded, the percentage of interpolated frames/joints, and how many outliers were removed? It could be also interesting to show a plot of a raw sequence of keypoint values vs. a post-processed one, so the reader can better understand how big the actual changes are.
Smoothing will always come with a loss of information. As is mentioned before, subtle differences could be relevant to distinguish between typically developing and high-risk infants. Could you comment on the effect of smoothing with respect to this? Is it really necessary to apply smoothing? Is there a lot of "jitter" in the keypoints?
Features
"A set of 38 movement features was derived from expert clinician input to ensure clinical relevance."
I don't think this statement is necessarily true. If the features selected by clinicians do not lead to good predictions, there is no clinical relevance. If some features, e.g., deep-learned ones without any connection to the clinician's features, perfectly solve the problem, then this is clinically relevant.
"By excluding any direct features related to GMA-specific fidgety movements, we aimed to ensure generalizability and avoid overfitting the model to the clinical dataset."
I don't really understand this reasoning. If FM-specific features work best, why not use them? You can still test for overfitting. It would be great to find features that work extremely well for the given dataset

(using cross-validation). If you show that they do, and then show that they don't generalize to other data sets, then you can go back to other features. But if you have features you think are best for the job, you should evaluate them.
It is mentioned that the (aggregated) feature vector that is used for classification is explainable, but it seems to me that the classifier is a black-box, and there is no way to tell based on which features/weights the classification was made. If this is possible, there should be an analysis of the predictions, regarding which combination of features is most important/can be trusted most.
The AutoML method to find a good classifier should be explained in more detail. From what I understand, it tests many different classifiers, preprocessing methods and different hyperparameters, and selects those that work best. It should be described in more detail which model and settings were selected for the experiments.
Results/discussion
It is mentioned several times, that the risk of overfitting is minimized by
* using a lock-box test set
* doing feature selection based on clinicians' opinion
* not using FM-related features
I think using a separate test set will not prevent overfitting, it will only make it visible. And I don't really see why the last two should minimize overfitting. Please elaborate.
"We can be confident that [...] it will generalize well to other datasets."
"...suggests that the model generalizes well to unseen data"
"We have shown [...] that our model generalizes well to unseen data."
There is not enough detail presented about the data sets to assess such statement. If, e.g., the camera angles of the used data sets are all similar, and the method is tested on a new video that was filmed from a completely different angle, I have doubts that it will actually generalize.
If you can show that the datasets are very diverse and cover all/most possible settings, this will be more convincing.
It would be interesting to check the results for such connections - e.g., if samples filmed from a specific angle are misclassified more often than others.
The paper puts a lot of emphasis on the "lock-box" test set (mentioned 11 times in text and figure captions). Could you elaborate more on how this set is different from the main data set? If there is no real difference (same population, same camera angles, same distribution of other properties) and it is just a randomly selected subset of the main data set (keeping the GM label ratio), how is it different from simply being an additional cross-validation split?
It is mentioned that "the difference between infants whose movements are typically developing and those that are not is often subtle." - still, a binary classifier is used, which does not account for any subtle differences. I think it would be beneficial to aim for a more fine-grained classification, so that the prediction could also provide some kind of confidence level. This will be much more valuable to clinicians, to know which predictions can be really trusted, and which are uncertain.
"Given that the clinician-selected movement features can predict GMA score in such a large sample, we have a strong indication that models that include even more data from a wider sample of infants, and more precise features, should perform even better."
Could you elaborate what you mean by "more precise features"? In literature, many people have used

all kinds of features for nearly 20 years, and nobody has found the best combination of features, yet. It is not clear to me why more data should improve the results. Particularly if the data is from a lower-risk population.
"we find that our machine learning predictions are highly predictive of the clinician-assessed GMA (AUC=0.79). Given its low cost, our video-based approach may be useful for clinical screening applications..."
From Fig. 4, selecting a true positive rate of roughly 0.8 will give a false positive rate of roughly 0.25. Calculating from the numbers in the dataset (820 samples FMs, 105 samples absent FMs), the model predicts ~289 infants with absent FMs, of which 84 are true positives and 205 are false positives. This seems far from being usable in a screening application, particularly given that in a low-risk population the number of infants with absent FMs will be even lower.
I think it would be interesting to dive deeper into the results analysis - do any specific patient characteristics correlate with the correctness of predictions? Or camera angles, recording length, etc... There is a lot more to find out, but this will be probably out of scope for this paper.
Additional comments/questions
* GMA standard recording length is recommended to be 3-5 minutes to my knowledge, why are only 1-2 minutes recorded? Is this considered enough by the clinicians to perform GMA?
* Fig. 4: Precision-Recall Curve is not mentioned in the text.
* "computed movement features have been made available on the OSF Pre-registration site." - Please add a link to the website where this can be found.
* Could you please state clearly the criteria for requests for the data set (keypoint data) to be considered as "reasonable"? The sentence "De-identified keypoint timeseries may be made available upon reasonable request." sounds like this is an arbitrary choice of the authors whether or not someone can get access. If the keypoint data is not really made available to the research community, this will greatly reduce the impact that this paper/data set can have for the field of automated CP detection.
Review Conclusion
This paper can have a significant impact on research on automated CP detection. In my opinion, several changes need to be made.
My suggestion would be to put the focus of the paper on the data set that will be made available. This is a strong contribution and can have a big impact on the field. (I would also change the title accordingly.)
I would put less emphasis on the feature extraction and classification method. There have been many methods using hand-crafted features, and the results do not seem like a major breakthrough. The method can serve a good baseline for future comparisons, particularly because it is also open-sourced.
In many parts, more detail needs to be added as mentioned above.
Overall, I think this paper can be an important step towards solving automated early detection of CP!

Methods

Are the methods appropriate to the aims of the study, are they well described, and are necessary controls included? Choose an item.

Conclusions

Are the conclusions adequately supported by the data shown? Choose an item.

Reporting Standards

Does the manuscript adhere to the journal's guidelines on [minimum standards of reporting?] Choose an item.

Choose an item.

Statistics

Are you able to assess all statistics in the manuscript, including the appropriateness of statistical tests used? Choose an item.

Quality of Written English

Please indicate the quality of language in the manuscript: Choose an item.

Declaration of Competing Interests

Please complete a declaration of competing interests, considering the following questions:

- Have you in the past five years received reimbursements, fees, funding, or salary from an organisation that may in any way gain or lose financially from the publication of this manuscript, either now or in the future?
- Do you hold any stocks or shares in an organisation that may in any way gain or lose financially from the publication of this manuscript, either now or in the future?
- Do you hold or are you currently applying for any patents relating to the content of the manuscript?
- Have you received reimbursements, fees, funding, or salary from an organization that holds or has applied for patents relating to the content of the manuscript?
- Do you have any other financial competing interests?
- Do you have any non-financial competing interests in relation to this paper?

If you can answer no to all of the above, write 'I declare that I have no competing interests' below. If your reply is yes to any, please give details below.

I declare that I have no competing interests

I agree to the open peer review policy of the journal. I understand that my name will be included on my report to the authors and, if the manuscript is accepted for publication, my named report including any attachments I upload will be posted on the website along with the authors' responses. I agree for my report to be made available under an Open Access Creative Commons CC-BY license (http://creativecommons.org/licenses/by/4.0/). I understand that any comments which I do not wish to

be included in my named report can be included as confidential comments to the editors, which will not be published.

Choose an item.

To further support our reviewers, we have joined with Publons, where you can gain additional credit to further highlight your hard work (see: https://publons.com/journal/530/gigascience). On publication of this paper, your review will be automatically added to Publons, you can then choose whether or not to claim your Publons credit. I understand this statement.

Yes Choose an item.
