# Supplementary material for: A preregistered, open pipeline for early cerebral palsy risk assessment from infant videos
Source: Gigascience. 2026 Jan 20;15:giag003. doi: 10.1093/gigascience/giag003 (PMC13152017; doi:10.1093/gigascience/giag003)
Supplement: giag003_Reviewer_4_Report_revision_1 — Reviewer 4 -- 6/6/2025 [file giag003_reviewer_4_report_revision_1.pdf]

Reviewer Report

Title: A Pre-Registered, Open Pipeline for Early Cerebral Palsy Risk Assessment from Infant Videos

Version: Revision 1 **Date:** 6/6/2025

Reviewer name: Reviewer 4

Reviewer Comments to Author:

Smoothing: The visualization in Fig 7 looks like a lot of information is lost and that it's likely that any fast motions will be smoothed out. This will most probably also affect the features and predictions. You write that you will most likely improve this in the future. Will the released features then be updated accordingly?

â€žA set of 38 kinematic features was selected based on clinician input [40, 41]," For references 40 and 41 it is unclear what kind of publications these are. Searching for them points to the OSF site, but there seems to be no additional information to that presented in the text regarding how the features are actually computed. Some details would be helpful, e.g., median velocity - is the velocity in each frame considered one measurement, and then the median value of all measurements is taken? This would not take into account non-movement periods. To understand this better, it would be good to have all the information on how the features are computed (without having to dig into the code).

The term â€žexplainable" features (reminds me of â€žexplainable AI"), means to me that it is possible to explain the reasoning why a test sample was scored the way it was. I don't mean to give overall importances for the separate features in the classifier, but explanations to each specific sample. E.g., infant sequence X is predicted as having absent FMs. Is it because of variability/complexity (entropy feature)? Or because of movement smoothness (IQR velocity)? Etc…

If it is possible to explain a classification like this, then the GM experts can go back and verify in the videos if these features actually capture what you assume they do. If such explanation is not possible, I would not use the term â€žexplainable" in connection to the classification.

â€žOur decision not to include features exclusively indicative of Fidgety Movements (FMs) relates more to our goal of developing a model with broader developmental applicability rather than directly minimizing statistical overfitting for the 3-4 month age window. FMs are powerful predictors within that specific timeframe. However, a model heavily reliant on FM-specific features would be inherently specialized to that narrow age range, limiting its utility for detecting risk at earlier or potentially later stages."

You argue that the features are aimed at predicting broad developmental status. However, your clinical scores/class labels are â€žpresent FMs" vs. â€žabsent FMs", and hence, your classifier is (supposedly) learning to predict the presence/absence of FMs. I don't think it's reasonable to assume that this approach will generalize to other age ranges/outcomes.

"What we showed is that our model generalized to a sequestered set of data that had never been seen by the processing pipeline, so in that sense it did generalize well to unseen data."

If the data hadn't been "unseen" at test time, this would not be a valid evaluation. Since it is drawn from the same distribution as the training set, I would not make a bold claim on the model's generalization

abilities.
I would consider the recordings done by parents at home to have at least some differences (even if the instructions were the same). So if the results on training with the data captured at the clinic and testing with parent videos is the same, this would convince me more that the model generalizes.
In the text, I would find it easier to read if GMA score 1/2/3 was replaced with something else, e.g. FMs present, FMs absent (, abnormal movements), or shorter FM-, FM+.
Not releasing the keypoints will definitely decrease the impact of the data set. I understand that this is not the author's choice, but I don't really understand how (normalized) keypoints are considered personally identifying information. Given the high barriers for getting access, I doubt that many people will actually be able to work with the keypoint data.
Gathering a large dataset of the (current) features doesn't seem like a promising avenue to me, due to the limitations that are also discussed in the paper/comments.
Overall, I still think there are enough contributions that justify publication.

Methods

Are the methods appropriate to the aims of the study, are they well described, and are necessary controls included? Choose an item.

Conclusions

Are the conclusions adequately supported by the data shown? Choose an item.

Reporting Standards

Does the manuscript adhere to the journal's guidelines on minimum standards of reporting? Choose an item.

Choose an item.

Statistics

Are you able to assess all statistics in the manuscript, including the appropriateness of statistical tests used? Choose an item.

Quality of Written English

Please indicate the quality of language in the manuscript: Choose an item.

Declaration of Competing Interests

Please complete a declaration of competing interests, considering the following questions:

- Have you in the past five years received reimbursements, fees, funding, or salary from an organisation that may in any way gain or lose financially from the publication of this manuscript, either now or in the future?
- Do you hold any stocks or shares in an organisation that may in any way gain or lose financially from the publication of this manuscript, either now or in the future?
- Do you hold or are you currently applying for any patents relating to the content of the manuscript?
- Have you received reimbursements, fees, funding, or salary from an organization that holds or has applied for patents relating to the content of the manuscript?
- Do you have any other financial competing interests?
- Do you have any non-financial competing interests in relation to this paper?

If you can answer no to all of the above, write 'I declare that I have no competing interests' below. If your reply is yes to any, please give details below.

I declare that I have no competing interests

I agree to the open peer review policy of the journal. I understand that my name will be included on my report to the authors and, if the manuscript is accepted for publication, my named report including any attachments I upload will be posted on the website along with the authors' responses. I agree for my report to be made available under an Open Access Creative Commons CC-BY license (http://creativecommons.org/licenses/by/4.0/). I understand that any comments which I do not wish to be included in my named report can be included as confidential comments to the editors, which will not be published.

Choose an item.

To further support our reviewers, we have joined with Publons, where you can gain additional credit to further highlight your hard work (see: https://publons.com/journal/530/gigascience). On publication of this paper, your review will be automatically added to Publons, you can then choose whether or not to claim your Publons credit. I understand this statement.

Yes Choose an item.
